# Altering micro-environments to change population health behaviour: towards an evidence base for choice architecture interventions

**DOI:** 10.1186/1471-2458-13-1218

**Published:** 2013-12-21

**Authors:** Gareth J Hollands, Ian Shemilt, Theresa M Marteau, Susan A Jebb, Michael P Kelly, Ryota Nakamura, Marc Suhrcke, David Ogilvie

**Affiliations:** 1 Behaviour and Health Research Unit, Institute of Public Health, University of Cambridge, Forvie Site, Robinson Way, Cambridge CB2 0SR, UK; 2 Department of Primary Care Health Sciences, University of Oxford, Oxford, UK; 3 The Centre for Public Health, The National Institute for Health and Care Excellence (NICE), London, UK; 4 Norwich Medical School, University of East Anglia, Norwich, UK; 5 School of Economics, University of East Anglia, Norwich, UK; 6 UKCRC Centre for Diet and Activity Research, Institute of Public Health, University of Cambridge, Cambridge, UK; 7 MRC Epidemiology Unit, University of Cambridge, Cambridge, UK

**Keywords:** Choice architecture, Nudge, Nudging, Behaviour change, Health behaviour

## Abstract

**Background:**

The idea that behaviour can be influenced at population level by altering the environments within which people make choices (choice architecture) has gained traction in policy circles. However, empirical evidence to support this idea is limited, especially its application to changing health behaviour. We propose an evidence-based definition and typology of choice architecture interventions that have been implemented within small-scale micro-environments and evaluated for their effects on four key sets of health behaviours: diet, physical activity, alcohol and tobacco use.

**Discussion:**

We argue that the limitations of the evidence base are due not simply to an absence of evidence, but also to a prior lack of definitional and conceptual clarity concerning applications of choice architecture to public health intervention. This has hampered the potential for systematic assessment of existing evidence. By seeking to address this issue, we demonstrate how our definition and typology have enabled systematic identification and preliminary mapping of a large body of available evidence for the effects of choice architecture interventions. We discuss key implications for further primary research, evidence synthesis and conceptual development to support the design and evaluation of such interventions.

**Summary:**

This conceptual groundwork provides a foundation for future research to investigate the effectiveness of choice architecture interventions within micro-environments for changing health behaviour. The approach we used may also serve as a template for mapping other under-explored fields of enquiry.

## Background

Changing patterns of behaviour to reduce the prevalence and burden of non-communicable diseases linked to poor diet, lack of physical activity, alcohol consumption and smoking is one of the most important global health challenges of the 21^st^ Century. Increasingly it is recognised that physical and social environments contribute greatly to these behaviours and, correspondingly, that altering these environments may be an important catalyst for change [1]. The idea that behaviour could be changed in predictable ways by changing the environments within which people make choices – choice architecture – is not new, having been a core focus of psychological and behavioural sciences over the past century [2]. It received considerable impetus from the publication in 2008 of the book ‘*Nudge: Improving decisions about health, wealth and happiness*’ [3], and has subsequently gained traction in policy circles [4,5]. However, empirical evidence to support the effectiveness of such approaches is limited [2], especially as it applies to interventions designed to change population health behaviour. We propose that this is not primarily due to an absence of evidence, but rather to a lack of definitional and conceptual clarity concerning applications of choice architecture to public health intervention. Without such clarity, attempts to develop and evaluate the effects of scalable interventions are severely hampered [6].

In health research, the term ‘choice architecture’ (and the related term ‘nudging’) has previously been broadly applied to refer to a range of intervention types across multiple behavioural and environmental contexts [2,3,7,8]. We focus here on one specific context that has been a core focus of the literature to date, namely interventions that involve altering small-scale physical and social environments, or micro-environments [9] — principally those within buildings such as restaurants, workplaces, homes and shops — to cue healthier behaviour. Following others, we consider micro-environments to be settings in which people may gather for specific purposes and in which they may acquire or consume food, alcohol, tobacco or be physically active [9]. For example, changing the size of plates, bowls or glasses, or placing less healthy foods further away from customers in a cafeteria, may influence the amounts and types of food selected and consumed [10]. Similarly, increasing the time taken for elevator doors to close may increase the likelihood of people using the stairs instead [11]. It is proposed that interventions of this kind typically require little conscious engagement on the part of the individual to realise their intended effects, mainly working via automatic or non-conscious psychological processes [2,12,13]. Being less dependent on recipients’ literacy, numeracy and self-regulatory skills, which are generally lower in those who are more deprived [14-16], they may also have the potential to reduce health inequalities [13].

### Defining choice architecture interventions

Prior work in this area has not included a clear definition of choice architecture interventions applicable to public health. In ‘*Nudge: Improving decisions about health, wealth and happiness*’ [3], Thaler and Sunstein define a ‘nudge’ as “any aspect of the choice architecture that alters people’s behaviour in a predictable way without forbidding any options or significantly changing their economic incentives”. In terms of operationalising this definition, they outline a set of mechanisms by which choice architecture can be altered to change behaviour, such as via incentives and defaults, but there are no precise operational definitions of what these terms mean in an applied sense [2]. Such definitions are instead largely implicit in the illustrative examples chosen, and apply to specific behavioural and environmental contexts that may have limited public health relevance. For example, possibilities for altering defaults are presented largely by reference to opt-in/opt-out systems such as for organ donation or pension schemes – examples not obviously applicable to key sets of health behaviours related to diet, physical activity, alcohol and tobacco use. Continued policy interest worldwide has stimulated efforts to clarify the concept, but to date this has been reflected only in non-systematic attempts to group intervention approaches that have common mechanisms and highlight examples of these approaches that may be scalable to population level [2,4,7,17,18]. A small number of recent studies in public health have examined choice architecture interventions as applied to health behaviours and conceptualised these as changes to environments to alter the ways in which choices are presented, taking into account that people’s choices are not always rational [19,20]. However, such examples, in this case being respectively a primary research study in a cafeteria environment [19] and a systematic review of interventions in self-service eating settings [20], focus on highly specific intervention contexts and so contain little examination of the broader conceptualisation of choice architecture interventions for changing health behaviour that we suggest is needed. The fact that terminology continues to be used inconsistently [6] emphasises the need for a more systematic approach to conceptual development.

### Towards a definition and typology of choice architecture interventions

To consolidate understanding and enhance conceptual clarity, we conducted a large-scale scoping review of evidence of the effects of choice architecture interventions within small-scale micro-environments on diet, physical activity, alcohol and tobacco use. The review had four aims:

1. To formulate an operational definition of choice architecture within micro-environments applicable to public health interventions.

2. To develop a provisional typology of such interventions.

3. To map the available empirical evidence for their effects on diet, physical activity, alcohol and tobacco use

4. To identify next steps for the development and evaluation of choice architecture interventions designed to change health behaviour at population level.

We consider each of these aims in the Discussion section. We developed, tested and refined our analysis through a systematic, iterative and configurative scoping process, a short summary of which is provided in Additional file 1. A detailed description of the methods used for the review is provided in the full report (Additional file 2) [21] and in a separate open-access methods paper [22].

## Discussion

### Defining choice architecture in micro-environments for changing health-related behaviour

We propose the following operational definition:

Interventions that involve altering the properties or placement of objects or stimuli within micro-environments with the intention of changing health-related behaviour. Such interventions are implemented within the same micro-environment as that in which the target behaviour is performed, typically require minimal conscious engagement, can in principle influence the behaviour of many people simultaneously, and are not targeted or tailored to specific individuals.

This reflects our focus on physical and social dimensions of micro-environments. In practice we did not encounter empirical studies of interventions that involve altering social dimensions of micro-environments (such as those centred on changing social norms [23]) that met our definition. In line with prior formulations of choice architecture [3], we excluded interventions that involved the use of economic instruments (e.g. taxes, subsidies), unless these also included other intervention components that met our operational definition. We conducted a concurrent scoping review of the effects of economic instruments on diet and physical activity, reported elsewhere [24]. Use of the term ‘typically require minimal conscious engagement’ reflects our view (consistent with that of others e.g. [20]) that interventions that alter micro-environments often display this characteristic [13,25], while also recognising the potential for varying degrees of conscious engagement with such interventions.

### Provisional typology of choice architecture interventions

**Figure 1:**
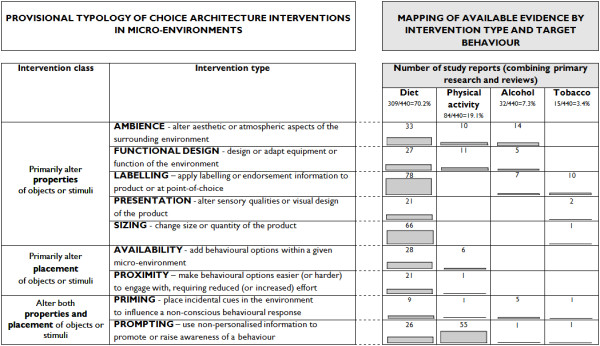
**Provisional typology of choice architecture interventions in micro-environments (left side) and mapping of available evidence (right side).** (NB Numbers include primary and secondary research reports featuring multiple intervention types and across multiple behaviours)

We grouped the available evidence that was consistent with our definition in the four domains of diet, physical activity, alcohol and tobacco use into nine types, each comprising a range of interventions that share similar characteristics or proposed mechanisms of action (Figure 1, left side). These nine intervention types can be aggregated into two higher-level classes of intervention: i. those that involve altering the *properties* of objects or stimuli within a micro-environment, and ii. those that involve altering their *placement* (with some interventions involving both). These two broad classes of intervention map well onto one of the more detailed models describing how environmental stimuli elicit behavioural responses outside of awareness [26].

### Mapping the available empirical evidence

This conceptual groundwork enabled the systematic identification and preliminary mapping of a large body of empirical evidence for the effects of choice architecture interventions for health behaviour change related to diet, physical activity, alcohol and tobacco use. The right side of Figure 1 shows the numbers of study reports we identified that met our operational definition, disaggregated by intervention type and target behaviour. Our evaluation of the text mining technologies used to expedite study selection (published elsewhere [22]) engenders confidence that the distribution of studies we assembled is likely to be largely representative of the full spectrum of available evidence for target interventions.

This evidence base is dominated by studies of interventions to change diet-related behaviours such as food purchasing and consumption (70.2% of study reports), with many fewer studies relating to physical activity (19.1%), alcohol (7.3%) or tobacco use (3.4%). The two types of intervention most frequently encountered, together accounting for over 40% of study reports, were those involving point-of-choice labelling, and prompting. These interventions generally involve providing information — about the nutritional content of food, for example, or the health benefits of climbing stairs — similar to that communicated in more conventional approaches to health promotion. Other types of intervention closer to the spirit of the formulated definition of choice architecture, i.e. less reliant on the conscious engagement of the individual, are less well represented in the literature. Further description of the nature of the studies identified for each intervention type is provided in Additional files 2 and 3.

### Implications and next steps for intervention development and evaluation

We identified a considerable volume and range of evidence for choice architecture interventions and their potential effects on four key health behaviours. We also found some notable gaps in the evidence base, including a lack of high-quality systematic reviews of the effects of interventions contained within the parameters of our typology (with exceptions, such as interventions to prompt stair use [27]). Most of the research evidence we identified focuses on the effects of interventions on behaviours related to diet. This imbalance in the distribution of evidence between the four sets of behaviours considered is perhaps surprising, given that most of the intervention types that comprise our typology could in principle be applied to alcohol and tobacco use, which (like eating) necessarily involve the consumption of products. The relative scarcity of evidence for impacts on physical activity may in part reflect the fact that some intervention types (such as product sizing) are less applicable to physical activity. We did, however, locate studies of interventions to promote physical activity in six of the nine intervention types identified. This highlights the potential to take interventions that have been applied in one behavioural domain and develop and test them in others. For example, interventions to alter the portion or package size of foods have been widely developed, but we found less evidence for similar interventions to alter the use of alcohol or tobacco products. This may be explained in part by the fact that diet-related behaviours provide a more diverse range of opportunities for intervention spanning a much larger range of products and environments, relative to alcohol or tobacco.

Limitations of the evidence base support our previous assertion that evidence to support altering choice architecture as a population health strategy is currently weak [2], but it is premature to conclude whether or not choice architecture and other potentially non-regulatory interventions are likely to be effective [6]. Formal critical appraisal and synthesis to estimate the direction (health-enhancing or not) and magnitude of effects reported in included studies was beyond the scope of this preliminary work. Further rigorous primary research and systematic reviews, conducted within a conceptually coherent framework, will be necessary to produce reliable assessments of the likely direction and magnitude of intervention effects, and of factors likely to moderate those effects.

This work provides a foundation for understanding the effects of a broad array of interventions to change health behaviour by altering micro-environments. The empirically grounded definition and typology of interventions are intended to inform and frame the scope of further investigations, as well as providing a platform for further conceptual development work. The definition and typology are both provisional and the typology is primarily descriptive. Additional development will be necessary before they can be applied to inform specific aspects of the design and evaluation of such interventions. In particular, ongoing work will need to compare and assimilate relevant theoretical and conceptual accounts of behaviour change interventions and processes with our provisional definition and typology. Such development would benefit from consideration of possible mechanisms of action, including the role of non-conscious processes in health-related behaviour [28,29]. It will also be necessary to monitor the emergence of new interventions proposed to alter choice architecture to change health behaviour. This will enable assessment of the extent to which their characteristics and proposed mechanisms of action are consistent with, or suggest further refinement of, the definition and typology.

Both the definition and typology were generated through systematic analysis of evidence for interventions that have not previously been evaluated within the specific context of this review. As such, they may not map neatly on to prior conceptualisations of choice architecture or nudging that have been proposed in the wider literature, encompassing various other fields of policy and practice. Despite challenges in translating the details of prior formulations of choice architecture to the different focus of this work, our definition is broadly consistent with their key principles. For example, Ploug and colleagues [8] characterise institutional strategies to shape behaviour based on choice architecture principles as: (1) imposition of trivial costs on those departing from welfare-promoting options, (2) the framing of options i.e. their presentation, and (3) setting of institutional default rules. Our definition and typology encompasses instances of (1), such as interventions altering proximity (e.g. placing less healthy foods further away); (2), such as nutritional labelling interventions; and (3), such as altering default portion sizes.

We found few intervention studies that explicitly linked proposed mechanisms of behaviour change with wider notions of ‘choice architecture’ or ‘nudging’. This lack of overlap between terms currently used in policy and research circles may reflect a time lag between these ideas becoming popular and studies of related interventions being completed and published. It may also signal a lack of conceptual clarity or agreed-upon terminology between different communities and research disciplines. The definition and typology we have presented here can therefore contribute to more fruitful translation between research and policy.

To improve population health and reduce health inequalities, we need to know not only the short-term effects of behaviour change interventions, but whether their effects — both singly and in combination— can be sustained and how these effects are distributed within and between social groups. Whilst we did not systematically extract data on this, our impression is that few studies have reported the long-term durability of behavioural effects of interventions. As many of these interventions rely on brief exposures in time and place, one-off or infrequent exposures to these interventions would not necessarily be expected to have enduring effects on behaviour. It is therefore important to investigate the cumulative effects of repeated exposure over time. This will require alteration of aspects of micro-environments within a long-term evaluative framework. As the evidence base develops, it will also be important to explore issues of implementation, such as public acceptability and financial impacts of interventions. Furthermore, whilst governments may be liable to present these interventions as non-regulatory approaches, it does not necessarily follow that regulatory or legislative frameworks will not be needed to ensure or enhance their implementation [2,30].

## Summary

We have argued that the prior lack of definitional and conceptual clarity concerning applications of choice architecture to public health intervention has hampered the potential for systematic assessment and synthesis of existing evidence for effects. We sought to address this issue through the development of an evidence-based definition and typology that can provide a foundation for future research. The approach we have used may also serve as a template for mapping other under-explored fields of enquiry, in particular those where intervention concepts and terminology may not be well-specified. We have identified significant requirements for further conceptual development, primary research and evidence synthesis to assess which choice architecture interventions are likely to be most effective in achieving sustained health behaviour change and reducing health inequalities. The extent to which their potential to change population behaviour can be realised on the scale needed to reduce the huge and growing burden of non-communicable disease awaits further study.

## Competing interests

The authors declare that they have no competing interests.

## Authors’ contributions

GJH contributed to study design, all stages of the scoping review (searches, study selection, data collection, data analysis, data interpretation) and co-led writing of the manuscript. DO contributed to study design and data interpretation, and co-led writing of the manuscript. RN contributed to study selection, data interpretation and writing of the manuscript. IS, TMM, SAJ, MPK, and MS contributed to study design, data interpretation and writing of the manuscript. All authors read and approved the final manuscript.

## Pre-publication history

The pre-publication history for this paper can be accessed here:

http://www.biomedcentral.com/1471-2458/13/1218/prepub

## Supplementary Material

Additional file 1 Summary of methods. Click here for file

Additional file 2 Full report on choice architecture scoping review. Click here for file

Additional file 3 Description of evidence by intervention type. Click here for file
