# Supplementary material for: Altering micro-environments to change population health behaviour: towards an evidence base for choice architecture interventions
Source: BMC Public Health. 2013 Dec 21;13:1218. doi: 10.1186/1471-2458-13-1218 (PMC3881502; doi:10.1186/1471-2458-13-1218)
Supplement: Additional file 1 — Summary of methods. [file 1471-2458-13-1218-S1.pdf]

Additional file 1 - Summary of methods

We conducted a large-scale, systematic scoping review of primary studies and review-level evidence of the effects of interventions and related conceptual material spanning several applied fields and disciplines [1]. Scoping reviews are typically conducted as a preliminary stage to systematic reviews, in order to map, delineate and describe an evidence base with boundaries and characteristics that are uncertain at the outset, rather than evaluating through aggregating such evidence, as exemplified by the use of meta-analysis to estimate pooled effect sizes [2-4]. A refined conceptual understanding of the material becomes an intended output of the scoping process rather than its starting point. They prioritise identifying a distribution of studies that is representative of the full spectrum of relevant evidence, whilst also assembling as many example studies as possible within available resources.

Preliminary indications that relevant studies would lack a common theoretical basis or vocabulary to describe interventions, coupled with our initially broad scope (with few eligibility criteria set for study designs, populations, comparators and outcomes) and multi-disciplinary focus, necessitated highly sensitive searches conducted in 15 electronic literature databases. Combined with records identified by parallel snowball searches, our electronic searches retrieved over 800,000 unique records. To prioritise these records for screening, we used text mining technologies, which automatically analyse text contained in a growing pool of screened study records [5] to identify patterns of key terms that distinguish eligible from ineligible records. We manually screened over 54,000 prioritised title and abstract records. Data extracted from 346 eligible full-text articles (including reviews) were used to configure and describe evaluative studies of interventions, including their design, intervention characteristics, outcome measures, and principal findings.

We report the numbers of studies identified by intervention type and target behaviour (Figure 1), give examples of each type of intervention, and provide summary statements of the reported effects of each type of intervention on behaviour (Additional file 3). Given the scale of the review, these statements are based on our reading of the original authors' conclusions only. In keeping with a scoping review, we did not conduct a critical appraisal of the primary studies and formal synthesis of the direction (health-enhancing or not) and magnitude of the reported effects.

The definition and typology we present reflect an iterative process of discussion and analysis of the evidence, in which we repeatedly tested and refined our positions against newly encountered material and validated these decisions in consultation with a multi-disciplinary group of internal and external experts.

Further details of the methods used in the review can be found in the full report, available as Additional file 1, and in a separate open-access methods paper [6].

References

1. Hollands GJ, Shemilt I, Marteau TM, Jebb SA, Kelly MP, Nakamura R, Suhrcke M, Ogilvie D: **Altering choice architecture to change population health behaviour: a large-**

scale conceptual and empirical scoping review of interventions within micro-environments. Cambridge: University of Cambridge; 2013.

2. Levac D, Colquhoun H, O'Brien K: **Scoping studies: advancing the methodology**. *Implement Sci* 2010, **5**(1):69.

3. Gough D, Thomas J: **Commonality and diversity in reviews**. In *An Introduction to Systematic Reviews*. Edited by Gough D, Oliver SJT. London: Sage Publications Ltd.; 2012:35-66.

4. Gough D, Thomas J, Oliver S: **Clarifying differences between review designs and methods**. *Syst Rev* 2012, **1**(1):28.

5. Thomas J, McNaught J, Ananiadou S: **Applications of text mining within systematic reviews**. *Res Synth Methods* 2011, **2**(1):1-14.

6. Shemilt I, Simon A, Hollands GJ, Marteau TM, Ogilvie D, O'Mara-Eves A, Kelly MP, Thomas J: **Pinpointing needles in giant haystacks: use of text mining to reduce impractical screening workload in extremely large scoping reviews**. *Res Synth Methods* 2013. DOI: 10.1002/jrsm.1093.
