# Supplementary material for: Altering micro-environments to change population health behaviour: towards an evidence base for choice architecture interventions
Source: BMC Public Health. 2013 Dec 21;13:1218. doi: 10.1186/1471-2458-13-1218 (PMC3881502; doi:10.1186/1471-2458-13-1218)
Supplement: Additional file 2 — Full report on choice architecture scoping review. [file 1471-2458-13-1218-S2.pdf]

December 2013

Altering choice architecture to change population health behaviour: a large-scale conceptual and empirical scoping review of interventions within micro-environments

Gareth J. Hollands, Ian Shemilt, Theresa M. Marteau*, Susan A. Jebb, Michael P. Kelly, Ryota Nakamura, Marc Suhrcke, David Ogilvie

The Behaviour and Health Research Unit (BHRU) is funded by the Department of Health Policy Research Programme as the Policy Research Unit in Behaviour and Health (PR-UN-0409-10109). The views expressed in this paper are those of the authors and not necessarily those of the Department of Health.

*Contact author: theresa.marteau@medschl.cam.ac.uk

Contents

	Page
**Executive Summary**	**3**
**Introduction**	**7**
**Methods**	**12**
**Results**	**18**
**Discussion**	**34**
**Acknowledgements**	**42**
**References**	**43**
- *References from report*	*43*
- *Included articles by intervention type*	*50*
- *Included articles by behaviour*	*88*
- *Included articles by intervention type and behaviour*	*120*
- *Excluded articles*	*160*
- *Articles identified but not assessed*	*175*
**Appendix 1: Search strategies, dates and yields**	**188**

How to cite this report:

Hollands, G. J., Shemilt, I., Marteau, T. M., Jebb, S. A., Kelly, M. P., Nakamura, R., Suhrcke, M., Ogilvie, D. (2013). Altering choice architecture to change population health behaviour: a large-scale conceptual and empirical scoping review of interventions within micro-environments. Cambridge: University of Cambridge.

Boxes

Page

Box 1. Operational definition of choice architecture interventions in micro-environments 19

Tables

Page

Table 1. Numbers of study reports identified by intervention type and target behaviour 26

Table 2. Distribution of primary and secondary research reports 27

Table 3. Intervention types identified and brief summaries of respective evidence 28

Figures

Page

Figure 1. PRISMA flow diagram 19

Figure 2. Emergent typology of intervention types 25

Executive Summary

Background

There is considerable public policy interest in choice architecture, an approach that involves altering features of physical or social environments to change behaviour. To date there has been no systematic attempt to clarify the concept and definition or describe the evidence base for such interventions. We conducted a systematic scoping review of empirical evidence, and related conceptual material, to identify the effects of choice architecture interventions in micro-environments on diet-, physical activity-, alcohol- and tobacco-related behaviours.

Methods

Highly sensitive systematic electronic searches were conducted in parallel with snowball searches because of the variation in terms used to describe choice architecture. Retrieved records were prioritised for manual screening using novel computer-aided approaches. A core dataset was extracted from eligible full-text articles to inform both assessment of the existing empirical evidence and concurrent conceptual work.

Results

804,919 unique records were retrieved, with 54,651 abstracts manually screened for eligibility. 346 full-text articles were included in the final review.

We developed an operational definition of choice architecture interventions in micro-environments for changing health-related behaviour as follows:
'Interventions that involve altering the properties or placement of objects or stimuli within micro-environments with the intention of changing health-related behaviour. Such interventions are implemented within the same micro-environment as that in which the target behaviour is performed, typically require minimal conscious engagement, can in principle influence the behaviour of many people simultaneously, and are not targeted or tailored to specific individuals.'

Most (70.2%) of the relevant primary research and reviews had been undertaken in relation to diet-related behaviours, compared to 19.1% for physical activity, 7.3% for alcohol and 3.4% for tobacco. We categorised studies according to an emergent typology of nine categories of micro-environmental interventions: sizing; presentation; labelling; functional design; ambience; proximity;

availability; prompting; priming. These categories were further grouped into broader classes according to whether they primarily involved altering the properties of objects or stimuli, their placement, or both. The categories with most study reports were labelling (primarily in relation to point-of-choice labelling and diet, representing ~22% of total reports), and prompting (primarily in relation to motivational prompts for stair use, representing ~19% of total reports). Notably, these types of interventions were the ones most closely related to more traditional information-giving approaches to eliciting behaviour change. Beyond these examples, few types of intervention had been examined in substantive, consistent bodies of apparently similar studies. In most categories, studies were heterogeneous with respect to the populations, interventions, comparators or counterfactuals, outcomes and moderators assessed. Although a number of non-systematic reviews and broad overviews had attempted to summarise parts of the overall evidence base, few high-quality systematic reviews were identified.

The available evidence as reported by study authors (but not critically appraised in this review) is summarised by intervention category, as follows:

Interventions that primarily alter ***properties*** *of objects or stimuli*

- **Ambience** (e.g. aesthetic improvements): a wide variety of interventions was identified, mostly studied in field trials with a variety of outcomes. The majority of studies reported an effect of the intervention on behaviour
- **Functional design** (e.g. size or shape of food and drink receptacles): a wide variety of interventions and study outcomes was identified. There was no consistent overall pattern of findings, with some studies reporting an effect of the intervention on behaviour and others reporting no effect
- **Labelling** (e.g. point-of-choice nutritional labelling): most primary research involved field trials, with many studies reporting multiple outcomes. There was no consistent overall pattern of reported findings (as above)
- **Presentation** (e.g. packaging design): a wide variety of interventions were identified. Most primary research was laboratory-based. There was no consistent overall pattern of findings (as above)
- **Sizing** (e.g. food and drink portion sizing): these were mostly laboratory-based studies, with the majority reporting an effect of portion size on consumption, a conclusion that is also supported by specific systematic review evidence.

Interventions that primarily alter ***placement*** *of objects or stimuli*

- **Availability** (e.g. increasing healthier food options): primary studies were identified in diet and physical activity only, mostly studied in field trials concerning food and drink options in restaurants and vending machines and the use of stairs or lifts. The majority of studies reported an effect of the intervention on behaviour, although this was often complicated by the concurrent implementation of multiple interventions or the assessment of multiple outcomes
- **Proximity** (e.g. altering proximity of options by changing layouts): all primary studies identified related to dietary behaviours, with a variety of outcomes reflecting changes in consumption, purchasing or selection of products. The majority of studies reported an effect of the intervention on behaviour.

Interventions that alter both ***properties and placement*** *of objects or stimuli*

- **Prompting** (e.g. motivational prompts for stair versus lift or escalator use): primary studies were identified within all four behavioural domains, using field-based (mainly time-series) designs. In relation to physical activity, the majority of studies reported an effect of the intervention on behaviour, supported by specific systematic review evidence. In relation to diet, many studies reported multiple outcomes and there was no consistent overall pattern of findings (as above)
- **Priming** (e.g. introduction of incidental cues to behaviour): primary studies of a wide variety of interventions were identified within all four behavioural domains, with a variety of outcomes. The majority of studies reported an effect of the intervention on behaviour.

Within most of these categories at least some of the available research concerned the effectiveness of interventions in *increasing* the purchase or consumption of a *less healthy* product, rather than in promoting health-enhancing behaviours.

Implications for research

This scoping review has identified two clear gaps in the evidence base that could be addressed through original primary research:

i) Interventions to change physical activity-related behaviours within micro-environments: the majority of primary research studies within the physical activity domain concerned interventions to prompt stair use, suggesting that opportunities for primary research in under-represented areas merit further attention

ii) Interventions to change alcohol- and tobacco-related behaviours within micro-environments: research on interventions to change these behaviours comprised only ~11% of the total studies identified, and these did not make a substantial contribution to the available evidence in any of the nine intervention categories (relative to other behavioural domains).

There is clear scope for further and more rigorous evidence synthesis in at least three areas of the evidence base:

i) Interventions that primarily alter the properties of objects or stimuli within micro-environments: all of the intervention categories grouped under this heading (ambience, functional design, labelling, presentation and sizing) had either not been the subject of systematic reviews, or those systematic reviews were in some way limited in their quality or relevance
ii) Interventions that primarily alter the placement of objects or stimuli within micro-environments: we identified no systematic reviews relating to the 'proximity' category of interventions
iii) Interventions that target automatic psychological processes: we identified no systematic reviews relating to the 'priming' category of interventions.

We also identified a need for more conceptual work to develop taxonomies or typologies of population-level interventions underpinned by a theoretical understanding of behaviour change processes. It is also important that future work should recognise and examine issues relating to the durability of intervention effects and the distribution of such effects between social groups, and hence their potential to reduce health inequalities.

Conclusion

This scoping review has made the following three key contributions to the evidence base:

i) Development of a definition and a provisional typology of choice architecture interventions in micro-environments
ii) Description of a large body of relevant primary and secondary research
iii) Identification of significant opportunities for further primary research and evidence synthesis as well as conceptual work to contribute to international efforts to change behaviour to improve population health and reduce health inequalities.

Introduction

Non-communicable conditions, principally cardiovascular diseases, diabetes, cancers, and chronic respiratory diseases, accounted for an estimated 36 million deaths worldwide in 2008 (56% of all deaths globally) and in the UK accounted for 83% of years of life lost due to premature death (WHO, 2011). These conditions are strongly related to potentially modifiable patterns of behaviour. Achieving health behaviour change is clearly important but is also difficult (NICE, 2007). To this end, there is a continuing public health imperative to develop effective interventions to promote behaviour change.

Interventions to change health behaviour have traditionally been developed in accordance with models of rational decision-making, viewing human actions as reasoned, conscious and intentional acts requiring an individual's volitional control. Many theoretical models of health-related decisions and behaviour emphasise the importance of the rational cognitive appraisal of presenting stimuli in determining a person's response (e.g. Schwarzer, 2001; Rogers, 1983; Ajzen, 1991). It follows from such models that providing information that allows the recipient to reflect on behavioural options that are beneficial to health may be sufficient to bring about change. In many contexts, however, empirical evidence indicates that the effectiveness of behavioural interventions that deliberately target rational or reflective decision-making, for example by giving information, is limited (Albarracin et al, 2005; WHO, 2008). In addition, significant discrepancies have consistently been found between individuals' behavioural intentions and their actual behaviour (Webb & Sheeran, 2006). One proposed explanation for this is that there are considerable influences on behaviour outside of the individual's control. This has led to increasing policy and research attention on other mechanisms key to understanding behaviour and behaviour change, such as the role of automatic or non-conscious cognitive, emotional and behavioural processes, and the effect of the environment in shaping people's actions (Marteau et al 2011; Marteau et al, 2012).

In recent years, a more comprehensive approach to understanding behaviour change has been adopted, with the development of dual-system models of cognition and behaviour (e.g. Strack & Deutsch, 2004; Gawronski & Bodenhausen, 2006; Hofmann et al, 2008). Such models propose two systems of information processing; one consisting of the aforementioned processes of reflective, syllogistic reasoning, and requiring cognitive capacity; and the other consisting of the automatic or non-conscious processes of learned associations, giving rise to behavioural impulses. The latter are characterised by their relation to specific situational and environmental cues and the inclusion of a

behavioural urge to approach or engage with a stimulus, thus limiting or directing behaviour beyond immediate conscious control (Bargh & Morsella, 2009). Coexisting with this shift in theoretical understanding, is a recognition that individual-level factors are rarely sufficient to explain human behaviour and that physical and social environments directly and indirectly shape our behaviour and thus health outcomes. This awareness is manifest in explicit recognition of these processes within both the primary and secondary research literature and within conceptual frameworks that attempt to represent determinants of human behaviour and/or health (e.g. Dolan et al, 2010; Michie et al, 2011; Solar & Irwin, 2010; Dahlgren & Whitehead, 1991; Bonnefoy et al, 2007). These developments provide the context for an emergence of interest in interventions targeting these mechanisms to improve individual and population health and well-being.

Choice architecture and nudge interventions

One such approach is that of so-called ‘choice architecture’ (or ‘nudge’) interventions[1]. These terms have been used to refer to interventions that involve altering physical or social environments or settings to cue healthier behaviour, principally via the engagement of automatic cognitive processes. These ideas have been brought to the forefront of public awareness in recent times through the work of Thaler and Sunstein (Nudge: Improving decisions about health, wealth and happiness, Thaler & Sunstein, 2008) and whilst the ideas (and their related terminology) remain contentious and have been the focus of considerable debate in the specialist and mainstream media, they have proven influential within the UK and US governments in regards to behavioural intervention and public health policy (MINDSPACE, Dolan et al, 2010; Cabinet Office Behavioural Insights Team, 2010,2011). It is this public policy interest, in conjunction with the emergence of key scientific questions, that underpins this review.

Objectives

The objectives of this scoping review were to:

- to formulate an operational definition of choice architecture within micro-environments applicable to public health interventions;
- to develop a provisional typology of such interventions;
- to map the available empirical evidence for their effects on diet, physical activity, alcohol and tobacco use; and

[1] We note that the increasing interest in these interventions cannot be attributed solely to shifts in theoretical understanding or a belief that such interventions may be more effective than existing options, but is also determined by issues of political acceptability (as where possible, governments and the public may prefer ‘light-touch’ intervention rather than legislation and regulation), feasibility, and likely cost (such interventions, at least as commonly perceived, may be considered inexpensive and easily implemented without complex legislative or regulatory processes).

- to identify next steps for the development and evaluation of choice architecture interventions designed to change health behaviour at population level.

Defining the topic area

In order to adequately describe and assess a body of evidence, some level of definitional clarity is required. The term 'choice architecture' is typically used to refer to the ways in which choices or behavioural options are presented, and their influence on behaviour. This principle is central to the definition of a 'nudge', which according to the eponymously-titled book (Thaler & Sunstein, 2008) is "any aspect of the choice architecture that alters people's behaviour in a predictable way without forbidding any options or significantly changing their economic incentives". However, there is no precise operational definition of what these terms actually mean in an applied sense (Marteau et al, 2011) and in relation to our specified behavioural and environmental context. Although Thaler and Sunstein provide some definitions of key terms and concepts, the principal aim of their book is to outline a specific political philosophy and broad approach to intervention, rather than to provide a conceptual framework for research. While subsequent policy-oriented work has linked these principles to real-world examples, we are not aware of any explicit attempt to systematically conceptualise or categorise interventions or their components in detail, and the terminology continues to be used inconsistently (House of Lords, 2011).

The lack of clear definition is problematic for any attempt to review existing evidence, because if we take the initial definition at face value, then all interventions intended to impact on behaviour, other than legislation, regulation and changing economic incentives[2], have components that could be regarded as potentially relevant. This would encompass a broad spectrum of research and present a practically insurmountable challenge with respect to scoping and describing the relevant empirical evidence. In addition, any review undertaken on such a scale and including numerous types of interventions would necessarily duplicate or overlap with the content of many existing evidence syntheses. Clearer delineation of our sphere of primary interest would enable us to better contextualise, structure and interpret the empirical research identified in the scoping review.

Placement within a conceptual framework

Choice architecture interventions are principally intended to be delivered at the population level,

[2] This wording is in line with Thaler and Sunstein's original formulation, but we note that most economists would technically consider many choice architecture interventions as changing economic incentives, because they impact on opportunity costs. As such, we have opted to use the term 'fiscal policy intervention' elsewhere in this review.

whereas efforts to map the content of behaviour change interventions have typically been limited to the individual- or group-level perspective (e.g. Abraham & Michie, 2008; Michie et al, 2011; Lowe et al, 2011) or focused on a relatively narrow range of interventions or behaviours (Wansink, 2004; Sobal & Wansink, 2007; Stroebele & De Castro, 2004; Turley & Milliman, 2000). For the current review, we required a broader consideration of the entire range of interventions possible within our definitional scope, and across multiple health behaviours. While frameworks from the conceptual literature are available to help structure the task (e.g. Thaler & Sunstein, 2008; Dolan et al, 2010), they may not be suitable for this purpose because they lack clear definitions, representative exemplars and evidence of systematic development. There are, however, some general organisational frameworks of determinants of behaviour and health which help provide some conceptual clarity.

For example, the ANGELO framework, originally developed in relation to obesity, proposes two key dimensions along which to conceptualise environmental factors: environmental scale (micro-environmental 'settings' and macro-environmental 'sectors', the former being geographically distinct, small-scale environments in which groups of people gather for specific purposes and activities, such as shops, schools, and homes) and type (physical, economic, political and sociocultural) (Swinburn et al, 1999). Based on current knowledge, we anticipated that most choice architecture interventions could be regarded as forming a subset of interventions within physical micro-environments.

Empirical component of the review

The second key component of the scoping review was an assessment of the available primary and secondary research on the impact of choice architecture interventions on health behaviour. Although there are non-systematic overviews of relevant evidence (e.g. Marteau et al, 2011; Dolan et al, 2010; Thaler & Sunstein, 2008), we were not aware of any systematic, comprehensive reviews explicitly framed in relation to the concept of choice architecture. Whilst there is a large body of systematic and non-systematic review evidence of the effects of a wide variety of environmental factors and interventions on health behaviour and outcomes (e.g. Faith et al, 2007; French et al, 2001; Matson-Koffman et al, 2005; de Vet et al, 2011; Davison & Lawson, 2006), these reviews have typically addressed the effects of much narrower, or much broader, classes of intervention, generally only within a solitary behavioural domain. This review aimed to address the call for greater conceptual clarity to support the development of a robust evidence base for 'environmental interventions' (Sallis et al, 1998; Kirk et al, 2010) by adopting a relatively narrow

focus on interventions that alter choice architecture within micro-environments, while systematically mapping the available evidence across multiple domains of health behaviour. The expectation was that this process would also allow us to identify specific research questions for further in-depth systematic review or primary research.

Given the lack of conceptual and definitional clarity outlined above, any attempt to map this evidence required a set of highly sensitive literature searches which are liable to produce extremely large records sets that it would not be practicable to screen using conventional methods. We therefore developed a novel approach to the searching and screening process, outlined in the Methods section.

Methods

Criteria for considering studies for this review

Types of studies

Studies that described empirical research of any design (primary or secondary) were eligible for consideration, as were articles that provided highly relevant conceptual or theoretical material such as descriptions or explanations of terms or concepts pertaining to nudge or choice architecture and applied within a behavioural intervention context.

Types of participants

Studies were eligible for inclusion irrespective of their focus on individuals, dyads, families, households, organisations, areas or communities. We applied no restrictions on geographical or social setting of the study, or the age, health or clinical characteristics of participants.

Types of interventions

Preliminary analysis of relevant literature in conjunction with subsequent cycles of discussion and development within the review team led to a provisional working definition of choice architecture within micro-environments:

'Interventions that have the potential to change health-related behaviour and are implemented within the same micro-environment as that in which the targeted behaviour is enacted.'

Initial research suggested that relevant studies would lack a common theoretical basis or vocabulary to describe interventions and would not allow us to capture the intervention concept satisfactorily in any database search strategies. We therefore adopted extremely broad search strategies that focused on the broad nature of potentially relevant manipulations and obviated the need to rely on presence of specific terms or labels such as "choice architecture" or "micro-environments". We did include a range of terms that would potentially reflect alterations to spatial or quantitative properties of environments, or indicate studies of the effects of interventions (e.g. change$, alter$, add$, decreas$).

As the review proceeded, the definition was developed and refined iteratively through identifying, discussing and clarifying specific 'boundary issues' as they arose. Any changes to operational

definitions were agreed by consensus and documented to ensure transparency in the selection of studies for inclusion. Three key developments were as follows:

1) The above provisional working definition was initially supplemented with explicit reference to automatic or non-conscious psychological processes, whereby an additional condition to be met by eligible interventions was that they targeted these processes. However, it was agreed, upon considering extant literature and discussion between the review team, that the majority of studies would not explicitly reference such underlying theoretical concepts, meaning assessment would be overly subjective and complex to implement. A similar term was included in our final definition because it reflected a concept common to prior research on choice architecture, but it did not need to be met for inclusion.

2) The above provisional working definition was initially supplemented with the criterion that interventions would be excluded should they contain significant levels of information provision. It was agreed that this was not a workable exclusion criterion, given that visual, as well as verbal and textual information is communicated via changes to the environment and its amount is not readily assessed.

3) The above provisional working definition was initially supplemented with the criterion that eligible interventions are in principle scalable for implementation at population level. It became clear that the term scalable is open to a number of interpretations and potentially applies to all interventions, and that a more workable operationalisation was that interventions are not targeted or tailored to specific individuals and can influence the behaviour of many people simultaneously.

We also consulted with external experts to discuss and validate our developing conceptual positions and definitions. This process centred around a one-day expert workshop organised midway through the scoping process. Those who attended the expert workshop or also provided additional feedback and discussion were as follows:

- Dr. Amy Ahern, MRC Human Nutrtion Research, Cambridge, UK (psychology, nutrition)
- Professor John Frank, Scottish Collaboration for Public Health Research and Policy, University of Edinburgh, UK (public health and policy)
- Professor Simon Griffin, MRC Epidemiology Unit, Cambridge, UK (epidemiology, public health)
- Michael Hallsworth, Institute for Government, UK (policy)

- Dr. Vivien Hendry, Centre for Diet and Activity Research, University of Cambridge, UK (public health)
- Professor Ann Louise Kinmonth, Primary Care Unit, University of Cambridge (primary care)
- Dr. Rachel Pechey, Behaviour and Health Research Unit, University of Cambridge, UK (psychology)
- Professor Mark Petticrew, London School of Hygiene and Tropical Medicine, UK (evidence synthesis, policy)
- Dr. Andrew Prestwich, University of Leeds, UK (psychology)
- Dr. Catherine Swann, National Institute for Health and Clinical Excellence, UK (public health and policy)
- Professor Daniel Zizzo, University of East Anglia, UK (economics)

The core review team's disciplinary background is characterised as follows:

Health psychology (Hollands, Marteau), evidence synthesis (Shemilt), public health (Ogilvie), public health and policy (Kelly), nutrition (Jebb), economics (Nakamura, Suhrcke).

Further inclusion/exclusion criteria for interventions were guided by the scope of previous conceptualisations of choice architecture and practical considerations. For example, consistent with Thaler and Sunstein (Thaler and Sunstein, 2008), interventions involving legislation, regulation or fiscal policy interventions were included only if they also featured components that met the operational definition of choice architecture interventions. Furthermore, we were already conducting in parallel a scoping review of the effects of the economic environment on diet and physical activity (Shemilt et al, 2013). Mass-media interventions, interventions with content personalised to the recipient, brief interventions in primary care (NICE, 2006), and interventions that involved major changes to existing physical infrastructure were excluded, irrespective of the nature of other intervention components. Interventions involving 'choice editing' (the addition or removal of behavioural options) were included providing equivalent options or behaviours from the previous potential behaviour set remained available within the micro-environment[3].

[3] This is only a guiding principle, with its application being dependent on the level of explanation regarding the equivalence of available behaviours or options. For example, in relation to dietary behaviour, removing several types of a given food category e.g. confectionery, but still leaving the available option of another food from that category, may not be regarded as significantly restricting the available options. Alternatively, an equivalent available option may be regarded as needing to be more (having the option to eat a specific type or brand of chocolate bar) or less specific (having the option to eat any high-sugar food).

See 'Results' for our final working definition.

Types of outcomes

The primary outcomes were engagement in behaviours related to diet, physical activity, or the use of alcohol or tobacco. Included studies needed to report a behavioural outcome relating to food, alcohol or tobacco consumption or the performance of physical activity, or a measure of a proximal direct consequence (e.g. dietary intake, energy expenditure, tobacco or alcohol intake) or determinant of such behaviour (e.g. food, alcohol or tobacco purchasing).

Search methods

We searched the following electronic literature databases, with no date restrictions: MEDLINE; Embase; PsycINFO; Cochrane Database of Systematic Reviews; Database of Abstracts of Reviews of Effects; Health Technology Assessment Database; Database of Promoting Health Effectiveness Reviews (DoPHER); Social Sciences Citation Index; Science Citation Index Expanded; Applied Social Sciences Index and Abstracts (ASSIA); SPORTDiscus; EconLit; NHS Economic Evaluation Database (NHS EED). We also searched grey literature databases (Conference Proceedings Citation Index – Science; Conference Proceedings Citation Index – Social Science & Humanities) and organisational websites including those of the World Health Organization, National Institute for Health and Clinical Excellence (UK) and Agency for Health Care Research and Quality (USA). Search strategies are included as Appendix 1.

Snowball search techniques (Greenhalgh & Peacock, 2005) (i.e. searching reference lists and electronic citation tracking from published reports within a corpus of eligible studies) and personal contacts with colleagues and through our academic research networks were used to identify further potentially eligible studies.

Data collection and analysis

In this section we describe the key elements but for further details please see a complementary methods paper (Shemilt et al, 2013). Data collection and analysis were managed using EPPI Reviewer 4 systematic review software (Thomas et al, 2010). Records retrieved were imported into the software for title and abstract screening by two reviewers (GJH and RN). Studies judged potentially eligible for inclusion at this stage were grouped using pre-specified rules into three categories (A, B and C) according to the probability of final inclusion: category A (those judged to have a high probability of meeting inclusion criteria); category B (those judged likely to be excluded, but requiring careful consideration because the intervention was close to a borderline of

eligibility criteria with respect to interventions or outcomes); and category C (those judged likely to be excluded but with insufficient information in the title and abstract to warrant their definitive exclusion).

We initially screened a random sample of titles and abstracts to estimate the baseline inclusion rate (BIR). The BIR is a figure that represents the frequency at which we could expect to find eligible records in the complete record set if we were able to screen it in its entirety. This allowed us to gauge our overall progress and the performance of our methods in identifying eligible records at a better than random chance. For the substantive body of the title and abstract screening process we employed novel software-based methods to increase the efficiency of this work by prioritising our screening of the overall record set. These methods comprised a 'prioritisation method', which employs text-mining methods to order the large record set, drawing on words frequently used in a researcher-specified initial corpus of eligible studies to cluster the record set; and a 'classification method', which employs machine learning to prioritise screening based on a large researcher-specified list of terms indicative of inclusion or exclusion. The use of multiple methods reduces the likelihood of only certain types of studies being identified (and of this becoming ever more likely as the reference body of studies increases in size). Concurrently, we also screened records identified through snowball searching.

From screened records determined to fall in category A, we obtained full-text reports (where possible), completed a further stage of full-text screening to confirm the inclusion of each study, and extracted a core dataset from each paper. This consisted of the citation; year of publication; study design; target behavioural domain; study population; details of the intervention; the primary outcome measure(s); and principal findings. These data were then used in a range of analyses including the production of tabulated and textual summaries of included studies, drawing on narrative and meta-narrative synthesis approaches (Greenhalgh et al, 2005; Rodgers et al, 2009) to map the available evidence and inform the iterative development of a typology of the interventions of interest.

Typology development

Having extracted a description of the intervention from all included studies, on multiple occasions we formed a list of all interventions encountered up to that point, at a relatively detailed level of explanation. We then looked for commonalities between similar interventions and considered forming broader categories based on emergent clusters of intervention types. We took a relatively cautious approach to reducing the level of granularity in this way, testing the capacity of the

emergent typology to accommodate new material as it was encountered and reaching consensus through internal and external discussion and expert consultation.

Three examples of this process are as follows. For each, it was felt that the similarity in form of these interventions outweighed differences in their potential content, and reflected a coherent category in the context of attempting to broadly describe the evidence base.

1) The intervention category ‘labelling’ combines what were originally five individual categories, those being: labelling (nutrient claims); labelling (reference portion); labelling (nutritional labelling); labelling (health warning labels); endorsements on packaging.

2) The intervention category ‘sizing’ combines what were originally three individual categories: portion sizing; unit sizing; package sizing.

3) The intervention category ‘proximity’ combines what were originally two individual categories: altering proximity of behavioural options; altering visibility of behavioural options.

Further methodological considerations

We formulated approximate stopping rules for the scoping process dependent on having found a majority (>50%) of the eligible studies that would be expected to be found based on the BIR. It was not intended (and certainly not practically realisable[4]) for the review to be exhaustive in identifying all existing literature meeting our criteria. Due to the large quantity of literature identified within category A, studies in categories B and C were not subjected to routine full-text screening nor data extraction, although their content was discussed when this was deemed informative to the previously-outlined processes of definition development. It should also be noted that, given the scale of the review, we did not consider it practicable to attempt to appraise the quality or risk of bias of included studies.

[4] Whilst the methods we applied potentially significantly increase the efficiency of the overall process, their performance will inevitably show a decline over time. Whilst text-mining approaches get progressively ‘better’ at ‘learning’ to identify eligible studies, it concurrently gets more difficult to actually locate such studies (as fewer remain to be found).

Results

Search results

After accounting for the removal of duplicates, our electronic literature database searches identified 804,919 unique records. Of these we manually screened 54,651 records at the title and abstract stage and marked 358 records as eligible within category A. We can assess how this compares to the number of records we would have expected to find had we been able to manually screen titles and abstracts for the entire record set, by reference to the BIR. The BIR for studies in category A was calculated as 0.066%, suggesting that we could have expected to identify 533 such studies within the complete record set. We therefore estimate that the degree of completion of the title and abstract screening process was 358/533 or ~67%[5]. To get to this point, we screened ~7% of all the records retrieved (54,651/804,919), indicating that our method for detecting eligible records performed over 9 times better than we would have expected had we simply screened records at random (see BHRU review methods paper; in preparation).

With the addition of 495 articles identified via the snowball searching process, 853 articles were designated for full-text screening. 346 articles passed the full-text screening stage and had data extracted for inclusion in analysis. See Figure 1 for PRISMA flow diagram (Moher et al, 2009).

Development of an operational definition of choice architecture interventions in micro-environments

The first objective of the current review was to develop and refine a working definition and a conceptual framework for choice architecture interventions in micro-environments. Our original working definition was sufficiently inclusive that it allowed us to begin the scoping process, but it resulted in the provisional inclusion of studies whose characteristics did not meet our conception of choice architecture (and that the working definition had attempted to capture). As a result of the iterative process described in our methods, including a broad consensus being reached regarding key definitional elements during the expert workshop, we produced the final version in Box 1.

[5] These figures do not give a reliable assessment of the degree of completion of the overall scoping process, as they do not account for eligibility at the full-text stage, and we do not know for certain that all possible eligible studies were ever present in the set of 804,919 records on which the calculations are based. However, we did check that the records within a researcher-identified initial corpus of relevant studies were captured by our searches, and, such was the scale of the overall record set, it is unlikely that a substantial proportion of existing eligible studies were not included therein. Furthermore, the snowball searching process we used enabled us to incorporate literature not captured through the other processes, and which may have proven not to be within the as-yet-unscreened portion of the overall record set.

Figure 1. PRISMA flow diagram

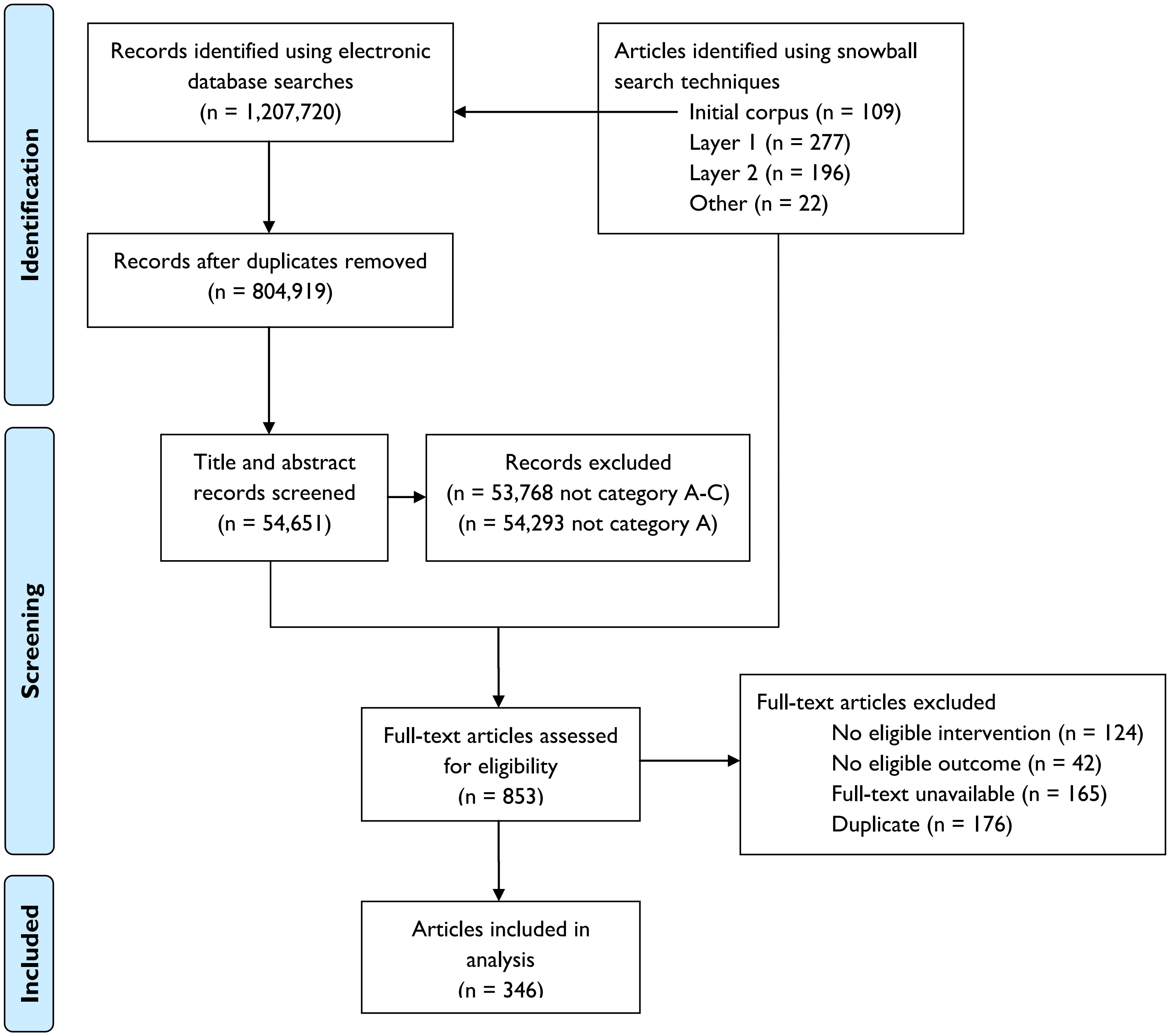

Box 1 – Operational definition of choice architecture interventions in micro-environments for changing health-related behaviour

'Interventions that involve altering the properties or placement of objects or stimuli within micro-environments with the intention of changing health-related behaviour.

Such interventions are implemented within the same micro-environment as that in which the target behaviour is performed, typically require minimal conscious engagement, can in principle influence the behaviour of many people simultaneously, and are not targeted or tailored to specific individuals.'

The term 'placement' includes interventions that involve placing one or more new objects or stimuli within a micro-environment, as well as those that involve altering the placement of objects or stimuli that were already present. The placement of these objects can be incidental to or intrinsically linked to undertaking a given behaviour. Use of the term 'typically require minimal conscious engagement' reflects our view that interventions of this kind most closely embody the outlined concept of choice architecture, while also recognising the potential for some degree of conscious engagement with the intervention (e.g. people may consciously engage with nutritional labelling or motivational signage by reading it, or with auditory stimuli by listening to them).

The core inclusion and exclusion criteria detailed previously remained largely consistent throughout the development of the definition.

Treatment of boundary issues and implications for inclusion/exclusion criteria

Whilst we think the working definition effectively captures the essence of choice architecture interventions in the context of public health, we recognised that applying such a definition to a wide range of existing interventions would result in an imperfect fit. Treatment of the boundary issues we encountered was therefore important in attempting to limit our operational definition and the scope of the review. Inevitably the principles could not always be applied without some degree of discretion by the review team. We judged the following boundary issues as the most salient:

1) Interventions not obviously scalable to population level

We excluded (by coding as category B) studies of interventions that did not appear practically feasible for replication or application in a similar form at a population level. Two notable groups of studies in this category were laboratory studies of individually administered interventions, and interventions reliant on manipulations of the behaviour or characteristics of other individuals within a social environment. The former involved, for example, the completion of computerised or information-processing tasks requiring significant instruction from the experimenter (e.g. Field et al, 2007; Wiers et al, 2010; Hollands et al, 2011). This contrasted with, for example, laboratory studies of the effect of portion sizing on food consumption, where there is no reason in principle why an essentially similar intervention could not be applied at a group or population level. The latter involved, for example, altering the physical appearance of waiting staff in a restaurant, or

altering the number of people sharing an environment with a target participant (e.g. McFerran et al, 2010).

2) Interventions that depend on provision of equipment or adaptation of environments

We excluded (by coding as category B) interventions consisting of the simple provision of equipment (such as installation of gym equipment) to provide previously unavailable behavioural options. However, we identified a boundary issue regarding interventions featuring the superficial physical adaptation of existing environments (such as painting a school playground surface to encourage active play) or the addition of equipment where the original purpose or function is not altered (such as equipping a school classroom with desks suitable for standing). In light of the difficulty in applying inclusion criteria consistently at this boundary, we included all such studies in category A.

3) Interventions delivered through 'new media'

The content of interventions delivered via the Internet or mobile phones is potentially accessible at all times, including in the micro-environments in which particular behavioural decisions are made. Some studies of interventions of this kind may therefore have been capable of meeting our operational definition of choice architecture interventions. However, we found it impossible to ascertain this level of detail at the title and abstract screening stage. These studies were therefore grouped in category C.

Development of a typology of choice architecture interventions

The second component of the first objective concerned the development of a conceptual framework or typology for choice architecture interventions, allowing us to map the range of such interventions and locate this within the broader array of public health interventions. We envisaged this process as drawing on both conceptual material and analysis of the empirical studies encountered, although ultimately the predominant focus was on the latter. During the scoping exercise, we captured only a small amount of conceptual or theoretical material considered highly relevant to the current review i.e. containing descriptions or explanations of terms or concepts pertaining to nudge or choice architecture and applied within a behavioural intervention context. Most of this was well known to us[6].

---

6 By primarily searching for conceptual work that explicitly referenced choice architecture, we necessarily excluded a large amount of material focusing on broader issues regarding environmental interventions or influences on behaviour and health. This wider evidence base may be useful in framing attempts to systematically map the broader terrain in which the subset of choice architecture interventions is nested. We intend to address this and related issues in future work.

We present an emergent typology of intervention types in Figure 2. This is a preliminary attempt to categorise the content of choice architecture interventions according to our operational definition. It primarily serves a descriptive function, needing further work to be able to integrate more complex theoretical or conceptual ideas. The typology comprises a list of nine categories of micro-environmental interventions, each encompassing a range of interventions with key common characteristics. A more detailed explanation of the content of each category is contained within Table 3. Within the typology, we suggest three broad classes that reflect the operational definition. The first class comprises intervention categories that primarily alter the properties of objects or stimuli, the second class comprises intervention categories that primarily alter the placement of objects or stimuli, and the third class comprises those interventions that alter both the properties and the placement of objects or stimuli[7]. We observe that these groupings map well onto one of the most detailed models describing how environmental stimuli elicit behavioural responses outside of awareness, through perceptual, evaluative, motivational and emotional processes (Bargh & Morsella 2009).

Mapping the available evidence

The second objective of the review was to identify and describe existing empirical research on the effects of interventions that met our operational definition. These data are presented in Tables 1, 2 and 3. Tables 1 and 2 show the numbers of study reports identified in the scoping review by behavioural domain and intervention category. Whilst we do not claim that the table shows *all* the available primary or secondary research, given the systematic large-scale approach taken to the review, the table should broadly reflect the true overall *distribution* of evidence, highlighting areas of relative paucity and abundance of evidence.

It is clear that most of the relevant primary research and systematic and non-systematic reviews had been undertaken in relation to diet-related behaviours, representing 70.2% of total study reports. No cell in the table is empty within this behavioural domain. Evidence relating to physical activity was the next most represented (19.1%), followed by that relating to alcohol (7.3%) and then to tobacco (representing just 3.4% of total study reports). The apparent relative scarcity of evidence on physical activity may reflect the fact that physical activity behaviours do not necessarily involve the use of a product, and therefore some of the intervention categories may be

---

[7] We discussed but did not implement alternative ways of organising the intervention categories, including in relation to the cognitive and emotional mechanisms likely to be engaged (such as automatic versus reflective processing systems), the spatial scale of the intervention relative to the target behaviour, and the level of declarative information given. Whilst it was agreed by consensus that the figure presented was an appropriate way to structure this information, it could also have been structured at a higher (i.e. linking broad commonalities or themes) or lower level (sub-classifications by e.g. behavioural domain, setting, detailed analysis of intervention components) with ever more granularity.

less applicable. But it is less clear why the types of intervention studied in relation to diet might not also, in principle, be applicable to alcohol and tobacco. Within physical activity, one cluster of studies – examining the use of motivational prompts for stair use – has received a great deal of research attention and been comprehensively reviewed, but we found no comparable examples of intense research activity relating to choice architecture and alcohol or tobacco.

Across intervention categories, the two most studied types of intervention were point-of-choice labelling (representing ~22% of total study reports, primarily in relation to diet), and prompting, such as standardised information or motivational prompts (representing ~19% of total reports, primarily in relation to physical activity). It is notable that these two most prevalent intervention types, labelling and prompting, were those that are most closely related to the tradition of information-giving interventions (and its corresponding theoretical foundations) and lie farthest from the centre of our outlined conception of choice architecture interventions. Outside of these examples, few intervention types were represented in substantive, consistent bodies of apparently similar studies. Only the prompting and priming categories were represented by at least one study report within all four behavioural domains.

Point-of-choice labelling was the category containing the greatest number of both primary and secondary research reports. However, it had not received much focused systematic review work, being covered either in (predominantly non-systematic) broader-scale reviews and overviews, or in narrow reviews examining specific implementation contexts. Prompting via standardised information or motivational prompts formed a somewhat unique area of the evidence mapped in this review: in relation to the principal cluster of interventions to promote stair use, it comprised a consistent body of primary research, undertaken within broadly equivalent settings and using similar methods, which had been covered in a series of systematic reviews that continue to be updated.

A further observation on the identified evidence is that there were relatively few high-quality systematic reviews that had been conducted within the scope of this review, with non-systematic reviews and broad overviews far more prevalent. Furthermore, relevant systematic reviews often encompassed a wide range of interventions, making it difficult to reliably assess the likely effect of specific intervention types.

Table 3 maps onto the typology outlined in Figure 2. Here we present a definition of each category of intervention types, detail the range of interventions within each category identified in the review, and briefly summarise the body of research found. This includes a qualitative overview

of the effects of the intervention on behavioural outcomes *as reported* in the primary studies or systematic reviews. It is important to note that it has not been possible either to critically appraise the studies mapped in this scoping review or to verify authors' conclusions regarding effectiveness. As such, for the most part we were unable to indicate the direction or magnitude of the effects with any confidence. The qualitative summaries varied between intervention categories. In some instances (in relation to sizing and prompting, the latter when applied to physical activity) there were a large number of studies of which the majority reported an effect of the intervention on behaviour, additionally supported by the conclusions of specific systematic review evidence. Within other categories (proximity, priming, availability, ambience) the majority of studies reported an effect on behaviour. A qualitative overview of the remaining intervention categories (presentation, labelling, functional design) revealed no consistent overall pattern of findings, with some studies reporting an effect of the intervention on behaviour and others reporting no effect. These observations should be interpreted with due caution. First, the reporting of an effect does not necessarily equate to an effect beneficial to health. For example, within most of the categories, at least some of the available research concerned the effectiveness of interventions in promoting behavioural outcomes that would be undesirable for health, such as the purchase or consumption of a less healthy product. While evidence from studies of this kind can suggest potential for mechanistically similar interventions to alter behaviour in the opposite direction, it is important to note that they do not provide direct evidence of effectiveness in promoting health-enhancing behaviours. Second, in most categories, studies were heterogeneous with respect to the populations, interventions, comparators or counterfactuals, outcomes and moderators assessed, and all studies remain to be examined in relation to potential confounders, the reporting of multiple outcome measures, subgroup effects and many other aspects of design, analysis and reporting.

Figure 2. Emergent typology of intervention types

Primarily alter **properties** of objects or stimuli	**AMBIENCE**<br>**FUNCTIONAL DESIGN**<br>**LABELLING**<br>**PRESENTATION**<br>**SIZING**
Primarily alter **placement** of objects or stimuli	**AVAILABILITY**<br>**PROXIMITY**
Alter both **properties and placement** of objects or stimuli	**PRIMING**<br>**PROMPTING**

Table 1 – Numbers of study reports identified by intervention type and target behaviour
(NB Numbers include reports featuring multiple intervention types and across multiple behaviours)

Intervention class	Intervention type	Number of studies			
		**Diet** 309/440=70.2%	**Physical activity** 84/440=19.1%	**Alcohol** 32/440=7.3%	**Smoking** 15/440=3.4%
Primarily alter **properties** of objects or stimuli	**AMBIENCE** - alter aesthetic or ambient aspects of the surrounding environment	33	10	14	
	**FUNCTIONAL DESIGN** - design or adapt equipment or function of the environment	27	11	5	
	**LABELLING –** apply labelling or endorsement information to product or at point-of-choice	78		7	10
	**PRESENTATION** - alter sensory qualities or visual design of the product	21			2
	**SIZING** - change size or quantity of the product	66			1
Primarily alter **placement** of objects or stimuli	**AVAILABILITY** - add behavioural options within a given micro-environment	28	6		
	**PROXIMITY -** make behavioural options easier (or harder) to engage with, requiring reduced (or increased) effort	21	1		
Alter both **properties and placement** of objects or stimuli	**PRIMING** - place incidental cues in the environment to influence a non-conscious behavioural response	9	1	5	1
	**PROMPTING -** use non-personalised information to promote or raise awareness of a behaviour	26	55	1	1

Table 2 – Distribution of primary and secondary research reports
(NB Numbers include reports featuring multiple intervention types and across multiple behaviours)

Behaviour / Intervention type	Diet (n=309/440=70.2%)		Physical activity (n=84/440=19.1%)		Alcohol (n=32/440; 7.3%)		Tobacco (n=15/440; 3.4%)	
	Primary	Secondary	Primary	Secondary	Primary	Secondary	Primary	Secondary
Primarily alter **properties** of objects or stimuli								
**Ambience** (n=57/440; 13.0%)	22	11	10	-	10	4	-	-
**Functional design** (n=43/440; 9.8%)	17	10	10	1	2	3	-	-
**Labelling** (n=95/440; 21.6%)	69	9	-	-	2	5	7	3
**Presentation** (n=23/440; 5.2%)	15	6	-	-	-	-	1	1
**Sizing** (n=67/440; 15.2%)	48	18	-	-	-	-	1	-
Primarily alter **placement** of objects or stimuli								
**Availability** (n=34/440; 7.7%)	17	11	5	1	-	-	-	-
**Proximity** (n=22/440; 5.0%)	14	7	-	1	-	-	-	-
Alter both **properties and placement** of objects or stimuli								
**Priming** (n=16/440; 3.6%)	7	2	1	-	3	2	1	-
**Prompting** (n=83/440; 18.9%)	17	9	42	13	1	-	1	-

Table 3 - Intervention types identified and brief summaries of respective evidence

Intervention type	Range of interventions identified	Summary of evidence
Primarily alter **properties** of objects or stimuli		
**AMBIENCE**<br>Interventions or manipulated factors that alter ambient, atmospheric or aesthetic aspects of the micro-environment surrounding a behaviour, but which are independent of or incidental to it. Differentiated from priming interventions as the latter category contains interventions which, whilst also independent of or incidental to a behaviour, are explicitly designed to activate non-conscious behavioural processes via their content	• Decoration, including colour, artwork, carpeting, use of different materials (e.g. painting stairwells or school playgrounds to enhance appeal);<br>• Brightness of lighting;<br>• Music volume, tempo (e.g. altering music played in food or alcohol purchasing/consumption environments);<br>• Distraction via television or radio	Represented ~13% of total studies. A wide variety of interventions was identified. The majority of primary research studies were field studies with a range of study designs. There was substantial variety in primary outcomes. A number of non-systematic reviews were identified that took a broader or narrower perspective, or that mapped relatively closely on to the parameters of this category. Only one systematic review was identified, which was comparatively less relevant and included primarily observational evidence. The majority of studies reported an effect of the intervention on behaviour.
**FUNCTIONAL DESIGN**<br>The design or adaptation of the physical micro-environment, through changes to equipment or objects. Excludes labelling and presentation	• Demarcation of supermarket trolley space for fruit and vegetables;<br>• Supermarket trolleys necessitating increased effort to push;<br>• Supermarket trolleys with handle displays to indicate qualities of products;<br>• Trays versus lack of trays within cafeterias;<br>• Trolley versus basket use within supermarket environments;	Represented ~10% of total studies. A wide variety of interventions was identified, with one relatively substantive cluster of studies that altered size, shape or design of food and drink receptacles. Primary research studies were both laboratory- and field-based using a wide variety of study designs including controlled trials and time series designs. There was substantial variety in primary outcomes. One systematic review was identified, focusing on the effect of environmental factors within alcohol drinking venues, but comprised primarily observational evidence. There was no consistent overall pattern of findings, with

	• School classroom and desk design to encourage standing;<br>• Work desks adapted to include exercise opportunities;<br>• Painting of school playgrounds to indicate and create activity opportunities;<br>• Marking physical activity routes within existing micro-environments;<br>• Changes in amounts of seating available in consumption environments;<br>• Tableware design (e.g. plates with surface markings to suggest food consumption, such as portion control plates);<br>• Shape and size of plates and drinking glasses;<br>• Type or size of eating utensils	some studies reporting an effect of the intervention on behaviour and others reporting no effect
**LABELLING**<br>Interventions that present labelling or endorsement information specific to a product, either directly applied to the product itself or at point-of-choice (e.g. shelf-edge labelling, menu labelling)	• Indication of typical reference portion;<br>• Nutritional labelling indicating quantity of nutrients contained (e.g. calories, fat or multi-nutrient, traffic light, Guideline Daily Amounts);<br>• Health warnings;<br>• Nutrient claims (e.g. 'low fat', 'reduced salt';<br>• Product endorsements (e.g. sporting or celebrity endorsements)	This was the largest single category, and represented ~22% of total studies. The majority of primary research studies were field studies using a wide variety of study designs including controlled trials and time series designs. There was substantial variety in primary outcomes including consumption, purchasing and selection, and multiple outcomes were often assessed within studies. A number of systematic and non-systematic reviews were identified. In relation to diet, these typically had a scope markedly wider than this category. There were also highly relevant systematic reviews within alcohol and tobacco domains. There was no consistent overall pattern of findings, with some studies reporting an effect of the intervention on behaviour and others reporting no effect, and many studies reporting multiple outcomes. In relation to alcohol, systematic review evidence reported that the intervention had little or no effect on purchasing or consumption. In relation to tobacco, systematic review evidence reported the difficulty in ascertaining the effect of health warnings on smoking behaviour

**PRESENTATION**<br>Interventions that alter the sensory qualities or visual design of the product itself, including that actually consumed and its packaging, but not factors external to that. Excludes labelling	• Elements of packaging design including plain versus branded packaging, colour of packaging;<br>• Presenting different amounts of a product on packaging illustration (to alter consumption anchors);<br>• Characteristics of the consumed substance itself including manipulating variety in appearance through altering colour of food / way food is arranged, e.g. shaping or presenting food to enhance visual appeal	Represented ~5% of total studies. All but two studies were in relation to diet, with a wide variety of interventions identified. The majority of primary research studies were laboratory-based using both within- and between-subject designs. Consumption was the predominant primary outcome. Two relevant systematic reviews were identified, each focusing on small areas within this category. One reviewed the effect of sensory properties of foods including appearance and variety; the other reviewed the effect of plain packaging of tobacco products. There was no consistent overall pattern of findings, with some studies reporting an effect of the intervention on behaviour and others reporting no effect
**SIZING**<br>Interventions that change the size or quantity of the product itself. This can relate to size of the overall package, size of a portion served or contained within the overall package, or size of an individual unit within a portion	• Changes to package size, portion size or unit size of a product (these may be interchangeable or synonymous depending on the targeted product)	Represented ~15% of total studies. All but one study were in relation to diet, with most studies examining the effects of changes to portion size. The majority of primary research studies were laboratory-based and both within- and between-subject designs were used. Consumption was the predominant primary outcome. Wide coverage in non-systematic reviews. One systematic review was identified that mapped closely on to the parameters of this category (Steenhuis & Vermeer, 2009). This review identified 13 studies on the effects of portion size on food intake, although there were methodological limitations in terms of search strategy and lack of adequate quality assessment. Our search identified all 13 of these studies. The specific systematic review supports the same conclusion as the broader range of primary research studies identified, reporting that there was an effect of portion size on consumption

Primarily alter **placement** of objects or stimuli		
**AVAILABILITY**<br>Interventions that alter availability through adding behavioural options, or changing capacity for engagement with behavioural options, providing broadly equivalent options/behaviours from the previous potential behaviour set remain available within the micro-environment	• Increasing available healthier food options via e.g. increasing variety of healthy options or providing more or less of specific nutrients/foods in available options, such as introducing more low-fat items in vending machines;<br>• Altering quantity of specific available products within a given environment (i.e. stockpiling);<br>• Altering availability of stairs or of alternatives to stair use i.e. escalators and lifts, altering lift use speed, increasing stair width, implementing 'skip-stop' lifts that do not serve every floor of a building	Represented ~8% of total studies. The majority of primary studies were within the diet domain with none in alcohol or tobacco. Two marked clusters of studies were identified specific to behavioural domains: increasing availability of healthier food and drink options in restaurants and vending machines; and a small group of studies of interventions to increase or decrease the availability of stairs, lifts or escalators to impact on their respective use. The majority of primary research studies were field studies with a range of study designs including controlled trials and time series designs being used. There was substantial variety in primary outcomes, and multiple outcomes were often assessed within studies. A number of systematic and non-systematic reviews were identified although these typically had a scope markedly wider than this category thus not providing specific in-depth coverage. The majority of studies reported an effect of the intervention on behaviour, although this was often complicated by the concurrent implementation of multiple interventions or the assessment of multiple outcomes
**PROXIMITY**<br>Interventions that facilitate engagement with available behavioural options by making such options more immediately salient or reducing required effort, primarily through altering proximity, but also accessibility or visibility	• Altering layouts within micro-environments to increase/decrease distance of products from routes of passage or seating e.g. placing area for dispensing certain foods at greater distance;<br>• Placing products out of immediate sight or making them less visible e.g. displayed within or behind opaque versus transparent materials;<br>• Changing item positions within a food menu;<br>• Making purchasing process more or less convenient for certain products;	Represented ~5% of total studies. All primary studies identified were in relation to diet. The majority of primary research studies were field studies or naturalistic laboratory studies, with a range of study designs including between- and within-subjects designs and time series designs. There was substantial variety in primary outcomes, reflecting changes in consumption, purchasing or selection of products. No relevant systematic reviews were identified although this category was covered within a number of broadly focused non-systematic reviews. The majority of studies reported an effect of the intervention on behaviour

	• Highlighting potential swaps for healthier products within ashopping environment	
Alter both **properties and placement** of objects or stimuli		
**PRIMING**<br>Interventions that involve the placement of incidental cues, objects or stimuli within the micro-environment, or within material that a person is exposed to, to induce or influence non-conscious behavioural response via the activation of e.g. semantic relationships or associative processes	• Priming of dieting goals via recipe on shop door;<br>• Placing decorative objects within restaurants to prime consumption of specific food types;<br>• Placing smoking-related objects in a room incidental to an assigned task;<br>• Content of music to evoke associations (origin of music, lyrical content);<br>• Décor and table furniture of restaurants to suggest ethnic theme or elicit associations with snack versus meal consumption	This was the smallest category, and represented ~4% of total studies, with examples identified within every behavioural domain. In addition, a wide variety of interventions was identified, with no obvious clusters of highly-related studies. The majority of primary research studies were field or naturalistic laboratory studies with a range of study designs being used. There was substantial variety in primary outcomes. No relevant systematic reviews were identified, although this category was covered within non-systematic reviews. The majority of studies reported an effect of the intervention on behaviour
**PROMPTING**<br>Interventions that contain standardised explicit verbal, visual and/or numeric information intending to promote or raise awareness of, and thus motivation for, a given behaviour. Differentiated from labelling interventions by not being specific to the content of individual products and providing more general motivational prompting	• Promotional signage and materials including posters, screens, audio, public announcements;<br>• Motivational prompts (signs, posters, footprint symbols) for stair versus lift or escalator use including on surfaces proximal to stairwells and on stair risers	This was the second largest category, and represented ~19% of total studies, with examples identified within every behavioural domain. The majority of the studies were within the physical activity domain, with a dominant cluster of studies of motivational point-of-decision prompts for stair versus lift or escalator use. Primary research studies were all field-based, using mainly time series designs. Stair use was the predominant primary outcome. A number of systematic reviews were identified that map closely on to the parameters of this category. The most recent systematic review evidence concerning stair use interventions included 12 eligible studies and conducted systematic searches, assessment of study quality and quantitative synthesis (Soler et al, 2010). Our search identified all 12 of these studies. In relation to physical activity, the majority of studies reported an effect of the intervention on behaviour, supported by the specific systematic review

		evidence. In relation to the minority of studies concerning diet, many studies reported multiple outcomes and there was no consistent overall pattern of findings, with some studies reporting an effect of the intervention on behaviour and others reporting no effect

Discussion

Principal findings

This review has developed a definition and a provisional typology of choice architecture interventions, and has identified and described a large body of relevant primary and secondary research. This body of literature was characterised by a predominance of research on diet-related behaviours (relative to physical activity-, alcohol- and tobacco-related behaviours), and of the interventions of labelling and prompting, which are most closely related to the tradition of information-giving. The literature was also characterised by a lack of good quality systematic review evidence. It has revealed significant opportunities for further primary and secondary research as well as conceptual work to inform international efforts to change behaviour to improve population health and reduce health inequalities. For the most part, we do not yet have a clear indication of the direction and magnitude of the effects of interventions on short-term behavioural outcomes (and certainly not on enduring behaviour change), and how such effects might be distributed between social groups.

Strengths, limitations and challenges

The primary strengths of this review derive from its broad-ranging scope in attempting to systematically map the entire range of choice architecture interventions represented in the empirical research literature. Concurrently we have been able to apply a level of focus sufficient to delineate intervention types and components, and also to identify and describe the majority of the relevant evidence, a conclusion supported by the fact that our searches detected every study included in substantive systematic reviews within our intervention categories. Irrespective of our use of the contentious and ill-defined choice architecture terminology to frame this review, we have applied operational definitions that mean the review has essentially encompassed interventions that change micro-environments. As such, its content has value beyond current popular thinking and its specific terminology, and resonates with more traditional concepts of the lifeworld and the social structure and the ways that these impact on social actors (Kelly, 2006). Whilst the conceptual and definitional work is presented as provisional rather than definitive, it provides a solid starting point for future work, with no such substantive foundation having existed previously.

An inherent component of such a comprehensive approach has been the deliberate crossing of disciplinary boundaries throughout the review process, giving recognition to the unique explanatory power each perspective offers in a way that is often proposed but rarely done. The review team incorporates academic backgrounds in public health, psychology, economics, nutrition, sociology and specific evidence synthesis and other methodological expertise, as well as knowledge and experience of public policy processes. This is important, as the design, evaluation and implementation of behaviour change programmes and the translation of evidence into policy has arguably been hampered by being located within single disciplines.

A final key strength of this review is that its completion validates our chosen methods, having made manageable what initially seemed an impossible task. Applied more generally, this can inform the development of scoping reviews (i.e. those not predicated on exhaustively identifying every eligible study) in which it is not possible to apply common structural approaches (i.e. the PICOS approach[8]) in developing search strategies, or in which part of the goal is to iteratively define and focus on the target itself. This review illustrates that such work can be conducted in a systematic manner, increasing confidence that a broad range of evidence has been captured.

The scale of the review and its specific aims necessarily also result in some limitations and challenges. First, whilst we are able to represent the likely relative distribution, and the overall range, of existing evidence, the review is not (and was never intended to be) exhaustive. The range and heterogeneity of the interventions covered, and prior lack of definitional clarity (with corresponding implications for the search process), precluded in-depth systematic review as the starting point. The implication is that inevitably we will not have identified (or at least not extracted into the review) all informative individual studies and thus sections of the landscape may be misrepresented in scale or content, or not represented at all. This concern is mitigated by reference to the explicitly iterative nature of this work, allowing it to be built upon by future research. Second, we were unable to undertake detailed assessment of study quality, characteristics or results. Had we been able to do this, we would inevitably have identified potentially important considerations and perhaps summarised the evidence differently[9]. This means

---

[8] The PICOS approach helps to structure the research question(s) by using five components where each letter refers to a component: the patient population or the disease being addressed (P), the interventions or exposure (I), the comparator group (C), the outcome or endpoint (O), and the study design chosen (S) (O'Connor et al, 2008).

[9] As illustration, looking across the cluster of studies of point-of-decision motivational prompts for stair use, the evidence appears consistently promising, and it seems reasonable to extract such a gist. Upon closer inspection, the picture is more complex, with, for example, an apparent lack of evidence of the long-term duration of effects (Soler et al, 2010), and evidence of the interactive effects of participant characteristics and message content used (Lewis & Eves, 2011, 2012), and of the intervention context (behavioural options are stairs versus escalators, or stairs versus lifts) (Eves, 2010).

that the brief evidence summaries provided are highly provisional and should be interpreted with due caution. Finally, given that the priority here is to consider the *potential* of interventions to change behaviour, attention was not paid to issues of implementation, such as cost, the potential need for regulatory or legislative frameworks to enable the use of other types of interventions, or public acceptability (Ipsos MORI, 2010).

Further comments on the conceptual component

Our proposed definition reflects our stated focus on physical and social dimensions of micro-environments. In practice we did not encounter empirical studies of interventions that involve altering social dimensions of micro-environments (such as those centred on changing social norms (Moreira et al, 2009)) that met our definition. We excluded interventions that involve the use of economic instruments (e.g. taxes, subsidies, income transfers), in line with prior formulations of choice architecture. However, these exclusions do not represent an undermining of their potential value. The latter is the result of a deliberate *a priori* decision, in the knowledge of a parallel workstream on the effects of economic interventions on diet and physical activity.

Only one included study used the term 'choice architecture' in its title (Thorndike et al, 2012) and only three used the term 'nudge' (Rozin et al, 2011; Dayan & Bar-Hillel, 2011; Kalnikaite et al, 2011). This affirms our search strategy because it suggests that traditional search methods may have failed to detect relevant studies. The lack of overlap between the terms used in current policy spheres and the academic literature may reflect the time needed to conduct and publish studies. It may also reflect the lack of conceptual clarity required for fruitful translation between the two communities and links between different literatures, which would support the need to develop a stronger conceptual framework and associated typology or taxonomy. Given the lack of prior consideration of the terms, producing workable definitions was a challenging element of this review. The difficulty of reaching agreement is neatly highlighted by objections raised within the scoping process to the term at the very centre of this review – choice architecture – with it having been suggested that neither 'choice' nor 'architecture' express the desired meaning. We reached a consensus to continue to use the term for pragmatic reasons, but note that a desirable potential outcome of the further mapping of public health interventions described in this review would be the refinement of this and associated terms.

Further comments on the empirical component

Key to improving population health and reducing health inequalities, is understanding the short-term and sustained effects of interventions, both singly and in combination, and their effects not

just in aggregate but within and across social groups. Whilst these issues could not be examined in detail within the scope of the review, it is clear that the next generation of primary and secondary research (including those suggestions proposed in this review) would benefit from attempting to address them, preferably concurrently where possible. For example, regarding sustained behaviour change, it appears that there was typically little reporting of the durability of effects. As many of the interventions rely on brief exposures limited to time and place, such as point-of-choice prompts, or altering layouts within buildings, a one-off exposure to an intervention would not necessarily be expected to have an enduring behavioural impact. Accordingly, we are instead most interested in the consistency of an effect evoked on multiple occasions over time within the same population. This requires long-term alteration of aspects of the micro-environment within an evaluative framework.

On a more practical note, in attempting to summarise the evidence within each category, producing a qualitative overview of the reported evidence for each was difficult. It required, for example, consideration of evidence from complex interventions with multiple components (some, such as pricing and educational programmes, potentially falling outside the boundaries of choice architecture) where it may not be possible to isolate the effects of specific active elements. It was also not feasible to consider subgroup effects within such overviews. In addition, particularly in relation to studies of diet-related behaviours, multiple outcomes were often reported with no clear identification of which were the primary outcomes. Finally, within this review, we have typically considered the evidence within intervention categories across all four behavioural domains, and looking at each domain separately might have resulted in different readings.

Differences in research focus between behavioural domains

Most of the extant research relates to the effects of interventions on diet-related behaviours. In considering the reasons for this, the pertinent comparison is between diet and the two other consumptive behaviours, alcohol- and tobacco-related behaviours, where we might expect similar interventions to be feasible in principle. Alcohol and tobacco products have historically been more subject to interventions based on regulation, restriction and pricing than to the types of interventions within the scope of this review. From an economic perspective, there is a more obvious case for these types of approaches in alcohol and tobacco than there is in the case of diet: it is harder in the latter case to argue for the presence of external effects (i.e. the 'harm to others'), the classical market failure that would justify public policy intervention in the traditional welfare economic sense (Suhrcke et al, 2006). In addition, diet-related behaviours arguably offer greater scope for research in general; therefore it is not surprising that we should have found

more evidence for choice architecture interventions. Unlike the consumption of alcohol and tobacco, eating and drinking are essential to survival, and diet-related behaviours offer a greater variety of opportunities for intervention over a range of products, situations and environments. Furthermore, larger proportions of the population (including children) are identified as being at increased risk of chronic disease through being overweight or obese, compared with those who use tobacco or consume alcohol in harmful ways. Such factors may also influence the applicability or feasibility of specific choice architecture interventions. For example, those intervening to improve dietary intake by altering availability can choose to decrease the availability of a wide variety of less healthy products, or increase the availability of a wide variety of more healthy products. There are fewer comparable intervention options for alcohol and tobacco, although there are examples of healthier alternatives, such as low alcohol or alcohol-free drinks, and, more contentiously, so-called 'safer' (e.g. electronic) cigarettes.

Scarcity of evidence for the physical activity domain probably reflects the fact that the intervention categories cannot be consistently applied, as well as the greater focus in existing research on the external built environment and the relative difficulty of undertaking small-scale laboratory research comparable to that in other domains[10].

Implications for research

1) Identifying candidates for in-depth systematic review

Unlike in a review of a clearly conceptualised area, in which apparent gaps in the evidence and their significance would be readily identified, the greatest contribution of this review has been to attempt to map and define a previously undefined set of interventions. It follows that some of the apparent gaps mapped in the available evidence may not be particularly meaningful, instead reflecting the fact that some types of intervention may simply not be applicable to some target behaviours. Furthermore, all of these areas require closer examination and specific searches to determine more definitively whether we have represented them fairly and whether they do indeed merit further review. With these caveats in mind, we identified the following potential areas for in-depth systematic reviews:

i) Interventions that primarily alter the properties of objects or stimuli within micro-environments. All of the intervention categories we grouped in this way, namely sizing, presentation, labelling, functional design, and ambience, had either not been the subject of systematic reviews, or those systematic reviews typically had limitations relating to search

[10] The mechanisms may also differ between behavioural domains. Regarding physical activity, the public health problem is a result of people not engaging in these behaviours or not engaging enough, whereas regarding consumptive behaviours, this is principally a problem of overconsumption.

strategies, replicable methodology or synthesis of findings; were not up-to-date, or did not map closely on to the parameters of the intervention categories. This suggests that well-conducted systematic review work within or across any of these areas would have potential to significantly enhance the evidence base for choice architecture interventions. This primarily applies to diet-related behaviours, reflecting the lack of primary research in other behavioural domains.

ii) Interventions that primarily alter the placement of objects or stimuli within micro-environments. Although the intervention category of proximity comprised a relatively small number of primary research studies, we identified no systematic review of the area, suggesting value in a review focusing on the effects of the layout of internal built environments.

iii) Interventions that target automatic or non-conscious psychological processes. The intervention category of priming was the only one that inherently suggests its mechanism of effect i.e. evoking a behavioural response via activation of automatic semantic relationships or associative processes. Although there were a relatively small number of studies, we identified no systematic review of the area.

However, there were examples of primary research studies across all behavioural domains. These observations suggest need for a more theoretically-driven systematic review that would need to address how such concepts translate into public health intervention contexts.

2) Identifying candidates for primary research

In such a large scoping review it has not been possible to identify methodological issues or highly specific unanswered research questions arising from the body of existing primary research within those areas that are heavily populated. As such, it was not possible to identify with rigour or confidence, specific avenues of enquiry that are likely to be fruitful. However, we were able to identify broad areas where there was a relative paucity of existing primary research and where we think there exists a range of potentially tractable research questions.

i) Interventions to change physical activity-related behaviours within micro-environments. The majority of primary research studies within the physical activity domain concerned stair use interventions (categorised within the prompting category). Whilst a number of intervention categories that we identified do not readily translate to physical activity, several do, as evidenced by the fact that at least some primary research was identified in a further four categories. This suggests that opportunities for primary research in all such under-represented areas merit further attention.

ii) Interventions to change alcohol- and tobacco-related behaviours within micro-environments. Research on these behaviours comprised only ~11% of the total studies identified and no intervention categories were heavily populated relative to other behavioural domains. This is notable because, as with diet, these are consumptive behaviours, and we would have expected to identify a more comparable level of primary research. For example, in relation to alcohol, no primary research studies on sizing, presentation or proximity were identified, but work in these areas could address the potential impact of factors such as the size and packaging of alcohol products, and layouts of retail outlets.

3) Conceptual development

The conceptual and definitional work within this review encompasses the operational definition and an emergent typology of choice architecture interventions in micro-environments. These primarily serve a descriptive function and whilst they have been extensively and carefully developed throughout the scoping process, are presented as provisional rather than definitive in nature. They will likely evolve and be refined and elaborated upon through exposure to new evidence and immersion in salient theoretically grounded work on behaviour. In addition, we propose that there would be significant value in work attempting to integrate these broad descriptive sketches of the area with more complex theoretical and conceptual ideas, in order to examine the social, cognitive, emotional and behavioural processes and mechanisms that may underlie observed effects of interventions. Significant conceptual and practical issues encountered within the scoping process also suggest a number of other opportunities to more widely inform the field. Examples include detailed conceptual analysis of other existing frameworks of population health interventions and important work on how to define key behaviours and behavioural outcomes. We also note that the relative scarcity of intervention evidence in many areas means that there may be value in reviews of correlational research (which was excluded from this review) to provide guidance as to likely effect sizes and targets for intervention.

Whilst the present review focuses on a specific subset of interventions within the micro-environment, the general iterative approach we have taken and the specific methods developed may also be extended to detailed mapping of other areas of the far larger landscape of population-level interventions. Combined with efforts to improve definitional and conceptual clarity concerning interventions, outcomes and their measurement, this could aid in the process of generating a systematic and rigorous evidence base for the effects of all interventions to change

population health behaviour, helping to structure existing evidence, inform future research and provide a framework for understanding potential mechanisms of effect.

Conclusion

This scoping review has made the following three key contributions to the evidence base for choice architecture interventions:

i) Development of a definition and a provisional typology of choice architecture interventions in micro-environments
ii) Description of a large body of relevant primary and secondary research
iii) Identification of significant opportunities for further primary research and evidence synthesis as well as conceptual work to contribute to international efforts to change behaviour to improve population health and reduce health inequalities.

Acknowledgements

The scoping review was conducted in collaboration with colleagues at the Evidence for Policy and Practice Information and Co-ordinating Centre (EPPI-Centre), Social Science Research Unit, Institute of Education, University of London. In particular, we acknowledge the contributions of the following EPPI-Centre staff: James Thomas, Jeff Brunton, Sergio Graziosi, Alison O'Mara-Eves and Claire Stansfield. We also acknowledge the support provided by colleagues in MRC Human Nutrition Research, the UKCRC Centre for Diet and Activity Research (CEDAR) and the wider BHRU team, including members of the BHRU Scientific Advisory Board, plus the contributions of attendees at an expert workshop held in Cambridge in 2011.

References

References from report (Cited in Introduction, Methods or Discussion)

Abraham, C., & Michie, S. (2008). A taxonomy of behavior change techniques used in interventions. *Health Psychology*, *27*, 379-87.

Ajzen, I. (1991). The theory of planned behaviour. *Organisational Behavior and Human Decision Processes*, *50*, 179-211.

Albarracin, D., Gillette, J. C., Earl, A. N., Glasman, L. R., Durantini, M. R., & Ho, M.-H. (2005). A test of major assumptions about behavior change: A comprehensive look at the effects of passive and active HIV-prevention interventions since the beginning of the epidemic. *Psychological Bulletin*, *131*, 856 – 897.

Bargh, J.A., Morsella, E. (2009). Unconscious behavioral guidance systems. In: Agnew, C., Graziano, W., Kelly, J., editors. *Then a miracle occurs: Focusing on behavior in social psychological theory and research*. New York: Oxford University Press.

Bonnefoy, J., Morgan, A., Kelly, M.P., Butt, J., Bergman, V., with P. Tugwell, V. Robinson, M. Exworthy, J. Mackenbach, J. Popay, C. Pope, T. Narayan, L. Myer, S. Simpson, T. Houweling, L. Jadue (2007). Constructing the evidence base on the social determinants of health: A guide. Report to the World Health Organization Commission on the Social Determinants of Health, from Measurement and Evidence Knowledge Network. Accessed at http://www.who.int/social_determinants/knowledge_networks/add_documents/mekn_final_guide_112007.pdf

Cabinet Office Behavioural Insights Team (2010). Applying behavioural insight to health. Cabinet Office.

Cabinet Office Behavioural Insights Team (2011). Behavioural Insights Team Annual update 2010–11. Cabinet Office.

Dahlgren, G., & Whitehead, M. (1991) Policies and strategies to promote social equity in health. Stockholm: Stockholm Institute of Future Studies.

Davison, K.K., & Lawson C.T. (2006). Do attributes of the physical environment influence children's physical activity? A review of the literature. International Journal of Behavioral Nutrition and Physical Activity, *3*:19.

Dayan, E., & Bar-Hillel, M. (2011). Nudge to nobesity II: Menu positions influence food orders. *Judgment and Decision Making*, *6*(4), 333-342.

De Vet, E., de Ridder, D.T.D., & de Wit J.B.F. (2011) Environmental correlates of physical activity and dietary behaviours among young people: a systematic review of reviews. *Obesity Reviews*, *12*(5), 130-42.

Dolan, P., Hallsworth, M., Halpern, D., King, D., & Vlaev, I. (2010). MINDSPACE: influencing behaviour through public policy. Cabinet Office.

Eves, F.F. (2010) Effects of point-of-decision prompts for stair use depend on the alternative. *American Journal of Preventive Medicine*, *38*, 573–574.

Faith, M.S., Fontaine, K.R., Baskin, M.L., & Allison, D.B. (2007). Toward the Reduction of Population Obesity: Macrolevel Environmental Approaches to the Problems of Food, Eating, and Obesity. *Psychological Bulletin*, *133*(2), 205-226.

Field, M., Duka, T., Eastwood, B., Child, R., Santarcangelo, M., & Gayton, M. (2007) Experimental manipulation of attentional biases in heavy drinkers: do the effects generalise? *Psychopharmacology*, *192*, 593-608.

French, S.A., Story, M., & Jeffery, R.W. (2001). Environmental influences on eating and physical activity. *Annual Review of Public Health*, *22*, 309–335.

Gawronski, B., & Bodenhausen, G.V. (2006). Associative and propositional processes in evaluation: An integrative review of implicit and explicit attitude change. *Psychological Bulletin*, *132*, 692-731.

Greenhalgh, T., & Peacock, R. (2005). Effectiveness and efficiency of search methods in systematic reviews of complex evidence: audit of primary sources. *British Medical Journal*, *331*, 1064-1065.

Greenhalgh, T., Robert, G., Macfarlane, F., Bate, P., Kyriakidou, O., & Peacock, R. (2005). Storylines of research in diffusion of innovation: a meta-narrative approach to systematic review. *Social Science & Medicine*, *61*, 417-430.

Hofmann, W., Friese, M., & Wiers, R. W. (2008). Impulsive versus reflective influences on health behavior: A theoretical framework and empirical review. *Health Psychology Review*, 2, 111-137.

Hollands, G.J., Prestwich, A., Marteau, T.M. (2011). Using aversive images to enhance healthy food choices and implicit attitudes: an experimental test of evaluative conditioning. *Health Psychology*, 30(2):195-203.

House of Lords Science and Technology Select Committee (2011). Report on behaviour change. House of Lords.

Ipsos MORI (2010). National Health? Citizens' views of health services around the world. Ipsos MORI.

Kalnikaite, V., Rogers, Y., Bird, J., Villar, N., Bachour, K., Payne, S., Todd, P.M., Schöning, J., Krüger, A. (2011). How to Nudge In Situ: Designing Lambent Devices to Deliver Information Salience in Supermarkets. *Proceedings of the 13th ACM International conference on Ubiquitous computing (Ubicomp '11).*

Kelly, M.P. (2006). Mapping the life world: a future research priority for public health. In Killoran, A., Swann, C., Kelly, M.P., (Eds). Public Health Evidence: Tackling Health Inequalities. Oxford: Oxford University Press.

Kirk, S.F.L., Penney, T.L., & McHugh, T.L.F. (2010). Characterizing the obesogenic environment: the state of the evidence with directions for future research. *Obesity Reviews*, *11*, 109-117.

Lewis, A.L., & Eves, F.F. (2011). Specific Effects of a Calorie-Based Intervention on Stair Climbing in Overweight Commuters. *Annals of Behavioral Medicine*, *42*(2), 257-261.

Lewis, A.L., & Eves, F.F. (2012). Prompts to increase stair climbing in stations; the effect of message specificity. *Journal of Physical Activity and Health*, online pre-print.

Lowe, D., Ryan, R., Santesso, N., & Hill, S. (2011). Development of a taxonomy of interventions to organise the evidence on consumers' medicines use. *Patient Education and Counseling*, *85*(2), 101-7.

Marteau, T.M., Hollands, G.J., Fletcher, P.C. (2012). Changing human behaviour to prevent disease: the importance of targeting automatic processes. *Science*, 337(6101), 1492-1495..

Marteau, T.M., Ogilvie, D., Roland, M., Suhrcke, M., & Kelly, M.P. (2011). Judging nudging: can nudging improve population health? *BMJ*, *342*, 263-265.

Matson-Koffman, D.M., Brownstein, J.N., Neiner, J.A., & Greaney, M.L. (2005). A Site-specific Literature Review of Policy and Environmental Interventions that Promote Physical Activity and Nutrition for Cardiovascular Health: What Works? *American Journal of Health Promotion*, *9*, 167–193.

McFerran, B., Dahl, D.W., Fitzsimons, G.J., & Morales, A.C. (2010). Might an Overweight Waitress Make You Eat More? How the Body Type of Others Is Sufficient to Alter Our Food Consumption. *Journal of Consumer Psychology*, *20*(2), 146–5.

Michie, S., Abraham, C., Eccles, M.P., Francis, J.J., Hardeman, W., & Johnston, M. (2011). Strengthening evaluation and implementation by specifying components of behaviour change interventions: a study protocol. *Implementation Science*, *6*:10.

Michie, S., van Stralen, M.M., & West, R. (2011). The behaviour change wheel: A new method for characterizing and designing behaviour change interventions. *Implementation Science*, *23*: 6–42.

Moher, D., Liberati, A., Tetzlaff, J., Altman, D.G., & The, P.G. (2009). Preferred Reporting Items for Systematic Reviews and Meta-Analyses: The PRISMA Statement. *PLoS Medicine*, *6*:7.

Moreira, M.T., Smith, L.A., Foxcroft, D. (2009). Social norms interventions to reduce alcohol misuse in university or college students. *Cochrane Database of Systematic Reviews*, *3*:CD006748.

National Institute of Health and Clinical Excellence (NICE) (2006). Four commonly used methods to increase physical activity: brief interventions in primary care, exercise referral schemes, pedometers and community-based exercise programmes for walking and cycling (Public Health Intervention Guidance 2). National Institute of Health and Clinical Excellence.

National Institute of Health and Clinical Excellence (NICE) (2007). Behaviour change at population, community and individual levels (Public Health Guidance 6). National Institute of Health and Clinical Excellence.

O'Connor, D., Green, S., & Higgins, J.P.T. (2008). Chapter 5: Defining the review question and developing criteria for including studies. In: Higgins, J.P.T., Green, S., (Eds). Cochrane Handbook for Systematic Reviews of Interventions version 5.0.0 [updated February 2008]. The Cochrane Collaboration.

Rodgers, M., Sowden, A., Petticrew, M., Arai, L., Roberts, H., Britten, N., & Popay, J. (2009). Testing Methodological Guidance on the Conduct of Narrative Synthesis in Systematic Reviews: Effectiveness of Interventions to Promote Smoke Alarm Ownership and Function. *Evaluation*, *15*, 47-71.

Rogers, R. W. (1983). Cognitive and physiological processes in fear appeals and attitude change: A revised theory of protection motivation. In Cacioppo, B.L. & Petty, L.L. (Eds.), *Social psychophysiology: A source book* (pp. 153-176). London: Guildford Press.

Rozin, P., Scott, S., Dingley, M., Urbanek, J.K., Jiang, H., & Kaltenbach, M. (2011). Nudge to nobesity I: Minor changes in accessibility decrease food intake. *Judgment and Decision Making*, *6*(4), 323–332

Sallis, J.F., Bauman, A., Pratt, M. (1998). Environmental and Policy Interventions to Promote Physical Activity. *American Journal of Preventive Medicine*, *15*(4), 379-397.

Schwarzer, R. (2001). Social-Cognitive Factors in Changing Health-Related Behaviors. *Current Directions in Psychological Science*, *10*(2), 47-51.

Shemilt, I., Hollands, G.J., Marteau, T.M., Jebb, S.A., Kelly, M.P., Nakamura, R., Suhrcke, M., Ogilvie, D. (2013). Economic instruments for population diet and physical activity behaviour change: a systematic scoping review. PLOS ONE; 8(9):e75070.

Shemilt, I., Simon, A., Hollands, G.J., Marteau, T.M., Ogilvie, D., O'Mara-Eves, A., Kelly, M.P., Thomas, J. (2013). Pinpointing needles in giant haystacks: use of text mining to reduce impractical screening workload in extremely large scoping reviews. *Research Synthesis Methods*; DOI: 10.1002/jrsm.1093.

Sobal, J., & Wansink, B. (2007). Kitchenscapes, tablescapes, platescapes, and foodscapes: Influence of microscale built environments on food intake. *Environment and Behavior*, *39*, 124–142.

Solar, O., & Irwin, A. (2010). A conceptual framework for action on the social determinants of health (Social Determinants of Health Discussion Paper 2 (Policy and Practice)). Geneva:WHO.

Soler, R.E., Leeks, K.D., Buchanan, L.R., Brownson, R.C., Heath, G.W., & Hopkins, D.H. (2010). Point-of-decision prompts to increase stair use: a systematic review update. *American Journal of Preventive Medicine*, *38*, 292–300.

Steenhuis, I.H.M., & Vermeer, W.M. (2009). Portion size: review and framework for interventions. *International Journal of Behavioral Nutrition and Physical Activity*, *6*:58.

Strack, F., & Deutsch, R. (2004). Reflective and impulsive determinants of social behaviour. *Personality and Social Psychology Review*, *8*, 220-247.

Stroebele, N., & De Castro, J.M. (2004). Effect of ambience on food intake and food choice. *Nutrition*, *20*(9), 821-38.

Suhrcke, M., Nugent, R.A., Stuckler, D., & Rocco L. (2006). Chronic Disease: An Economic Perspective. London: Oxford Health Alliance

Swinburn, B., Egger, G., & Raza, F. (1999). Dissecting obesogenic environments: the development and application of a framework for identifying and prioritizing environmental interventions for obesity. *Preventive Medicine*, *29*, 563–570.

Thaler, R.H., & Sunstein, C.R. (2008). Nudge: Improving Decisions About Health, Wealth, and Happiness. New Haven,CT: Yale Univ. Press.

Thomas, J., Brunton, J., & Graziosi, S. (2010) EPPI-Reviewer 4.0: software for research synthesis. EPPI-Centre Software. London: Social Science Research Unit, Institute of Education.

Thorndike, A.N., Sonnenberg, L., Riis, J., Barraclough, S., & Levy, D.E. (2012). A 2-Phase Labeling and Choice Architecture Intervention to Improve Healthy Food and Beverage Choices. *American Journal of Public Health, online pre-print.*

Turley, L.W., & Milliman, R. (2000). Atmospheric Effects on Shopping Behavior: A Review of the Experimental Evidence. *Journal of Business Research*, *49*, 193–211.

Wansink, B. (2004). Environmental factors that increase the food intake and consumption volume of unknowing consumers. *Annual Review of Nutrition*, *24*, 455-479.

Webb, T.L., & Sheeran, P. (2006). Does changing behavioural intentions engender behavior change? A meta-analysis of the experimental evidence. *Psychological Bulletin*, *132*, 249-268.

Wiers, R.W., Rinck, M., Kordts, R., Houben, K., & Strack, F. (2010) Retraining automatic action-tendencies to approach alcohol in hazardous drinkers. *Addiction*, *105*(2), 279–287.

World Health Organization (2011). World Health Statistics 2011. Geneva: World Health Organization.

WHO Regional Committee for Europe (2008). Behaviour change strategies and health: the role of health systems. Geneva: World Health Organization.

Included articles by intervention type

AMBIENCE

Areni, C. S., & Kim, D. (1993). The influence of background music on shopping behavior: Classical versus top-forty music in a wine store. *Advances in Consumer Research, 20*, 336-340.

Areni, C. S., & Kim, D. (1994). The influence of in-store lighting on consumers' examination of merchandise in a wine store. *International Journal of Research in Marketing, 11*(2), 117-125.

Barwood, M. J., Weston, N. J. V., Thelwell, R., & Page, J. (2009). A motivational music and video intervention improves high-intensity exercise performance. *Journal of Sports Science and Medicine, 8*(3), 435-442.

Bell, R., Meiselman, H. L., Pierson, B. J., & Reeve, W. G. (1994). Effects of adding an Italian theme to a restaurant on the perceived ethnicity, acceptability, and selection of foods. *Appetite, 22*(1), 11-24.

Bellisle, F., & Dalix, A.-M. (2001). Cognitive restraint can be offset by distraction, leading to increased meal intake in women. *The American Journal of Clinical Nutrition, 74*(2), 197-200.

Bellisle, F., Dalix, A. M., & Slama, G. (2004). Non food-related environmental stimuli induce increased meal intake in healthy women: Comparison of television viewing versus listening to a recorded story in laboratory settings. *Appetite, 43*(2), 175-180.

Bellisle, F., Dalix, A.-M., Airinei, G., Hercberg, S., & Péneau, S. (2009). Influence of dietary restraint and environmental factors on meal size in normal-weight women. A laboratory study. *Appetite, 53*(3), 309-313.

Blass, E. M., Anderson, D. R., Kirkorian, H. L., Pempek, T. A., Price, I., & Koleini, M. F. (2006). On the road to obesity: Television viewing increases intake of high-density foods. *Physiology & Behavior, 88*(4-5), 597-604.

Boutelle, K. N., Jeffery, R. W., Murray, D. M., & Schmitz, M. K. (2001). Using signs, artwork, and music to promote stair use in a public building. *American Journal of Public Health, 91*(12), 2004-2006.

Caldwell, C., & Hibbert, S. A. (1999). Play that one again: The effect of music tempo on consumer behaviour in a restaurant *European Advances in Consumer Research, 4*, 58-62.

Caldwell, C., & Hibbert, S. A. (2002). The influence of music tempo and musical preference on restaurant patrons' behavior. *Psychology and Marketing, 19*(11), 895-917.

Cardon, G., Labarque, V., Smits, D., & Bourdeaudhuij, I. D. (2009). Promoting physical activity at the pre-school playground: The effects of providing markings and play equipment. *Preventive Medicine, 48*(4), 335-340.

Chandon, P., & Wansink, B. (2011). Is food marketing making us fat? A multi-disciplinary review *Faculty & Research Working Paper*. Fountainbleau, France: Instead: The Business School for the World.

Coelho, J. S., Polivy, J., Peter, H. C., & Pliner, P. (2009). Wake up and smell the cookies. Effects of olfactory food-cue exposure in restrained and unrestrained eaters. *Appetite, 52*(2), 517-520.

Fedoroff, I., Polivy, J., & Peter, H. C. (2003). The specificity of restrained versus unrestrained eaters' responses to food cues: General desire to eat, or craving for the cued food? *Appetite, 41*(1), 7-13.

Francis, L. A., & Birch, L. L. (2006). Does eating during television viewing affect preschool children's intake? *Journal of the American Dietetic Association, 106*(4), 598-600.

Gueguen, N., Le Guellec, H., & Jacob, C. (2004). Sound level of background music and alcohol consumption: An empirical evaluation. *Perceptual and Motor Skills, 99*(1), 34-38.

Gueguen, N., & Petr, C. (2006). Odors and consumer behavior in a restaurant. *International Journal of Hospitality Management, 25*(2), 335-339.

Gueguen, N., Jacob, C., Le Guellec, H., Morineau, T., & Lourel, M. (2008). Sound level of environmental music and drinking behavior: A field experiment with beer drinkers. *Alcoholism, Clinical and Experimental Research, 32*(10), 1795-1798.

Hetherington, M. M., Anderson, A. S., Norton, G. N., & Newson, L. (2006). Situational effects on meal intake: A comparison of eating alone and eating with others. *Physiology & Behavior, 88*(4-5), 498-505.

Hetherington, M. M. (2007). Cues to overeat: Psychological factors influencing overconsumption. *Proceedings of the Nutrition Society, 66*(1), 113-123.

Higgs, S., & Woodward, M. (2009). Television watching during lunch increases afternoon snack intake of young women. *Appetite, 52*(1), 39-43.

Hughes, K., Quigg, Z., Eckley, L., Bellis, M., Jones, L., Calafat, A., . . . van, H. (2011). Environmental factors in drinking venues and alcohol-related harm: The evidence base for European intervention. *Addiction, 106*(Suppl.1), 37-46.

Jacob, C. (2006). Styles of background music and consumption in a bar: An empirical evaluation. *International Journal of Hospitality Management, 25*(4), 716-720.

Kerr, N. A., Yore, M. M., Ham, S. A., & Dietz, W. H. (2004). Increasing stair use in a worksite through environmental changes. *American Journal of Health Promotion, 18*(4), 312-315.

Loucaides, C. A., Jago, R., & Charalambous, I. (2009). Promoting physical activity during school break times: Piloting a simple, low cost intervention. *Preventive Medicine, 48*(4), 332-334.

Mathey, M. F. A. M., Vanneste, V. G. G., de Graaf, C., de Groot, L. C. P. G. M., & van Staveren, W. A. (2001). Health effect of improved meal ambiance in a Dutch nursing home: A 1-year intervention study. *Preventive Medicine, 32*(5), 416-423.

McCarron, A., & Tierney, K. J. (1989). The effect of auditory stimulation on the consumption of soft drinks. *Appetite, 13*(2), 155-159.

Milliman, R. E. (1982). Using background music to affect the behavior of supermarket shoppers. *The Journal of Marketing, 46*(3), 86-91.

Milliman, R. E. (1986). The influence of background music on the behavior of restaurant patrons. *Journal of Consumer Research, 13*(2), 286-289.

Moray, J., Fu, A., Brill, K., & Mayoral, M. S. (2007). Viewing television while eating impairs the ability to accurately estimate total amount of food consumed. *Bariatric Nursing and Surgical Patient Care, 2*(1), 71-76.

North, A. C., Hargreaves, D. J., & McKendrick, J. (1997). In-store music affects product choice. *Nature, 390*(6656), 132.

North, A. C., & Hargreaves, D. J. (1998). The effect of music on atmosphere and purchase intentions in a cafeteria. *Journal of Applied Social Psychology, 28*(24), 2254-2273.

North, A. C., Hargreaves, D. J., & McKendrick, J. (1999). The influence of in-store music on wine selections. . *Journal of Applied Psychology, 84*(2), 271-276.

North, A. C., Shilcock, A., & Hargreaves, D. J. (2003). The effect of musical style on restaurant customers' spending. *Environment and Behavior, 35*(5), 712-718.

Peck, J., & Childers, T. L. (2008). If it tastes, smells, sounds, and feels like a duck, then it must be a….: Effects of sensory factors on consumer behaviors. In P. M. Haugtvedt, & F. R. Kardes (Ed.), *Handbook of consumer psychology* (pp. 193-219). New York: Psychology Press.

Peneau, S., Mekhmoukh, A., Chapelot, D., Dalix, A.-M., Airinei, G., Hercberg, S., & Bellisle, F. (2009). Influence of environmental factors on food intake and choice of beverage during meals in teenagers: A laboratory study. *British Journal of Nutrition, 102*(12), 1854-1859.

Poothullil, J. M. (2002). Role of oral sensory signals in determining meal size in lean women. *Nutrition, 18*(6), 479-483.

Quartier, K., Christiaans, H., & Van Cleempoel, K. (2009). *Retail design: Lighting as an atmospheric tool, creating experiences which influence consumers' mood and behaviour in commercial spaces.* Paper presented at the Undisciplined! Design Research Society Conference 2008, Sheffield, UK.

Ridgers, N. D., Stratton, G., Fairclough, S. J., & Twisk, J. W. R. (2007). Long-term effects of a playground markings and physical structures on children's recess physical activity levels. *Preventive Medicine, 44*(5), 393-397.

Rogers, Y., Hazlewood, W. R., Marshall, P., Dalton, N., & Hetrich, S. (2010). Ambient influence: Can twinkly lights lure and abstract representations trigger behavioural change? *UbiComp 2010, 26-29*, 261-270.

Smith, J. M., & Ditschun, T. L. (2009). Controlling satiety: How environmental factors influence food intake. *Trends in Food Science & Technology, 20*(6-7), 271-277.

Sobal, J., & Wansink, B. (2007). Kitchenscapes, tablescapes, platescapes, and foodscapes. *Environment and Behavior, 39*(1), 124-142.

Spence, C., & Shankar, M. U. (2010). The influence of auditory cues on the perception of, and responses to, food and drink. *Journal of Sensory Studies, 25*(3), 406-430.

Stratton, G., & Mullan, E. (2005). The effect of multicolor playground markings on children's physical activity level during recess. *Preventive Medicine, 41*(5-6), 828-833.

Stroebele, N., & de Castro, J. M. (2004). Effect of ambience on food intake and food choice. *Nutrition, 20*(9), 821-838.

Turley, L. W., & Milliman, R. E. (2000). Atmospheric effects on shopping behavior: A review of the experimental evidence. *Journal of Business Research, 49*(2), 193-211.

van Nieuw-Amerongen, M. E., Kremers, S. P. J., de Vries, N. K., & Kok, G. (2011). The use of prompts, increased accessibility, visibility, and aesthetics of the stairwell to promote stair use in a university building. *Environment and Behavior, 43*(1), 131-139.

Wansink, B. (2004). Environmental factors that increase the food intake and consumption volume of unknowing consumers. *Annual Review of Nutrition, 24*, 455-479.

Webb, O. J., & Eves, F. F. (2007). Effects of environmental changes in a stair climbing intervention: generalization to stair descent. *American Journal of Health Promotion, 22*(1), 38-44.

AVAILABILITY

Anderson, A. S., Porteous, L. E. G., Foster, E., Higgins, C., Stead, M., Hetherington, M., . . . Adamson, A. J. (2005). The impact of a school-based nutrition education intervention on dietary intake and cognitive and attitudinal variables relating to fruits and vegetables. *Public Health Nutrition, 8*(6), 650-656.

Angelopoulos, P. D., Milionis, H. J., Grammatikaki, E., Moschonis, G., & Manios, Y. (2009). Changes in BMI and blood pressure after a school based intervention: The CHILDREN study. *European Journal of Public Health, 19*(3), 319-325.

Bartholomew, J. B., & Jowers, E. M. (2006). Increasing frequency of lower-fat entrees offered at school lunch: An environmental change strategy to increase healthful selections. *Journal of the American Dietetic Association, 106*(2), 248-252.

Chandon, P., & Wansink, B. (2002). When are stockpiled products consumed faster? A convenience-salience framework of postpurchase consumption incidence and quantity. *Journal of Marketing Research, 39*(3), 321-335.

Chandon, P., & Wansink, B. (2011). Is food marketing making us fat? A multi-disciplinary review *Faculty & Research Working Paper*. Fountainbleau, France: Instead: The Business School for the World.

Engbers, L. H., van Poppel, M. N. M., Chin A Paw, M. J. M., & van Mechelen, W. (2005). Worksite health promotion programs with environmental changes: A systematic review. *American Journal of Preventive Medicine, 29*(1), 61-70.

Eves, F. F., Lewis, A. L., & Griffin, C. (2008). Modelling effects of stair width on rates of stair climbing in a train station. *Preventive Medicine, 47*(3), 270-272.

Faskunger, J., Poortvliet, E., Nylund, K., & Rossen, J. (2003). Effect of an environmental barrier to physical activity on commuter stair use. *Scandinavian Journal of Nutrition [Naringsforskning], 47*(1), 26-28.

Fiske, A., & Cullen, K. W. (2004). Effects of promotional materials on vending sales of low-fat items in teachers' lounges. *Journal of the American Dietetic Association, 104*(1), 90-93.

French, S. A., Story, M., Fulkerson, J. A., & Hannan, P. (2004). An environmental intervention to promote lower-fat food choices in secondary schools: Outcomes of the TACOS study. *American Journal of Public Health, 94*(9), 1507-1512.

French, S. A., Hannan, P. J., Harnack, L. J., Mitchell, N. R., Toomey, T. L., & Gerlach, A. (2010). Pricing and availability intervention in vending machines at four bus garages. *Journal of Occupational and Environmental Medicine, 52* (Suppl.1), s29-s33.

Glanz, K., & Mullis, R. M. (1988). Environmental interventions to promote healthy eating: A review of models, programs, and evidence. *Health Education & Behavior, 15*(4), 395-415.

Glanz, K., & Hoelscher, D. (2004). Increasing fruit and vegetable intake by changing environments, policy and pricing: Restaurant-based research, strategies, and recommendations. *Preventive Medicine, 39* (Suppl.2), s88-s93.

Goldberg, M. E., & Gunasti, K. (2007). Creating an environment in which youths are encouraged to eat a healthier diet. *Journal of Public Policy & Marketing, 26*(2), 162-181.

Jeffery, R. W., French, S. A., Raether, C., & Baxter, J. E. (1994). An environmental intervention to increase fruit and salad purchases in a cafeteria. *Preventive Medicine, 23*(6), 788-792.

Kimathi, A. N., Gregoire, M. B., Dowling, R. A., & Stone, M. K. (2009). A healthful options food station can improve satisfaction and generate gross profit in a worksite cafeteria. *Journal of the American Dietetic Association 109*(5), 914-917.

Lowe, M. R., Tappe, K. A., Butryn, M. L., Annunziato, R. A., Coletta, M. C., Ochner, C. N., & Rolls, B. J. (2010). An intervention study targeting energy and nutrient intake in worksite cafeterias. *Eating Behaviors, 11*(3), 144-151.

Nicoll, G., & Zimring, C. (2009). Effect of innovative building design on physical activity. *Journal of Public Health Policy, 30* (Suppl.1), s111-s123.

Olander, E. K., & Eves, F. F. (2011). Elevator availability and its impact on stair use in a workplace. *Journal of Environmental Psychology, 31*(2), 200-206.

Patrick, H., & Nicklas, T. A. (2005). A review of family and social determinants of children's eating patterns and diet quality. *Journal of the American College of Nutrition, 24*(2), 83-92.

Perlmutter, C. A., Canter, D. D., & Gregoire, M. B. (1997). Profitability and acceptability of fat- and sodium-modified hot entrees in a worksite cafeteria. *Journal of the American Dietetic Association, 97*(4), 391-395.

Perry, C. L., Bishop, D. B., Taylor, G. L., Davis, M., Story, M., Gray, C., . . . Harnack, L. (2004). A randomized school trial of environmental strategies to encourage fruit and vegetable consumption among children. *Health Education & Behavior, 31*(1), 65-76.

Richard, L., O'Loughlin, J., Masson, P., & Devost, S. (1999). Healthy menu intervention in restaurants in low-income neighbourhoods: A field experience. *Journal of Nutrition Education, 31*(1), 54-59.

Seymour, J. D., Yaroch, A. L., Serdula, M., Blanck, H. M., & Khan, L. K. (2004). Impact of nutrition environmental interventions on point-of-purchase behavior in adults: A review. *Preventive Medicine, 39* (Suppl.2), s108-s136.

Shepherd, J., Harden, A., Rees, R., Brunton, G., Garcia, J., Oliver, S., & Oakley, A. (2006). Young people and healthy eating: A systematic review of research on barriers and facilitators. *Health Education Research, 21*(2), 239-257.

Sobal, J., & Wansink, B. (2007). Kitchenscapes, tablescapes, platescapes, and foodscapes. *Environment and Behavior, 39*(1), 124-142.

Sorensen, L. B., Moller, P., Flint, A., Martens, M., & Raben, A. (2003). Effect of sensory perception of foods on appetite and food intake: A review of studies on humans. *International Journal on Obesity and Related Metabolic Disorders, 27*(10), 1152-1166.

Sorensen, G., Linnan, L., & Hunt, M. K. (2004). Worksite-based research and initiatives to increase fruit and vegetable consumption. *Preventive Medicine, 39* (Suppl.2), s94-s100.

Steenhuis, I., van Assema, P., van Breukelen, G., Glanz, K., Kok, G., & de Vries, H. (2004). The impact of educational and environmental interventions in Dutch worksite cafeterias. *Health Promotion International, 19*(3), 335-343.

van Houten, R. V., Nau, P. A., & Merrigan, M. (1981). Reducing elevator energy use: A comparison of posted feedback and reduced elevator convenience. *Journal of Applied Behavior Analysis, 14*(4), 377-387.

Wansink, B. (2004). Environmental factors that increase the food intake and consumption volume of unknowing consumers. *Annual Review of Nutrition, 24*, 455-479.

Whitaker, R. C., Wright, J. A., Finch, A. J., & Psaty, B. M. (1993). An environmental intervention to reduce dietary fat in school lunches. *Pediatrics, 91*(6), 1107-1111.

Wilbur, C. S., Zifferblatt, S. M., Pinsky, J. L., & Zifferblatt, S. (1981). Healthy vending: A cooperative pilot research program to stimulate good health in the marketplace. *Preventive Medicine, 10*(1), 85-93.

Wilcox, K., Vallen, B., Block, L., & Fitzsimons, G. J. (2009). Vicarious goal fulfillment: When the mere presence of a healthy option leads to an ironically indulgent decision. *Journal of Consumer Research, 36*(3), 380-393.

FUNCTIONAL DESIGN

Ahn, H. J., Han, K. A., Kwon, H. R., & Min, K. W. (2010). The small rice bowl-based meal plan was effective at reducing dietary energy intake, body weight, and blood glucose levels in Korean women with type 2 diabetes mellitus. *Korean Diabetes Journal, 34*(6), 340-349.

Andrade, A. M., Greene, G. W., & Melanson, K. J. (2008). Eating slowly led to decreases in energy intake within meals in healthy women. *Journal of the American Dietetic Association, 108*(7), 1186-1191.

Benden, M. E., Blake, J. J., Wendel, M. L., & Huber, J. C. (2011). The impact of stand-biased desks in classrooms on calorie expenditure in children. *American Journal of Public Health, 101*(8), 1433-1436.

Bohnert, A. M., Randall, E. T., Tharp, S., & Germann, J. (2011). The development and evaluation of a portion plate for youth: A pilot study. *Journal of Nutrition Education & Behavior, 43*(4), 268-273.

Cardon, G., Labarque, V., Smits, D., & Bourdeaudhuij, I. D. (2009). Promoting physical activity at the pre-school playground: The effects of providing markings and play equipment. *Preventive Medicine, 48*(4), 335-340.

Chandon, P., & Wansink, B. (2011). Is food marketing making us fat? A multi-disciplinary review *Faculty & Research Working Paper*. Fountainbleau, France: Instead: The Business School for the World.

Engbers, L. H., van Poppel, M. N. M., Chin A Paw, M. J. M., & van Mechelen, W. (2005). Worksite health promotion programs with environmental changes: A systematic review. *American Journal of Preventive Medicine, 29*(1), 61-70.

Geier, A. B., Rozin, P., & Doros, G. (2006). Unit bias. A new heuristic that helps explain the effect of portion size on food intake. *Psychological Science, 17*(6), 521-525.

Goldberg, M. E., & Gunasti, K. (2007). Creating an environment in which youths are encouraged to eat a healthier diet. *Journal of Public Policy & Marketing, 26*(2), 162-181.

Hogenkamp, P. S., Mars, M., Stafleu, A., & de, G. (2010). Intake during repeated exposure to low- and high-energy-dense yogurts by different means of consumption. *American Journal of Clinical Nutrition, 91*(4), 841-847.

Hughes, K., Quigg, Z., Eckley, L., Bellis, M., Jones, L., Calafat, A., . . . van, H. (2011). Environmental factors in drinking venues and alcohol-related harm: The evidence base for European intervention. *Addiction, 106*(Suppl.1), 37-46.

Jenum, A. K., Lorentzen, C. A. N., & Ommundsen, Y. (2009). Targeting physical activity in a low socioeconomic status population: Observations from the Norwegian "Romsas in Motion" study. *British Journal of Sports Medicine, 43*(1), 64-69.

Just, D., & Wansink, B. (2009). Smarter lunchrooms: Using behavioral economics to improve meal selection. *Choices, 29*(3).

Kalnikaitė, V., Yvonne, R., Bird, J., Villar, N., Bachour, K., Payne, S., . . . Krüger, A. (2011). *How to nudge in situ: Designing lambent devices to deliver information salience in supermarkets.* Paper presented at the 13th ACM International conference on Ubiquitous computing, New York, NY, United States.

Koh, J., & Pliner, P. (2009). The effects of degree of acquaintance, plate size, and sharing on food intake. *Appetite, 52*(3), 595-602.

Lanningham-Foster, L., Foster, R. C., McCrady, S. K., Manohar, C. U., Jensen, T. B., Mitre, N. G., . . . Levine, J. A. (2008). Changing the school environment to increase physical activity in children. *Obesity (Silver Spring), 16*(8), 1849-1853.

Lombardini-Riipinen, C., & Lankoski, L. (2010). *Take off the heater: Utility effect and food environment effect in food consumption decisions.* Paper presented at the 1st Joint EAAE/AAEA Seminar, Munich, Germany.

Loucaides, C. A., Jago, R., & Charalambous, I. (2009). Promoting physical activity during school break times: Piloting a simple, low cost intervention. *Preventive Medicine, 48*(4), 332-334.

Mishra, A., Mishra, H., & Masters, T. M. (2011). The influence of bite-size on quantity of food consumed: A field study. *Journal of Consumer Research*.

Ridgers, N., Stratton, G., Fairclough, S., & Twisk, J. (2007). Children's physical activity levels during school recess: A quasi-experimental intervention study. *International Journal of Behavioral Nutrition and Physical Activity, 4*(1), 19.

Ridgers, N. D., Stratton, G., Fairclough, S. J., & Twisk, J. W. R. (2007). Long-term effects of a playground markings and physical structures on children's recess physical activity levels. *Preventive Medicine, 44*(5), 393-397.

Ridgers, N. D., Fairclough, S. J., & Stratton, G. (2010). Twelve-month effects of a playground intervention on children's morning and lunchtime recess physical activity levels. *Journal of Physical Activity & Health, 7*(2), 167-175.

Rolls, B. J., Roe, L. S., Halverson, K. H., & Meengs, J. S. (2007). Using a smaller plate did not reduce energy intake at meals. *Appetite, 49*(3), 652-660.

Rozin, P., Scott, S., Dingley, M., Urbanek, J. K., Jiang, H., & Kaltenbach, M. (2011). Nudge to nobesity I: Minor changes in accessibility decrease food intake. *Judgment and Decision Making, 6*(4), 323-332.

Shah, M., Schroeder, R., Winn, W., & Adams-Huet, B. (2011). A pilot study to investigate the effect of plate size on meal energy intake in normal weight and overweight/obese women. *Journal of Human Nutrition and Dietetics, 24*(6), 612-615.

Smith, J. M., & Ditschun, T. L. (2009). Controlling satiety: How environmental factors influence food intake. *Trends in Food Science & Technology, 20*(6-7), 271-277.

Sobal, J., & Wansink, B. (2007). Kitchenscapes, tablescapes, platescapes, and foodscapes. *Environment and Behavior, 39*(1), 124-142.

Stratton, G., & Mullan, E. (2005). The effect of multicolor playground markings on children's physical activity level during recess. *Preventive Medicine, 41*(5-6), 828-833.

Stroebele, N., & de Castro, J. M. (2004). Effect of ambience on food intake and food choice. *Nutrition, 20*(9), 821-838.

Thompson, W. G., Foster, R. C., Eide, D. S., & Levine, J. A. (2008). Feasibility of a walking workstation to increase daily walking. *British Journal of Sports Medicine, 42*(3), 225-228.

Van den Bergh, B., Schmitt, J., & Warlop, L. (2011). Embodied Myopia. *Journal of Marketing Research, 48*(6), 1033-1044.

van Kleef, E., Shimizu, M., & Wansink, B. (2011). Serving bowl selection biases the amount of food served. *Journal of Nutrition Education and Behavior, 44*(1), 66-70.

Wansink, B., & van Ittersum, K. (2003). Bottoms up! The influence of elongation on pouring and consumption volume. *Journal of Consumer Research, 30*(3), 455-463.

Wansink, B., & Cheney, M. M. (2005). Super bowls: Serving bowl size and food consumption. *Journal of the American Medical Association, 293*(14), 1727-1728.

Wansink, B., & van Ittersum, K. (2005). Shape of glass and amount of alcohol poured: Comparative study of effect of practice and concentration. *British Medical Journal, 331*(7531), 1512-1514.

Wansink, B., van Ittersum, K., & Painter, J. E. (2006). Ice cream illusions: Bowls, spoons, and self-served portion sizes. *American Journal of Preventive Medicine, 31*(3), 240-243.

Wansink, B. (2010). From mindless eating to mindlessly eating better. *Physiology & Behavior, 100*(5), 454-463.

LABELLING

Antonuk, B., & Block, L. G. (2006). The effect of single serving versus entire package nutritional information on consumption norms and actual consumption of a snack food. *Journal of Nutrition Education and Behavior, 38*(6), 365-370.

Aron, J. I., Evans, R. E., & Mela, D. J. (1995). Paradoxical effect of a nutrition labelling scheme in a student cafeteria. *Nutrition Research, 15*(9), 1251-1261.

Bailey, J., Poole, R., Zinovieff, F., Robinson, C. A., Parry, O., Tocque, K., & Kennedy, L. (2011). Achieving positive change in the drinking culture of Wales *Research report*. Wrexham and Bangor, Wales: Glyndwr University / Bangor University.

Bassett, M. T., Dumanovsky, T., Huang, C., Silver, L. D., Young, C., Nonas, C., . . . Frieden, T. R. (2008). Purchasing behavior and calorie information at fast-food chains in New York City, 2007. *American Journal of Public Health, 98*(8), 1457-1459.

Bergen, D., & Yeh, M. C. (2006). Effects of energy-content labels and motivational posters on sales of sugar-sweetened beverages: Stimulating sales of diet drinks among adults study. *Journal of the American Dietetic Association, 106*(11), 1866-1869.

Bollinger, B., Leslie, P., & Sorensen, A. (2011). Calorie posting in chain restaurants. *American Economic Journal: Economic Policy, 3*(1), 91-128.

Borgmeier, I., & Westenhoefer, J. (2009). Impact of different food label formats on healthiness evaluation and food choice of consumers: A randomized-controlled study. *BMC Public Health, 9*, 184.

Borland, R. (1997). Tobacco health warnings and smoking-related cognitions and behaviours. *Addiction, 92*(11), 1427-1435.

Borland, R., Wilson, N., Fong, G. T., Hammond, D., Cummings, K. M., Yong, H. H., . . . McNeill, A. (2009). Impact of graphic and text warnings on cigarette packs: Findings from four countries over five years. *Tobacco Control, 18*(5), 358-364.

Burton, S., Creyer, E. H., Kees, J., & Huggins, K. (2006). Attacking the obesity epidemic: The potential health benefits of providing nutrition information in restaurants. *American Journal of Public Health, 96*(9), 1669-1675.

Chandon, P., & Wansink, B. (2011). Is food marketing making us fat? A multi-disciplinary review *Faculty & Research Working Paper*. Fountainbleau, France: Instead: The Business School for the World.

Chu, Y. H., Frongillo, E. A., Jones, S. J., & Kaye, G. L. (2009). Improving patrons' meal selections through the use of point-of-selection nutrition labels. *American Journal of Public Health, 99*(11), 2001-2005.

Cinciripini, P. M. (1984). Changing food selections in a public cafeteria. *Behavior Modification, 8*(4), 520-539.

Devlin, E., Anderson, S., Hastings, G., & Macfadyen, L. (2005). Targeting smokers via tobacco product labelling: Opportunities and challenges for Pan European health promotion. *Health Promotion International, 20*(1), 41-49.

Dixon, H., Scully, M., Wakefield, M., Kelly, B., Chapman, K., & Donovan, R. (2011). Parent's responses to nutrient claims and sports celebrity endorsements on energy-dense and nutrient-poor foods: An experimental study. *Public Health Nutrition, 14*(6), 1071-1079.

Dubbert, P. M., Johnson, W. G., Schlundt, D. G., & Montague, N. W. (1984). The influence of caloric information on cafeteria food choices. *Journal of Applied Behavior Analysis, 17*(1), 85-92.

Dumanovsky, T., Huang, C. Y., Nonas, C. A., Matte, T. D., Bassett, M. T., & Silver, L. D. (2011). Changes in energy content of lunchtime purchases from fast food restaurants after introduction of calorie labelling: Cross sectional customer surveys. *British Medical Journal, 343*.

Elbel, B., Kersh, R., Brescoll, V. L., & Dixon, L. B. (2009). Calorie labeling and food choices: A first look at the effects on low-income people In New York City. *Health Affairs, 28*(6), w1110-w1121.

Elbel, B., Gyamfi, J., & Kersh, R. (2011). Child and adolescent fast-food choice and the influence of calorie labeling: A natural experiment. *International Journal of Obesity, 35*(4), 493-500.

Engbers, L. H., van Poppel, M. N. M., Chin A Paw, M. J. M., & van Mechelen, W. (2005). Worksite health promotion programs with environmental changes: A systematic review. *American Journal of Preventive Medicine, 29*(1), 61-70.

Engbers, L. H., van Poppel, M. N., Chin, A. P. M., & van Mechelen, W. (2006). The effects of a controlled worksite environmental intervention on determinants of dietary behavior and self-reported fruit, vegetable and fat intake. *BMC Public Health, 6*, 253.

Finkelstein, E. A., Strombotne, K. L., Chan, N. L., & Krieger, J. (2011). Mandatory menu labeling in one fast-food chain in King County, Washington. *American Journal of Preventive Medicine, 40*(2), 122-127.

Fong, G. T., Hammond, D., & Hitchman, S. C. (2009). The impact of pictures on the effectiveness of tobacco warnings. *Bulletin of the World Health Organization, 87*(8), 640-643.

Freedman, M. R., & Connors, R. (2010). Point-of-purchase nutrition information influences food-purchasing behaviors of college students: A pilot study. *Journal of the American Dietetic Association, 110*(8), 1222-1226.

French, S. A., Jeffery, R. W., Story, M., Breitlow, K. K., Baxter, J. S., Hannan, P., & Snyder, M. P. (2001). Pricing and promotion effects on low-fat vending snack purchases: The CHIPS Study. *American Journal of Public Health, 91*(1), 112-127.

Garg, N., Wansink, B., & Inman, J. J. (2007). The influence of incidental affect on consumers' food intake. *Journal of Marketing, 71*(1), 194-206.

Gerend, M. A. (2009). Does calorie information promote lower calorie fast food choices among college students? *Journal of Adolescent Health, 44*(1), 84-86.

Giesen, J. C., Payne, C. R., Havermans, R. C., & Jansen, A. (2011). Exploring how calorie information and taxes on high-calorie foods influence lunch decisions. *The American Journal of Clinical Nutrition, 93*(4), 689-694.

Gittelsohn, J., Vijayadeva, V., Davison, N., Ramirez, V., Cheung, L. W., Murphy, S., & Novotny, R. (2010). A food store intervention trial improves caregiver psychosocial factors and children's dietary intake in Hawaii. *Obesity (Silver Spring), 18* (Suppl.1), s84-s90.

Glanz, K., & Mullis, R. M. (1988). Environmental interventions to promote healthy eating: A review of models, programs, and evidence. *Health Education & Behavior, 15*(4), 395-415.

Glanz, K., & Hoelscher, D. (2004). Increasing fruit and vegetable intake by changing environments, policy and pricing: Restaurant-based research, strategies, and recommendations. *Preventive Medicine, 39* (Suppl.2), s88-s93.

Hammond, D., Fong, G. T., McDonald, P. W., Cameron, R., & Brown, K. S. (2003). Impact of the graphic Canadian warning labels on adult smoking behaviour. *Tobacco Control, 12*(4), 391-395.

Hammond, D. (2011). Health warning messages on tobacco products: A review. *Tobacco Control, 20*(5), 327-337.

Harnack, L. J., & French, S. A. (2008). Effect of point-of-purchase calorie labeling on restaurant and cafeteria food choices: A review of the literature. *International Journal of Behavioral Nutriton and Physical Activity, 5*, 51.

Harnack, L. J., French, S. A., Oakes, J. M., Story, M. T., Jeffery, R. W., & Rydell, S. A. (2008). Effects of calorie labeling and value size pricing on fast food meal choices: Results from an experimental trial. *International Journal of Behavioral Nutrition and Physical Activity, 5*, 63.

Hassan, L. M., Shiu, E. M. K., & Michaelidou, N. (2010). The influence of nutrition information on choice: The roles of temptation, conflict and self-control. *Journal of Consumer Affairs, 44*(3), 499-515.

Hoefkens, C., Lachat, C., Kolsteren, P., Van Camp, J., & Verbeke, W. (2011). Posting point-of-purchase nutrition information in university canteens does not influence meal choice and nutrient intake. *American Journal of Clinical Nutrition, 94*(2), 562-570.

Horgen, K. B., & Brownell, K. D. (2002). Comparison of price change and health message interventions in promoting healthy food choices. *Health Psychology, 21*(5), 505-512.

Howlett, E. A., Burton, S., Bates, K., & Huggins, K. (2009). Coming to a restaurant near you? Potential consumer responses to nutrition information disclosure on menus. *Journal of Consumer Research, 36*(3), 494-503.

Hrovat, K. B., Harris, K. Z., Leach, A. D., Russell, B. S., Harris, B. V., & Sprecher, D. L. (1994). The new food label, type of fat, and consumer choice. A pilot study. *Archives of Family Medicine, 3*(8), 690-695.

Jones, S. C., & Gregory, P. (2009). The impact of more visible standard drink labelling on youth alcohol consumption: Helping young people drink (ir)responsibly? *Drug and Alcohol Review, 28*(3), 230-234.

Kelly-Weeder, S., Phillips, K., & Rounseville, S. (2011). Effectiveness of public health programs for decreasing alcohol consumption. *Patient Intelligence, 2011*(3), 29-38.

Kiesel, K., & Villas-Boas, S. B. (2009). Can information costs affect consumer choice? Nutritional labels in a supermarket experiment *Working Paper No.1060R2, International Journal of Industrial Organization*. Berkeley, CA: Department of Agricultural and Resource Economics, University of California, Berkeley.

Kral, T. V., Roe, L. S., & Rolls, B. J. (2002). Does nutrition information about the energy density of meals affect food intake in normal-weight women? *Appetite, 39*(2), 137-145.

Li, J., & Grigg, M. (2009). New Zealand: New graphic warnings encourage registrations with the quitline. *Tobacco Control, 18*(1), 72.

Lin, C.-H., Lin, H.-C., & Lee, S.-H. (2011). The influence of health-related information on variety-seeking behavior: The moderating roles of mood states and gender. *British Food Journal, 113*(11).

Lowe, M. R., Tappe, K. A., Butryn, M. L., Annunziato, R. A., Coletta, M. C., Ochner, C. N., & Rolls, B. J. (2010). An intervention study targeting energy and nutrient intake in worksite cafeterias. *Eating Behaviors, 11*(3), 144-151.

MacKinnon, D. P., Pentz, M. A., & Stacy, A. W. (1993). The alcohol warning label and adolescents: The first year. *American Journal of Public Health, 83*(4), 585-587.

Mathios, A. D. (2000). The impact of mandatory disclosure laws on product choices: An analysis of the salad dressing market. *Journal of Law and Economics, 43*(2), 651-677.

Mayer, J. A., Heins, J. M., Vogel, J. M., Morrison, D. C., Lankester, L. D., & Jacobs, A. L. (1986). Promoting low-fat entree choices in a public cafeteria. *Journal of Applied Behavior Analysis, 19*(4), 397-402.

Mayer, J. A., Dubbert, P. M., & Elder, J. P. (1989). Promoting nutrition at the point of choice: A review. *Health Education Quarterly, 16*(1), 31-43.

Miller, D. L., Castellanos, V. H., Shide, D. J., Peters, J. C., & Rolls, B. J. (1998). Effect of fat-free potato chips with and without nutrition labels on fat and energy intakes. *American Journal of Clinical Nutrition, 68*(2), 282-290.

Moodie, C., MacKintosh, A. M., & Hammond, D. (2010). Adolescents' response to text-only tobacco health warnings: Results from the 2008 UK Youth Tobacco Policy Survey. *European Journal of Public Health, 20*(4), 463-469.

Muller, T. E. (1985). Structural information factors which stimulate the use of nutrition information: A field experiment. *Journal of Marketing Research, 22*(2), 143-157.

Perlmutter, C. A., Canter, D. D., & Gregoire, M. B. (1997). Profitability and acceptability of fat- and sodium-modified hot entrees in a worksite cafeteria. *Journal of the American Dietetic Association, 97*(4), 391-395.

Peterson, S., Duncan, D. P., Null, D. B., Roth, S. L., & Gill, L. (2010). Positive changes in perceptions and selections of healthful foods by college students after a short-term point-of-selection intervention at a dining hall. *Journal of American College Health, 58*(5), 425-431.

Pulos, E., & Leng, K. (2010). Evaluation of a voluntary menu-labeling program in full-service restaurants. *American Journal of Public Health, 100*(6), 1035-1039.

Roberto, C. A., Larsen, P. D., Agnew, H., Baik, J., & Brownell, K. D. (2010). Evaluating the impact of menu labeling on food choices and intake. *American Journal of Public Health, 100*(2), 312-318.

Roefs, A., & Jansen, A. (2004). The effect of information about fat content on food consumption in overweight/obese and lean people. *Appetite, 43*(3), 319-322.

Russo, J., Staelin, R., Nolan, C. A., Russell, G. J., & Metcalf, B. L. (1986). Nutrition information in the supermarket. *Journal of Consumer Research, 13*(1), 48-70.

Sacks, G., Rayner, M., & Swinburn, B. (2009). Impact of front-of-pack 'traffic-light' nutrition labelling on consumer food purchases in the UK. *Health Promotion International, 24*(4), 344-352.

Sacks, G., Tikellis, K., Millar, L., & Swinburn, B. (2011). Impact of 'traffic-light' nutrition information on online food purchases in Australia. *Australian and New Zealand Journal of Public Health, 35*(2), 122-126.

Seymour, J. D., Yaroch, A. L., Serdula, M., Blanck, H. M., & Khan, L. K. (2004). Impact of nutrition environmental interventions on point-of-purchase behavior in adults: A review. *Preventive Medicine, 39* (Suppl.2), s108-s136.

Sharma, S., Wagle, A., Sucher, K., & Bugwadia, N. (2011). Impact of point of selection nutrition information on meal choices at a table-service restaurant. *Journal of Foodservice Business Research, 14*(2), 146-161.

Shide, D. J., & Rolls, B. J. (1995). Information about the fat content of preloads influences energy intake in healthy women. *Journal of the American Dietetic Association, 95*(9), 993-998.

Sorensen, L. B., Moller, P., Flint, A., Martens, M., & Raben, A. (2003). Effect of sensory perception of foods on appetite and food intake: A review of studies on humans. *International Journal on Obesity and Related Metabolic Disorders, 27*(10), 1152-1166.

Sorensen, G., Linnan, L., & Hunt, M. K. (2004). Worksite-based research and initiatives to increase fruit and vegetable consumption. *Preventive Medicine, 39* (Suppl.2), s94-s100.

Steenhuis, I., van Assema, P., van Breukelen, G., Glanz, K., Kok, G., & de Vries, H. (2004). The impact of educational and environmental interventions in Dutch worksite cafeterias. *Health Promotion International, 19*(3), 335-343.

Steenhuis, I. H. M., Kroeze, W., Vyth, E. L., Valk, S., Verbauwen, R., & Seidell, J. C. (2010). The effects of using a nutrition logo on consumption and product evaluation of a sweet pastry. *Appetite, 55*(3), 707-709.

Stockley, C. S. (2001). The effectiveness of strategies such as health warning labels to reduce alcohol-related harms: An Australian perspective. *International Journal of Drug Policy, 12*(2), 153-166.

Stockwell, T. (2006). A review of research into the impacts of alcohol warning labels on attitudes and behaviour *Centre of Addictions Research of BC*. Victoria, BC: Centre for Addictions Research of British Columbia.

Sutherland, L. A., Kaley, L. A., & Fischer, L. (2010). Guiding stars: The effect of a nutrition navigation program on consumer purchases at the supermarket. *American Journal of Clinical Nutrition, 91*(4), s1090-s1094.

Tandon, P. S., Wright, J., Zhou, C., Rogers, C. B., & Christakis, D. A. (2010). Nutrition menu labeling may lead to lower-calorie restaurant meal choices for children. *Pediatrics, 125*(2), 244-248.

Tandon, P. S., Zhou, C., Chan, N. L., Lozano, P., Couch, S. C., Glanz, K., . . . Saelens, B. E. (2011). The impact of menu labeling on fast-food purchases for children and parents. *American Journal of Preventive Medicine, 41*(4), 434-438.

Temple, J. L., Johnson, K., Recupero, K., & Suders, H. (2010). Nutrition labels decrease energy intake in adults consuming lunch in the laboratory. *Journal of the American Dietetic Association, 110*(7), 1094-1097.

Temple, J. L., Johnson, K. M., Archer, K., Lacarte, A., Yi, C., & Epstein, L. H. (2011). Influence of simplified nutrition labeling and taxation on laboratory energy intake in adults. *Appetite, 57*(1), 184-192.

Thorndike, A. N., Sonnenberg, L., Riis, J., Barraclough, S., & Levy, D. E. (2012). A 2-Phase labeling and choice architecture intervention to improve healthy food and beverage choices. *American Journal of Public Health, 102*(3), 527-533.

Ueland, Ø., Cardello, A. V., Merrill, E. P., & Lesher, L. L. (2009). Effect of portion size information on food intake. *Journal of the American Dietetic Association, 109*(1), 124-127.

Vadiveloo, M. K., Dixon, L. B., & Elbel, B. (2011). Consumer purchasing patterns in response to calorie labeling legislation in New York City. *International Journal of Behavioral Nutrition and Physical Activity, 8*, 51.

van Herpen, E., & Trijp, H. C. (2011). Front-of-pack nutrition labels. Their effect on attention and choices when consumers have varying goals and time constraints. *Appetite, 57*(1), 148-160.

Variyam, J. N. (2008). Do nutrition labels improve dietary outcomes? *Health Economics, 17*(6), 695-708.

Vermeer, W. M., Steenhuis, I. H., Leeuwis, F. H., Bos, A. E., de Boer, M., & Seidell, J. C. (2010). Portion size labeling and intended soft drink consumption: The impact of labeling format and size portfolio. *Journal of Nutrition Education and Behavior, 42*(6), 422-426.

Vermeer, W. M., Steenhuis, I. H. M., Leeuwis, F. H., Bos, A. E. R., de Boer, M., & Seidell, J. C. (2011). View the label before you view the movie: A field experiment into the impact of portion size and guideline daily amounts labelling on soft drinks in cinemas. *BMC Public Health, 11*, 438.

Vyth, E. L., Steenhuis, I. H., Vlot, J. A., Wulp, A., Hogenes, M. G., Looije, D. H., . . . Seidell, J. C. (2010). Actual use of a front-of-pack nutrition logo in the supermarket: Consumers' motives in food choice. *Public Health Nutrition, 13*(11), 1882-1889.

Vyth, E. L., Steenhuis, I. H. M., Heymans, M. W., Roodenburg, A. J. C., Brug, J., & Seidell, J. C. (2011). Influence of placement of a nutrition logo on cafeteria menu items on lunchtime food choices at Dutch work sites. *Journal of the American Dietetic Association, 111*(1), 131-136.

Wansink, B., & Chandon, P. (2006). Can "low-fat" nutrition labels lead to obesity? *Journal of Marketing Research, 43*, 605-617.

Wardle, J., & Solomons, W. (1994). Naughty but nice: A laboratory study of health information and food preferences in a community sample. *Health Psychology, 13*(2), 180-183.

Whitaker, R. C., Wright, J. A., Koepsell, T. D., Finch, A. J., & Psaty, B. M. (1994). Randomized intervention to increase children's selection of low-fat foods in school lunches. *Journal of Pediatrics, 125*(4), 535-540.

Wilbur, C. S., Zifferblatt, S. M., Pinsky, J. L., & Zifferblatt, S. (1981). Healthy vending: A cooperative pilot research program to stimulate good health in the marketplace. *Preventive Medicine, 10*(1), 85-93.

Wilkinson, C., & Room, R. (2009). Warnings on alcohol containers and advertisements: International experience and evidence on effects. *Drug and Alcohol Review, 28*(4), 426-435.

Willemsen, M. C. (2005). The new EU cigarette health warnings benefit smokers who want to quit the habit: Results from the Dutch Continuous survey of smoking habits. *European Journal of Public Health, 15*(4), 389-392.

Wisdom, J., Downs, J. S., & Loewenstein, G. (2010). Promoting healthy choices: Information versus convenience. *American Economic Journal: Applied Economics, 2*(2), 164-178.

Yamamoto, J. A., Yamamoto, J. B., Yamamoto, B. E., & Yamamoto, L. G. (2005). Adolescent fast food and restaurant ordering behavior with and without calorie and fat content menu information. *Journal of Adolescent Health, 37*(5), 397-402.

Yeomans, M. R., Lartamo, S., Procter, E. L., Lee, M. D., & Gray, R. W. (2001). The actual, but not labelled, fat content of a soup preload alters short-term appetite in healthy men. *Physiology & Behavior, 73*(4), 533-540.

PRESENTATION

Branen, L., Fletcher, J., & Hilbert, L. (2002). Snack consumption and waste by preschool children served "cute" versus regular snacks. *Journal of Nutrition Education and Behavior, 34*(5), 279-282.

Brondel, L., Romer, M., Van Wymelbeke, V., Pineau, N., Jiang, T., Hanus, C., & Rigaud, D. (2009). Variety enhances food intake in humans: Role of sensory-specific satiety. *Physiology & Behavior, 97*(1), 44-51.

Chandon, P., & Wansink, B. (2011). Is food marketing making us fat? A multi-disciplinary review *Faculty & Research Working Paper*. Fountainbleau, France: Instead: The Business School for the World.

Forman, J., Halford, J. C. G., Summe, H., MacDougall, M., & Keller, K. L. (2009). Food branding influences ad libitum intake differently in children depending on weight status. Results of a pilot study. *Appetite, 53*(1), 76-83.

Gosnell, B. A., Mitchell, J. E., Lancaster, K. L., Burgard, M. A., Wonderlich, S. A., & Crosby, R. D. (2001). Food presentation and energy intake in a feeding laboratory study of subjects with binge eating disorder. *International Journal of Eating Disorders, 30*(4), 441-446.

Guerrieri, R., Nederkoorn, C., & Jansen, A. (2007). How impulsiveness and variety influence food intake in a sample of healthy women. *Appetite, 48*(1), 119-122.

Guerrieri, R., Nederkoorn, C., & Jansen, A. (2008). The interaction between impulsivity and a varied food environment: Its influence on food intake and overweight. *International Journal of Obesity (London), 32*(4), 708-714.

Kahn, B. E., & Wansink, B. (2004). The influence of assortment structure on perceived variety and consumption quantities. *Journal of Consumer Research, 30*(4), 519-533.

Madzharov, A. V., & Block, L. G. (2010). Effects of product unit image on consumption of snack foods. *Journal of Consumer Psychology, 20*(4), 398-409.

Moodie, C., Mackintosh, A. M., Hastings, G., & Ford, A. (2011). Young adult smokers' perceptions of plain packaging: A pilot naturalistic study. *Tobacco Control, 20*(5), 367-373.

Moodie, C., Stead, M., Bauld, L., McNeill, A., Angus, K., Hinds, K., . . . O'Mara-Eves, A. (2012). Plain Tobacco Packaging: A Systematic Review *UK Centre for Tobacco Control Studies*. Stirling: University of Stirling and the Open University.

Norton, G. N., Anderson, A. S., & Hetherington, M. M. (2006). Volume and variety: Relative effects on food intake. *Physiology & Behavior, 87*(4), 714-722.

Rolls, B. J., Rowe, E. A., Rolls, E. T., Kingston, B., Megson, A., & Gunary, R. (1981). Variety in a meal enhances food intake in man. *Physiology & Behavior, 26*(2), 215-221.

Rolls, B. J., Rowe, E. A., & Rolls, E. T. (1982). How sensory properties of foods affect human feeding behavior. *Physiology & Behavior, 29*(3), 409-417.

Rolls, B. J. (1985). Experimental analyses of the effects of variety in a meal on human feeding. *American Journal of Clinical Nutrition, 42*(Suppl.5), 932-939.

Smith, J. M., & Ditschun, T. L. (2009). Controlling satiety: How environmental factors influence food intake. *Trends in Food Science & Technology, 20*(6-7), 271-277.

Sobal, J., & Wansink, B. (2007). Kitchenscapes, tablescapes, platescapes, and foodscapes. *Environment and Behavior, 39*(1), 124-142.

Sorensen, L. B., Moller, P., Flint, A., Martens, M., & Raben, A. (2003). Effect of sensory perception of foods on appetite and food intake: A review of studies on humans. *International Journal on Obesity and Related Metabolic Disorders, 27*(10), 1152-1166.

Stubbs, R. J., Johnstone, A. M., Mazlan, N., Mbaiwa, S. E., & Ferris, S. (2001). Effect of altering the variety of sensorially distinct foods, of the same macronutrient content, on food intake and body weight in men. *European Journal of Clinical Nutrition, 55*(1), 19-28.

Temple, J. L., Giacomelli, A. M., Roemmich, J. N., & Epstein, L. H. (2008). Dietary variety impairs habituation in children. *Health Psychology, 27*(Suppl.1), s10-s19.

van Dongen, M. V., De Graaf, C., Siebelink, E., & Kok, F. J. (2009). Hidden fat facilitates passive overconsumption. *Journal of Nutrition, 139*(2), 394-399.

Yeomans, M. R., Gould, N. J., Leitch, M., & Mobini, S. (2009). Effects of energy density and portion size on development of acquired flavour liking and learned satiety. *Appetite, 52*(2), 469-478.

PRIMING

Barwood, M. J., Weston, N. J. V., Thelwell, R., & Page, J. (2009). A motivational music and video intervention improves high-intensity exercise performance. *Journal of Sports Science and Medicine, 8*(3), 435-442.

Bell, R., Meiselman, H. L., Pierson, B. J., & Reeve, W. G. (1994). Effects of adding an Italian theme to a restaurant on the perceived ethnicity, acceptability, and selection of foods. *Appetite, 22*(1), 11-24.

Coelho, J. S., Polivy, J., Peter, H. C., & Pliner, P. (2009). Wake up and smell the cookies. Effects of olfactory food-cue exposure in restrained and unrestrained eaters. *Appetite, 52*(2), 517-520.

Cohen, D., & Farley, T. A. (2008). Eating as an automatic behavior. *Preventing Chronic Disease, 5*(1), A23.

Jacob, C. (2006). Styles of background music and consumption in a bar: An empirical evaluation. *International Journal of Hospitality Management, 25*(4), 716-720.

Jacob, C., Gueguen, N., & Boulbry, G. (2010). L'effet d'elements figuratifs sur le comportement de consommation: Une illustration de l'influence du choix d'un plat dans un restaurant [The effect of incidental information on consumption behaviour: An Illustration with the choice of a meal in a restaurant. With English summary.]. *La Revue des Sciences de Gestion, 45*(242), 61-67.

Papies, E. K., & Hamstra, P. (2010). Goal priming and eating behavior: Enhancing self-regulation by environmental cues. *Health Psychology, 29*(4), 384-388.

Polivy, J., Herman, C. P., Hackett, R., & Kuleshnyk, I. (1986). The effects of self-attention and public attention on eating in restrained and unrestrained subjects. *Journal of Personality and Social Psychology, 50*(6), 1253-1260.

Rohsenow, D. J., Monti, P. M., Colby, S. M., Gulliver, S. B., & et al. (1997). Effects of alcohol cues on smoking urges and topography among alcoholic men. *Alcoholism: Clinical and Experimental Research, 21*(1), 101-107.

Shimizu, M., Payne, C. R., & Wansink, B. (2010). When snacks become meals: How hunger and environmental cues bias food intake. *International Journal of Behavioral Nutrition and Physical Activity, 7*(63).

Spence, C., & Shankar, M. U. (2010). The influence of auditory cues on the perception of, and responses to, food and drink. *Journal of Sensory Studies, 25*(3), 406-430.

Zampini, M., & Spence, C. (2010). Assessing the role of sound in the perception of food and drink. *Chemosensory Perception, 3*(1), 57-67.

PROMPTING

Adams, J., & White, M. (2002). A systematic approach to the development and evaluation of an intervention promoting stair use. *Health Education Journal, 61*(3), 272-286.

Andersen, R. E., Franckowiak, S. C., Snyder, J., Bartlett, S. J., & Fontaine, K. R. (1998). Can inexpensive signs encourage the use of stairs? Results from a community intervention. *Annals of Internal Medicine, 129*(5), 363-369.

Andersen, R., Franckowiak, S., Zuzak, K., Cummings, E., Bartlett, S., & Crespo, C. (2006). Effects of a culturally sensitive sign on the use of stairs in African American commuters. *Sozial- und Präventivmedizin [Social and Preventive Medicine], 51*(6), 373-380.

Anderson, A. S., Porteous, L. E. G., Foster, E., Higgins, C., Stead, M., Hetherington, M., . . . Adamson, A. J. (2005). The impact of a school-based nutrition education intervention on dietary intake and cognitive and attitudinal variables relating to fruits and vegetables. *Public Health Nutrition, 8*(6), 650-656.

Areni, C. S., Duhan, D. F., & Kiecker, P. (1999). Point-of-purchase displays, product organization and brand purchase likelihoods. *Journal of the Academy of Marketing Science, 27*(4), 428-441.

Badland, H. M., & Schofield, G. M. (2005). Posters in a sample of professional worksites have no effect on objectively measured physical activity. *Health Promotion Journal of Australia, 16*(1), 78-81.

Blake, H., Lee, S., Stanton, T., & Gorely, T. (2008). Workplace intervention to promote stair-use in an NHS setting. *International Journal of Workplace Health Management, 1*(3), 162-175.

Blamey, A., Mutrie, N., & Aitchison, T. (1995). Health promotion by encouraged use of stairs. *British Medical Journal, 311*(7000), 289-290.

Boen, F., Maurissen, K., & Opdenacker, J. (2010). A simple health sign increases stair use in a shopping mall and two train stations in Flanders, Belgium. *Health Promotion International, 25*(2), 183-191.

Boutelle, K. N., Jeffery, R. W., Murray, D. M., & Schmitz, M. K. (2001). Using signs, artwork, and music to promote stair use in a public building. *American Journal of Public Health, 91*(12), 2004-2006.

Breugelmans, E., & Campo, K. (2011). Effectiveness of in-store displays in a virtual store environment. *Journal of retailing, 87*(1), 75-89.

Buscher, L. A., Martin, K. A., & Crocker, S. (2001). Point-of-purchase messages framed in terms of cost, convenience, taste, and energy improve healthful snack selection in a college foodservice setting. *Journal of the American Dietetic Association, 101*(8), 909-913.

Cheung, P. P., Chow, B. C., & Parfitt, G. (2008). Using environmental stimuli in physical activity intervention for school teachers: A pilot study *International Electronic Journal of Health Education, 11*, 47-56.

Chevalier, M. (1975). Increase in sales due to in-store display. *Journal of Marketing Research, 12*(4), 426-431.

Cohen, D., & Farley, T. A. (2008). Eating as an automatic behavior. *Preventing Chronic Disease, 5*(1), A23.

Coleman, K. J., & Gonzalez, E. C. (2001). Promoting stair use in a US-Mexico border community. *American Journal of Public Health, 91*(12), 2007-2009.

Connell, D., Goldberg, J. P., & Folta, S. C. (2001). An intervention to increase fruit and vegetable consumption using audio communications: In-store public service announcements and audiotapes. *Journal of Health Communication, 6*(1), 31-43.

Cook, C., Simmons, G., Swinburn, B., & Stewart, J. (2001). Changing risk behaviours for non-communicable disease in New Zealand working men--is workplace intervention effective? *The New Zealand Medical Journal, 114*(1130), 175-178.

Dolan, M. S., Weiss, L. A., Lewis, R. A., Pietrobelli, A., Heo, M., & Faith, M. S. (2006). 'Take the stairs instead of the escalator': Effect of environmental prompts on community stair use and implications for a national 'Small Steps' campaign. *Obesity Reviews, 7*(1), 25-32.

Dunn, A. L., Andersen, R. E., & Jakicic, J. M. (1998). Lifestyle physical activity interventions. History, short- and long-term effects, and recommendations. *American Journal of Preventive Medicine, 15*(4), 398-412.

Engbers, L. H., van Poppel, M. N. M., Chin A Paw, M. J. M., & van Mechelen, W. (2005). Worksite health promotion programs with environmental changes: A systematic review. *American Journal of Preventive Medicine, 29*(1), 61-70.

Engbers, L. H., van Poppel, M. N., Chin, A. P. M., & van Mechelen, W. (2006). The effects of a controlled worksite environmental intervention on determinants of dietary behavior and self-reported fruit, vegetable and fat intake. *BMC Public Health, 6*, 253.

Eves, F. F., & Masters, R. S. (2006). An uphill struggle: Effects of a point-of-choice stair climbing intervention in a non-English speaking population. *International Journal of Epidemiology, 35*(5), 1286-1290.

Eves, F. F., & Webb, O. J. (2006). Worksite interventions to increase stair climbing: Reasons for caution. *Preventive Medicine, 43*(1), 4-7.

Eves, F. F., Webb, O. J., & Mutrie, N. (2006). A workplace intervention to promote stair climbing: Greater effects in the overweight. *Obesity (Silver Spring), 14*(12), 2210-2216.

Eves, F. F. (2008). All choices are not equal: Effects of context on point-of-choice prompts for stair climbing. *Obesity Reviews, 9*(1), 83-84; author reply 85-86.

Eves, F. F., Masters, R. S., & McManus, A. M. (2008). Effects of point-of-choice stair climbing interventions in Hong Kong. *Hong Kong Medical Journal, 14*(Suppl.5), 36-39.

Eves, F. F. (2010). Point-of-decision prompts to increase stair use. *American Journal of Preventive Medicine, 38*(5), 573-574.

Eves, F. F., Olander, E. K., Nicoll, G., Puig-Ribera, A., & Griffin, C. (2009). Increasing stair climbing in a train station: The effects of contextual variables and visibility. *Journal of Environmental Psychology, 29*(2), 300-303.

Eves, F. F., Olander, E. K., Webb, O. J., Griffin, C., & Chambers, J. (2012). Likening stairs in buildings to climbing a mountain: Self-reports of expected effects on stair climbing and objective measures of effectiveness. *Psychology of Sport and Exercise, 13*(2), 170-176.

Foster, C., & Hillsdon, M. (2004). Changing the environment to promote health-enhancing physical activity. *Journal of Sports Sciences, 22*(8), 755-769.

Foster, C., Hillsdon, M., Cavill, N., Bull, F., Buxton, K., & Crombie, H. (2006). Interventions that use the environment to encourage physical activity *Evidence review*. London, UK: National Institute for Health and Clinical Excellence.

Freedman, M. R., & Connors, R. (2010). Point-of-purchase nutrition information influences food-purchasing behaviors of college students: A pilot study. *Journal of the American Dietetic Association, 110*(8), 1222-1226.

French, S. A., Jeffery, R. W., Story, M., Breitlow, K. K., Baxter, J. S., Hannan, P., & Snyder, M. P. (2001). Pricing and promotion effects on low-fat vending snack purchases: The CHIPS Study. *American Journal of Public Health, 91*(1), 112-127.

Glanz, K., & Mullis, R. M. (1988). Environmental interventions to promote healthy eating: A review of models, programs, and evidence. *Health Education & Behavior, 15*(4), 395-415.

Glanz, K., & Hoelscher, D. (2004). Increasing fruit and vegetable intake by changing environments, policy and pricing: Restaurant-based research, strategies, and recommendations. *Preventive Medicine, 39* (Suppl.2), s88-s93.

Goldberg, M. E., & Gunasti, K. (2007). Creating an environment in which youths are encouraged to eat a healthier diet. *Journal of Public Policy & Marketing, 26*(2), 162-181.

Greco, A. J., & Johnson, E. B. (1997). Supermarket shoppers' response to mature models in point-of-purchase displays. *Journal of Food Products Marketing, 4*(3), 9-23.

Horgen, K. B., & Brownell, K. D. (2002). Comparison of price change and health message interventions in promoting healthy food choices. *Health Psychology, 21*(5), 505-512.

Iversen, M. K., Handel, M. N., Jensen, E. N., Frederiksen, P., & Heitmann, B. L. (2007). Effect of health-promoting posters placed on the platforms of two train stations in Copenhagen, Denmark, on the choice between taking the stairs or the escalators: A secondary publication. *International Journal of Obesity, 31*(6), 950-955.

Jenum, A. K., Lorentzen, C. A. N., & Ommundsen, Y. (2009). Targeting physical activity in a low socioeconomic status population: Observations from the Norwegian "Romsas in Motion" study. *British Journal of Sports Medicine, 43*(1), 64-69.

Kahn, E. B., Ramsey, L. T., Brownson, R. C., Heath, G. W., Howze, E. H., Powell, K. E., . . . Corso, P. (2002). The effectiveness of interventions to increase physical activity. A systematic review. *American Journal of Preventive Medicine, 22*(Suppl.4), 73-107.

Kerr, J., Eves, F., & Carroll, D. (2000). Posters can prompt less active people to use the stairs. *Journal of Epidemiology & Community Health, 54*(12), 942.

Kerr, J., Eves, F., & Carroll, D. (2001). Encouraging stair use: Stair-riser banners are better than posters. *American Journal of Public Health, 91*(8), 1192-1193.

Kerr, J., Eves, F., & Carroll, D. (2001). Six-month observational study of prompted stair climbing. *Preventive Medicine, 33*(5), 422-427.

Kerr, J., Eves, F. F., & Carroll, D. (2001). The influence of poster prompts on stair use: The effects of setting, poster size and content. *British Journal of Health Psychology, 6*(Pt.4), 397-405.

Kerr, N. A., Yore, M. M., Ham, S. A., & Dietz, W. H. (2004). Increasing stair use in a worksite through environmental changes. *American Journal of Health Promotion, 18*(4), 312-315.

Kwak, L., Kremers, S. P., van Baak, M. A., & Brug, J. (2007). A poster-based intervention to promote stair use in blue- and white-collar worksites. *Preventive Medicine, 45*(2-3), 177-1781.

Lewis, A., & Eves, F. (2011). Specific effects of a calorie-based intervention on stair climbing in overweight commuters. *Annals of Behavioral Medicine, 42*(2), 257-261.

Lewis, A. L., & Eves, F. F. (2012). Prompts to increase stair climbing in stations: The effect of message complexity. *Journal of Physical Activity & Health, In Press.*

Lewis, A. L., & Eves, F. F. (2012). Testing the theory underlying the success of point-of-choice prompts: A multi-component stair climbing intervention. *Psychology of Sport and Exercise, 13*(2), 126-132.

Marshall, A. L., Bauman, A. E., Patch, C., Wilson, J., & Chen, J. (2002). Can motivational signs prompt increases in incidental physical activity in an Australian health-care facility? *Health Education Research, 17*(6), 743-749.

Mayer, J. A., Heins, J. M., Vogel, J. M., Morrison, D. C., Lankester, L. D., & Jacobs, A. L. (1986). Promoting low-fat entree choices in a public cafeteria. *Journal of Applied Behavior Analysis, 19*(4), 397-402.

Mayer, J. A., Dubbert, P. M., & Elder, J. P. (1989). Promoting nutrition at the point of choice: A review. *Health Education Quarterly, 16*(1), 31-43.

Meyer, P., Kayser, B., Kossovsky, M. P., Sigaud, P., Carballo, D., Keller, P. F., . . . Mach, F. (2010). Stairs instead of elevators at workplace: Cardioprotective effects of a pragmatic intervention. *European Journal of Cardiovascular Prevention & Rehabilitation, 17*(5), 569-575.

Muller-Riemenschneider, F., Nocon, M., Reinhold, T., & Willich, S. N. (2010). Promotion of physical activity using point-of-decision prompts in Berlin underground stations. *International Journal of Environmental Research and Public Health, 7*(8), 3063-3070.

Nocon, M., Muller-Riemenschneider, F., Nitzschke, K., & Willich, S. N. (2010). Review Article: Increasing physical activity with point-of-choice prompts--a systematic review. *Scandinavian Journal of Public Health, 38*(6), 633-638.

Nomura, T., Yoshimoto, Y., Akezaki, Y., & Sato, A. (2009). Changing behavioral patterns to promote physical activity with motivational signs. *Environmental Health and Preventive Medicine, 14*(1), 20-25.

Ogawa, Y., Tanabe, N., Honda, A., Azuma, T., Seki, N., Suzuki, T., & Suzuki, H. (2011). Point-of-purchase health information encourages customers to purchase vegetables: Objective analysis by using a point-of-sales system. *Environmental Health and Preventive Medicine, 16*(4), 239-246.

Olander, E. K., Eves, F. F., & Puig-Ribera, A. (2008). Promoting stair climbing: Stair-riser banners are better than posters… sometimes. *Preventive Medicine, 46*(4), 308-310.

Olander, E. K., & Eves, F. F. (2011). Elevator availability and its impact on stair use in a workplace. *Journal of Environmental Psychology, 31*(2), 200-206.

Olander, E. K., & Eves, F. F. (2011). Effectiveness and cost of two stair-climbing interventions—less is more. *American Journal of Health Promotion, 25*(4), 231-236.

Pillay, J. D., Kolbe-Alexander, T., Achmat, M., Carstene, M., & Lambert, E. V. (2009). Are point-of-decision prompts in a sports science and medicine centre effective in changing the prevalence of stair usage? A preliminary study. *South African Journal of Sports Medicine, 21*(2).

Puig-Ribera, A., & Eves, F. F. (2010). Promoting stair climbing in Barcelona: Similarities and differences with interventions in English-speaking populations. *European Journal of Public Health, 20*(1), 100-102.

Rogers, Y., Hazlewood, W. R., Marshall, P., Dalton, N., & Hetrich, S. (2010). Ambient influence: Can twinkly lights lure and abstract representations trigger behavioural change? *UbiComp 2010, 26-29*, 261-270.

Sallis, J. F., Bauman, A., & Pratt, M. (1998). Environmental and policy interventions to promote physical activity. *American Journal of Preventive Medicine, 15*(4), 379-397.

Seymour, J. D., Yaroch, A. L., Serdula, M., Blanck, H. M., & Khan, L. K. (2004). Impact of nutrition environmental interventions on point-of-purchase behavior in adults: A review. *Preventive Medicine, 39* (Suppl.2), s108-s136.

Shepherd, J., Harden, A., Rees, R., Brunton, G., Garcia, J., Oliver, S., & Oakley, A. (2006). Young people and healthy eating: A systematic review of research on barriers and facilitators. *Health Education Research, 21*(2), 239-257.

Soler, R. E., Leeks, K. D., Buchanan, L. R., Brownson, R. C., Heath, G. W., & Hopkins, D. H. (2010). Point-of-decision prompts to increase stair use. A systematic review update. *American Journal of Preventive Medicine, 38*(Suppl.2), s292-s300.

Sorensen, L. B., Moller, P., Flint, A., Martens, M., & Raben, A. (2003). Effect of sensory perception of foods on appetite and food intake: A review of studies on humans. *International Journal on Obesity and Related Metabolic Disorders, 27*(10), 1152-1166.

Sorensen, G., Linnan, L., & Hunt, M. K. (2004). Worksite-based research and initiatives to increase fruit and vegetable consumption. *Preventive Medicine, 39* (Suppl.2), s94-s100.

van Houten, R. V., Nau, P. A., & Merrigan, M. (1981). Reducing elevator energy use: A comparison of posted feedback and reduced elevator convenience. *Journal of Applied Behavior Analysis, 14*(4), 377-387.

van Nieuw-Amerongen, M. E., Kremers, S. P. J., de Vries, N. K., & Kok, G. (2011). The use of prompts, increased accessibility, visibility, and aesthetics of the stairwell to promote stair use in a university building. *Environment and Behavior, 43*(1), 131-139.

Wagner, J. L., & Winett, R. A. (1988). Prompting one low-fat, high-fiber selection in a fast-food restaurant. *Journal of Applied Behavior Analysis, 21*(2), 179-185.

Wakefield, M., Germain, D., & Henriksen, L. (2008). The effect of retail cigarette pack displays on impulse purchase. *Addiction, 103*(2), 322-328.

Wansink, B., Kent, R. J., & Hoch, S. J. (1998). An anchoring and adjustment model of purchase quantity decisions. *Journal of Marketing Research, 35*(1), 71-81.

Webb, O. J., & Eves, F. F. (2007). Effects of environmental changes in a stair climbing intervention: generalization to stair descent. *American Journal of Health Promotion, 22*(1), 38-44.

Webb, O. J., & Cheng, T.-F. (2010). An informational stair climbing intervention with greater effects in overweight pedestrians. *Health Education Research, 25*(6), 936-944.

Webb, O. J., Eves, F. F., & Kerr, J. (2011). A statistical summary of mall-based stair-climbing interventions. *Journal of Physical Activity & Health, 8*(4), 558-565.

Wilbur, C. S., Zifferblatt, S. M., Pinsky, J. L., & Zifferblatt, S. (1981). Healthy vending: A cooperative pilot research program to stimulate good health in the marketplace. *Preventive Medicine, 10*(1), 85-93.

Winett, R. A., Moore, J. F., Wagner, J. L., Hite, L. A., Leahy, M., Neubauer, T. E., . . . Mundy, L. (1991). Altering shoppers' supermarket purchases to fit nutritional guidelines: An interactive information system. *Journal of Applied Behavior Analysis, 24*(1), 95-105.

Wu, S., Cohen, D., Shi, Y., Pearson, M., & Sturm, R. (2011). Economic analysis of physical activity interventions. *American Journal of Preventive Medicine, 40*(2), 149-158.

PROXIMITY

Chandon, P., & Wansink, B. (2011). Is food marketing making us fat? A multi-disciplinary review *Faculty & Research Working Paper*. Fountainbleau, France: Instead: The Business School for the World.

Cohen, D., & Farley, T. A. (2008). Eating as an automatic behavior. *Preventing Chronic Disease, 5*(1), A23.

Dayan, E., & Bar-Hillel, M. (2011). Nudge to nobesity II: Menu positions influence food orders. *Judgment and Decision Making, 6*(4), 333-342.

Engell, D., Kramer, M., Malafi, T., Salomon, M., & Lesher, L. (1996). Effects of effort and social modeling on drinking in humans. *Appetite, 26*(2), 129-138.

Glanz, K., & Hoelscher, D. (2004). Increasing fruit and vegetable intake by changing environments, policy and pricing: Restaurant-based research, strategies, and recommendations. *Preventive Medicine, 39* (Suppl.2), s88-s93.

Huang, A., Barzi, F., Huxley, R., Denyer, G., Rohrlach, B., Jayne, K., & Neal, B. (2006). The effects on saturated fat purchases of providing internet shoppers with purchase- specific dietary advice: A randomised trial. *PLoS Clinical Trials, 1*(5), e22.

Just, D., & Wansink, B. (2009). Smarter lunchrooms: Using behavioral economics to improve meal selection. *Choices, 29*(3).

Kincaid, C. S., & Corsun, D. L. (2003). Are consultants blowing smoke? An empirical test of the impact of menu layout on item sales. *International Journal of Contemporary Hospitality Management, 15*(4).

Lombardini-Riipinen, C., & Lankoski, L. (2010). *Take off the heater: Utility effect and food environment effect in food consumption decisions.* Paper presented at the 1st Joint EAAE/AAEA Seminar, Munich, Germany.

Maas, J., de Ridder, D. T., de Vet, E., & de Wit, J. B. (2011). Do distant foods increase intake? The effect of food accessibility on consumption. *Psychology & Health*.

Meiselman, H. L., Hedderley, D., Staddon, S. L., Pierson, B. J., & Symonds, C. R. (1994). Effect of effort on meal selection and meal acceptability in a student cafeteria. *Appetite, 23*(1), 43-55.

Meyers, A. W., Stunkard, A. J., & Coll, M. (1980). Food accessibility and food choice. A test of Schachter's externality hypothesis. *Archives of General Psychiatry, 37*(10), 1133-1135.

Musher-Eizenman, D. R., Young, K. M., Laurene, K., Galliger, C., Hauser, J., & Wagner, O. M. (2010). Children's sensitivity to external food cues: How distance to serving bowl influences children's consumption. *Health Education & Behavior, 37*(2), 186-192.

Painter, J. E., Wansink, B., & Hieggelke, J. B. (2002). How visibility and convenience influence candy consumption. *Appetite, 38*(3), 237-238.

Rozin, P., Scott, S., Dingley, M., Urbanek, J. K., Jiang, H., & Kaltenbach, M. (2011). Nudge to nobesity I: Minor changes in accessibility decrease food intake. *Judgment and Decision Making, 6*(4), 323-332.

Sigurdsson, V., Larsen, N. M., & Gunnarsson, D. (2011). An in-store experimental analysis of consumers' selection of fruits and vegetables. *The Service Industries Journal, 31*(15), 2587-2602.

Sobal, J., & Wansink, B. (2007). Kitchenscapes, tablescapes, platescapes, and foodscapes. *Environment and Behavior, 39*(1), 124-142.

Thorndike, A. N., Sonnenberg, L., Riis, J., Barraclough, S., & Levy, D. E. (2012). A 2-Phase labeling and choice architecture intervention to improve healthy food and beverage choices. *American Journal of Public Health, 102*(3), 527-533.

Wansink, B. (2004). Environmental factors that increase the food intake and consumption volume of unknowing consumers. *Annual Review of Nutrition, 24*, 455-479.

Wansink, B., Painter, J. E., & Lee, Y. K. (2006). The office candy dish: Proximity's influence on estimated and actual consumption. *International Journal of Obesity, 30*(5), 871-875.

Wisdom, J., Downs, J. S., & Loewenstein, G. (2010). Promoting healthy choices: Information versus convenience. *American Economic Journal: Applied Economics, 2*(2), 164-178.

Zimring, C., Joseph, A., Nicoll, G. L., & Tsepas, S. (2005). Influences of building design and site design on physical activity: Research and intervention opportunities. *American Journal of Preventive Medicine, 28*(2 Suppl.2), 186-193.

SIZING

Burger, K. S., Fisher, J. O., & Johnson, S. L. (2011). Mechanisms behind the portion size effect: Visibility and bite size. *Obesity, 19*(3), 546-551.

Chait, L. D., & Griffiths, R. R. (1982). Smoking behavior and tobacco smoke intake: Response of smokers to shortened cigarettes. *Clinical Pharmacology and Therapeutics, 32*(1), 90-97.

Chandon, P., & Wansink, B. (2011). Is food marketing making us fat? A multi-disciplinary review *Faculty & Research Working Paper*. Fountainbleau, France: Instead: The Business School for the World.

Cohen, D., & Farley, T. A. (2008). Eating as an automatic behavior. *Preventing Chronic Disease, 5*(1), A23.

DeGraffe, C., & Hulshof, T. (1996). Effects of weight and energy content of preloads on subsequent appetite and food intake. *Appetite, 26*(2), 139-151.

Devitt, A. A., & Mattes, R. D. (2004). Effects of food unit size and energy density on intake in humans. *Appetite, 42*(2), 213-220.

Diliberti, N., Bordi, P. L., Conklin, M. T., Roe, L. S., & Rolls, B. J. (2004). Increased portion size leads to increased energy intake in a restaurant meal. *Obesity, 12*(3), 562-568.

Ebbeling, C. B., Garcia-Lago, E., Leidig, M. M., Seger-Shippee, L. G., Feldman, H. A., & Ludwig, D. S. (2007). Altering portion sizes and eating rate to attenuate gorging during a fast food meal: Effects on energy intake. *Pediatrics, 119*(5), 869-875.

Ello-Martin, J. A., Ledikwe, J. H., & Rolls, B. J. (2005). The influence of food portion size and energy density on energy intake: Implications for weight management. *American Journal of Clinical Nutrition, 82*(Suppl.1), s236-s241.

Fisher, J. O., Rolls, B. J., & Birch, L. L. (2003). Children's bite size and intake of an entrée are greater with large portions than with age-appropriate or self-selected portions. *The American Journal of Clinical Nutrition, 77*(5), 1164-1170.

Fisher, J. O., Liu, Y., Birch, L. L., & Rolls, B. J. (2007). Effects of portion size and energy density on young children's intake at a meal. *American Journal of Clinical Nutrition, 86*(1), 174-179.

Fisher, J. O., Arreola, A., Birch, L. L., & Rolls, B. J. (2007). Portion size effects on daily energy intake in low-income Hispanic and African American children and their mothers. *The American Journal of Clinical Nutrition, 86*(6), 1709-1716.

Fisher, J. O., & Kral, T. V. (2008). Super-size me: Portion size effects on young children's eating. *Physiology & Behavior, 94*(1), 39-47.

Flood, J. E., Roe, L. S., & Rolls, B. J. (2006). The effect of increased beverage portion size on energy intake at a meal. *Journal of the American Dietetic Association, 106*(12), 1984-1990.

Freedman, M. R., & Brochado, C. (2010). Reducing portion size reduces food intake and plate waste. *Obesity, 18*(9), 1864-1866.

Geier, A. B., Rozin, P., & Doros, G. (2006). Unit bias. A new heuristic that helps explain the effect of portion size on food intake. *Psychological Science, 17*(6), 521-525.

Gosnell, B. A., Mitchell, J. E., Lancaster, K. L., Burgard, M. A., Wonderlich, S. A., & Crosby, R. D. (2001). Food presentation and energy intake in a feeding laboratory study of subjects with binge eating disorder. *International Journal of Eating Disorders, 30*(4), 441-446.

Hartstein, J., Cullen, K. W., Reynolds, K. D., Harrell, J., Resnicow, K., & Kennel, P. (2008). Impact of portion-size control for school a la carte items: Changes in kilocalories and macronutrients purchased by middle school students. *Journal of the American Dietetic Association, 108*(1), 140-144.

Jeffery, R. W., Rydell, S., Dunn, C. L., Harnack, L. J., Levine, A. S., Pentel, P. R., . . . Walsh, E. M. (2007). Effects of portion size on chronic energy intake. *International Journal of Behavioral Nutrition and Physical Activity, 4*, 27.

Kelly, M. T., Wallace, J. M., Robson, P. J., Rennie, K. L., Welch, R. W., Hannon-Fletcher, M. P., . . . Livingstone, M. B. (2009). Increased portion size leads to a sustained increase in energy intake over 4d in normal-weight and overweight men and women. *British Journal of Nutrition, 102*(3), 470-477.

Kral, T. V. E., Roe, L. S., & Rolls, B. J. (2004). Combined effects of energy density and portion size on energy intake in women. *The American Journal of Clinical Nutrition, 79*(6), 962-968.

Kral, T. V., & Rolls, B. J. (2004). Energy density and portion size: Their independent and combined effects on energy intake. *Physiology & Behavior, 82*(1), 131-138.

Kral, T. V. E. (2006). Effects on hunger and satiety, perceived portion size and pleasantness of taste of varying the portion size of foods: A brief review of selected studies. *Appetite, 46*(1), 103-105.

Kral, T. V., Kabay, A. C., Roe, L. S., & Rolls, B. J. (2010). Effects of doubling the portion size of fruit and vegetable side dishes on children's intake at a meal. *Obesity (Silver Spring), 18*(3), 521-527.

Leahy, K. E., Birch, L. L., Fisher, J. O., & Rolls, B. J. (2008). Reductions in entree energy density increase children's vegetable intake and reduce energy intake. *Obesity (Silver Spring), 16*(7), 1559-1565.

Ledikwe, J. H., Ello-Martin, J. A., & Rolls, B. J. (2005). Portion sizes and the obesity epidemic. *Journal of Nutrition, 135*(4), 905-909.

Levitsky, D. A., & Youn, T. (2004). The more food young adults are served, the more they overeat. *Journal of Nutrition, 134*(10), 2546-2549.

Levitsky, D. A. (2005). The non-regulation of food intake in humans: Hope for reversing the epidemic of obesity. *Physiology & Behavior, 86*(5), 623-632.

Lombardini-Riipinen, C., & Lankoski, L. (2010). *Take off the heater: Utility effect and food environment effect in food consumption decisions.* Paper presented at the 1st Joint EAAE/AAEA Seminar, Munich, Germany.

Looney, S. M., & Raynor, H. A. (2011). Impact of portion size and energy density on snack intake in preschool-aged children. *Journal of the American Dietetic Association, 111*(3), 414-418.

Marchiori, D., Waroquier, L., & Klein, O. (2011). Smaller food item sizes of snack foods influence reduced portions and caloric intake in young adults. *Journal of the American Dietetic Association, 111*(5), 727-731.

Marchiori, D., Corneille, O., & Klein, O. (2012). Container size influences snack food intake independently of portion size. *Appetite, 58*(3), 814-817.

Mathias, K. C., Rolls, B. J., Birch, L. L., Kral, T. V. E., Hanna, E. L., Davey, A., & Fisher, J. O. (2011). Serving larger portions of fruits and vegetables together at dinner promotes intake of both foods among young children. *Journal of the American Dietetic Association, 20*(10), 1-5.

Mendoza, J. A., Watson, K., & Cullen, K. W. (2010). Change in dietary energy density after implementation of the Texas Public School Nutrition Policy. *Journal of the American Dietetic Association, 110*(3), 434-440.

Raynor, H. A., & Wing, R. R. (2007). Package unit size and amount of food: Do both influence intake? *Obesity, 15*(9), 2311-2319.

Raynor, H. A., Van Walleghen, E. L., Niemeier, H., Butryn, M. L., & Wing, R. R. (2009). Do food provisions packaged in single-servings reduce energy intake at breakfast during a brief behavioral weight-loss intervention? *Journal of the American Dietetic Association, 109*(11), 1922-1925.

Rolls, B. J., Castellanos, V. H., Halford, J. C., Kilara, A., Panyam, D., Pelkman, C. L., . . . Thorwart, M. L. (1998). Volume of food consumed affects satiety in men. *American Journal of Clinical Nutrition, 67*(6), 1170-1177.

Rolls, B. J., Bell, E. A., & Waugh, B. A. (2000). Increasing the volume of a food by incorporating air affects satiety in men. *American Journal of Clinical Nutrition, 72*(2), 361-368.

Rolls, B. J., Engell, D., & Birch, L. L. (2000). Serving portion size influences 5-year-old but not 3-year-old children's food intakes. *Journal of the American Dietetic Association, 100*(2), 232-234.

Rolls, B. J., Morris, E. L., & Roe, L. S. (2002). Portion size of food affects energy intake in normal-weight and overweight men and women. *The American Journal of Clinical Nutrition, 76*(6), 1207-1213.

Rolls, B. J., Roe, L. S., & Meengs, J. S. (2004). Salad and satiety: Energy density and portion size of a first-course salad affect energy intake at lunch. *Journal of the American Dietetic Association, 104*(10), 1570-1576.

Rolls, B. J., Roe, L. S., Kral, T. V., Meengs, J. S., & Wall, D. E. (2004). Increasing the portion size of a packaged snack increases energy intake in men and women. *Appetite, 42*(1), 63-69.

Rolls, B. J., Roe, L. S., Meengs, J. S., & Wall, D. E. (2004). Increasing the portion size of a sandwich increases energy intake. *Journal of the American Dietetic Association, 104*(3), 367-372.

Rolls, B. J., Roe, L. S., & Meengs, J. S. (2006). Larger portion sizes lead to a sustained increase in energy intake over 2 days. *Journal of the American Dietetic Association, 106*(4), 543-549.

Rolls, B. J., Roe, L. S., & Meengs, J. S. (2006). Reductions in portion size and energy density of foods are additive and lead to sustained decreases in energy intake. *American Journal of Clinical Nutrition, 83*(1), 11-17.

Rolls, B. J., Roe, L. S., & Meengs, J. S. (2007). The effect of large portion sizes on energy intake is sustained for 11 days. *Obesity (Silver Spring), 15*(6), 1535-1543.

Rolls, B. J. (2010). Dietary strategies for the prevention and treatment of obesity. *Proceedings of the Nutrition Society, 69*(1), 70-79.

Rolls, B. J., Roe, L. S., & Meengs, J. S. (2010). Portion size can be used strategically to increase vegetable consumption in adults. *American Journal of Clinical Nutrition, 91*(4), 913-922.

Scott, M. L., Nowlis, S. M., Mandel, N., & Morales, A. C. (2008). The effects of reduced food size and package size on the consumption behavior of restrained and unrestrained eaters. *Journal of Consumer Research, 35*(3), 391-405.

Shepherd, J., Harden, A., Rees, R., Brunton, G., Garcia, J., Oliver, S., & Oakley, A. (2006). Young people and healthy eating: A systematic review of research on barriers and facilitators. *Health Education Research, 21*(2), 239-257.

Smith, J. M., & Ditschun, T. L. (2009). Controlling satiety: How environmental factors influence food intake. *Trends in Food Science & Technology, 20*(6-7), 271-277.

Sobal, J., & Wansink, B. (2007). Kitchenscapes, tablescapes, platescapes, and foodscapes. *Environment and Behavior, 39*(1), 124-142.

Spiegel, T. A., Kaplan, J. M., Tomassini, A., & Stellar, E. (1993). Bite size, ingestion rate, and meal size in lean and obese women. *Appetite, 21*(2), 131-145.

Spill, M. K., Birch, L. L., Roe, L. S., & Rolls, B. J. (2010). Eating vegetables first: The use of portion size to increase vegetable intake in preschool children. *The American Journal of Clinical Nutrition, 91*(5), 1237-1243.

Spill, M. K., Birch, L. L., Roe, L. S., & Rolls, B. J. (2011). Serving large portions of vegetable soup at the start of a meal affected children's energy and vegetable intake. *Appetite, 57*(1), 213-219.

Steenhuis, I. H., & Vermeer, W. M. (2009). Portion size: Review and framework for interventions. *The International Journal of Behavioral Nutrition and Physical Activity, 6*, 58.

Stroebele, N., Ogden, L. G., & Hill, J. O. (2009). Do calorie-controlled portion sizes of snacks reduce energy intake? *Appetite, 52*(3), 793-796.

Wansink, B. (1996). Can package size accelerate usage volume? *The Journal of Marketing, 60*(3), 1-14.

Wansink, B., & Park, S. B. (2001). At the movies: How external cues and perceived taste impact consumption volume. *Food Quality and Preference, 12*(1), 69-74.

Wansink, B. (2004). Environmental factors that increase the food intake and consumption volume of unknowing consumers. *Annual Review of Nutrition, 24*, 455-479.

Wansink, B., & Kim, J. (2005). Bad popcorn in big buckets: Portion size can influence intake as much as taste. *Journal of Nutrition Education and Behavior, 37*(5), 242-245.

Wansink, B., Painter, J. E., & North, J. (2005). Bottomless bowls: Why visual cues of portion size may influence intake. *Obesity Research, 13*(1), 93-100.

Wansink, B., & van Ittersum, K. (2007). Portion size me: Downsizing our consumption norms. *Journal of the American Dietetic Association, 107*(7), 1103-1106.

Wansink, B. (2010). From mindless eating to mindlessly eating better. *Physiology & Behavior, 100*(5), 454-463.

Weijzen, P. L. G., Liem, D. G., Zandstra, E. H., & de Graaf, C. (2008). Sensory specific satiety and intake: The difference between nibble- and bar-size snacks. *Appetite, 50*(2-3), 435-442.

Yeomans, M. R., Gould, N. J., Leitch, M., & Mobini, S. (2009). Effects of energy density and portion size on development of acquired flavour liking and learned satiety. *Appetite, 52*(2), 469-478.

Included articles by behaviour

DIET

Ahn, H. J., Han, K. A., Kwon, H. R., & Min, K. W. (2010). The small rice bowl-based meal plan was effective at reducing dietary energy intake, body weight, and blood glucose levels in Korean women with type 2 diabetes mellitus. *Korean Diabetes Journal, 34*(6), 340-349.

Anderson, A. S., Porteous, L. E. G., Foster, E., Higgins, C., Stead, M., Hetherington, M., . . . Adamson, A. J. (2005). The impact of a school-based nutrition education intervention on dietary intake and cognitive and attitudinal variables relating to fruits and vegetables. *Public Health Nutrition, 8*(6), 650-656.

Andrade, A. M., Greene, G. W., & Melanson, K. J. (2008). Eating slowly led to decreases in energy intake within meals in healthy women. *Journal of the American Dietetic Association, 108*(7), 1186-1191.

Angelopoulos, P. D., Milionis, H. J., Grammatikaki, E., Moschonis, G., & Manios, Y. (2009). Changes in BMI and blood pressure after a school based intervention: The CHILDREN study. *European Journal of Public Health, 19*(3), 319-325.

Antonuk, B., & Block, L. G. (2006). The effect of single serving versus entire package nutritional information on consumption norms and actual consumption of a snack food. *Journal of Nutrition Education and Behavior, 38*(6), 365-370.

Aron, J. I., Evans, R. E., & Mela, D. J. (1995). Paradoxical effect of a nutrition labelling scheme in a student cafeteria. *Nutrition Research, 15*(9), 1251-1261.

Bartholomew, J. B., & Jowers, E. M. (2006). Increasing frequency of lower-fat entrees offered at school lunch: An environmental change strategy to increase healthful selections. *Journal of the American Dietetic Association, 106*(2), 248-252.

Bassett, M. T., Dumanovsky, T., Huang, C., Silver, L. D., Young, C., Nonas, C., . . . Frieden, T. R. (2008). Purchasing behavior and calorie information at fast-food chains in New York City, 2007. *American Journal of Public Health, 98*(8), 1457-1459.

Bell, R., Meiselman, H. L., Pierson, B. J., & Reeve, W. G. (1994). Effects of adding an Italian theme to a restaurant on the perceived ethnicity, acceptability, and selection of foods. *Appetite, 22*(1), 11-24.

Bellisle, F., & Dalix, A.-M. (2001). Cognitive restraint can be offset by distraction, leading to increased meal intake in women. *The American Journal of Clinical Nutrition, 74*(2), 197-200.

Bellisle, F., Dalix, A. M., & Slama, G. (2004). Non food-related environmental stimuli induce increased meal intake in healthy women: Comparison of television viewing versus listening to a recorded story in laboratory settings. *Appetite, 43*(2), 175-180.

Bellisle, F., Dalix, A.-M., Airinei, G., Hercberg, S., & Péneau, S. (2009). Influence of dietary restraint and environmental factors on meal size in normal-weight women. A laboratory study. *Appetite, 53*(3), 309-313.

Bergen, D., & Yeh, M. C. (2006). Effects of energy-content labels and motivational posters on sales of sugar-sweetened beverages: Stimulating sales of diet drinks among adults study. *Journal of the American Dietetic Association, 106*(11), 1866-1869.

Blass, E. M., Anderson, D. R., Kirkorian, H. L., Pempek, T. A., Price, I., & Koleini, M. F. (2006). On the road to obesity: Television viewing increases intake of high-density foods. *Physiology & Behavior, 88*(4-5), 597-604.

Bohnert, A. M., Randall, E. T., Tharp, S., & Germann, J. (2011). The development and evaluation of a portion plate for youth: A pilot study. *Journal of Nutrition Education & Behavior, 43*(4), 268-273.

Bollinger, B., Leslie, P., & Sorensen, A. (2011). Calorie posting in chain restaurants. *American Economic Journal: Economic Policy, 3*(1), 91-128.

Borgmeier, I., & Westenhoefer, J. (2009). Impact of different food label formats on healthiness evaluation and food choice of consumers: A randomized-controlled study. *BMC Public Health, 9*, 184.

Branen, L., Fletcher, J., & Hilbert, L. (2002). Snack consumption and waste by preschool children served "cute" versus regular snacks. *Journal of Nutrition Education and Behavior, 34*(5), 279-282.

Breugelmans, E., & Campo, K. (2011). Effectiveness of in-store displays in a virtual store environment. *Journal of retailing, 87*(1), 75-89.

Brondel, L., Romer, M., Van Wymelbeke, V., Pineau, N., Jiang, T., Hanus, C., & Rigaud, D. (2009). Variety enhances food intake in humans: Role of sensory-specific satiety. *Physiology & Behavior, 97*(1), 44-51.

Burger, K. S., Fisher, J. O., & Johnson, S. L. (2011). Mechanisms behind the portion size effect: Visibility and bite size. *Obesity, 19*(3), 546-551.

Burton, S., Creyer, E. H., Kees, J., & Huggins, K. (2006). Attacking the obesity epidemic: The potential health benefits of providing nutrition information in restaurants. *American Journal of Public Health, 96*(9), 1669-1675.

Buscher, L. A., Martin, K. A., & Crocker, S. (2001). Point-of-purchase messages framed in terms of cost, convenience, taste, and energy improve healthful snack selection in a college foodservice setting. *Journal of the American Dietetic Association, 101*(8), 909-913.

Caldwell, C., & Hibbert, S. A. (1999). Play that one again: The effect of music tempo on consumer behaviour in a restaurant *European Advances in Consumer Research, 4*, 58-62.

Caldwell, C., & Hibbert, S. A. (2002). The influence of music tempo and musical preference on restaurant patrons' behavior. *Psychology and Marketing, 19*(11), 895-917.

Chandon, P., & Wansink, B. (2002). When are stockpiled products consumed faster? A convenience-salience framework of postpurchase consumption incidence and quantity. *Journal of Marketing Research, 39*(3), 321-335.

Chandon, P., & Wansink, B. (2011). Is food marketing making us fat? A multi-disciplinary review *Faculty & Research Working Paper*. Fountainbleau, France: Instead: The Business School for the World.

Chevalier, M. (1975). Increase in sales due to in-store display. *Journal of Marketing Research, 12*(4), 426-431.

Chu, Y. H., Frongillo, E. A., Jones, S. J., & Kaye, G. L. (2009). Improving patrons' meal selections through the use of point-of-selection nutrition labels. *American Journal of Public Health, 99*(11), 2001-2005.

Cinciripini, P. M. (1984). Changing food selections in a public cafeteria. *Behavior Modification, 8*(4), 520-539.

Coelho, J. S., Polivy, J., Peter, H. C., & Pliner, P. (2009). Wake up and smell the cookies. Effects of olfactory food-cue exposure in restrained and unrestrained eaters. *Appetite, 52*(2), 517-520.

Cohen, D., & Farley, T. A. (2008). Eating as an automatic behavior. *Preventing Chronic Disease, 5*(1), A23.

Connell, D., Goldberg, J. P., & Folta, S. C. (2001). An intervention to increase fruit and vegetable consumption using audio communications: In-store public service announcements and audiotapes. *Journal of Health Communication, 6*(1), 31-43.

Cook, C., Simmons, G., Swinburn, B., & Stewart, J. (2001). Changing risk behaviours for non-communicable disease in New Zealand working men--is workplace intervention effective? *The New Zealand Medical Journal, 114*(1130), 175-178.

Dayan, E., & Bar-Hillel, M. (2011). Nudge to nobesity II: Menu positions influence food orders. *Judgment and Decision Making, 6*(4), 333-342.

DeGraffe, C., & Hulshof, T. (1996). Effects of weight and energy content of preloads on subsequent appetite and food intake. *Appetite, 26*(2), 139-151.

Devitt, A. A., & Mattes, R. D. (2004). Effects of food unit size and energy density on intake in humans. *Appetite, 42*(2), 213-220.

Diliberti, N., Bordi, P. L., Conklin, M. T., Roe, L. S., & Rolls, B. J. (2004). Increased portion size leads to increased energy intake in a restaurant meal. *Obesity, 12*(3), 562-568.

Dixon, H., Scully, M., Wakefield, M., Kelly, B., Chapman, K., & Donovan, R. (2011). Parent's responses to nutrient claims and sports celebrity endorsements on energy-dense and nutrient-poor foods: An experimental study. *Public Health Nutrition, 14*(6), 1071-1079.

Dubbert, P. M., Johnson, W. G., Schlundt, D. G., & Montague, N. W. (1984). The influence of caloric information on cafeteria food choices. *Journal of Applied Behavior Analysis, 17*(1), 85-92.

Dumanovsky, T., Huang, C. Y., Nonas, C. A., Matte, T. D., Bassett, M. T., & Silver, L. D. (2011). Changes in energy content of lunchtime purchases from fast food restaurants after introduction of calorie labelling: Cross sectional customer surveys. *British Medical Journal, 343*.

Ebbeling, C. B., Garcia-Lago, E., Leidig, M. M., Seger-Shippee, L. G., Feldman, H. A., & Ludwig, D. S. (2007). Altering portion sizes and eating rate to attenuate gorging during a fast food meal: Effects on energy intake. *Pediatrics, 119*(5), 869-875.

Elbel, B., Kersh, R., Brescoll, V. L., & Dixon, L. B. (2009). Calorie labeling and food choices: A first look at the effects on low-income people In New York City. *Health Affairs, 28*(6), w1110-w1121.

Elbel, B., Gyamfi, J., & Kersh, R. (2011). Child and adolescent fast-food choice and the influence of calorie labeling: A natural experiment. *International Journal of Obesity, 35*(4), 493-500.

Ello-Martin, J. A., Ledikwe, J. H., & Rolls, B. J. (2005). The influence of food portion size and energy density on energy intake: Implications for weight management. *American Journal of Clinical Nutrition, 82*(Suppl.1), s236-s241.

Engbers, L. H., van Poppel, M. N. M., Chin A Paw, M. J. M., & van Mechelen, W. (2005). Worksite health promotion programs with environmental changes: A systematic review. *American Journal of Preventive Medicine, 29*(1), 61-70.

Engbers, L. H., van Poppel, M. N., Chin, A. P. M., & van Mechelen, W. (2006). The effects of a controlled worksite environmental intervention on determinants of dietary behavior and self-reported fruit, vegetable and fat intake. *BMC Public Health, 6*, 253.

Engell, D., Kramer, M., Malafi, T., Salomon, M., & Lesher, L. (1996). Effects of effort and social modeling on drinking in humans. *Appetite, 26*(2), 129-138.

Fedoroff, I., Polivy, J., & Peter, H. C. (2003). The specificity of restrained versus unrestrained eaters' responses to food cues: General desire to eat, or craving for the cued food? *Appetite, 41*(1), 7-13.

Finkelstein, E. A., Strombotne, K. L., Chan, N. L., & Krieger, J. (2011). Mandatory menu labeling in one fast-food chain in King County, Washington. *American Journal of Preventive Medicine, 40*(2), 122-127.

Fisher, J. O., Rolls, B. J., & Birch, L. L. (2003). Children's bite size and intake of an entrée are greater with large portions than with age-appropriate or self-selected portions. *The American Journal of Clinical Nutrition, 77*(5), 1164-1170.

Fisher, J. O., Liu, Y., Birch, L. L., & Rolls, B. J. (2007). Effects of portion size and energy density on young children's intake at a meal. *American Journal of Clinical Nutrition, 86*(1), 174-179.

Fisher, J. O., Arreola, A., Birch, L. L., & Rolls, B. J. (2007). Portion size effects on daily energy intake in low-income Hispanic and African American children and their mothers. *The American Journal of Clinical Nutrition, 86*(6), 1709-1716.

Fisher, J. O., & Kral, T. V. (2008). Super-size me: Portion size effects on young children's eating. *Physiology & Behavior, 94*(1), 39-47.

Fiske, A., & Cullen, K. W. (2004). Effects of promotional materials on vending sales of low-fat items in teachers' lounges. *Journal of the American Dietetic Association, 104*(1), 90-93.

Flood, J. E., Roe, L. S., & Rolls, B. J. (2006). The effect of increased beverage portion size on energy intake at a meal. *Journal of the American Dietetic Association, 106*(12), 1984-1990.

Forman, J., Halford, J. C. G., Summe, H., MacDougall, M., & Keller, K. L. (2009). Food branding influences ad libitum intake differently in children depending on weight status. Results of a pilot study. *Appetite, 53*(1), 76-83.

Francis, L. A., & Birch, L. L. (2006). Does eating during television viewing affect preschool children's intake? *Journal of the American Dietetic Association, 106*(4), 598-600.

Freedman, M. R., & Brochado, C. (2010). Reducing portion size reduces food intake and plate waste. *Obesity, 18*(9), 1864-1866.

Freedman, M. R., & Connors, R. (2010). Point-of-purchase nutrition information influences food-purchasing behaviors of college students: A pilot study. *Journal of the American Dietetic Association, 110*(8), 1222-1226.

French, S. A., Jeffery, R. W., Story, M., Breitlow, K. K., Baxter, J. S., Hannan, P., & Snyder, M. P. (2001). Pricing and promotion effects on low-fat vending snack purchases: The CHIPS Study. *American Journal of Public Health, 91*(1), 112-127.

French, S. A., Story, M., Fulkerson, J. A., & Hannan, P. (2004). An environmental intervention to promote lower-fat food choices in secondary schools: Outcomes of the TACOS study. *American Journal of Public Health, 94*(9), 1507-1512.

French, S. A., Hannan, P. J., Harnack, L. J., Mitchell, N. R., Toomey, T. L., & Gerlach, A. (2010). Pricing and availability intervention in vending machines at four bus garages. *Journal of Occupational and Environmental Medicine, 52* (Suppl.1), s29-s33.

Garg, N., Wansink, B., & Inman, J. J. (2007). The influence of incidental affect on consumers' food intake. *Journal of Marketing, 71*(1), 194-206.

Geier, A. B., Rozin, P., & Doros, G. (2006). Unit bias. A new heuristic that helps explain the effect of portion size on food intake. *Psychological Science, 17*(6), 521-525.

Gerend, M. A. (2009). Does calorie information promote lower calorie fast food choices among college students? *Journal of Adolescent Health, 44*(1), 84-86.

Giesen, J. C., Payne, C. R., Havermans, R. C., & Jansen, A. (2011). Exploring how calorie information and taxes on high-calorie foods influence lunch decisions. *The American Journal of Clinical Nutrition, 93*(4), 689-694.

Gittelsohn, J., Vijayadeva, V., Davison, N., Ramirez, V., Cheung, L. W., Murphy, S., & Novotny, R. (2010). A food store intervention trial improves caregiver psychosocial factors and children's dietary intake in Hawaii. *Obesity (Silver Spring), 18* (Suppl.1), s84-s90.

Glanz, K., & Mullis, R. M. (1988). Environmental interventions to promote healthy eating: A review of models, programs, and evidence. *Health Education & Behavior, 15*(4), 395-415.

Glanz, K., & Hoelscher, D. (2004). Increasing fruit and vegetable intake by changing environments, policy and pricing: Restaurant-based research, strategies, and recommendations. *Preventive Medicine, 39* (Suppl.2), s88-s93.

Goldberg, M. E., & Gunasti, K. (2007). Creating an environment in which youths are encouraged to eat a healthier diet. *Journal of Public Policy & Marketing, 26*(2), 162-181.

Gosnell, B. A., Mitchell, J. E., Lancaster, K. L., Burgard, M. A., Wonderlich, S. A., & Crosby, R. D. (2001). Food presentation and energy intake in a feeding laboratory study of subjects with binge eating disorder. *International Journal of Eating Disorders, 30*(4), 441-446.

Greco, A. J., & Johnson, E. B. (1997). Supermarket shoppers' response to mature models in point-of-purchase displays. *Journal of Food Products Marketing, 4*(3), 9-23.

Gueguen, N., & Petr, C. (2006). Odors and consumer behavior in a restaurant. *International Journal of Hospitality Management, 25*(2), 335-339.

Guerrieri, R., Nederkoorn, C., & Jansen, A. (2007). How impulsiveness and variety influence food intake in a sample of healthy women. *Appetite, 48*(1), 119-122.

Guerrieri, R., Nederkoorn, C., & Jansen, A. (2008). The interaction between impulsivity and a varied food environment: Its influence on food intake and overweight. *International Journal of Obesity (London), 32*(4), 708-714.

Harnack, L. J., & French, S. A. (2008). Effect of point-of-purchase calorie labeling on restaurant and cafeteria food choices: A review of the literature. *International Journal of Behavioral Nutriton and Physical Activity, 5*, 51.

Harnack, L. J., French, S. A., Oakes, J. M., Story, M. T., Jeffery, R. W., & Rydell, S. A. (2008). Effects of calorie labeling and value size pricing on fast food meal choices: Results from an experimental trial. *International Journal of Behavioral Nutrition and Physical Activity, 5*, 63.

Hartstein, J., Cullen, K. W., Reynolds, K. D., Harrell, J., Resnicow, K., & Kennel, P. (2008). Impact of portion-size control for school a la carte items: Changes in kilocalories and macronutrients purchased by middle school students. *Journal of the American Dietetic Association, 108*(1), 140-144.

Hassan, L. M., Shiu, E. M. K., & Michaelidou, N. (2010). The influence of nutrition information on choice: The roles of temptation, conflict and self-control. *Journal of Consumer Affairs, 44*(3), 499-515.

Hetherington, M. M., Anderson, A. S., Norton, G. N., & Newson, L. (2006). Situational effects on meal intake: A comparison of eating alone and eating with others. *Physiology & Behavior, 88*(4-5), 498-505.

Hetherington, M. M. (2007). Cues to overeat: Psychological factors influencing overconsumption. *Proceedings of the Nutrition Society, 66*(1), 113-123.

Higgs, S., & Woodward, M. (2009). Television watching during lunch increases afternoon snack intake of young women. *Appetite, 52*(1), 39-43.

Hoefkens, C., Lachat, C., Kolsteren, P., Van Camp, J., & Verbeke, W. (2011). Posting point-of-purchase nutrition information in university canteens does not influence meal choice and nutrient intake. *American Journal of Clinical Nutrition, 94*(2), 562-570.

Hogenkamp, P. S., Mars, M., Stafleu, A., & de, G. (2010). Intake during repeated exposure to low- and high-energy-dense yogurts by different means of consumption. *American Journal of Clinical Nutrition, 91*(4), 841-847.

Horgen, K. B., & Brownell, K. D. (2002). Comparison of price change and health message interventions in promoting healthy food choices. *Health Psychology, 21*(5), 505-512.

Howlett, E. A., Burton, S., Bates, K., & Huggins, K. (2009). Coming to a restaurant near you? Potential consumer responses to nutrition information disclosure on menus. *Journal of Consumer Research, 36*(3), 494-503.

Hrovat, K. B., Harris, K. Z., Leach, A. D., Russell, B. S., Harris, B. V., & Sprecher, D. L. (1994). The new food label, type of fat, and consumer choice. A pilot study. *Archives of Family Medicine, 3*(8), 690-695.

Huang, A., Barzi, F., Huxley, R., Denyer, G., Rohrlach, B., Jayne, K., & Neal, B. (2006). The effects on saturated fat purchases of providing internet shoppers with purchase- specific dietary advice: A randomised trial. *PLoS Clinical Trials, 1*(5), e22.

Jacob, C., Gueguen, N., & Boulbry, G. (2010). L'effet d'elements figuratifs sur le comportement de consommation: Une illustration de l'influence du choix d'un plat dans un restaurant [The effect of incidental information on consumption behaviour: An Illustration with the choice of a meal in a restaurant. With English summary.]. *La Revue des Sciences de Gestion, 45*(242), 61-67.

Jeffery, R. W., French, S. A., Raether, C., & Baxter, J. E. (1994). An environmental intervention to increase fruit and salad purchases in a cafeteria. *Preventive Medicine, 23*(6), 788-792.

Jeffery, R. W., Rydell, S., Dunn, C. L., Harnack, L. J., Levine, A. S., Pentel, P. R., . . . Walsh, E. M. (2007). Effects of portion size on chronic energy intake. *International Journal of Behavioral Nutrition and Physical Activity, 4*, 27.

Just, D., & Wansink, B. (2009). Smarter lunchrooms: Using behavioral economics to improve meal selection. *Choices, 29*(3).

Kahn, B. E., & Wansink, B. (2004). The influence of assortment structure on perceived variety and consumption quantities. *Journal of Consumer Research, 30*(4), 519-533.

Kalnikaitė, V., Yvonne, R., Bird, J., Villar, N., Bachour, K., Payne, S., . . . Krüger, A. (2011). *How to nudge in situ: Designing lambent devices to deliver information salience in supermarkets.* Paper presented at the 13th ACM International conference on Ubiquitous computing, New York, NY, United States.

Kelly, M. T., Wallace, J. M., Robson, P. J., Rennie, K. L., Welch, R. W., Hannon-Fletcher, M. P., . . . Livingstone, M. B. (2009). Increased portion size leads to a sustained increase in energy intake over 4d in normal-weight and overweight men and women. *British Journal of Nutrition, 102*(3), 470-477.

Kiesel, K., & Villas-Boas, S. B. (2009). Can information costs affect consumer choice? Nutritional labels in a supermarket experiment *Working Paper No.1060R2, International Journal of Industrial Organization*. Berkeley, CA: Department of Agricultural and Resource Economics, University of California, Berkeley.

Kimathi, A. N., Gregoire, M. B., Dowling, R. A., & Stone, M. K. (2009). A healthful options food station can improve satisfaction and generate gross profit in a worksite cafeteria. *Journal of the American Dietetic Association 109*(5), 914-917.

Kincaid, C. S., & Corsun, D. L. (2003). Are consultants blowing smoke? An empirical test of the impact of menu layout on item sales. *International Journal of Contemporary Hospitality Management, 15*(4).

Koh, J., & Pliner, P. (2009). The effects of degree of acquaintance, plate size, and sharing on food intake. *Appetite, 52*(3), 595-602.

Kral, T. V., Roe, L. S., & Rolls, B. J. (2002). Does nutrition information about the energy density of meals affect food intake in normal-weight women? *Appetite, 39*(2), 137-145.

Kral, T. V. E., Roe, L. S., & Rolls, B. J. (2004). Combined effects of energy density and portion size on energy intake in women. *The American Journal of Clinical Nutrition, 79*(6), 962-968.

Kral, T. V., & Rolls, B. J. (2004). Energy density and portion size: Their independent and combined effects on energy intake. *Physiology & Behavior, 82*(1), 131-138.

Kral, T. V. E. (2006). Effects on hunger and satiety, perceived portion size and pleasantness of taste of varying the portion size of foods: A brief review of selected studies. *Appetite, 46*(1), 103-105.

Kral, T. V., Kabay, A. C., Roe, L. S., & Rolls, B. J. (2010). Effects of doubling the portion size of fruit and vegetable side dishes on children's intake at a meal. *Obesity (Silver Spring), 18*(3), 521-527.

Leahy, K. E., Birch, L. L., Fisher, J. O., & Rolls, B. J. (2008). Reductions in entree energy density increase children's vegetable intake and reduce energy intake. *Obesity (Silver Spring), 16*(7), 1559-1565.

Ledikwe, J. H., Ello-Martin, J. A., & Rolls, B. J. (2005). Portion sizes and the obesity epidemic. *Journal of Nutrition, 135*(4), 905-909.

Levitsky, D. A., & Youn, T. (2004). The more food young adults are served, the more they overeat. *Journal of Nutrition, 134*(10), 2546-2549.

Levitsky, D. A. (2005). The non-regulation of food intake in humans: Hope for reversing the epidemic of obesity. *Physiology & Behavior, 86*(5), 623-632.

Lin, C.-H., Lin, H.-C., & Lee, S.-H. (2011). The influence of health-related information on variety-seeking behavior: The moderating roles of mood states and gender. *British Food Journal, 113*(11).

Lombardini-Riipinen, C., & Lankoski, L. (2010). *Take off the heater: Utility effect and food environment effect in food consumption decisions.* Paper presented at the 1st Joint EAAE/AAEA Seminar, Munich, Germany.

Looney, S. M., & Raynor, H. A. (2011). Impact of portion size and energy density on snack intake in preschool-aged children. *Journal of the American Dietetic Association, 111*(3), 414-418.

Lowe, M. R., Tappe, K. A., Butryn, M. L., Annunziato, R. A., Coletta, M. C., Ochner, C. N., & Rolls, B. J. (2010). An intervention study targeting energy and nutrient intake in worksite cafeterias. *Eating Behaviors, 11*(3), 144-151.

Maas, J., de Ridder, D. T., de Vet, E., & de Wit, J. B. (2011). Do distant foods increase intake? The effect of food accessibility on consumption. *Psychology & Health*.

Madzharov, A. V., & Block, L. G. (2010). Effects of product unit image on consumption of snack foods. *Journal of Consumer Psychology, 20*(4), 398-409.

Marchiori, D., Waroquier, L., & Klein, O. (2011). Smaller food item sizes of snack foods influence reduced portions and caloric intake in young adults. *Journal of the American Dietetic Association, 111*(5), 727-731.

Marchiori, D., Corneille, O., & Klein, O. (2012). Container size influences snack food intake independently of portion size. *Appetite, 58*(3), 814-817.

Mathey, M. F. A. M., Vanneste, V. G. G., de Graaf, C., de Groot, L. C. P. G. M., & van Staveren, W. A. (2001). Health effect of improved meal ambiance in a Dutch nursing home: A 1-year intervention study. *Preventive Medicine, 32*(5), 416-423.

Mathias, K. C., Rolls, B. J., Birch, L. L., Kral, T. V. E., Hanna, E. L., Davey, A., & Fisher, J. O. (2011). Serving larger portions of fruits and vegetables together at dinner promotes intake of both foods among young children. *Journal of the American Dietetic Association, 20*(10), 1-5.

Mathios, A. D. (2000). The impact of mandatory disclosure laws on product choices: An analysis of the salad dressing market. *Journal of Law and Economics, 43*(2), 651-677.

Mayer, J. A., Heins, J. M., Vogel, J. M., Morrison, D. C., Lankester, L. D., & Jacobs, A. L. (1986). Promoting low-fat entree choices in a public cafeteria. *Journal of Applied Behavior Analysis, 19*(4), 397-402.

Mayer, J. A., Dubbert, P. M., & Elder, J. P. (1989). Promoting nutrition at the point of choice: A review. *Health Education Quarterly, 16*(1), 31-43.

McCarron, A., & Tierney, K. J. (1989). The effect of auditory stimulation on the consumption of soft drinks. *Appetite, 13*(2), 155-159.

Meiselman, H. L., Hedderley, D., Staddon, S. L., Pierson, B. J., & Symonds, C. R. (1994). Effect of effort on meal selection and meal acceptability in a student cafeteria. *Appetite, 23*(1), 43-55.

Mendoza, J. A., Watson, K., & Cullen, K. W. (2010). Change in dietary energy density after implementation of the Texas Public School Nutrition Policy. *Journal of the American Dietetic Association, 110*(3), 434-440.

Meyers, A. W., Stunkard, A. J., & Coll, M. (1980). Food accessibility and food choice. A test of Schachter's externality hypothesis. *Archives of General Psychiatry, 37*(10), 1133-1135.

Miller, D. L., Castellanos, V. H., Shide, D. J., Peters, J. C., & Rolls, B. J. (1998). Effect of fat-free potato chips with and without nutrition labels on fat and energy intakes. *American Journal of Clinical Nutrition, 68*(2), 282-290.

Milliman, R. E. (1982). Using background music to affect the behavior of supermarket shoppers. *The Journal of Marketing, 46*(3), 86-91.

Milliman, R. E. (1986). The influence of background music on the behavior of restaurant patrons. *Journal of Consumer Research, 13*(2), 286-289.

Mishra, A., Mishra, H., & Masters, T. M. (2011). The influence of bite-size on quantity of food consumed: A field study. *Journal of Consumer Research*.

Moray, J., Fu, A., Brill, K., & Mayoral, M. S. (2007). Viewing television while eating impairs the ability to accurately estimate total amount of food consumed. *Bariatric Nursing and Surgical Patient Care, 2*(1), 71-76.

Muller, T. E. (1985). Structural information factors which stimulate the use of nutrition information: A field experiment. *Journal of Marketing Research, 22*(2), 143-157.

Musher-Eizenman, D. R., Young, K. M., Laurene, K., Galliger, C., Hauser, J., & Wagner, O. M. (2010). Children’s sensitivity to external food cues: How distance to serving bowl influences children’s consumption. *Health Education & Behavior, 37*(2), 186-192.

North, A. C., & Hargreaves, D. J. (1998). The effect of music on atmosphere and purchase intentions in a cafeteria. *Journal of Applied Social Psychology, 28*(24), 2254-2273.

North, A. C., Shilcock, A., & Hargreaves, D. J. (2003). The effect of musical style on restaurant customers' spending. *Environment and Behavior, 35*(5), 712-718.

Norton, G. N., Anderson, A. S., & Hetherington, M. M. (2006). Volume and variety: Relative effects on food intake. *Physiology & Behavior, 87*(4), 714-722.

Ogawa, Y., Tanabe, N., Honda, A., Azuma, T., Seki, N., Suzuki, T., & Suzuki, H. (2011). Point-of-purchase health information encourages customers to purchase vegetables: Objective analysis by using a point-of-sales system. *Environmental Health and Preventive Medicine, 16*(4), 239-246.

Painter, J. E., Wansink, B., & Hieggelke, J. B. (2002). How visibility and convenience influence candy consumption. *Appetite, 38*(3), 237-238.

Papies, E. K., & Hamstra, P. (2010). Goal priming and eating behavior: Enhancing self-regulation by environmental cues. *Health Psychology, 29*(4), 384-388.

Patrick, H., & Nicklas, T. A. (2005). A review of family and social determinants of children's eating patterns and diet quality. *Journal of the American College of Nutrition, 24*(2), 83-92.

Peck, J., & Childers, T. L. (2008). If it tastes, smells, sounds, and feels like a duck, then it must be a….: Effects of sensory factors on consumer behaviors. In P. M. Haugtvedt, & F. R. Kardes (Ed.), *Handbook of consumer psychology* (pp. 193-219). New York: Psychology Press.

Peneau, S., Mekhmoukh, A., Chapelot, D., Dalix, A.-M., Airinei, G., Hercberg, S., & Bellisle, F. (2009). Influence of environmental factors on food intake and choice of beverage during meals in teenagers: A laboratory study. *British Journal of Nutrition, 102*(12), 1854-1859.

Perlmutter, C. A., Canter, D. D., & Gregoire, M. B. (1997). Profitability and acceptability of fat- and sodium-modified hot entrees in a worksite cafeteria. *Journal of the American Dietetic Association, 97*(4), 391-395.

Perry, C. L., Bishop, D. B., Taylor, G. L., Davis, M., Story, M., Gray, C., . . . Harnack, L. (2004). A randomized school trial of environmental strategies to encourage fruit and vegetable consumption among children. *Health Education & Behavior, 31*(1), 65-76.

Peterson, S., Duncan, D. P., Null, D. B., Roth, S. L., & Gill, L. (2010). Positive changes in perceptions and selections of healthful foods by college students after a short-term point-of-selection intervention at a dining hall. *Journal of American College Health, 58*(5), 425-431.

Polivy, J., Herman, C. P., Hackett, R., & Kuleshnyk, I. (1986). The effects of self-attention and public attention on eating in restrained and unrestrained subjects. *Journal of Personality and Social Psychology, 50*(6), 1253-1260.

Poothullil, J. M. (2002). Role of oral sensory signals in determining meal size in lean women. *Nutrition, 18*(6), 479-483.

Pulos, E., & Leng, K. (2010). Evaluation of a voluntary menu-labeling program in full-service restaurants. *American Journal of Public Health, 100*(6), 1035-1039.

Quartier, K., Christiaans, H., & Van Cleempoel, K. (2009). *Retail design: Lighting as an atmospheric tool, creating experiences which influence consumers' mood and behaviour in commercial spaces.* Paper presented at the Undisciplined! Design Research Society Conference 2008, Sheffield, UK.

Raynor, H. A., & Wing, R. R. (2007). Package unit size and amount of food: Do both influence intake? *Obesity, 15*(9), 2311-2319.

Raynor, H. A., Van Walleghen, E. L., Niemeier, H., Butryn, M. L., & Wing, R. R. (2009). Do food provisions packaged in single-servings reduce energy intake at breakfast during a brief behavioral weight-loss intervention? *Journal of the American Dietetic Association, 109*(11), 1922-1925.

Richard, L., O'Loughlin, J., Masson, P., & Devost, S. (1999). Healthy menu intervention in restaurants in low-income neighbourhoods: A field experience. *Journal of Nutrition Education, 31*(1), 54-59.

Roberto, C. A., Larsen, P. D., Agnew, H., Baik, J., & Brownell, K. D. (2010). Evaluating the impact of menu labeling on food choices and intake. *American Journal of Public Health, 100*(2), 312-318.

Roefs, A., & Jansen, A. (2004). The effect of information about fat content on food consumption in overweight/obese and lean people. *Appetite, 43*(3), 319-322.

Rolls, B. J., Rowe, E. A., Rolls, E. T., Kingston, B., Megson, A., & Gunary, R. (1981). Variety in a meal enhances food intake in man. *Physiology & Behavior, 26*(2), 215-221.

Rolls, B. J., Rowe, E. A., & Rolls, E. T. (1982). How sensory properties of foods affect human feeding behavior. *Physiology & Behavior, 29*(3), 409-417.

Rolls, B. J. (1985). Experimental analyses of the effects of variety in a meal on human feeding. *American Journal of Clinical Nutrition, 42*(Suppl.5), 932-939.

Rolls, B. J., Castellanos, V. H., Halford, J. C., Kilara, A., Panyam, D., Pelkman, C. L., . . . Thorwart, M. L. (1998). Volume of food consumed affects satiety in men. *American Journal of Clinical Nutrition, 67*(6), 1170-1177.

Rolls, B. J., Bell, E. A., & Waugh, B. A. (2000). Increasing the volume of a food by incorporating air affects satiety in men. *American Journal of Clinical Nutrition, 72*(2), 361-368.

Rolls, B. J., Engell, D., & Birch, L. L. (2000). Serving portion size influences 5-year-old but not 3-year-old children's food intakes. *Journal of the American Dietetic Association, 100*(2), 232-234.

Rolls, B. J., Morris, E. L., & Roe, L. S. (2002). Portion size of food affects energy intake in normal-weight and overweight men and women. *The American Journal of Clinical Nutrition, 76*(6), 1207-1213.

Rolls, B. J., Roe, L. S., Kral, T. V., Meengs, J. S., & Wall, D. E. (2004). Increasing the portion size of a packaged snack increases energy intake in men and women. *Appetite, 42*(1), 63-69.

Rolls, B. J., Roe, L. S., Meengs, J. S., & Wall, D. E. (2004). Increasing the portion size of a sandwich increases energy intake. *Journal of the American Dietetic Association, 104*(3), 367-372.

Rolls, B. J., Roe, L. S., & Meengs, J. S. (2004). Salad and satiety: Energy density and portion size of a first-course salad affect energy intake at lunch. *Journal of the American Dietetic Association, 104*(10), 1570-1576.

Rolls, B. J., Roe, L. S., & Meengs, J. S. (2006). Larger portion sizes lead to a sustained increase in energy intake over 2 days. *Journal of the American Dietetic Association, 106*(4), 543-549.

Rolls, B. J., Roe, L. S., & Meengs, J. S. (2006). Reductions in portion size and energy density of foods are additive and lead to sustained decreases in energy intake. *American Journal of Clinical Nutrition, 83*(1), 11-17.

Rolls, B. J., Roe, L. S., & Meengs, J. S. (2007). The effect of large portion sizes on energy intake is sustained for 11 days. *Obesity (Silver Spring), 15*(6), 1535-1543.

Rolls, B. J., Roe, L. S., Halverson, K. H., & Meengs, J. S. (2007). Using a smaller plate did not reduce energy intake at meals. *Appetite, 49*(3), 652-660.

Rolls, B. J. (2010). Dietary strategies for the prevention and treatment of obesity. *Proceedings of the Nutrition Society, 69*(1), 70-79.

Rolls, B. J., Roe, L. S., & Meengs, J. S. (2010). Portion size can be used strategically to increase vegetable consumption in adults. *American Journal of Clinical Nutrition, 91*(4), 913-922.

Rozin, P., Scott, S., Dingley, M., Urbanek, J. K., Jiang, H., & Kaltenbach, M. (2011). Nudge to nobesity I: Minor changes in accessibility decrease food intake. *Judgment and Decision Making, 6*(4), 323-332.

Russo, J., Staelin, R., Nolan, C. A., Russell, G. J., & Metcalf, B. L. (1986). Nutrition information in the supermarket. *Journal of Consumer Research, 13*(1), 48-70.

Sacks, G., Rayner, M., & Swinburn, B. (2009). Impact of front-of-pack 'traffic-light' nutrition labelling on consumer food purchases in the UK. *Health Promotion International, 24*(4), 344-352.

Sacks, G., Tikellis, K., Millar, L., & Swinburn, B. (2011). Impact of 'traffic-light' nutrition information on online food purchases in Australia. *Australian and New Zealand Journal of Public Health, 35*(2), 122-126.

Scott, M. L., Nowlis, S. M., Mandel, N., & Morales, A. C. (2008). The effects of reduced food size and package size on the consumption behavior of restrained and unrestrained eaters. *Journal of Consumer Research, 35*(3), 391-405.

Seymour, J. D., Yaroch, A. L., Serdula, M., Blanck, H. M., & Khan, L. K. (2004). Impact of nutrition environmental interventions on point-of-purchase behavior in adults: A review. *Preventive Medicine, 39* (Suppl.2), s108-s136.

Shah, M., Schroeder, R., Winn, W., & Adams-Huet, B. (2011). A pilot study to investigate the effect of plate size on meal energy intake in normal weight and overweight/obese women. *Journal of Human Nutrition and Dietetics, 24*(6), 612-615.

Sharma, S., Wagle, A., Sucher, K., & Bugwadia, N. (2011). Impact of point of selection nutrition information on meal choices at a table-service restaurant. *Journal of Foodservice Business Research, 14*(2), 146-161.

Shepherd, J., Harden, A., Rees, R., Brunton, G., Garcia, J., Oliver, S., & Oakley, A. (2006). Young people and healthy eating: A systematic review of research on barriers and facilitators. *Health Education Research, 21*(2), 239-257.

Shide, D. J., & Rolls, B. J. (1995). Information about the fat content of preloads influences energy intake in healthy women. *Journal of the American Dietetic Association, 95*(9), 993-998.

Shimizu, M., Payne, C. R., & Wansink, B. (2010). When snacks become meals: How hunger and environmental cues bias food intake. *International Journal of Behavioral Nutrition and Physical Activity, 7*(63).

Sigurdsson, V., Larsen, N. M., & Gunnarsson, D. (2011). An in-store experimental analysis of consumers' selection of fruits and vegetables. *The Service Industries Journal, 31*(15), 2587-2602.

Smith, J. M., & Ditschun, T. L. (2009). Controlling satiety: How environmental factors influence food intake. *Trends in Food Science & Technology, 20*(6-7), 271-277.

Sobal, J., & Wansink, B. (2007). Kitchenscapes, tablescapes, platescapes, and foodscapes. *Environment and Behavior, 39*(1), 124-142.

Sorensen, L. B., Moller, P., Flint, A., Martens, M., & Raben, A. (2003). Effect of sensory perception of foods on appetite and food intake: A review of studies on humans. *International Journal on Obesity and Related Metabolic Disorders, 27*(10), 1152-1166.

Sorensen, G., Linnan, L., & Hunt, M. K. (2004). Worksite-based research and initiatives to increase fruit and vegetable consumption. *Preventive Medicine, 39* (Suppl.2), s94-s100.

Spence, C., & Shankar, M. U. (2010). The influence of auditory cues on the perception of, and responses to, food and drink. *Journal of Sensory Studies, 25*(3), 406-430.

Spiegel, T. A., Kaplan, J. M., Tomassini, A., & Stellar, E. (1993). Bite size, ingestion rate, and meal size in lean and obese women. *Appetite, 21*(2), 131-145.

Spill, M. K., Birch, L. L., Roe, L. S., & Rolls, B. J. (2010). Eating vegetables first: The use of portion size to increase vegetable intake in preschool children. *The American Journal of Clinical Nutrition, 91*(5), 1237-1243.

Spill, M. K., Birch, L. L., Roe, L. S., & Rolls, B. J. (2011). Serving large portions of vegetable soup at the start of a meal affected children's energy and vegetable intake. *Appetite, 57*(1), 213-219.

Steenhuis, I., van Assema, P., van Breukelen, G., Glanz, K., Kok, G., & de Vries, H. (2004). The impact of educational and environmental interventions in Dutch worksite cafeterias. *Health Promotion International, 19*(3), 335-343.

Steenhuis, I. H., & Vermeer, W. M. (2009). Portion size: Review and framework for interventions. *The International Journal of Behavioral Nutrition and Physical Activity, 6*, 58.

Steenhuis, I. H. M., Kroeze, W., Vyth, E. L., Valk, S., Verbauwen, R., & Seidell, J. C. (2010). The effects of using a nutrition logo on consumption and product evaluation of a sweet pastry. *Appetite, 55*(3), 707-709.

Stroebele, N., & de Castro, J. M. (2004). Effect of ambience on food intake and food choice. *Nutrition, 20*(9), 821-838.

Stroebele, N., Ogden, L. G., & Hill, J. O. (2009). Do calorie-controlled portion sizes of snacks reduce energy intake? *Appetite, 52*(3), 793-796.

Stubbs, R. J., Johnstone, A. M., Mazlan, N., Mbaiwa, S. E., & Ferris, S. (2001). Effect of altering the variety of sensorially distinct foods, of the same macronutrient content, on food intake and body weight in men. *European Journal of Clinical Nutrition, 55*(1), 19-28.

Sutherland, L. A., Kaley, L. A., & Fischer, L. (2010). Guiding stars: The effect of a nutrition navigation program on consumer purchases at the supermarket. *American Journal of Clinical Nutrition, 91*(4), s1090-s1094.

Tandon, P. S., Wright, J., Zhou, C., Rogers, C. B., & Christakis, D. A. (2010). Nutrition menu labeling may lead to lower-calorie restaurant meal choices for children. *Pediatrics, 125*(2), 244-248.

Tandon, P. S., Zhou, C., Chan, N. L., Lozano, P., Couch, S. C., Glanz, K., . . . Saelens, B. E. (2011). The impact of menu labeling on fast-food purchases for children and parents. *American Journal of Preventive Medicine, 41*(4), 434-438.

Temple, J. L., Giacomelli, A. M., Roemmich, J. N., & Epstein, L. H. (2008). Dietary variety impairs habituation in children. *Health Psychology, 27*(Suppl.1), s10-s19.

Temple, J. L., Johnson, K., Recupero, K., & Suders, H. (2010). Nutrition labels decrease energy intake in adults consuming lunch in the laboratory. *Journal of the American Dietetic Association, 110*(7), 1094-1097.

Temple, J. L., Johnson, K. M., Archer, K., Lacarte, A., Yi, C., & Epstein, L. H. (2011). Influence of simplified nutrition labeling and taxation on laboratory energy intake in adults. *Appetite, 57*(1), 184-192.

Thorndike, A. N., Sonnenberg, L., Riis, J., Barraclough, S., & Levy, D. E. (2012). A 2-Phase labeling and choice architecture intervention to improve healthy food and beverage choices. *American Journal of Public Health, 102*(3), 527-533.

Turley, L. W., & Milliman, R. E. (2000). Atmospheric effects on shopping behavior: A review of the experimental evidence. *Journal of Business Research, 49*(2), 193-211.

Ueland, Ø., Cardello, A. V., Merrill, E. P., & Lesher, L. L. (2009). Effect of portion size information on food intake. *Journal of the American Dietetic Association, 109*(1), 124-127.

Vadiveloo, M. K., Dixon, L. B., & Elbel, B. (2011). Consumer purchasing patterns in response to calorie labeling legislation in New York City. *International Journal of Behavioral Nutrition and Physical Activity, 8*, 51.

Van den Bergh, B., Schmitt, J., & Warlop, L. (2011). Embodied Myopia. *Journal of Marketing Research, 48*(6), 1033-1044.

van Dongen, M. V., De Graaf, C., Siebelink, E., & Kok, F. J. (2009). Hidden fat facilitates passive overconsumption. *Journal of Nutrition, 139*(2), 394-399.

van Herpen, E., & Trijp, H. C. (2011). Front-of-pack nutrition labels. Their effect on attention and choices when consumers have varying goals and time constraints. *Appetite, 57*(1), 148-160.

van Kleef, E., Shimizu, M., & Wansink, B. (2011). Serving bowl selection biases the amount of food served. *Journal of Nutrition Education and Behavior, 44*(1), 66-70.

Variyam, J. N. (2008). Do nutrition labels improve dietary outcomes? *Health Economics, 17*(6), 695-708.

Vermeer, W. M., Steenhuis, I. H., Leeuwis, F. H., Bos, A. E., de Boer, M., & Seidell, J. C. (2010). Portion size labeling and intended soft drink consumption: The impact of labeling format and size portfolio. *Journal of Nutrition Education and Behavior, 42*(6), 422-426.

Vermeer, W. M., Steenhuis, I. H. M., Leeuwis, F. H., Bos, A. E. R., de Boer, M., & Seidell, J. C. (2011). View the label before you view the movie: A field experiment into the impact of portion size and guideline daily amounts labelling on soft drinks in cinemas. *BMC Public Health, 11*, 438.

Vyth, E. L., Steenhuis, I. H., Vlot, J. A., Wulp, A., Hogenes, M. G., Looije, D. H., . . . Seidell, J. C. (2010). Actual use of a front-of-pack nutrition logo in the supermarket: Consumers' motives in food choice. *Public Health Nutrition, 13*(11), 1882-1889.

Vyth, E. L., Steenhuis, I. H. M., Heymans, M. W., Roodenburg, A. J. C., Brug, J., & Seidell, J. C. (2011). Influence of placement of a nutrition logo on cafeteria menu items on lunchtime food choices at Dutch work sites. *Journal of the American Dietetic Association, 111*(1), 131-136.

Wagner, J. L., & Winett, R. A. (1988). Prompting one low-fat, high-fiber selection in a fast-food restaurant. *Journal of Applied Behavior Analysis, 21*(2), 179-185.

Wansink, B. (1996). Can package size accelerate usage volume? *The Journal of Marketing, 60*(3), 1-14.

Wansink, B., Kent, R. J., & Hoch, S. J. (1998). An anchoring and adjustment model of purchase quantity decisions. *Journal of Marketing Research, 35*(1), 71-81.

Wansink, B., & Park, S. B. (2001). At the movies: How external cues and perceived taste impact consumption volume. *Food Quality and Preference, 12*(1), 69-74.

Wansink, B., & van Ittersum, K. (2003). Bottoms up! The influence of elongation on pouring and consumption volume. *Journal of Consumer Research, 30*(3), 455-463.

Wansink, B. (2004). Environmental factors that increase the food intake and consumption volume of unknowing consumers. *Annual Review of Nutrition, 24*, 455-479.

Wansink, B., & Cheney, M. M. (2005). Super bowls: Serving bowl size and food consumption. *Journal of the American Medical Association, 293*(14), 1727-1728.

Wansink, B., & Kim, J. (2005). Bad popcorn in big buckets: Portion size can influence intake as much as taste. *Journal of Nutrition Education and Behavior, 37*(5), 242-245.

Wansink, B., Painter, J. E., & North, J. (2005). Bottomless bowls: Why visual cues of portion size may influence intake. *Obesity Research, 13*(1), 93-100.

Wansink, B., & Chandon, P. (2006). Can "low-fat" nutrition labels lead to obesity? *Journal of Marketing Research, 43*, 605-617.

Wansink, B., Painter, J. E., & Lee, Y. K. (2006). The office candy dish: Proximity's influence on estimated and actual consumption. *International Journal of Obesity, 30*(5), 871-875.

Wansink, B., van Ittersum, K., & Painter, J. E. (2006). Ice cream illusions: Bowls, spoons, and self-served portion sizes. *American Journal of Preventive Medicine, 31*(3), 240-243.

Wansink, B., & van Ittersum, K. (2007). Portion size me: Downsizing our consumption norms. *Journal of the American Dietetic Association, 107*(7), 1103-1106.

Wansink, B. (2010). From mindless eating to mindlessly eating better. *Physiology & Behavior, 100*(5), 454-463.

Wardle, J., & Solomons, W. (1994). Naughty but nice: A laboratory study of health information and food preferences in a community sample. *Health Psychology, 13*(2), 180-183.

Weijzen, P. L. G., Liem, D. G., Zandstra, E. H., & de Graaf, C. (2008). Sensory specific satiety and intake: The difference between nibble- and bar-size snacks. *Appetite, 50*(2-3), 435-442.

Whitaker, R. C., Wright, J. A., Finch, A. J., & Psaty, B. M. (1993). An environmental intervention to reduce dietary fat in school lunches. *Pediatrics, 91*(6), 1107-1111.

Whitaker, R. C., Wright, J. A., Koepsell, T. D., Finch, A. J., & Psaty, B. M. (1994). Randomized intervention to increase children's selection of low-fat foods in school lunches. *Journal of Pediatrics, 125*(4), 535-540.

Wilbur, C. S., Zifferblatt, S. M., Pinsky, J. L., & Zifferblatt, S. (1981). Healthy vending: A cooperative pilot research program to stimulate good health in the marketplace. *Preventive Medicine, 10*(1), 85-93.

Wilcox, K., Vallen, B., Block, L., & Fitzsimons, G. J. (2009). Vicarious goal fulfillment: When the mere presence of a healthy option leads to an ironically indulgent decision. *Journal of Consumer Research, 36*(3), 380-393.

Winett, R. A., Moore, J. F., Wagner, J. L., Hite, L. A., Leahy, M., Neubauer, T. E., . . . Mundy, L. (1991). Altering shoppers' supermarket purchases to fit nutritional guidelines: An interactive information system. *Journal of Applied Behavior Analysis, 24*(1), 95-105.

Wisdom, J., Downs, J. S., & Loewenstein, G. (2010). Promoting healthy choices: Information versus convenience. *American Economic Journal: Applied Economics, 2*(2), 164-178.

Yamamoto, J. A., Yamamoto, J. B., Yamamoto, B. E., & Yamamoto, L. G. (2005). Adolescent fast food and restaurant ordering behavior with and without calorie and fat content menu information. *Journal of Adolescent Health, 37*(5), 397-402.

Yeomans, M. R., Lartamo, S., Procter, E. L., Lee, M. D., & Gray, R. W. (2001). The actual, but not labelled, fat content of a soup preload alters short-term appetite in healthy men. *Physiology & Behavior, 73*(4), 533-540.

Yeomans, M. R., Gould, N. J., Leitch, M., & Mobini, S. (2009). Effects of energy density and portion size on development of acquired flavour liking and learned satiety. *Appetite, 52*(2), 469-478.

Zampini, M., & Spence, C. (2010). Assessing the role of sound in the perception of food and drink. *Chemosensory Perception, 3*(1), 57-67.

PHYSICAL ACTIVITY

Adams, J., & White, M. (2002). A systematic approach to the development and evaluation of an intervention promoting stair use. *Health Education Journal, 61*(3), 272-286.

Andersen, R. E., Franckowiak, S. C., Snyder, J., Bartlett, S. J., & Fontaine, K. R. (1998). Can inexpensive signs encourage the use of stairs? Results from a community intervention. *Annals of Internal Medicine, 129*(5), 363-369.

Andersen, R., Franckowiak, S., Zuzak, K., Cummings, E., Bartlett, S., & Crespo, C. (2006). Effects of a culturally sensitive sign on the use of stairs in African American commuters. *Sozial- und Präventivmedizin [Social and Preventive Medicine], 51*(6), 373-380.

Anderson, A. S., Porteous, L. E. G., Foster, E., Higgins, C., Stead, M., Hetherington, M., . . . Adamson, A. J. (2005). The impact of a school-based nutrition education intervention on dietary intake and cognitive and attitudinal variables relating to fruits and vegetables. *Public Health Nutrition, 8*(6), 650-656.

Badland, H. M., & Schofield, G. M. (2005). Posters in a sample of professional worksites have no effect on objectively measured physical activity. *Health Promotion Journal of Australia, 16*(1), 78-81.

Barwood, M. J., Weston, N. J. V., Thelwell, R., & Page, J. (2009). A motivational music and video intervention improves high-intensity exercise performance. *Journal of Sports Science and Medicine, 8*(3), 435-442.

Benden, M. E., Blake, J. J., Wendel, M. L., & Huber, J. C. (2011). The impact of stand-biased desks in classrooms on calorie expenditure in children. *American Journal of Public Health, 101*(8), 1433-1436.

Blake, H., Lee, S., Stanton, T., & Gorely, T. (2008). Workplace intervention to promote stair-use in an NHS setting. *International Journal of Workplace Health Management, 1*(3), 162-175.

Blamey, A., Mutrie, N., & Aitchison, T. (1995). Health promotion by encouraged use of stairs. *British Medical Journal, 311*(7000), 289-290.

Boen, F., Maurissen, K., & Opdenacker, J. (2010). A simple health sign increases stair use in a shopping mall and two train stations in Flanders, Belgium. *Health Promotion International, 25*(2), 183-191.

Boutelle, K. N., Jeffery, R. W., Murray, D. M., & Schmitz, M. K. (2001). Using signs, artwork, and music to promote stair use in a public building. *American Journal of Public Health, 91*(12), 2004-2006.

Cardon, G., Labarque, V., Smits, D., & Bourdeaudhuij, I. D. (2009). Promoting physical activity at the pre-school playground: The effects of providing markings and play equipment. *Preventive Medicine, 48*(4), 335-340.

Cheung, P. P., Chow, B. C., & Parfitt, G. (2008). Using environmental stimuli in physical activity intervention for school teachers: A pilot study *International Electronic Journal of Health Education, 11*, 47-56.

Coleman, K. J., & Gonzalez, E. C. (2001). Promoting stair use in a US-Mexico border community. *American Journal of Public Health, 91*(12), 2007-2009.

Cook, C., Simmons, G., Swinburn, B., & Stewart, J. (2001). Changing risk behaviours for non-communicable disease in New Zealand working men--is workplace intervention effective? *The New Zealand Medical Journal, 114*(1130), 175-178.

Dolan, M. S., Weiss, L. A., Lewis, R. A., Pietrobelli, A., Heo, M., & Faith, M. S. (2006). 'Take the stairs instead of the escalator': Effect of environmental prompts on community stair use and implications for a national 'Small Steps' campaign. *Obesity Reviews, 7*(1), 25-32.

Dunn, A. L., Andersen, R. E., & Jakicic, J. M. (1998). Lifestyle physical activity interventions. History, short- and long-term effects, and recommendations. *American Journal of Preventive Medicine, 15*(4), 398-412.

Engbers, L. H., van Poppel, M. N. M., Chin A Paw, M. J. M., & van Mechelen, W. (2005). Worksite health promotion programs with environmental changes: A systematic review. *American Journal of Preventive Medicine, 29*(1), 61-70.

Eves, F. F., & Masters, R. S. (2006). An uphill struggle: Effects of a point-of-choice stair climbing intervention in a non-English speaking population. *International Journal of Epidemiology, 35*(5), 1286-1290.

Eves, F. F., & Webb, O. J. (2006). Worksite interventions to increase stair climbing: Reasons for caution. *Preventive Medicine, 43*(1), 4-7.

Eves, F. F., Webb, O. J., & Mutrie, N. (2006). A workplace intervention to promote stair climbing: Greater effects in the overweight. *Obesity (Silver Spring), 14*(12), 2210-2216.

Eves, F. F. (2008). All choices are not equal: Effects of context on point-of-choice prompts for stair climbing. *Obesity Reviews, 9*(1), 83-84; author reply 85-86.

Eves, F. F., Lewis, A. L., & Griffin, C. (2008). Modelling effects of stair width on rates of stair climbing in a train station. *Preventive Medicine, 47*(3), 270-272.

Eves, F. F., Masters, R. S., & McManus, A. M. (2008). Effects of point-of-choice stair climbing interventions in Hong Kong. *Hong Kong Medical Journal, 14*(Suppl.5), 36-39.

Eves, F. F., Olander, E. K., Nicoll, G., Puig-Ribera, A., & Griffin, C. (2009). Increasing stair climbing in a train station: The effects of contextual variables and visibility. *Journal of Environmental Psychology, 29*(2), 300-303.

Eves, F. F. (2010). Point-of-decision prompts to increase stair use. *American Journal of Preventive Medicine, 38*(5), 573-574.

Eves, F. F., Olander, E. K., Webb, O. J., Griffin, C., & Chambers, J. (2012). Likening stairs in buildings to climbing a mountain: Self-reports of expected effects on stair climbing and objective measures of effectiveness. *Psychology of Sport and Exercise, 13*(2), 170-176.

Faskunger, J., Poortvliet, E., Nylund, K., & Rossen, J. (2003). Effect of an environmental barrier to physical activity on commuter stair use. *Scandinavian Journal of Nutrition [Naringsforskning], 47*(1), 26-28.

Foster, C., & Hillsdon, M. (2004). Changing the environment to promote health-enhancing physical activity. *Journal of Sports Sciences, 22*(8), 755-769.

Foster, C., Hillsdon, M., Cavill, N., Bull, F., Buxton, K., & Crombie, H. (2006). Interventions that use the environment to encourage physical activity *Evidence review*. London, UK: National Institute for Health and Clinical Excellence.

Iversen, M. K., Handel, M. N., Jensen, E. N., Frederiksen, P., & Heitmann, B. L. (2007). Effect of health-promoting posters placed on the platforms of two train stations in Copenhagen, Denmark, on the choice between taking the stairs or the escalators: A secondary publication. *International Journal of Obesity, 31*(6), 950-955.

Jenum, A. K., Lorentzen, C. A. N., & Ommundsen, Y. (2009). Targeting physical activity in a low socioeconomic status population: Observations from the Norwegian "Romsas in Motion" study. *British Journal of Sports Medicine, 43*(1), 64-69.

Kahn, E. B., Ramsey, L. T., Brownson, R. C., Heath, G. W., Howze, E. H., Powell, K. E., . . . Corso, P. (2002). The effectiveness of interventions to increase physical activity. A systematic review. *American Journal of Preventive Medicine, 22*(Suppl.4), 73-107.

Kerr, J., Eves, F., & Carroll, D. (2000). Posters can prompt less active people to use the stairs. *Journal of Epidemiology & Community Health, 54*(12), 942.

Kerr, J., Eves, F., & Carroll, D. (2001). Encouraging stair use: Stair-riser banners are better than posters. *American Journal of Public Health, 91*(8), 1192-1193.

Kerr, J., Eves, F., & Carroll, D. (2001). Six-month observational study of prompted stair climbing. *Preventive Medicine, 33*(5), 422-427.

Kerr, J., Eves, F. F., & Carroll, D. (2001). The influence of poster prompts on stair use: The effects of setting, poster size and content. *British Journal of Health Psychology, 6*(Pt.4), 397-405.

Kerr, N. A., Yore, M. M., Ham, S. A., & Dietz, W. H. (2004). Increasing stair use in a worksite through environmental changes. *American Journal of Health Promotion, 18*(4), 312-315.

Kwak, L., Kremers, S. P., van Baak, M. A., & Brug, J. (2007). A poster-based intervention to promote stair use in blue- and white-collar worksites. *Preventive Medicine, 45*(2-3), 177-1781.

Lanningham-Foster, L., Foster, R. C., McCrady, S. K., Manohar, C. U., Jensen, T. B., Mitre, N. G., . . . Levine, J. A. (2008). Changing the school environment to increase physical activity in children. *Obesity (Silver Spring), 16*(8), 1849-1853.

Lewis, A., & Eves, F. (2011). Specific effects of a calorie-based intervention on stair climbing in overweight commuters. *Annals of Behavioral Medicine, 42*(2), 257-261.

Lewis, A. L., & Eves, F. F. (2012). Prompts to increase stair climbing in stations: The effect of message complexity. *Journal of Physical Activity & Health, In Press.*

Lewis, A. L., & Eves, F. F. (2012). Testing the theory underlying the success of point-of-choice prompts: A multi-component stair climbing intervention. *Psychology of Sport and Exercise, 13*(2), 126-132.

Loucaides, C. A., Jago, R., & Charalambous, I. (2009). Promoting physical activity during school break times: Piloting a simple, low cost intervention. *Preventive Medicine, 48*(4), 332-334.

Marshall, A. L., Bauman, A. E., Patch, C., Wilson, J., & Chen, J. (2002). Can motivational signs prompt increases in incidental physical activity in an Australian health-care facility? *Health Education Research, 17*(6), 743-749.

Meyer, P., Kayser, B., Kossovsky, M. P., Sigaud, P., Carballo, D., Keller, P. F., . . . Mach, F. (2010). Stairs instead of elevators at workplace: Cardioprotective effects of a pragmatic intervention. *European Journal of Cardiovascular Prevention & Rehabilitation, 17*(5), 569-575.

Muller-Riemenschneider, F., Nocon, M., Reinhold, T., & Willich, S. N. (2010). Promotion of physical activity using point-of-decision prompts in Berlin underground stations. *International Journal of Environmental Research and Public Health, 7*(8), 3063-3070.

Nicoll, G., & Zimring, C. (2009). Effect of innovative building design on physical activity. *Journal of Public Health Policy, 30* (Suppl.1), s111-s123.

Nocon, M., Muller-Riemenschneider, F., Nitzschke, K., & Willich, S. N. (2010). Review Article: Increasing physical activity with point-of-choice prompts--a systematic review. *Scandinavian Journal of Public Health, 38*(6), 633-638.

Nomura, T., Yoshimoto, Y., Akezaki, Y., & Sato, A. (2009). Changing behavioral patterns to promote physical activity with motivational signs. *Environmental Health and Preventive Medicine, 14*(1), 20-25.

Olander, E. K., Eves, F. F., & Puig-Ribera, A. (2008). Promoting stair climbing: Stair-riser banners are better than posters… sometimes. *Preventive Medicine, 46*(4), 308-310.

Olander, E. K., & Eves, F. F. (2011). Elevator availability and its impact on stair use in a workplace. *Journal of Environmental Psychology, 31*(2), 200-206.

Olander, E. K., & Eves, F. F. (2011). Effectiveness and cost of two stair-climbing interventions—less is more. *American Journal of Health Promotion, 25*(4), 231-236.

Pillay, J. D., Kolbe-Alexander, T., Achmat, M., Carstene, M., & Lambert, E. V. (2009). Are point-of-decision prompts in a sports science and medicine centre effective in changing the prevalence of stair usage? A preliminary study. *South African Journal of Sports Medicine, 21*(2).

Puig-Ribera, A., & Eves, F. F. (2010). Promoting stair climbing in Barcelona: Similarities and differences with interventions in English-speaking populations. *European Journal of Public Health, 20*(1), 100-102.

Ridgers, N. D., Stratton, G., Fairclough, S. J., & Twisk, J. W. R. (2007). Long-term effects of a playground markings and physical structures on children's recess physical activity levels. *Preventive Medicine, 44*(5), 393-397.

Ridgers, N., Stratton, G., Fairclough, S., & Twisk, J. (2007). Children's physical activity levels during school recess: A quasi-experimental intervention study. *International Journal of Behavioral Nutrition and Physical Activity, 4*(1), 19.

Ridgers, N. D., Fairclough, S. J., & Stratton, G. (2010). Twelve-month effects of a playground intervention on children's morning and lunchtime recess physical activity levels. *Journal of Physical Activity & Health, 7*(2), 167-175.

Rogers, Y., Hazlewood, W. R., Marshall, P., Dalton, N., & Hetrich, S. (2010). Ambient influence: Can twinkly lights lure and abstract representations trigger behavioural change? *UbiComp 2010, 26-29*, 261-270.

Sallis, J. F., Bauman, A., & Pratt, M. (1998). Environmental and policy interventions to promote physical activity. *American Journal of Preventive Medicine, 15*(4), 379-397.

Soler, R. E., Leeks, K. D., Buchanan, L. R., Brownson, R. C., Heath, G. W., & Hopkins, D. H. (2010). Point-of-decision prompts to increase stair use. A systematic review update. *American Journal of Preventive Medicine, 38*(Suppl.2), s292-s300.

Stratton, G., & Mullan, E. (2005). The effect of multicolor playground markings on children's physical activity level during recess. *Preventive Medicine, 41*(5-6), 828-833.

Thompson, W. G., Foster, R. C., Eide, D. S., & Levine, J. A. (2008). Feasibility of a walking workstation to increase daily walking. *British Journal of Sports Medicine, 42*(3), 225-228.

van Houten, R. V., Nau, P. A., & Merrigan, M. (1981). Reducing elevator energy use: A comparison of posted feedback and reduced elevator convenience. *Journal of Applied Behavior Analysis, 14*(4), 377-387.

van Nieuw-Amerongen, M. E., Kremers, S. P. J., de Vries, N. K., & Kok, G. (2011). The use of prompts, increased accessibility, visibility, and aesthetics of the stairwell to promote stair use in a university building. *Environment and Behavior, 43*(1), 131-139.

Webb, O. J., & Cheng, T.-F. (2010). An informational stair climbing intervention with greater effects in overweight pedestrians. *Health Education Research, 25*(6), 936-944.

Webb, O. J., & Eves, F. F. (2007). Effects of environmental changes in a stair climbing intervention: generalization to stair descent. *American Journal of Health Promotion, 22*(1), 38-44.

Webb, O. J., Eves, F. F., & Kerr, J. (2011). A statistical summary of mall-based stair-climbing interventions. *Journal of Physical Activity & Health, 8*(4), 558-565.

Wu, S., Cohen, D., Shi, Y., Pearson, M., & Sturm, R. (2011). Economic analysis of physical activity interventions. *American Journal of Preventive Medicine, 40*(2), 149-158.

Zimring, C., Joseph, A., Nicoll, G. L., & Tsepas, S. (2005). Influences of building design and site design on physical activity: Research and intervention opportunities. *American Journal of Preventive Medicine, 28*(2 Suppl.2), 186-193.

ALCOHOL

Areni, C. S., & Kim, D. (1993). The influence of background music on shopping behavior: Classical versus top-forty music in a wine store. *Advances in Consumer Research, 20*, 336-340.

Areni, C. S., & Kim, D. (1994). The influence of in-store lighting on consumers' examination of merchandise in a wine store. *International Journal of Research in Marketing, 11*(2), 117-125.

Areni, C. S., Duhan, D. F., & Kiecker, P. (1999). Point-of-purchase displays, product organization and brand purchase likelihoods. *Journal of the Academy of Marketing Science, 27*(4), 428-441.

Bailey, J., Poole, R., Zinovieff, F., Robinson, C. A., Parry, O., Tocque, K., & Kennedy, L. (2011). Achieving positive change in the drinking culture of Wales *Research report*. Wrexham and Bangor, Wales: Glyndwr University / Bangor University.

Gueguen, N., Le Guellec, H., & Jacob, C. (2004). Sound level of background music and alcohol consumption: An empirical evaluation. *Perceptual and Motor Skills, 99*(1), 34-38.

Gueguen, N., & Petr, C. (2006). Odors and consumer behavior in a restaurant. *International Journal of Hospitality Management, 25*(2), 335-339.

Gueguen, N., Jacob, C., Le Guellec, H., Morineau, T., & Lourel, M. (2008). Sound level of environmental music and drinking behavior: A field experiment with beer drinkers. *Alcoholism, Clinical and Experimental Research, 32*(10), 1795-1798.

Hughes, K., Quigg, Z., Eckley, L., Bellis, M., Jones, L., Calafat, A., . . . van, H. (2011). Environmental factors in drinking venues and alcohol-related harm: The evidence base for European intervention. *Addiction, 106*(Suppl.1), 37-46.

Jacob, C. (2006). Styles of background music and consumption in a bar: An empirical evaluation. *International Journal of Hospitality Management, 25*(4), 716-720.

Jones, S. C., & Gregory, P. (2009). The impact of more visible standard drink labelling on youth alcohol consumption: Helping young people drink (ir)responsibly? *Drug and Alcohol Review, 28*(3), 230-234.

Kelly-Weeder, S., Phillips, K., & Rounseville, S. (2011). Effectiveness of public health programs for decreasing alcohol consumption. *Patient Intelligence, 2011*(3), 29-38.

MacKinnon, D. P., Pentz, M. A., & Stacy, A. W. (1993). The alcohol warning label and adolescents: The first year. *American Journal of Public Health, 83*(4), 585-587.

Milliman, R. E. (1986). The influence of background music on the behavior of restaurant patrons. *Journal of Consumer Research, 13*(2), 286-289.

North, A. C., Hargreaves, D. J., & McKendrick, J. (1997). In-store music affects product choice. *Nature, 390*(6656), 132.

North, A. C., Hargreaves, D. J., & McKendrick, J. (1999). The influence of in-store music on wine selections. . *Journal of Applied Psychology, 84*(2), 271-276.

North, A. C., Shilcock, A., & Hargreaves, D. J. (2003). The effect of musical style on restaurant customers' spending. *Environment and Behavior, 35*(5), 712-718.

Spence, C., & Shankar, M. U. (2010). The influence of auditory cues on the perception of, and responses to, food and drink. *Journal of Sensory Studies, 25*(3), 406-430.

Stockwell, T. (2006). A review of research into the impacts of alcohol warning labels on attitudes and behaviour *Centre of Addictions Research of BC*. Victoria, BC: Centre for Addictions Research of British Columbia.

Stockley, C. S. (2001). The effectiveness of strategies such as health warning labels to reduce alcohol-related harms: An Australian perspective. *International Journal of Drug Policy, 12*(2), 153-166.

Turley, L. W., & Milliman, R. E. (2000). Atmospheric effects on shopping behavior: A review of the experimental evidence. *Journal of Business Research, 49*(2), 193-211.

Wansink, B., & van Ittersum, K. (2003). Bottoms up! The influence of elongation on pouring and consumption volume. *Journal of Consumer Research, 30*(3), 455-463.

Wansink, B. (2004). Environmental factors that increase the food intake and consumption volume of unknowing consumers. *Annual Review of Nutrition, 24*, 455-479.

Wansink, B., & van Ittersum, K. (2005). Shape of glass and amount of alcohol poured: Comparative study of effect of practice and concentration. *British Medical Journal, 331*(7531), 1512-1514.

Wansink, B. (2010). From mindless eating to mindlessly eating better. *Physiology & Behavior, 100*(5), 454-463.

Wilkinson, C., & Room, R. (2009). Warnings on alcohol containers and advertisements: International experience and evidence on effects. *Drug and Alcohol Review, 28*(4), 426-435.

Zampini, M., & Spence, C. (2010). Assessing the role of sound in the perception of food and drink. *Chemosensory Perception, 3*(1), 57-67.

TOBACCO

Borland, R. (1997). Tobacco health warnings and smoking-related cognitions and behaviours. *Addiction, 92*(11), 1427-1435.

Borland, R., Wilson, N., Fong, G. T., Hammond, D., Cummings, K. M., Yong, H. H., . . . McNeill, A. (2009). Impact of graphic and text warnings on cigarette packs: Findings from four countries over five years. *Tobacco Control, 18*(5), 358-364.

Chait, L. D., & Griffiths, R. R. (1982). Smoking behavior and tobacco smoke intake: Response of smokers to shortened cigarettes. *Clinical Pharmacology and Therapeutics, 32*(1), 90-97.

Devlin, E., Anderson, S., Hastings, G., & Macfadyen, L. (2005). Targeting smokers via tobacco product labelling: Opportunities and challenges for Pan European health promotion. *Health Promotion International, 20*(1), 41-49.

Fong, G. T., Hammond, D., & Hitchman, S. C. (2009). The impact of pictures on the effectiveness of tobacco warnings. *Bulletin of the World Health Organization, 87*(8), 640-643.

Hammond, D. (2011). Health warning messages on tobacco products: A review. *Tobacco Control, 20*(5), 327-337.

Hammond, D., Fong, G. T., McDonald, P. W., Cameron, R., & Brown, K. S. (2003). Impact of the graphic Canadian warning labels on adult smoking behaviour. *Tobacco Control, 12*(4), 391-395.

Li, J., & Grigg, M. (2009). New Zealand: New graphic warnings encourage registrations with the quitline. *Tobacco Control, 18*(1), 72.

Moodie, C., MacKintosh, A. M., & Hammond, D. (2010). Adolescents' response to text-only tobacco health warnings: Results from the 2008 UK Youth Tobacco Policy Survey. *European Journal of Public Health, 20*(4), 463-469.

Moodie, C., Mackintosh, A. M., Hastings, G., & Ford, A. (2011). Young adult smokers' perceptions of plain packaging: A pilot naturalistic study. *Tobacco Control, 20*(5), 367-373.

Moodie, C., Stead, M., Bauld, L., McNeill, A., Angus, K., Hinds, K., . . . O'Mara-Eves, A. (2012). Plain Tobacco Packaging: A Systematic Review *UK Centre for Tobacco Control Studies*. Stirling: University of Stirling and the Open University.

Rohsenow, D. J., Monti, P. M., Colby, S. M., Gulliver, S. B., & et al. (1997). Effects of alcohol cues on smoking urges and topography among alcoholic men. *Alcoholism: Clinical and Experimental Research, 21*(1), 101-107.

Stockley, C. S. (2001). The effectiveness of strategies such as health warning labels to reduce alcohol-related harms: An Australian perspective. *International Journal of Drug Policy, 12*(2), 153-166.

Wakefield, M., Germain, D., & Henriksen, L. (2008). The effect of retail cigarette pack displays on impulse purchase. *Addiction, 103*(2), 322-328.

Willemsen, M. C. (2005). The new EU cigarette health warnings benefit smokers who want to quit the habit: Results from the Dutch Continuous survey of smoking habits. *European Journal of Public Health, 15*(4), 389-392.

<u>**Included articles by intervention type and behaviour**</u>

AMBIENCE / DIET

Bell, R., Meiselman, H. L., Pierson, B. J., & Reeve, W. G. (1994). Effects of adding an Italian theme to a restaurant on the perceived ethnicity, acceptability, and selection of foods. *Appetite, 22*(1), 11-24.

Bellisle, F., & Dalix, A.-M. (2001). Cognitive restraint can be offset by distraction, leading to increased meal intake in women. *The American Journal of Clinical Nutrition, 74*(2), 197-200.

Bellisle, F., Dalix, A. M., & Slama, G. (2004). Non food-related environmental stimuli induce increased meal intake in healthy women: Comparison of television viewing versus listening to a recorded story in laboratory settings. *Appetite, 43*(2), 175-180.

Bellisle, F., Dalix, A.-M., Airinei, G., Hercberg, S., & Péneau, S. (2009). Influence of dietary restraint and environmental factors on meal size in normal-weight women. A laboratory study. *Appetite, 53*(3), 309-313.

Blass, E. M., Anderson, D. R., Kirkorian, H. L., Pempek, T. A., Price, I., & Koleini, M. F. (2006). On the road to obesity: Television viewing increases intake of high-density foods. *Physiology & Behavior, 88*(4-5), 597-604.

Caldwell, C., & Hibbert, S. A. (1999). Play that one again: The effect of music tempo on consumer behaviour in a restaurant *European Advances in Consumer Research, 4*, 58-62.

Caldwell, C., & Hibbert, S. A. (2002). The influence of music tempo and musical preference on restaurant patrons' behavior. *Psychology and Marketing, 19*(11), 895-917.

Chandon, P., & Wansink, B. (2011). Is food marketing making us fat? A multi-disciplinary review *Faculty & Research Working Paper*. Fountainbleau, France: Instead: The Business School for the World.

Coelho, J. S., Polivy, J., Peter, H. C., & Pliner, P. (2009). Wake up and smell the cookies. Effects of olfactory food-cue exposure in restrained and unrestrained eaters. *Appetite, 52*(2), 517-520.

Fedoroff, I., Polivy, J., & Peter, H. C. (2003). The specificity of restrained versus unrestrained eaters' responses to food cues: General desire to eat, or craving for the cued food? *Appetite, 41*(1), 7-13.

Francis, L. A., & Birch, L. L. (2006). Does eating during television viewing affect preschool children's intake? *Journal of the American Dietetic Association, 106*(4), 598-600.

Gueguen, N., & Petr, C. (2006). Odors and consumer behavior in a restaurant. *International Journal of Hospitality Management, 25*(2), 335-339.

Hetherington, M. M., Anderson, A. S., Norton, G. N., & Newson, L. (2006). Situational effects on meal intake: A comparison of eating alone and eating with others. *Physiology & Behavior, 88*(4-5), 498-505.

Hetherington, M. M. (2007). Cues to overeat: Psychological factors influencing overconsumption. *Proceedings of the Nutrition Society, 66*(1), 113-123.

Higgs, S., & Woodward, M. (2009). Television watching during lunch increases afternoon snack intake of young women. *Appetite, 52*(1), 39-43.

Mathey, M. F. A. M., Vanneste, V. G. G., de Graaf, C., de Groot, L. C. P. G. M., & van Staveren, W. A. (2001). Health effect of improved meal ambiance in a Dutch nursing home: A 1-year intervention study. *Preventive Medicine, 32*(5), 416-423.

McCarron, A., & Tierney, K. J. (1989). The effect of auditory stimulation on the consumption of soft drinks. *Appetite, 13*(2), 155-159.

Milliman, R. E. (1982). Using background music to affect the behavior of supermarket shoppers. *The Journal of Marketing, 46*(3), 86-91.

Milliman, R. E. (1986). The influence of background music on the behavior of restaurant patrons. *Journal of Consumer Research, 13*(2), 286-289.

Moray, J., Fu, A., Brill, K., & Mayoral, M. S. (2007). Viewing television while eating impairs the ability to accurately estimate total amount of food consumed. *Bariatric Nursing and Surgical Patient Care, 2*(1), 71-76.

North, A. C., & Hargreaves, D. J. (1998). The effect of music on atmosphere and purchase intentions in a cafeteria. *Journal of Applied Social Psychology, 28*(24), 2254-2273.

North, A. C., Shilcock, A., & Hargreaves, D. J. (2003). The effect of musical style on restaurant customers' spending. *Environment and Behavior, 35*(5), 712-718.

Peck, J., & Childers, T. L. (2008). If it tastes, smells, sounds, and feels like a duck, then it must be a….: Effects of sensory factors on consumer behaviors. In P. M. Haugtvedt, & F. R. Kardes (Ed.), *Handbook of consumer psychology* (pp. 193-219). New York: Psychology Press.

Peneau, S., Mekhmoukh, A., Chapelot, D., Dalix, A.-M., Airinei, G., Hercberg, S., & Bellisle, F. (2009). Influence of environmental factors on food intake and choice of beverage during meals in teenagers: A laboratory study. *British Journal of Nutrition, 102*(12), 1854-1859.

Poothullil, J. M. (2002). Role of oral sensory signals in determining meal size in lean women. *Nutrition, 18*(6), 479-483.

Quartier, K., Christiaans, H., & Van Cleempoel, K. (2009). *Retail design: Lighting as an atmospheric tool, creating experiences which influence consumers' mood and behaviour in commercial spaces.* Paper presented at the Undisciplined! Design Research Society Conference 2008, Sheffield, UK.

Smith, J. M., & Ditschun, T. L. (2009). Controlling satiety: How environmental factors influence food intake. *Trends in Food Science & Technology, 20*(6-7), 271-277.

Sobal, J., & Wansink, B. (2007). Kitchenscapes, tablescapes, platescapes, and foodscapes. *Environment and Behavior, 39*(1), 124-142.

Spence, C., & Shankar, M. U. (2010). The influence of auditory cues on the perception of, and responses to, food and drink. *Journal of Sensory Studies, 25*(3), 406-430.

Stroebele, N., & de Castro, J. M. (2004). Effect of ambience on food intake and food choice. *Nutrition, 20*(9), 821-838.

Turley, L. W., & Milliman, R. E. (2000). Atmospheric effects on shopping behavior: A review of the experimental evidence. *Journal of Business Research, 49*(2), 193-211.

Wansink, B. (2004). Environmental factors that increase the food intake and consumption volume of unknowing consumers. *Annual Review of Nutrition, 24*, 455-479.

Zampini, M., & Spence, C. (2010). Assessing the role of sound in the perception of food and drink. *Chemosensory Perception, 3*(1), 57-67.

AMBIENCE / PHYSICAL ACTIVITY

Barwood, M. J., Weston, N. J. V., Thelwell, R., & Page, J. (2009). A motivational music and video intervention improves high-intensity exercise performance. *Journal of Sports Science and Medicine, 8*(3), 435-442.

Boutelle, K. N., Jeffery, R. W., Murray, D. M., & Schmitz, M. K. (2001). Using signs, artwork, and music to promote stair use in a public building. *American Journal of Public Health, 91*(12), 2004-2006.

Cardon, G., Labarque, V., Smits, D., & Bourdeaudhuij, I. D. (2009). Promoting physical activity at the pre-school playground: The effects of providing markings and play equipment. *Preventive Medicine, 48*(4), 335-340.

Kerr, N. A., Yore, M. M., Ham, S. A., & Dietz, W. H. (2004). Increasing stair use in a worksite through environmental changes. *American Journal of Health Promotion, 18*(4), 312-315.

Loucaides, C. A., Jago, R., & Charalambous, I. (2009). Promoting physical activity during school break times: Piloting a simple, low cost intervention. *Preventive Medicine, 48*(4), 332-334.

Ridgers, N. D., Stratton, G., Fairclough, S. J., & Twisk, J. W. R. (2007). Long-term effects of a playground markings and physical structures on children's recess physical activity levels. *Preventive Medicine, 44*(5), 393-397.

Rogers, Y., Hazlewood, W. R., Marshall, P., Dalton, N., & Hetrich, S. (2010). Ambient influence: Can twinkly lights lure and abstract representations trigger behavioural change? *UbiComp 2010, 26-29*, 261-270.

Stratton, G., & Mullan, E. (2005). The effect of multicolor playground markings on children's physical activity level during recess. *Preventive Medicine, 41*(5-6), 828-833.

van Nieuw-Amerongen, M. E., Kremers, S. P. J., de Vries, N. K., & Kok, G. (2011). The use of prompts, increased accessibility, visibility, and aesthetics of the stairwell to promote stair use in a university building. *Environment and Behavior, 43*(1), 131-139.

Webb, O. J., & Eves, F. F. (2007). Effects of environmental changes in a stair climbing intervention: generalization to stair descent. *American Journal of Health Promotion, 22*(1), 38-44.

AMBIENCE / ALCOHOL

Areni, C. S., & Kim, D. (1993). The influence of background music on shopping behavior: Classical versus top-forty music in a wine store. *Advances in Consumer Research, 20*, 336-340.

Areni, C. S., & Kim, D. (1994). The influence of in-store lighting on consumers' examination of merchandise in a wine store. *International Journal of Research in Marketing, 11*(2), 117-125.

Gueguen, N., Le Guellec, H., & Jacob, C. (2004). Sound level of background music and alcohol consumption: An empirical evaluation. *Perceptual and Motor Skills, 99*(1), 34-38.

Gueguen, N., & Petr, C. (2006). Odors and consumer behavior in a restaurant. *International Journal of Hospitality Management, 25*(2), 335-339.

Gueguen, N., Jacob, C., Le Guellec, H., Morineau, T., & Lourel, M. (2008). Sound level of environmental music and drinking behavior: A field experiment with beer drinkers. *Alcoholism, Clinical and Experimental Research, 32*(10), 1795-1798.

Hughes, K., Quigg, Z., Eckley, L., Bellis, M., Jones, L., Calafat, A., . . . van, H. (2011). Environmental factors in drinking venues and alcohol-related harm: The evidence base for European intervention. *Addiction, 106*(Suppl.1), 37-46.

Jacob, C. (2006). Styles of background music and consumption in a bar: An empirical evaluation. *International Journal of Hospitality Management, 25*(4), 716-720.

Milliman, R. E. (1986). The influence of background music on the behavior of restaurant patrons. *Journal of Consumer Research, 13*(2), 286-289.

North, A. C., Hargreaves, D. J., & McKendrick, J. (1997). In-store music affects product choice. *Nature, 390*(6656), 132.

North, A. C., Hargreaves, D. J., & McKendrick, J. (1999). The influence of in-store music on wine selections. . *Journal of Applied Psychology, 84*(2), 271-276.

North, A. C., Shilcock, A., & Hargreaves, D. J. (2003). The effect of musical style on restaurant customers' spending. *Environment and Behavior, 35*(5), 712-718.

Spence, C., & Shankar, M. U. (2010). The influence of auditory cues on the perception of, and responses to, food and drink. *Journal of Sensory Studies, 25*(3), 406-430.

Turley, L. W., & Milliman, R. E. (2000). Atmospheric effects on shopping behavior: A review of the experimental evidence. *Journal of Business Research, 49*(2), 193-211.

Zampini, M., & Spence, C. (2010). Assessing the role of sound in the perception of food and drink. *Chemosensory Perception, 3*(1), 57-67.

AVAILABILITY / DIET

Anderson, A. S., Porteous, L. E. G., Foster, E., Higgins, C., Stead, M., Hetherington, M., . . . Adamson, A. J. (2005). The impact of a school-based nutrition education intervention on dietary intake and cognitive and attitudinal variables relating to fruits and vegetables. *Public Health Nutrition, 8*(6), 650-656.

Angelopoulos, P. D., Milionis, H. J., Grammatikaki, E., Moschonis, G., & Manios, Y. (2009). Changes in BMI and blood pressure after a school based intervention: The CHILDREN study. *European Journal of Public Health, 19*(3), 319-325.

Bartholomew, J. B., & Jowers, E. M. (2006). Increasing frequency of lower-fat entrees offered at school lunch: An environmental change strategy to increase healthful selections. *Journal of the American Dietetic Association, 106*(2), 248-252.

Chandon, P., & Wansink, B. (2002). When are stockpiled products consumed faster? A convenience-salience framework of postpurchase consumption incidence and quantity. *Journal of Marketing Research, 39*(3), 321-335.

Chandon, P., & Wansink, B. (2011). Is food marketing making us fat? A multi-disciplinary review *Faculty & Research Working Paper*. Fountainbleau, France: Instead: The Business School for the World.

Engbers, L. H., van Poppel, M. N. M., Chin A Paw, M. J. M., & van Mechelen, W. (2005). Worksite health promotion programs with environmental changes: A systematic review. *American Journal of Preventive Medicine, 29*(1), 61-70.

Fiske, A., & Cullen, K. W. (2004). Effects of promotional materials on vending sales of low-fat items in teachers' lounges. *Journal of the American Dietetic Association, 104*(1), 90-93.

French, S. A., Story, M., Fulkerson, J. A., & Hannan, P. (2004). An environmental intervention to promote lower-fat food choices in secondary schools: Outcomes of the TACOS study. *American Journal of Public Health, 94*(9), 1507-1512.

French, S. A., Hannan, P. J., Harnack, L. J., Mitchell, N. R., Toomey, T. L., & Gerlach, A. (2010). Pricing and availability intervention in vending machines at four bus garages. *Journal of Occupational and Environmental Medicine, 52* (Suppl.1), s29-s33.

Glanz, K., & Hoelscher, D. (2004). Increasing fruit and vegetable intake by changing environments, policy and pricing: Restaurant-based research, strategies, and recommendations. *Preventive Medicine, 39* (Suppl.2), s88-s93.

Glanz, K., & Mullis, R. M. (1988). Environmental interventions to promote healthy eating: A review of models, programs, and evidence. *Health Education & Behavior, 15*(4), 395-415.

Goldberg, M. E., & Gunasti, K. (2007). Creating an environment in which youths are encouraged to eat a healthier diet. *Journal of Public Policy & Marketing, 26*(2), 162-181.

Jeffery, R. W., French, S. A., Raether, C., & Baxter, J. E. (1994). An environmental intervention to increase fruit and salad purchases in a cafeteria. *Preventive Medicine, 23*(6), 788-792.

Kimathi, A. N., Gregoire, M. B., Dowling, R. A., & Stone, M. K. (2009). A healthful options food station can improve satisfaction and generate gross profit in a worksite cafeteria. *Journal of the American Dietetic Association 109*(5), 914-917.

Lowe, M. R., Tappe, K. A., Butryn, M. L., Annunziato, R. A., Coletta, M. C., Ochner, C. N., & Rolls, B. J. (2010). An intervention study targeting energy and nutrient intake in worksite cafeterias. *Eating Behaviors, 11*(3), 144-151.

Patrick, H., & Nicklas, T. A. (2005). A review of family and social determinants of children's eating patterns and diet quality. *Journal of the American College of Nutrition, 24*(2), 83-92.

Perlmutter, C. A., Canter, D. D., & Gregoire, M. B. (1997). Profitability and acceptability of fat- and sodium-modified hot entrees in a worksite cafeteria. *Journal of the American Dietetic Association, 97*(4), 391-395.

Perry, C. L., Bishop, D. B., Taylor, G. L., Davis, M., Story, M., Gray, C., . . . Harnack, L. (2004). A randomized school trial of environmental strategies to encourage fruit and vegetable consumption among children. *Health Education & Behavior, 31*(1), 65-76.

Richard, L., O'Loughlin, J., Masson, P., & Devost, S. (1999). Healthy menu intervention in restaurants in low-income neighbourhoods: A field experience. *Journal of Nutrition Education, 31*(1), 54-59.

Seymour, J. D., Yaroch, A. L., Serdula, M., Blanck, H. M., & Khan, L. K. (2004). Impact of nutrition environmental interventions on point-of-purchase behavior in adults: A review. *Preventive Medicine, 39* (Suppl.2), s108-s136.

Shepherd, J., Harden, A., Rees, R., Brunton, G., Garcia, J., Oliver, S., & Oakley, A. (2006). Young people and healthy eating: A systematic review of research on barriers and facilitators. *Health Education Research, 21*(2), 239-257.

Sobal, J., & Wansink, B. (2007). Kitchenscapes, tablescapes, platescapes, and foodscapes. *Environment and Behavior, 39*(1), 124-142.

Sorensen, G., Linnan, L., & Hunt, M. K. (2004). Worksite-based research and initiatives to increase fruit and vegetable consumption. *Preventive Medicine, 39* (Suppl.2), s94-s100.

Steenhuis, I., van Assema, P., van Breukelen, G., Glanz, K., Kok, G., & de Vries, H. (2004). The impact of educational and environmental interventions in Dutch worksite cafeterias. *Health Promotion International, 19*(3), 335-343.

Wansink, B. (2004). Environmental factors that increase the food intake and consumption volume of unknowing consumers. *Annual Review of Nutrition, 24*, 455-479.

Whitaker, R. C., Wright, J. A., Finch, A. J., & Psaty, B. M. (1993). An environmental intervention to reduce dietary fat in school lunches. *Pediatrics, 91*(6), 1107-1111.

Wilbur, C. S., Zifferblatt, S. M., Pinsky, J. L., & Zifferblatt, S. (1981). Healthy vending: A cooperative pilot research program to stimulate good health in the marketplace. *Preventive Medicine, 10*(1), 85-93.

Wilcox, K., Vallen, B., Block, L., & Fitzsimons, G. J. (2009). Vicarious goal fulfillment: When the mere presence of a healthy option leads to an ironically indulgent decision. *Journal of Consumer Research, 36*(3), 380-393.

AVAILABILITY / PHYSICAL ACTIVITY

Engbers, L. H., van Poppel, M. N. M., Chin A Paw, M. J. M., & van Mechelen, W. (2005). Worksite health promotion programs with environmental changes: A systematic review. *American Journal of Preventive Medicine, 29*(1), 61-70.

Eves, F. F., Lewis, A. L., & Griffin, C. (2008). Modelling effects of stair width on rates of stair climbing in a train station. *Preventive Medicine, 47*(3), 270-272.

Faskunger, J., Poortvliet, E., Nylund, K., & Rossen, J. (2003). Effect of an environmental barrier to physical activity on commuter stair use. *Scandinavian Journal of Nutrition [Naringsforskning], 47*(1), 26-28.

Nicoll, G., & Zimring, C. (2009). Effect of innovative building design on physical activity. *Journal of Public Health Policy, 30* (Suppl.1), s111-s123.

Olander, E. K., & Eves, F. F. (2011). Elevator availability and its impact on stair use in a workplace. *Journal of Environmental Psychology, 31*(2), 200-206.

van Houten, R. V., Nau, P. A., & Merrigan, M. (1981). Reducing elevator energy use: A comparison of posted feedback and reduced elevator convenience. *Journal of Applied Behavior Analysis, 14*(4), 377-387.

FUNCTIONAL DESIGN / DIET

Ahn, H. J., Han, K. A., Kwon, H. R., & Min, K. W. (2010). The small rice bowl-based meal plan was effective at reducing dietary energy intake, body weight, and blood glucose levels in Korean women with type 2 diabetes mellitus. *Korean Diabetes Journal, 34*(6), 340-349.

Andrade, A. M., Greene, G. W., & Melanson, K. J. (2008). Eating slowly led to decreases in energy intake within meals in healthy women. *Journal of the American Dietetic Association, 108*(7), 1186-1191.

Bohnert, A. M., Randall, E. T., Tharp, S., & Germann, J. (2011). The development and evaluation of a portion plate for youth: A pilot study. *Journal of Nutrition Education & Behavior, 43*(4), 268-273.

Chandon, P., & Wansink, B. (2011). Is food marketing making us fat? A multi-disciplinary review *Faculty & Research Working Paper*. Fountainbleau, France: Instead: The Business School for the World.

Engbers, L. H., van Poppel, M. N. M., Chin A Paw, M. J. M., & van Mechelen, W. (2005). Worksite health promotion programs with environmental changes: A systematic review. *American Journal of Preventive Medicine, 29*(1), 61-70.

Geier, A. B., Rozin, P., & Doros, G. (2006). Unit bias. A new heuristic that helps explain the effect of portion size on food intake. *Psychological Science, 17*(6), 521-525.

Goldberg, M. E., & Gunasti, K. (2007). Creating an environment in which youths are encouraged to eat a healthier diet. *Journal of Public Policy & Marketing, 26*(2), 162-181.

Hogenkamp, P. S., Mars, M., Stafleu, A., & de, G. (2010). Intake during repeated exposure to low- and high-energy-dense yogurts by different means of consumption. *American Journal of Clinical Nutrition, 91*(4), 841-847.

Just, D., & Wansink, B. (2009). Smarter lunchrooms: Using behavioral economics to improve meal selection. *Choices, 29*(3).

Kalnikaitė, V., Yvonne, R., Bird, J., Villar, N., Bachour, K., Payne, S., . . . Krüger, A. (2011). *How to nudge in situ: Designing lambent devices to deliver information salience in supermarkets.* Paper presented at the 13th ACM International conference on Ubiquitous computing, New York, NY, United States.

Koh, J., & Pliner, P. (2009). The effects of degree of acquaintance, plate size, and sharing on food intake. *Appetite, 52*(3), 595-602.

Lombardini-Riipinen, C., & Lankoski, L. (2010). *Take off the heater: Utility effect and food environment effect in food consumption decisions.* Paper presented at the 1st Joint EAAE/AAEA Seminar, Munich, Germany.

Marchiori, D., Corneille, O., & Klein, O. (2012). Container size influences snack food intake independently of portion size. *Appetite, 58*(3), 814-817.

Mishra, A., Mishra, H., & Masters, T. M. (2011). The influence of bite-size on quantity of food consumed: A field study. *Journal of Consumer Research*.

Rolls, B. J., Roe, L. S., Halverson, K. H., & Meengs, J. S. (2007). Using a smaller plate did not reduce energy intake at meals. *Appetite, 49*(3), 652-660.

Rozin, P., Scott, S., Dingley, M., Urbanek, J. K., Jiang, H., & Kaltenbach, M. (2011). Nudge to nobesity I: Minor changes in accessibility decrease food intake. *Judgment and Decision Making, 6*(4), 323-332.

Shah, M., Schroeder, R., Winn, W., & Adams-Huet, B. (2011). A pilot study to investigate the effect of plate size on meal energy intake in normal weight and overweight/obese women. *Journal of Human Nutrition and Dietetics, 24*(6), 612-615.

Smith, J. M., & Ditschun, T. L. (2009). Controlling satiety: How environmental factors influence food intake. *Trends in Food Science & Technology, 20*(6-7), 271-277.

Sobal, J., & Wansink, B. (2007). Kitchenscapes, tablescapes, platescapes, and foodscapes. *Environment and Behavior, 39*(1), 124-142.

Stroebele, N., & de Castro, J. M. (2004). Effect of ambience on food intake and food choice. *Nutrition, 20*(9), 821-838.

Van den Bergh, B., Schmitt, J., & Warlop, L. (2011). Embodied Myopia. *Journal of Marketing Research, 48*(6), 1033-1044.

van Kleef, E., Shimizu, M., & Wansink, B. (2011). Serving bowl selection biases the amount of food served. *Journal of Nutrition Education and Behavior, 44*(1), 66-70.

Wansink, B., & van Ittersum, K. (2003). Bottoms up! The influence of elongation on pouring and consumption volume. *Journal of Consumer Research, 30*(3), 455-463.

Wansink, B. (2004). Environmental factors that increase the food intake and consumption volume of unknowing consumers. *Annual Review of Nutrition, 24*, 455-479.

Wansink, B., & Cheney, M. M. (2005). Super bowls: Serving bowl size and food consumption. *Journal of the American Medical Association, 293*(14), 1727-1728.

Wansink, B., van Ittersum, K., & Painter, J. E. (2006). Ice cream illusions: Bowls, spoons, and self-served portion sizes. *American Journal of Preventive Medicine, 31*(3), 240-243.

Wansink, B. (2010). From mindless eating to mindlessly eating better. *Physiology & Behavior, 100*(5), 454-463.

FUNCTIONAL DESIGN / PHYSICAL ACTIVITY

Benden, M. E., Blake, J. J., Wendel, M. L., & Huber, J. C. (2011). The impact of stand-biased desks in classrooms on calorie expenditure in children. *American Journal of Public Health, 101*(8), 1433-1436.

Cardon, G., Labarque, V., Smits, D., & Bourdeaudhuij, I. D. (2009). Promoting physical activity at the pre-school playground: The effects of providing markings and play equipment. *Preventive Medicine, 48*(4), 335-340.

Engbers, L. H., van Poppel, M. N. M., Chin A Paw, M. J. M., & van Mechelen, W. (2005). Worksite health promotion programs with environmental changes: A systematic review. *American Journal of Preventive Medicine, 29*(1), 61-70.

Jenum, A. K., Lorentzen, C. A. N., & Ommundsen, Y. (2009). Targeting physical activity in a low socioeconomic status population: Observations from the Norwegian "Romsas in Motion" study. *British Journal of Sports Medicine, 43*(1), 64-69.

Lanningham-Foster, L., Foster, R. C., McCrady, S. K., Manohar, C. U., Jensen, T. B., Mitre, N. G., . . . Levine, J. A. (2008). Changing the school environment to increase physical activity in children. *Obesity (Silver Spring), 16*(8), 1849-1853.

Loucaides, C. A., Jago, R., & Charalambous, I. (2009). Promoting physical activity during school break times: Piloting a simple, low cost intervention. *Preventive Medicine, 48*(4), 332-334.

Ridgers, N., Stratton, G., Fairclough, S., & Twisk, J. (2007). Children's physical activity levels during school recess: A quasi-experimental intervention study. *International Journal of Behavioral Nutrition and Physical Activity, 4*(1), 19.

Ridgers, N. D., Stratton, G., Fairclough, S. J., & Twisk, J. W. R. (2007). Long-term effects of a playground markings and physical structures on children's recess physical activity levels. *Preventive Medicine, 44*(5), 393-397.

Ridgers, N. D., Fairclough, S. J., & Stratton, G. (2010). Twelve-month effects of a playground intervention on children's morning and lunchtime recess physical activity levels. *Journal of Physical Activity & Health, 7*(2), 167-175.

Stratton, G., & Mullan, E. (2005). The effect of multicolor playground markings on children's physical activity level during recess. *Preventive Medicine, 41*(5-6), 828-833.

Thompson, W. G., Foster, R. C., Eide, D. S., & Levine, J. A. (2008). Feasibility of a walking workstation to increase daily walking. *British Journal of Sports Medicine, 42*(3), 225-228.

FUNCTIONAL DESIGN / ALCOHOL

Hughes, K., Quigg, Z., Eckley, L., Bellis, M., Jones, L., Calafat, A., . . . van, H. (2011). Environmental factors in drinking venues and alcohol-related harm: The evidence base for European intervention. *Addiction, 106*(Suppl.1), 37-46.

Wansink, B., & van Ittersum, K. (2003). Bottoms up! The influence of elongation on pouring and consumption volume. *Journal of Consumer Research, 30*(3), 455-463.

Wansink, B. (2004). Environmental factors that increase the food intake and consumption volume of unknowing consumers. *Annual Review of Nutrition, 24*, 455-479.

Wansink, B., & van Ittersum, K. (2005). Shape of glass and amount of alcohol poured: Comparative study of effect of practice and concentration. *British Medical Journal, 331*(7531), 1512-1514.

Wansink, B. (2010). From mindless eating to mindlessly eating better. *Physiology & Behavior, 100*(5), 454-463.

LABELLING / DIET

Antonuk, B., & Block, L. G. (2006). The effect of single serving versus entire package nutritional information on consumption norms and actual consumption of a snack food. *Journal of Nutrition Education and Behavior, 38*(6), 365-370.

Aron, J. I., Evans, R. E., & Mela, D. J. (1995). Paradoxical effect of a nutrition labelling scheme in a student cafeteria. *Nutrition Research, 15*(9), 1251-1261.

Bassett, M. T., Dumanovsky, T., Huang, C., Silver, L. D., Young, C., Nonas, C., . . . Frieden, T. R. (2008). Purchasing behavior and calorie information at fast-food chains in New York City, 2007. *American Journal of Public Health, 98*(8), 1457-1459.

Bergen, D., & Yeh, M. C. (2006). Effects of energy-content labels and motivational posters on sales of sugar-sweetened beverages: Stimulating sales of diet drinks among adults study. *Journal of the American Dietetic Association, 106*(11), 1866-1869.

Bollinger, B., Leslie, P., & Sorensen, A. (2011). Calorie posting in chain restaurants. *American Economic Journal: Economic Policy, 3*(1), 91-128.

Borgmeier, I., & Westenhoefer, J. (2009). Impact of different food label formats on healthiness evaluation and food choice of consumers: A randomized-controlled study. *BMC Public Health, 9*, 184.

Burton, S., Creyer, E. H., Kees, J., & Huggins, K. (2006). Attacking the obesity epidemic: The potential health benefits of providing nutrition information in restaurants. *American Journal of Public Health, 96*(9), 1669-1675.

Chandon, P., & Wansink, B. (2011). Is food marketing making us fat? A multi-disciplinary review *Faculty & Research Working Paper*. Fountainbleau, France: Instead: The Business School for the World.

Chu, Y. H., Frongillo, E. A., Jones, S. J., & Kaye, G. L. (2009). Improving patrons' meal selections through the use of point-of-selection nutrition labels. *American Journal of Public Health, 99*(11), 2001-2005.

Cinciripini, P. M. (1984). Changing food selections in a public cafeteria. *Behavior Modification, 8*(4), 520-539.

Dixon, H., Scully, M., Wakefield, M., Kelly, B., Chapman, K., & Donovan, R. (2011). Parent's responses to nutrient claims and sports celebrity endorsements on energy-dense and nutrient-poor foods: An experimental study. *Public Health Nutrition, 14*(6), 1071-1079.

Dubbert, P. M., Johnson, W. G., Schlundt, D. G., & Montague, N. W. (1984). The influence of caloric information on cafeteria food choices. *Journal of Applied Behavior Analysis, 17*(1), 85-92.

Dumanovsky, T., Huang, C. Y., Nonas, C. A., Matte, T. D., Bassett, M. T., & Silver, L. D. (2011). Changes in energy content of lunchtime purchases from fast food restaurants after introduction of calorie labelling: Cross sectional customer surveys. *British Medical Journal, 343*.

Elbel, B., Kersh, R., Brescoll, V. L., & Dixon, L. B. (2009). Calorie labeling and food choices: A first look at the effects on low-income people In New York City. *Health Affairs, 28*(6), w1110-w1121.

Elbel, B., Gyamfi, J., & Kersh, R. (2011). Child and adolescent fast-food choice and the influence of calorie labeling: A natural experiment. *International Journal of Obesity, 35*(4), 493-500.

Engbers, L. H., van Poppel, M. N. M., Chin A Paw, M. J. M., & van Mechelen, W. (2005). Worksite health promotion programs with environmental changes: A systematic review. *American Journal of Preventive Medicine, 29*(1), 61-70.

Engbers, L. H., van Poppel, M. N., Chin, A. P. M., & van Mechelen, W. (2006). The effects of a controlled worksite environmental intervention on determinants of dietary behavior and self-reported fruit, vegetable and fat intake. *BMC Public Health, 6*, 253.

Finkelstein, E. A., Strombotne, K. L., Chan, N. L., & Krieger, J. (2011). Mandatory menu labeling in one fast-food chain in King County, Washington. *American Journal of Preventive Medicine, 40*(2), 122-127.

Freedman, M. R., & Connors, R. (2010). Point-of-purchase nutrition information influences food-purchasing behaviors of college students: A pilot study. *Journal of the American Dietetic Association, 110*(8), 1222-1226.

French, S. A., Jeffery, R. W., Story, M., Breitlow, K. K., Baxter, J. S., Hannan, P., & Snyder, M. P. (2001). Pricing and promotion effects on low-fat vending snack purchases: The CHIPS Study. *American Journal of Public Health, 91*(1), 112-127.

Garg, N., Wansink, B., & Inman, J. J. (2007). The influence of incidental affect on consumers' food intake. *Journal of Marketing, 71*(1), 194-206.

Gerend, M. A. (2009). Does calorie information promote lower calorie fast food choices among college students? *Journal of Adolescent Health, 44*(1), 84-86.

Giesen, J. C., Payne, C. R., Havermans, R. C., & Jansen, A. (2011). Exploring how calorie information and taxes on high-calorie foods influence lunch decisions. *The American Journal of Clinical Nutrition, 93*(4), 689-694.

Gittelsohn, J., Vijayadeva, V., Davison, N., Ramirez, V., Cheung, L. W., Murphy, S., & Novotny, R. (2010). A food store intervention trial improves caregiver psychosocial factors and children's dietary intake in Hawaii. *Obesity (Silver Spring), 18* (Suppl.1), s84-s90.

Glanz, K., & Mullis, R. M. (1988). Environmental interventions to promote healthy eating: A review of models, programs, and evidence. *Health Education & Behavior, 15*(4), 395-415.

Glanz, K., & Hoelscher, D. (2004). Increasing fruit and vegetable intake by changing environments, policy and pricing: Restaurant-based research, strategies, and recommendations. *Preventive Medicine, 39* (Suppl.2), s88-s93.

Goldberg, M. E., & Gunasti, K. (2007). Creating an environment in which youths are encouraged to eat a healthier diet. *Journal of Public Policy & Marketing, 26*(2), 162-181.

Harnack, L. J., & French, S. A. (2008). Effect of point-of-purchase calorie labeling on restaurant and cafeteria food choices: A review of the literature. *International Journal of Behavioral Nutriton and Physical Activity, 5*, 51.

Harnack, L. J., French, S. A., Oakes, J. M., Story, M. T., Jeffery, R. W., & Rydell, S. A. (2008). Effects of calorie labeling and value size pricing on fast food meal choices: Results from an experimental trial. *International Journal of Behavioral Nutrition and Physical Activity, 5*, 63.

Hassan, L. M., Shiu, E. M. K., & Michaelidou, N. (2010). The influence of nutrition information on choice: The roles of temptation, conflict and self-control. *Journal of Consumer Affairs, 44*(3), 499-515.

Hoefkens, C., Lachat, C., Kolsteren, P., Van Camp, J., & Verbeke, W. (2011). Posting point-of-purchase nutrition information in university canteens does not influence meal choice and nutrient intake. *American Journal of Clinical Nutrition, 94*(2), 562-570.

Horgen, K. B., & Brownell, K. D. (2002). Comparison of price change and health message interventions in promoting healthy food choices. *Health Psychology, 21*(5), 505-512.

Howlett, E. A., Burton, S., Bates, K., & Huggins, K. (2009). Coming to a restaurant near you? Potential consumer responses to nutrition information disclosure on menus. *Journal of Consumer Research, 36*(3), 494-503.

Hrovat, K. B., Harris, K. Z., Leach, A. D., Russell, B. S., Harris, B. V., & Sprecher, D. L. (1994). The new food label, type of fat, and consumer choice. A pilot study. *Archives of Family Medicine, 3*(8), 690-695.

Kiesel, K., & Villas-Boas, S. B. (2009). Can information costs affect consumer choice? Nutritional labels in a supermarket experiment *Working Paper No.1060R2, International Journal of Industrial Organization*. Berkeley, CA: Department of Agricultural and Resource Economics, University of California, Berkeley.

Kral, T. V., Roe, L. S., & Rolls, B. J. (2002). Does nutrition information about the energy density of meals affect food intake in normal-weight women? *Appetite, 39*(2), 137-145.

Lin, C.-H., Lin, H.-C., & Lee, S.-H. (2011). The influence of health-related information on variety-seeking behavior: The moderating roles of mood states and gender. *British Food Journal, 113*(11).

Lowe, M. R., Tappe, K. A., Butryn, M. L., Annunziato, R. A., Coletta, M. C., Ochner, C. N., & Rolls, B. J. (2010). An intervention study targeting energy and nutrient intake in worksite cafeterias. *Eating Behaviors, 11*(3), 144-151.

Mathios, A. D. (2000). The impact of mandatory disclosure laws on product choices: An analysis of the salad dressing market. *Journal of Law and Economics, 43*(2), 651-677.

Mayer, J. A., Heins, J. M., Vogel, J. M., Morrison, D. C., Lankester, L. D., & Jacobs, A. L. (1986). Promoting low-fat entree choices in a public cafeteria. *Journal of Applied Behavior Analysis, 19*(4), 397-402.

Mayer, J. A., Dubbert, P. M., & Elder, J. P. (1989). Promoting nutrition at the point of choice: A review. *Health Education Quarterly, 16*(1), 31-43.

Miller, D. L., Castellanos, V. H., Shide, D. J., Peters, J. C., & Rolls, B. J. (1998). Effect of fat-free potato chips with and without nutrition labels on fat and energy intakes. *American Journal of Clinical Nutrition, 68*(2), 282-290.

Muller, T. E. (1985). Structural information factors which stimulate the use of nutrition information: A field experiment. *Journal of Marketing Research, 22*(2), 143-157.

Perlmutter, C. A., Canter, D. D., & Gregoire, M. B. (1997). Profitability and acceptability of fat- and sodium-modified hot entrees in a worksite cafeteria. *Journal of the American Dietetic Association, 97*(4), 391-395.

Peterson, S., Duncan, D. P., Null, D. B., Roth, S. L., & Gill, L. (2010). Positive changes in perceptions and selections of healthful foods by college students after a short-term point-of-selection intervention at a dining hall. *Journal of American College Health, 58*(5), 425-431.

Pulos, E., & Leng, K. (2010). Evaluation of a voluntary menu-labeling program in full-service restaurants. *American Journal of Public Health, 100*(6), 1035-1039.

Roberto, C. A., Larsen, P. D., Agnew, H., Baik, J., & Brownell, K. D. (2010). Evaluating the impact of menu labeling on food choices and intake. *American Journal of Public Health, 100*(2), 312-318.

Roefs, A., & Jansen, A. (2004). The effect of information about fat content on food consumption in overweight/obese and lean people. *Appetite, 43*(3), 319-322.

Russo, J., Staelin, R., Nolan, C. A., Russell, G. J., & Metcalf, B. L. (1986). Nutrition information in the supermarket. *Journal of Consumer Research, 13*(1), 48-70.

Sacks, G., Rayner, M., & Swinburn, B. (2009). Impact of front-of-pack 'traffic-light' nutrition labelling on consumer food purchases in the UK. *Health Promotion International, 24*(4), 344-352.

Sacks, G., Tikellis, K., Millar, L., & Swinburn, B. (2011). Impact of 'traffic-light' nutrition information on online food purchases in Australia. *Australian and New Zealand Journal of Public Health, 35*(2), 122-126.

Seymour, J. D., Yaroch, A. L., Serdula, M., Blanck, H. M., & Khan, L. K. (2004). Impact of nutrition environmental interventions on point-of-purchase behavior in adults: A review. *Preventive Medicine, 39* (Suppl.2), s108-s136.

Sharma, S., Wagle, A., Sucher, K., & Bugwadia, N. (2011). Impact of point of selection nutrition information on meal choices at a table-service restaurant. *Journal of Foodservice Business Research, 14*(2), 146-161.

Shide, D. J., & Rolls, B. J. (1995). Information about the fat content of preloads influences energy intake in healthy women. *Journal of the American Dietetic Association, 95*(9), 993-998.

Sorensen, G., Linnan, L., & Hunt, M. K. (2004). Worksite-based research and initiatives to increase fruit and vegetable consumption. *Preventive Medicine, 39* (Suppl.2), s94-s100.

Steenhuis, I., van Assema, P., van Breukelen, G., Glanz, K., Kok, G., & de Vries, H. (2004). The impact of educational and environmental interventions in Dutch worksite cafeterias. *Health Promotion International, 19*(3), 335-343.

Steenhuis, I. H. M., Kroeze, W., Vyth, E. L., Valk, S., Verbauwen, R., & Seidell, J. C. (2010). The effects of using a nutrition logo on consumption and product evaluation of a sweet pastry. *Appetite, 55*(3), 707-709.

Sutherland, L. A., Kaley, L. A., & Fischer, L. (2010). Guiding stars: The effect of a nutrition navigation program on consumer purchases at the supermarket. *American Journal of Clinical Nutrition, 91*(4), s1090-s1094.

Tandon, P. S., Wright, J., Zhou, C., Rogers, C. B., & Christakis, D. A. (2010). Nutrition menu labeling may lead to lower-calorie restaurant meal choices for children. *Pediatrics, 125*(2), 244-248.

Tandon, P. S., Zhou, C., Chan, N. L., Lozano, P., Couch, S. C., Glanz, K., . . . Saelens, B. E. (2011). The impact of menu labeling on fast-food purchases for children and parents. *American Journal of Preventive Medicine, 41*(4), 434-438.

Temple, J. L., Johnson, K., Recupero, K., & Suders, H. (2010). Nutrition labels decrease energy intake in adults consuming lunch in the laboratory. *Journal of the American Dietetic Association, 110*(7), 1094-1097.

Temple, J. L., Johnson, K. M., Archer, K., Lacarte, A., Yi, C., & Epstein, L. H. (2011). Influence of simplified nutrition labeling and taxation on laboratory energy intake in adults. *Appetite, 57*(1), 184-192.

Thorndike, A. N., Sonnenberg, L., Riis, J., Barraclough, S., & Levy, D. E. (2012). A 2-Phase labeling and choice architecture intervention to improve healthy food and beverage choices. *American Journal of Public Health, 102*(3), 527-533.

Ueland, Ø., Cardello, A. V., Merrill, E. P., & Lesher, L. L. (2009). Effect of portion size information on food intake. *Journal of the American Dietetic Association, 109*(1), 124-127.

Vadiveloo, M. K., Dixon, L. B., & Elbel, B. (2011). Consumer purchasing patterns in response to calorie labeling legislation in New York City. *International Journal of Behavioral Nutrition and Physical Activity, 8*, 51.

van Herpen, E., & Trijp, H. C. (2011). Front-of-pack nutrition labels. Their effect on attention and choices when consumers have varying goals and time constraints. *Appetite, 57*(1), 148-160.

Variyam, J. N. (2008). Do nutrition labels improve dietary outcomes? *Health Economics, 17*(6), 695-708.

Vermeer, W. M., Steenhuis, I. H., Leeuwis, F. H., Bos, A. E., de Boer, M., & Seidell, J. C. (2010). Portion size labeling and intended soft drink consumption: The impact of labeling format and size portfolio. *Journal of Nutrition Education and Behavior, 42*(6), 422-426.

Vermeer, W. M., Steenhuis, I. H. M., Leeuwis, F. H., Bos, A. E. R., de Boer, M., & Seidell, J. C. (2011). View the label before you view the movie: A field experiment into the impact of portion size and guideline daily amounts labelling on soft drinks in cinemas. *BMC Public Health, 11*, 438.

Vyth, E. L., Steenhuis, I. H., Vlot, J. A., Wulp, A., Hogenes, M. G., Looije, D. H., . . . Seidell, J. C. (2010). Actual use of a front-of-pack nutrition logo in the supermarket: Consumers' motives in food choice. *Public Health Nutrition, 13*(11), 1882-1889.

Vyth, E. L., Steenhuis, I. H. M., Heymans, M. W., Roodenburg, A. J. C., Brug, J., & Seidell, J. C. (2011). Influence of placement of a nutrition logo on cafeteria menu items on lunchtime food choices at Dutch work sites. *Journal of the American Dietetic Association, 111*(1), 131-136.

Wansink, B., & Chandon, P. (2006). Can "low-fat" nutrition labels lead to obesity? *Journal of Marketing Research, 43*, 605-617.

Wardle, J., & Solomons, W. (1994). Naughty but nice: A laboratory study of health information and food preferences in a community sample. *Health Psychology, 13*(2), 180-183.

Whitaker, R. C., Wright, J. A., Koepsell, T. D., Finch, A. J., & Psaty, B. M. (1994). Randomized intervention to increase children's selection of low-fat foods in school lunches. *Journal of Pediatrics, 125*(4), 535-540.

Wilbur, C. S., Zifferblatt, S. M., Pinsky, J. L., & Zifferblatt, S. (1981). Healthy vending: A cooperative pilot research program to stimulate good health in the marketplace. *Preventive Medicine, 10*(1), 85-93.

Wisdom, J., Downs, J. S., & Loewenstein, G. (2010). Promoting healthy choices: Information versus convenience. *American Economic Journal: Applied Economics, 2*(2), 164-178.

Yamamoto, J. A., Yamamoto, J. B., Yamamoto, B. E., & Yamamoto, L. G. (2005). Adolescent fast food and restaurant ordering behavior with and without calorie and fat content menu information. *Journal of Adolescent Health, 37*(5), 397-402.

Yeomans, M. R., Lartamo, S., Procter, E. L., Lee, M. D., & Gray, R. W. (2001). The actual, but not labelled, fat content of a soup preload alters short-term appetite in healthy men. *Physiology & Behavior, 73*(4), 533-540.

LABELLING / ALCOHOL

Bailey, J., Poole, R., Zinovieff, F., Robinson, C. A., Parry, O., Tocque, K., & Kennedy, L. (2011). Achieving positive change in the drinking culture of Wales *Research report*. Wrexham and Bangor, Wales: Glyndwr University / Bangor University.

Jones, S. C., & Gregory, P. (2009). The impact of more visible standard drink labelling on youth alcohol consumption: Helping young people drink (ir)responsibly? *Drug and Alcohol Review, 28*(3), 230-234.

Kelly-Weeder, S., Phillips, K., & Rounseville, S. (2011). Effectiveness of public health programs for decreasing alcohol consumption. *Patient Intelligence, 2011*(3), 29-38.

MacKinnon, D. P., Pentz, M. A., & Stacy, A. W. (1993). The alcohol warning label and adolescents: The first year. *American Journal of Public Health, 83*(4), 585-587.

Stockwell, T. (2006). A review of research into the impacts of alcohol warning labels on attitudes and behaviour *Centre of Addictions Research of BC*. Victoria, BC: Centre for Addictions Research of British Columbia.

Stockley, C. S. (2001). The effectiveness of strategies such as health warning labels to reduce alcohol-related harms: An Australian perspective. *International Journal of Drug Policy, 12*(2), 153-166.

Wilkinson, C., & Room, R. (2009). Warnings on alcohol containers and advertisements: International experience and evidence on effects. *Drug and Alcohol Review, 28*(4), 426-435.

LABELLING / TOBACCO

Borland, R. (1997). Tobacco health warnings and smoking-related cognitions and behaviours. *Addiction, 92*(11), 1427-1435.

Borland, R., Wilson, N., Fong, G. T., Hammond, D., Cummings, K. M., Yong, H. H., . . . McNeill, A. (2009). Impact of graphic and text warnings on cigarette packs: Findings from four countries over five years. *Tobacco Control, 18*(5), 358-364.

Devlin, E., Anderson, S., Hastings, G., & Macfadyen, L. (2005). Targeting smokers via tobacco product labelling: Opportunities and challenges for Pan European health promotion. *Health Promotion International, 20*(1), 41-49.

Fong, G. T., Hammond, D., & Hitchman, S. C. (2009). The impact of pictures on the effectiveness of tobacco warnings. *Bulletin of the World Health Organization, 87*(8), 640-643.

Hammond, D., Fong, G. T., McDonald, P. W., Cameron, R., & Brown, K. S. (2003). Impact of the graphic Canadian warning labels on adult smoking behaviour. *Tobacco Control, 12*(4), 391-395.

Hammond, D. (2011). Health warning messages on tobacco products: A review. *Tobacco Control, 20*(5), 327-337.

Li, J., & Grigg, M. (2009). New Zealand: New graphic warnings encourage registrations with the quitline. *Tobacco Control, 18*(1), 72.

Moodie, C., MacKintosh, A. M., & Hammond, D. (2010). Adolescents' response to text-only tobacco health warnings: Results from the 2008 UK Youth Tobacco Policy Survey. *European Journal of Public Health, 20*(4), 463-469.

Stockley, C. S. (2001). The effectiveness of strategies such as health warning labels to reduce alcohol-related harms: An Australian perspective. *International Journal of Drug Policy, 12*(2), 153-166.

Willemsen, M. C. (2005). The new EU cigarette health warnings benefit smokers who want to quit the habit: Results from the Dutch Continuous survey of smoking habits. *European Journal of Public Health, 15*(4), 389-392.

PRESENTATION / DIET

Branen, L., Fletcher, J., & Hilbert, L. (2002). Snack consumption and waste by preschool children served "cute" versus regular snacks. *Journal of Nutrition Education and Behavior, 34*(5), 279-282.

Brondel, L., Romer, M., Van Wymelbeke, V., Pineau, N., Jiang, T., Hanus, C., & Rigaud, D. (2009). Variety enhances food intake in humans: Role of sensory-specific satiety. *Physiology & Behavior, 97*(1), 44-51.

Chandon, P., & Wansink, B. (2011). Is food marketing making us fat? A multi-disciplinary review *Faculty & Research Working Paper*. Fountainbleau, France: Instead: The Business School for the World.

Forman, J., Halford, J. C. G., Summe, H., MacDougall, M., & Keller, K. L. (2009). Food branding influences ad libitum intake differently in children depending on weight status. Results of a pilot study. *Appetite, 53*(1), 76-83.

Gosnell, B. A., Mitchell, J. E., Lancaster, K. L., Burgard, M. A., Wonderlich, S. A., & Crosby, R. D. (2001). Food presentation and energy intake in a feeding laboratory study of subjects with binge eating disorder. *International Journal of Eating Disorders, 30*(4), 441-446.

Guerrieri, R., Nederkoorn, C., & Jansen, A. (2007). How impulsiveness and variety influence food intake in a sample of healthy women. *Appetite, 48*(1), 119-122.

Guerrieri, R., Nederkoorn, C., & Jansen, A. (2008). The interaction between impulsivity and a varied food environment: Its influence on food intake and overweight. *International Journal of Obesity (London), 32*(4), 708-714.

Kahn, B. E., & Wansink, B. (2004). The influence of assortment structure on perceived variety and consumption quantities. *Journal of Consumer Research, 30*(4), 519-533.

Lombardini-Riipinen, C., & Lankoski, L. (2010). *Take off the heater: Utility effect and food environment effect in food consumption decisions.* Paper presented at the 1st Joint EAAE/AAEA Seminar, Munich, Germany.

Madzharov, A. V., & Block, L. G. (2010). Effects of product unit image on consumption of snack foods. *Journal of Consumer Psychology, 20*(4), 398-409.

Norton, G. N., Anderson, A. S., & Hetherington, M. M. (2006). Volume and variety: Relative effects on food intake. *Physiology & Behavior, 87*(4), 714-722.

Rolls, B. J. (1985). Experimental analyses of the effects of variety in a meal on human feeding. *American Journal of Clinical Nutrition, 42*(Suppl.5), 932-939.

Rolls, B. J., Rowe, E. A., Rolls, E. T., Kingston, B., Megson, A., & Gunary, R. (1981). Variety in a meal enhances food intake in man. *Physiology & Behavior, 26*(2), 215-221.

Rolls, B. J., Rowe, E. A., & Rolls, E. T. (1982). How sensory properties of foods affect human feeding behavior. *Physiology & Behavior, 29*(3), 409-417.

Smith, J. M., & Ditschun, T. L. (2009). Controlling satiety: How environmental factors influence food intake. *Trends in Food Science & Technology, 20*(6-7), 271-277.

Sobal, J., & Wansink, B. (2007). Kitchenscapes, tablescapes, platescapes, and foodscapes. *Environment and Behavior, 39*(1), 124-142.

Sorensen, L. B., Moller, P., Flint, A., Martens, M., & Raben, A. (2003). Effect of sensory perception of foods on appetite and food intake: A review of studies on humans. *International Journal on Obesity and Related Metabolic Disorders, 27*(10), 1152-1166.

Stubbs, R. J., Johnstone, A. M., Mazlan, N., Mbaiwa, S. E., & Ferris, S. (2001). Effect of altering the variety of sensorially distinct foods, of the same macronutrient content, on food intake and body weight in men. *European Journal of Clinical Nutrition, 55*(1), 19-28.

Temple, J. L., Giacomelli, A. M., Roemmich, J. N., & Epstein, L. H. (2008). Dietary variety impairs habituation in children. *Health Psychology, 27*(Suppl.1), s10-s19.

van Dongen, M. V., De Graaf, C., Siebelink, E., & Kok, F. J. (2009). Hidden fat facilitates passive overconsumption. *Journal of Nutrition, 139*(2), 394-399.

Yeomans, M. R., Gould, N. J., Leitch, M., & Mobini, S. (2009). Effects of energy density and portion size on development of acquired flavour liking and learned satiety. *Appetite, 52*(2), 469-478.

PRESENTATION / TOBACCO

Moodie, C., Mackintosh, A. M., Hastings, G., & Ford, A. (2011). Young adult smokers' perceptions of plain packaging: A pilot naturalistic study. *Tobacco Control, 20*(5), 367-373.

Moodie, C., Stead, M., Bauld, L., McNeill, A., Angus, K., Hinds, K., . . . O'Mara-Eves, A. (2012). Plain Tobacco Packaging: A Systematic Review *UK Centre for Tobacco Control Studies*. Stirling: University of Stirling and the Open University.

PRIMING / DIET

Bell, R., Meiselman, H. L., Pierson, B. J., & Reeve, W. G. (1994). Effects of adding an Italian theme to a restaurant on the perceived ethnicity, acceptability, and selection of foods. *Appetite, 22*(1), 11-24.

Coelho, J. S., Polivy, J., Peter, H. C., & Pliner, P. (2009). Wake up and smell the cookies. Effects of olfactory food-cue exposure in restrained and unrestrained eaters. *Appetite, 52*(2), 517-520.

Cohen, D., & Farley, T. A. (2008). Eating as an automatic behavior. *Preventing Chronic Disease, 5*(1), A23.

Jacob, C., Gueguen, N., & Boulbry, G. (2010). L'effet d'elements figuratifs sur le comportement de consommation: Une illustration de l'influence du choix d'un plat dans un restaurant [The effect of incidental information on consumption behaviour: An Illustration with the choice of a meal in a restaurant. With English summary.]. *La Revue des Sciences de Gestion, 45*(242), 61-67.

Papies, E. K., & Hamstra, P. (2010). Goal priming and eating behavior: Enhancing self-regulation by environmental cues. *Health Psychology, 29*(4), 384-388.

Polivy, J., Herman, C. P., Hackett, R., & Kuleshnyk, I. (1986). The effects of self-attention and public attention on eating in restrained and unrestrained subjects. *Journal of Personality and Social Psychology, 50*(6), 1253-1260.

Shimizu, M., Payne, C. R., & Wansink, B. (2010). When snacks become meals: How hunger and environmental cues bias food intake. *International Journal of Behavioral Nutrition and Physical Activity, 7*(63).

Spence, C., & Shankar, M. U. (2010). The influence of auditory cues on the perception of, and responses to, food and drink. *Journal of Sensory Studies, 25*(3), 406-430.

Zampini, M., & Spence, C. (2010). Assessing the role of sound in the perception of food and drink. *Chemosensory Perception, 3*(1), 57-67.

PRIMING / PHYSICAL ACTIVITY

Barwood, M. J., Weston, N. J. V., Thelwell, R., & Page, J. (2009). A motivational music and video intervention improves high-intensity exercise performance. *Journal of Sports Science and Medicine, 8*(3), 435-442.

PRIMING / ALCOHOL

Jacob, C. (2006). Styles of background music and consumption in a bar: An empirical evaluation. *International Journal of Hospitality Management, 25*(4), 716-720.

North, A. C., Hargreaves, D. J., & McKendrick, J. (1997). In-store music affects product choice. *Nature, 390*(6656), 132.

North, A. C., Hargreaves, D. J., & McKendrick, J. (1999). The influence of in-store music on wine selections. . *Journal of Applied Psychology, 84*(2), 271-276.

Spence, C., & Shankar, M. U. (2010). The influence of auditory cues on the perception of, and responses to, food and drink. *Journal of Sensory Studies, 25*(3), 406-430.

Zampini, M., & Spence, C. (2010). Assessing the role of sound in the perception of food and drink. *Chemosensory Perception, 3*(1), 57-67.

PRIMING / TOBACCO

Rohsenow, D. J., Monti, P. M., Colby, S. M., Gulliver, S. B., & et al. (1997). Effects of alcohol cues on smoking urges and topography among alcoholic men. *Alcoholism: Clinical and Experimental Research, 21*(1), 101-107.

PROMPTING / DIET

Anderson, A. S., Porteous, L. E. G., Foster, E., Higgins, C., Stead, M., Hetherington, M., . . . Adamson, A. J. (2005). The impact of a school-based nutrition education intervention on dietary intake and cognitive and attitudinal variables relating to fruits and vegetables. *Public Health Nutrition, 8*(6), 650-656.

Breugelmans, E., & Campo, K. (2011). Effectiveness of in-store displays in a virtual store environment. *Journal of retailing, 87*(1), 75-89.

Buscher, L. A., Martin, K. A., & Crocker, S. (2001). Point-of-purchase messages framed in terms of cost, convenience, taste, and energy improve healthful snack selection in a college foodservice setting. *Journal of the American Dietetic Association, 101*(8), 909-913.

Chevalier, M. (1975). Increase in sales due to in-store display. *Journal of Marketing Research, 12*(4), 426-431.

Cohen, D., & Farley, T. A. (2008). Eating as an automatic behavior. *Preventing Chronic Disease, 5*(1), A23.

Connell, D., Goldberg, J. P., & Folta, S. C. (2001). An intervention to increase fruit and vegetable consumption using audio communications: In-store public service announcements and audiotapes. *Journal of Health Communication, 6*(1), 31-43.

Cook, C., Simmons, G., Swinburn, B., & Stewart, J. (2001). Changing risk behaviours for non-communicable disease in New Zealand working men--is workplace intervention effective? *The New Zealand Medical Journal, 114*(1130), 175-178.

Engbers, L. H., van Poppel, M. N. M., Chin A Paw, M. J. M., & van Mechelen, W. (2005). Worksite health promotion programs with environmental changes: A systematic review. *American Journal of Preventive Medicine, 29*(1), 61-70.

Engbers, L. H., van Poppel, M. N., Chin, A. P. M., & van Mechelen, W. (2006). The effects of a controlled worksite environmental intervention on determinants of dietary behavior and self-reported fruit, vegetable and fat intake. *BMC Public Health, 6*, 253.

Freedman, M. R., & Connors, R. (2010). Point-of-purchase nutrition information influences food-purchasing behaviors of college students: A pilot study. *Journal of the American Dietetic Association, 110*(8), 1222-1226.

French, S. A., Jeffery, R. W., Story, M., Breitlow, K. K., Baxter, J. S., Hannan, P., & Snyder, M. P. (2001). Pricing and promotion effects on low-fat vending snack purchases: The CHIPS Study. *American Journal of Public Health, 91*(1), 112-127.

Glanz, K., & Mullis, R. M. (1988). Environmental interventions to promote healthy eating: A review of models, programs, and evidence. *Health Education & Behavior, 15*(4), 395-415.

Glanz, K., & Hoelscher, D. (2004). Increasing fruit and vegetable intake by changing environments, policy and pricing: Restaurant-based research, strategies, and recommendations. *Preventive Medicine, 39* (Suppl.2), s88-s93.

Goldberg, M. E., & Gunasti, K. (2007). Creating an environment in which youths are encouraged to eat a healthier diet. *Journal of Public Policy & Marketing, 26*(2), 162-181.

Greco, A. J., & Johnson, E. B. (1997). Supermarket shoppers' response to mature models in point-of-purchase displays. *Journal of Food Products Marketing, 4*(3), 9-23.

Horgen, K. B., & Brownell, K. D. (2002). Comparison of price change and health message interventions in promoting healthy food choices. *Health Psychology, 21*(5), 505-512.

Mayer, J. A., Heins, J. M., Vogel, J. M., Morrison, D. C., Lankester, L. D., & Jacobs, A. L. (1986). Promoting low-fat entree choices in a public cafeteria. *Journal of Applied Behavior Analysis, 19*(4), 397-402.

Mayer, J. A., Dubbert, P. M., & Elder, J. P. (1989). Promoting nutrition at the point of choice: A review. *Health Education Quarterly, 16*(1), 31-43.

Ogawa, Y., Tanabe, N., Honda, A., Azuma, T., Seki, N., Suzuki, T., & Suzuki, H. (2011). Point-of-purchase health information encourages customers to purchase vegetables: Objective analysis by using a point-of-sales system. *Environmental Health and Preventive Medicine, 16*(4), 239-246.

Seymour, J. D., Yaroch, A. L., Serdula, M., Blanck, H. M., & Khan, L. K. (2004). Impact of nutrition environmental interventions on point-of-purchase behavior in adults: A review. *Preventive Medicine, 39* (Suppl.2), s108-s136.

Shepherd, J., Harden, A., Rees, R., Brunton, G., Garcia, J., Oliver, S., & Oakley, A. (2006). Young people and healthy eating: A systematic review of research on barriers and facilitators. *Health Education Research, 21*(2), 239-257.

Sorensen, G., Linnan, L., & Hunt, M. K. (2004). Worksite-based research and initiatives to increase fruit and vegetable consumption. *Preventive Medicine, 39* (Suppl.2), s94-s100.

Wagner, J. L., & Winett, R. A. (1988). Prompting one low-fat, high-fiber selection in a fast-food restaurant. *Journal of Applied Behavior Analysis, 21*(2), 179-185.

Wansink, B., Kent, R. J., & Hoch, S. J. (1998). An anchoring and adjustment model of purchase quantity decisions. *Journal of Marketing Research, 35*(1), 71-81.

Wilbur, C. S., Zifferblatt, S. M., Pinsky, J. L., & Zifferblatt, S. (1981). Healthy vending: A cooperative pilot research program to stimulate good health in the marketplace. *Preventive Medicine, 10*(1), 85-93.

Winett, R. A., Moore, J. F., Wagner, J. L., Hite, L. A., Leahy, M., Neubauer, T. E., . . . Mundy, L. (1991). Altering shoppers' supermarket purchases to fit nutritional guidelines: An interactive information system. *Journal of Applied Behavior Analysis, 24*(1), 95-105.

PROMPTING / PHYSICAL ACTIVITY

Adams, J., & White, M. (2002). A systematic approach to the development and evaluation of an intervention promoting stair use. *Health Education Journal, 61*(3), 272-286.

Andersen, R., Franckowiak, S., Zuzak, K., Cummings, E., Bartlett, S., & Crespo, C. (2006). Effects of a culturally sensitive sign on the use of stairs in African American commuters. *Sozial- und Präventivmedizin [Social and Preventive Medicine], 51*(6), 373-380.

Andersen, R. E., Franckowiak, S. C., Snyder, J., Bartlett, S. J., & Fontaine, K. R. (1998). Can inexpensive signs encourage the use of stairs? Results from a community intervention. *Annals of Internal Medicine, 129*(5), 363-369.

Badland, H. M., & Schofield, G. M. (2005). Posters in a sample of professional worksites have no effect on objectively measured physical activity. *Health Promotion Journal of Australia, 16*(1), 78-81.

Blake, H., Lee, S., Stanton, T., & Gorely, T. (2008). Workplace intervention to promote stair-use in an NHS setting. *International Journal of Workplace Health Management, 1*(3), 162-175.

Blamey, A., Mutrie, N., & Aitchison, T. (1995). Health promotion by encouraged use of stairs. *British Medical Journal, 311*(7000), 289-290.

Boen, F., Maurissen, K., & Opdenacker, J. (2010). A simple health sign increases stair use in a shopping mall and two train stations in Flanders, Belgium. *Health Promotion International, 25*(2), 183-191.

Boutelle, K. N., Jeffery, R. W., Murray, D. M., & Schmitz, M. K. (2001). Using signs, artwork, and music to promote stair use in a public building. *American Journal of Public Health, 91*(12), 2004-2006.

Cheung, P. P., Chow, B. C., & Parfitt, G. (2008). Using environmental stimuli in physical activity intervention for school teachers: A pilot study *International Electronic Journal of Health Education, 11*, 47-56.

Coleman, K. J., & Gonzalez, E. C. (2001). Promoting stair use in a US-Mexico border community. *American Journal of Public Health, 91*(12), 2007-2009.

Cook, C., Simmons, G., Swinburn, B., & Stewart, J. (2001). Changing risk behaviours for non-communicable disease in New Zealand working men--is workplace intervention effective? *The New Zealand Medical Journal, 114*(1130), 175-178.

Dolan, M. S., Weiss, L. A., Lewis, R. A., Pietrobelli, A., Heo, M., & Faith, M. S. (2006). 'Take the stairs instead of the escalator': Effect of environmental prompts on community stair use and implications for a national 'Small Steps' campaign. *Obesity Reviews, 7*(1), 25-32.

Dunn, A. L., Andersen, R. E., & Jakicic, J. M. (1998). Lifestyle physical activity interventions. History, short- and long-term effects, and recommendations. *American Journal of Preventive Medicine, 15*(4), 398-412.

Engbers, L. H., van Poppel, M. N. M., Chin A Paw, M. J. M., & van Mechelen, W. (2005). Worksite health promotion programs with environmental changes: A systematic review. *American Journal of Preventive Medicine, 29*(1), 61-70.

Eves, F. F., & Masters, R. S. (2006). An uphill struggle: Effects of a point-of-choice stair climbing intervention in a non-English speaking population. *International Journal of Epidemiology, 35*(5), 1286-1290.

Eves, F. F., & Webb, O. J. (2006). Worksite interventions to increase stair climbing: Reasons for caution. *Preventive Medicine, 43*(1), 4-7.

Eves, F. F., Webb, O. J., & Mutrie, N. (2006). A workplace intervention to promote stair climbing: Greater effects in the overweight. *Obesity (Silver Spring), 14*(12), 2210-2216.

Eves, F. F. (2008). All choices are not equal: Effects of context on point-of-choice prompts for stair climbing. *Obesity Reviews, 9*(1), 83-84; author reply 85-86.

Eves, F. F., Masters, R. S., & McManus, A. M. (2008). Effects of point-of-choice stair climbing interventions in Hong Kong. *Hong Kong Medical Journal, 14*(Suppl.5), 36-39.

Eves, F. F., Olander, E. K., Nicoll, G., Puig-Ribera, A., & Griffin, C. (2009). Increasing stair climbing in a train station: The effects of contextual variables and visibility. *Journal of Environmental Psychology, 29*(2), 300-303.

Eves, F. F. (2010). Point-of-decision prompts to increase stair use. *American Journal of Preventive Medicine, 38*(5), 573-574.

Eves, F. F., Olander, E. K., Webb, O. J., Griffin, C., & Chambers, J. (2012). Likening stairs in buildings to climbing a mountain: Self-reports of expected effects on stair climbing and objective measures of effectiveness. *Psychology of Sport and Exercise, 13*(2), 170-176.

Foster, C., & Hillsdon, M. (2004). Changing the environment to promote health-enhancing physical activity. *Journal of Sports Sciences, 22*(8), 755-769.

Foster, C., Hillsdon, M., Cavill, N., Bull, F., Buxton, K., & Crombie, H. (2006). Interventions that use the environment to encourage physical activity *Evidence review*. London, UK: National Institute for Health and Clinical Excellence.

Iversen, M. K., Handel, M. N., Jensen, E. N., Frederiksen, P., & Heitmann, B. L. (2007). Effect of health-promoting posters placed on the platforms of two train stations in Copenhagen, Denmark, on the choice between taking the stairs or the escalators: A secondary publication. *International Journal of Obesity, 31*(6), 950-955.

Jenum, A. K., Lorentzen, C. A. N., & Ommundsen, Y. (2009). Targeting physical activity in a low socioeconomic status population: Observations from the Norwegian "Romsas in Motion" study. *British Journal of Sports Medicine, 43*(1), 64-69.

Kahn, E. B., Ramsey, L. T., Brownson, R. C., Heath, G. W., Howze, E. H., Powell, K. E., . . . Corso, P. (2002). The effectiveness of interventions to increase physical activity. A systematic review. *American Journal of Preventive Medicine, 22*(Suppl.4), 73-107.

Kerr, J., Eves, F., & Carroll, D. (2000). Posters can prompt less active people to use the stairs. *Journal of Epidemiology & Community Health, 54*(12), 942.

Kerr, J., Eves, F., & Carroll, D. (2001). Encouraging stair use: Stair-riser banners are better than posters. *American Journal of Public Health, 91*(8), 1192-1193.

Kerr, J., Eves, F., & Carroll, D. (2001). Six-month observational study of prompted stair climbing. *Preventive Medicine, 33*(5), 422-427.

Kerr, J., Eves, F. F., & Carroll, D. (2001). The influence of poster prompts on stair use: The effects of setting, poster size and content. *British Journal of Health Psychology, 6*(Pt.4), 397-405.

Kerr, N. A., Yore, M. M., Ham, S. A., & Dietz, W. H. (2004). Increasing stair use in a worksite through environmental changes. *American Journal of Health Promotion, 18*(4), 312-315.

Kwak, L., Kremers, S. P., van Baak, M. A., & Brug, J. (2007). A poster-based intervention to promote stair use in blue- and white-collar worksites. *Preventive Medicine, 45*(2-3), 177-1781.

Lewis, A., & Eves, F. (2011). Specific effects of a calorie-based intervention on stair climbing in overweight commuters. *Annals of Behavioral Medicine, 42*(2), 257-261.

Lewis, A. L., & Eves, F. F. (2012). Prompts to increase stair climbing in stations: The effect of message complexity. *Journal of Physical Activity & Health, In Press.*

Lewis, A. L., & Eves, F. F. (2012). Testing the theory underlying the success of point-of-choice prompts: A multi-component stair climbing intervention. *Psychology of Sport and Exercise, 13*(2), 126-132.

Marshall, A. L., Bauman, A. E., Patch, C., Wilson, J., & Chen, J. (2002). Can motivational signs prompt increases in incidental physical activity in an Australian health-care facility? *Health Education Research, 17*(6), 743-749.

Meyer, P., Kayser, B., Kossovsky, M. P., Sigaud, P., Carballo, D., Keller, P. F., . . . Mach, F. (2010). Stairs instead of elevators at workplace: Cardioprotective effects of a pragmatic intervention. *European Journal of Cardiovascular Prevention & Rehabilitation, 17*(5), 569-575.

Muller-Riemenschneider, F., Nocon, M., Reinhold, T., & Willich, S. N. (2010). Promotion of physical activity using point-of-decision prompts in Berlin underground stations. *International Journal of Environmental Research and Public Health, 7*(8), 3063-3070.

Nocon, M., Muller-Riemenschneider, F., Nitzschke, K., & Willich, S. N. (2010). Review Article: Increasing physical activity with point-of-choice prompts--a systematic review. *Scandinavian Journal of Public Health, 38*(6), 633-638.

Nomura, T., Yoshimoto, Y., Akezaki, Y., & Sato, A. (2009). Changing behavioral patterns to promote physical activity with motivational signs. *Environmental Health and Preventive Medicine, 14*(1), 20-25.

Olander, E. K., Eves, F. F., & Puig-Ribera, A. (2008). Promoting stair climbing: Stair-riser banners are better than posters… sometimes. *Preventive Medicine, 46*(4), 308-310.

Olander, E. K., & Eves, F. F. (2011). Effectiveness and cost of two stair-climbing interventions—less is more. *American Journal of Health Promotion, 25*(4), 231-236.

Olander, E. K., & Eves, F. F. (2011). Elevator availability and its impact on stair use in a workplace. *Journal of Environmental Psychology, 31*(2), 200-206.

Pillay, J. D., Kolbe-Alexander, T., Achmat, M., Carstene, M., & Lambert, E. V. (2009). Are point-of-decision prompts in a sports science and medicine centre effective in changing the prevalence of stair usage? A preliminary study. *South African Journal of Sports Medicine, 21*(2).

Puig-Ribera, A., & Eves, F. F. (2010). Promoting stair climbing in Barcelona: Similarities and differences with interventions in English-speaking populations. *European Journal of Public Health, 20*(1), 100-102.

Rogers, Y., Hazlewood, W. R., Marshall, P., Dalton, N., & Hetrich, S. (2010). Ambient influence: Can twinkly lights lure and abstract representations trigger behavioural change? *UbiComp 2010, 26-29*, 261-270.

Sallis, J. F., Bauman, A., & Pratt, M. (1998). Environmental and policy interventions to promote physical activity. *American Journal of Preventive Medicine, 15*(4), 379-397.

Soler, R. E., Leeks, K. D., Buchanan, L. R., Brownson, R. C., Heath, G. W., & Hopkins, D. H. (2010). Point-of-decision prompts to increase stair use. A systematic review update. *American Journal of Preventive Medicine, 38*(Suppl.2), s292-s300.

van Houten, R. V., Nau, P. A., & Merrigan, M. (1981). Reducing elevator energy use: A comparison of posted feedback and reduced elevator convenience. *Journal of Applied Behavior Analysis, 14*(4), 377-387.

van Nieuw-Amerongen, M. E., Kremers, S. P. J., de Vries, N. K., & Kok, G. (2011). The use of prompts, increased accessibility, visibility, and aesthetics of the stairwell to promote stair use in a university building. *Environment and Behavior, 43*(1), 131-139.

Webb, O. J., & Cheng, T.-F. (2010). An informational stair climbing intervention with greater effects in overweight pedestrians. *Health Education Research, 25*(6), 936-944.

Webb, O. J., & Eves, F. F. (2007). Effects of environmental changes in a stair climbing intervention: generalization to stair descent. *American Journal of Health Promotion, 22*(1), 38-44.

Webb, O. J., Eves, F. F., & Kerr, J. (2011). A statistical summary of mall-based stair-climbing interventions. *Journal of Physical Activity & Health, 8*(4), 558-565.

Wu, S., Cohen, D., Shi, Y., Pearson, M., & Sturm, R. (2011). Economic analysis of physical activity interventions. *American Journal of Preventive Medicine, 40*(2), 149-158.

PROMPTING / ALCOHOL

Areni, C. S., Duhan, D. F., & Kiecker, P. (1999). Point-of-purchase displays, product organization and brand purchase likelihoods. *Journal of the Academy of Marketing Science, 27*(4), 428-441.

PROMPTING / TOBACCO

Wakefield, M., Germain, D., & Henriksen, L. (2008). The effect of retail cigarette pack displays on impulse purchase. *Addiction, 103*(2), 322-328.

PROXIMITY / DIET

Chandon, P., & Wansink, B. (2011). Is food marketing making us fat? A multi-disciplinary review *Faculty & Research Working Paper*. Fountainbleau, France: Instead: The Business School for the World.

Cohen, D., & Farley, T. A. (2008). Eating as an automatic behavior. *Preventing Chronic Disease, 5*(1), A23.

Dayan, E., & Bar-Hillel, M. (2011). Nudge to nobesity II: Menu positions influence food orders. *Judgment and Decision Making, 6*(4), 333-342.

Engell, D., Kramer, M., Malafi, T., Salomon, M., & Lesher, L. (1996). Effects of effort and social modeling on drinking in humans. *Appetite, 26*(2), 129-138.

Glanz, K., & Hoelscher, D. (2004). Increasing fruit and vegetable intake by changing environments, policy and pricing: Restaurant-based research, strategies, and recommendations. *Preventive Medicine, 39* (Suppl.2), s88-s93.

Huang, A., Barzi, F., Huxley, R., Denyer, G., Rohrlach, B., Jayne, K., & Neal, B. (2006). The effects on saturated fat purchases of providing internet shoppers with purchase- specific dietary advice: A randomised trial. *PLoS Clinical Trials, 1*(5), e22.

Just, D., & Wansink, B. (2009). Smarter lunchrooms: Using behavioral economics to improve meal selection. *Choices, 29*(3).

Kincaid, C. S., & Corsun, D. L. (2003). Are consultants blowing smoke? An empirical test of the impact of menu layout on item sales. *International Journal of Contemporary Hospitality Management, 15*(4).

Lombardini-Riipinen, C., & Lankoski, L. (2010). *Take off the heater: Utility effect and food environment effect in food consumption decisions.* Paper presented at the 1st Joint EAAE/AAEA Seminar, Munich, Germany.

Maas, J., de Ridder, D. T., de Vet, E., & de Wit, J. B. (2011). Do distant foods increase intake? The effect of food accessibility on consumption. *Psychology & Health*.

Meiselman, H. L., Hedderley, D., Staddon, S. L., Pierson, B. J., & Symonds, C. R. (1994). Effect of effort on meal selection and meal acceptability in a student cafeteria. *Appetite, 23*(1), 43-55.

Meyers, A. W., Stunkard, A. J., & Coll, M. (1980). Food accessibility and food choice. A test of Schachter's externality hypothesis. *Archives of General Psychiatry, 37*(10), 1133-1135.

Musher-Eizenman, D. R., Young, K. M., Laurene, K., Galliger, C., Hauser, J., & Wagner, O. M. (2010). Children's sensitivity to external food cues: How distance to serving bowl influences children's consumption. *Health Education & Behavior, 37*(2), 186-192.

Painter, J. E., Wansink, B., & Hieggelke, J. B. (2002). How visibility and convenience influence candy consumption. *Appetite, 38*(3), 237-238.

Rozin, P., Scott, S., Dingley, M., Urbanek, J. K., Jiang, H., & Kaltenbach, M. (2011). Nudge to nobesity I: Minor changes in accessibility decrease food intake. *Judgment and Decision Making, 6*(4), 323-332.

Sigurdsson, V., Larsen, N. M., & Gunnarsson, D. (2011). An in-store experimental analysis of consumers' selection of fruits and vegetables. *The Service Industries Journal, 31*(15), 2587-2602.

Sobal, J., & Wansink, B. (2007). Kitchenscapes, tablescapes, platescapes, and foodscapes. *Environment and Behavior, 39*(1), 124-142.

Thorndike, A. N., Sonnenberg, L., Riis, J., Barraclough, S., & Levy, D. E. (2012). A 2-Phase labeling and choice architecture intervention to improve healthy food and beverage choices. *American Journal of Public Health, 102*(3), 527-533.

Wansink, B. (2004). Environmental factors that increase the food intake and consumption volume of unknowing consumers. *Annual Review of Nutrition, 24*, 455-479.

Wansink, B., Painter, J. E., & Lee, Y. K. (2006). The office candy dish: Proximity's influence on estimated and actual consumption. *International Journal of Obesity, 30*(5), 871-875.

Wisdom, J., Downs, J. S., & Loewenstein, G. (2010). Promoting healthy choices: Information versus convenience. *American Economic Journal: Applied Economics, 2*(2), 164-178.

PROXIMITY / PHYSICAL ACTIVITY

Zimring, C., Joseph, A., Nicoll, G. L., & Tsepas, S. (2005). Influences of building design and site design on physical activity: Research and intervention opportunities. *American Journal of Preventive Medicine, 28*(2 Suppl.2), 186-193.

SIZING / DIET

Burger, K. S., Fisher, J. O., & Johnson, S. L. (2011). Mechanisms behind the portion size effect: Visibility and bite size. *Obesity, 19*(3), 546-551.

Chandon, P., & Wansink, B. (2011). Is food marketing making us fat? A multi-disciplinary review *Faculty & Research Working Paper*. Fountainbleau, France: Instead: The Business School for the World.

Cohen, D., & Farley, T. A. (2008). Eating as an automatic behavior. *Preventing Chronic Disease, 5*(1), A23.

DeGraffe, C., & Hulshof, T. (1996). Effects of weight and energy content of preloads on subsequent appetite and food intake. *Appetite, 26*(2), 139-151.

Devitt, A. A., & Mattes, R. D. (2004). Effects of food unit size and energy density on intake in humans. *Appetite, 42*(2), 213-220.

Diliberti, N., Bordi, P. L., Conklin, M. T., Roe, L. S., & Rolls, B. J. (2004). Increased portion size leads to increased energy intake in a restaurant meal. *Obesity, 12*(3), 562-568.

Ebbeling, C. B., Garcia-Lago, E., Leidig, M. M., Seger-Shippee, L. G., Feldman, H. A., & Ludwig, D. S. (2007). Altering portion sizes and eating rate to attenuate gorging during a fast food meal: Effects on energy intake. *Pediatrics, 119*(5), 869-875.

Ello-Martin, J. A., Ledikwe, J. H., & Rolls, B. J. (2005). The influence of food portion size and energy density on energy intake: Implications for weight management. *American Journal of Clinical Nutrition, 82*(Suppl.1), s236-s241.

Fisher, J. O., Rolls, B. J., & Birch, L. L. (2003). Children's bite size and intake of an entrée are greater with large portions than with age-appropriate or self-selected portions. *The American Journal of Clinical Nutrition, 77*(5), 1164-1170.

Fisher, J. O., Arreola, A., Birch, L. L., & Rolls, B. J. (2007). Portion size effects on daily energy intake in low-income Hispanic and African American children and their mothers. *The American Journal of Clinical Nutrition, 86*(6), 1709-1716.

Fisher, J. O., Liu, Y., Birch, L. L., & Rolls, B. J. (2007). Effects of portion size and energy density on young children's intake at a meal. *American Journal of Clinical Nutrition, 86*(1), 174-179.

Fisher, J. O., & Kral, T. V. (2008). Super-size me: Portion size effects on young children's eating. *Physiology & Behavior, 94*(1), 39-47.

Flood, J. E., Roe, L. S., & Rolls, B. J. (2006). The effect of increased beverage portion size on energy intake at a meal. *Journal of the American Dietetic Association, 106*(12), 1984-1990.

Freedman, M. R., & Brochado, C. (2010). Reducing portion size reduces food intake and plate waste. *Obesity, 18*(9), 1864-1866.

Geier, A. B., Rozin, P., & Doros, G. (2006). Unit bias. A new heuristic that helps explain the effect of portion size on food intake. *Psychological Science, 17*(6), 521-525.

Gosnell, B. A., Mitchell, J. E., Lancaster, K. L., Burgard, M. A., Wonderlich, S. A., & Crosby, R. D. (2001). Food presentation and energy intake in a feeding laboratory study of subjects with binge eating disorder. *International Journal of Eating Disorders, 30*(4), 441-446.

Hartstein, J., Cullen, K. W., Reynolds, K. D., Harrell, J., Resnicow, K., & Kennel, P. (2008). Impact of portion-size control for school a la carte items: Changes in kilocalories and macronutrients purchased by middle school students. *Journal of the American Dietetic Association, 108*(1), 140-144.

Jeffery, R. W., Rydell, S., Dunn, C. L., Harnack, L. J., Levine, A. S., Pentel, P. R., . . . Walsh, E. M. (2007). Effects of portion size on chronic energy intake. *International Journal of Behavioral Nutrition and Physical Activity, 4*, 27.

Kelly, M. T., Wallace, J. M., Robson, P. J., Rennie, K. L., Welch, R. W., Hannon-Fletcher, M. P., . . . Livingstone, M. B. (2009). Increased portion size leads to a sustained increase in energy intake over 4d in normal-weight and overweight men and women. *British Journal of Nutrition, 102*(3), 470-477.

Kral, T. V. E., Roe, L. S., & Rolls, B. J. (2004). Combined effects of energy density and portion size on energy intake in women. *The American Journal of Clinical Nutrition, 79*(6), 962-968.

Kral, T. V., & Rolls, B. J. (2004). Energy density and portion size: Their independent and combined effects on energy intake. *Physiology & Behavior, 82*(1), 131-138.

Kral, T. V. E. (2006). Effects on hunger and satiety, perceived portion size and pleasantness of taste of varying the portion size of foods: A brief review of selected studies. *Appetite, 46*(1), 103-105.

Kral, T. V., Kabay, A. C., Roe, L. S., & Rolls, B. J. (2010). Effects of doubling the portion size of fruit and vegetable side dishes on children's intake at a meal. *Obesity (Silver Spring), 18*(3), 521-527.

Leahy, K. E., Birch, L. L., Fisher, J. O., & Rolls, B. J. (2008). Reductions in entree energy density increase children's vegetable intake and reduce energy intake. *Obesity (Silver Spring), 16*(7), 1559-1565.

Ledikwe, J. H., Ello-Martin, J. A., & Rolls, B. J. (2005). Portion sizes and the obesity epidemic. *Journal of Nutrition, 135*(4), 905-909.

Levitsky, D. A., & Youn, T. (2004). The more food young adults are served, the more they overeat. *Journal of Nutrition, 134*(10), 2546-2549.

Levitsky, D. A. (2005). The non-regulation of food intake in humans: Hope for reversing the epidemic of obesity. *Physiology & Behavior, 86*(5), 623-632.

Lombardini-Riipinen, C., & Lankoski, L. (2010). *Take off the heater: Utility effect and food environment effect in food consumption decisions.* Paper presented at the 1st Joint EAAE/AAEA Seminar, Munich, Germany.

Looney, S. M., & Raynor, H. A. (2011). Impact of portion size and energy density on snack intake in preschool-aged children. *Journal of the American Dietetic Association, 111*(3), 414-418.

Marchiori, D., Waroquier, L., & Klein, O. (2011). Smaller food item sizes of snack foods influence reduced portions and caloric intake in young adults. *Journal of the American Dietetic Association, 111*(5), 727-731.

Marchiori, D., Corneille, O., & Klein, O. (2012). Container size influences snack food intake independently of portion size. *Appetite, 58*(3), 814-817.

Mathias, K. C., Rolls, B. J., Birch, L. L., Kral, T. V. E., Hanna, E. L., Davey, A., & Fisher, J. O. (2011). Serving larger portions of fruits and vegetables together at dinner promotes intake of both foods among young children. *Journal of the American Dietetic Association, 20*(10), 1-5.

Mendoza, J. A., Watson, K., & Cullen, K. W. (2010). Change in dietary energy density after implementation of the Texas Public School Nutrition Policy. *Journal of the American Dietetic Association, 110*(3), 434-440.

Raynor, H. A., & Wing, R. R. (2007). Package unit size and amount of food: Do both influence intake? *Obesity, 15*(9), 2311-2319.

Raynor, H. A., Van Walleghen, E. L., Niemeier, H., Butryn, M. L., & Wing, R. R. (2009). Do food provisions packaged in single-servings reduce energy intake at breakfast during a brief behavioral weight-loss intervention? *Journal of the American Dietetic Association, 109*(11), 1922-1925.

Rolls, B. J., Castellanos, V. H., Halford, J. C., Kilara, A., Panyam, D., Pelkman, C. L., . . . Thorwart, M. L. (1998). Volume of food consumed affects satiety in men. *American Journal of Clinical Nutrition, 67*(6), 1170-1177.

Rolls, B. J., Bell, E. A., & Waugh, B. A. (2000). Increasing the volume of a food by incorporating air affects satiety in men. *American Journal of Clinical Nutrition, 72*(2), 361-368.

Rolls, B. J., Engell, D., & Birch, L. L. (2000). Serving portion size influences 5-year-old but not 3-year-old children's food intakes. *Journal of the American Dietetic Association, 100*(2), 232-234.

Rolls, B. J., Morris, E. L., & Roe, L. S. (2002). Portion size of food affects energy intake in normal-weight and overweight men and women. *The American Journal of Clinical Nutrition, 76*(6), 1207-1213.

Rolls, B. J., Roe, L. S., Kral, T. V., Meengs, J. S., & Wall, D. E. (2004). Increasing the portion size of a packaged snack increases energy intake in men and women. *Appetite, 42*(1), 63-69.

Rolls, B. J., Roe, L. S., & Meengs, J. S. (2004). Salad and satiety: Energy density and portion size of a first-course salad affect energy intake at lunch. *Journal of the American Dietetic Association, 104*(10), 1570-1576.

Rolls, B. J., Roe, L. S., Meengs, J. S., & Wall, D. E. (2004). Increasing the portion size of a sandwich increases energy intake. *Journal of the American Dietetic Association, 104*(3), 367-372.

Rolls, B. J., Roe, L. S., & Meengs, J. S. (2006). Larger portion sizes lead to a sustained increase in energy intake over 2 days. *Journal of the American Dietetic Association, 106*(4), 543-549.

Rolls, B. J., Roe, L. S., & Meengs, J. S. (2006). Reductions in portion size and energy density of foods are additive and lead to sustained decreases in energy intake. *American Journal of Clinical Nutrition, 83*(1), 11-17.

Rolls, B. J., Roe, L. S., & Meengs, J. S. (2007). The effect of large portion sizes on energy intake is sustained for 11 days. *Obesity (Silver Spring), 15*(6), 1535-1543.

Rolls, B. J. (2010). Dietary strategies for the prevention and treatment of obesity. *Proceedings of the Nutrition Society, 69*(1), 70-79.

Rolls, B. J., Roe, L. S., & Meengs, J. S. (2010). Portion size can be used strategically to increase vegetable consumption in adults. *American Journal of Clinical Nutrition, 91*(4), 913-922.

Scott, M. L., Nowlis, S. M., Mandel, N., & Morales, A. C. (2008). The effects of reduced food size and package size on the consumption behavior of restrained and unrestrained eaters. *Journal of Consumer Research, 35*(3), 391-405.

Shepherd, J., Harden, A., Rees, R., Brunton, G., Garcia, J., Oliver, S., & Oakley, A. (2006). Young people and healthy eating: A systematic review of research on barriers and facilitators. *Health Education Research, 21*(2), 239-257.

Smith, J. M., & Ditschun, T. L. (2009). Controlling satiety: How environmental factors influence food intake. *Trends in Food Science & Technology, 20*(6-7), 271-277.

Sobal, J., & Wansink, B. (2007). Kitchenscapes, tablescapes, platescapes, and foodscapes. *Environment and Behavior, 39*(1), 124-142.

Spiegel, T. A., Kaplan, J. M., Tomassini, A., & Stellar, E. (1993). Bite size, ingestion rate, and meal size in lean and obese women. *Appetite, 21*(2), 131-145.

Spill, M. K., Birch, L. L., Roe, L. S., & Rolls, B. J. (2010). Eating vegetables first: The use of portion size to increase vegetable intake in preschool children. *The American Journal of Clinical Nutrition, 91*(5), 1237-1243.

Spill, M. K., Birch, L. L., Roe, L. S., & Rolls, B. J. (2011). Serving large portions of vegetable soup at the start of a meal affected children's energy and vegetable intake. *Appetite, 57*(1), 213-219.

Steenhuis, I. H., & Vermeer, W. M. (2009). Portion size: Review and framework for interventions. *The International Journal of Behavioral Nutrition and Physical Activity, 6*, 58.

Stroebele, N., & de Castro, J. M. (2004). Effect of ambience on food intake and food choice. *Nutrition, 20*(9), 821-838.

Stroebele, N., Ogden, L. G., & Hill, J. O. (2009). Do calorie-controlled portion sizes of snacks reduce energy intake? *Appetite, 52*(3), 793-796.

Wansink, B. (1996). Can package size accelerate usage volume? *The Journal of Marketing, 60*(3), 1-14.

Wansink, B., & Park, S. B. (2001). At the movies: How external cues and perceived taste impact consumption volume. *Food Quality and Preference, 12*(1), 69-74.

Wansink, B. (2004). Environmental factors that increase the food intake and consumption volume of unknowing consumers. *Annual Review of Nutrition, 24*, 455-479.

Wansink, B., & Kim, J. (2005). Bad popcorn in big buckets: Portion size can influence intake as much as taste. *Journal of Nutrition Education and Behavior, 37*(5), 242-245.

Wansink, B., Painter, J. E., & North, J. (2005). Bottomless bowls: Why visual cues of portion size may influence intake. *Obesity Research, 13*(1), 93-100.

Wansink, B., & van Ittersum, K. (2007). Portion size me: Downsizing our consumption norms. *Journal of the American Dietetic Association, 107*(7), 1103-1106.

Wansink, B. (2010). From mindless eating to mindlessly eating better. *Physiology & Behavior, 100*(5), 454-463.

Weijzen, P. L. G., Liem, D. G., Zandstra, E. H., & de Graaf, C. (2008). Sensory specific satiety and intake: The difference between nibble- and bar-size snacks. *Appetite, 50*(2-3), 435-442.

Yeomans, M. R., Gould, N. J., Leitch, M., & Mobini, S. (2009). Effects of energy density and portion size on development of acquired flavour liking and learned satiety. *Appetite, 52*(2), 469-478.

SIZING / TOBACCO

Chait, L. D., & Griffiths, R. R. (1982). Smoking behavior and tobacco smoke intake: Response of smokers to shortened cigarettes. *Clinical Pharmacology and Therapeutics, 32*(1), 90-97.

Excluded articles (excluded at full-text screening stage)

Adams, M. A., Hovell, M. F., Irvin, V., Sallis, J. F., Coleman, K. J., & Liles, S. (2006). Promoting stair use by modeling: An experimental application of the Behavioral Ecological Model. *American Journal of Health Promotion, 21*(2), 101-109.

Addessi, E., Galloway, A. T., Visalberghi, E., & Birch, L. L. (2005). Specific social influences on the acceptance of novel foods in 2-5-year-old children. *Appetite, 45*(3), 264-271.

Attwood, A. S., O'Sullivan, H., Leonards, U., Mackintosh, B., & Munafo, M. R. (2008). Attentional bias training and cue reactivity in cigarette smokers. *Addiction, 103*(11), 1875-1882.

Bannon, K., & Schwartz, M. B. (2006). Impact of nutrition messages on children's food choice: Pilot study. *Appetite, 46*(2), 124-129.

Baranowski, T., Davis, M., Resnicow, K., Baranowski, J., Doyle, C., Lin, L. S., . . . Wang, D. T. (2000). Gimme 5 fruit, juice, and vegetables for fun and health: Outcome evaluation. *Health Education & Behavior, 27*(1), 96-111.

Bargh, J. A., Gollwitzer, P. M., Lee-Chai, A., Barndollar, K., & Trotschel, R. (2001). The automated will: Nonconscious activation and pursuit of behavioral goals. *Journal of Personality and Social Psychology, 81*(6), 1014-1027.

Bauer, D., & Cox, W. M. (1998). Alcohol-related words are distracting to both alcohol abusers and non-abusers in the Stroop colour-naming task. *Addiction, 93*(10), 1539-1542.

Bell, R., & Pliner, P. L. (2003). Time to eat: The relationship between the number of people eating and meal duration in three lunch settings. *Appetite, 41*(2), 215-218.

Bradley, B. P., Field, M., Healy, H., & Mogg, K. (2008). Do the affective properties of smoking-related cues influence attentional and approach biases in cigarette smokers? *Journal of Psychopharmacology, 22*(7), 737-745.

Bradlow, S. T. (2009). Does in-store marketing work? Effects of the number and position of shelf facings on brand attention and evaluation at the point of purchase. *Journal of Marketing, 73*(6), 1-17.

Chaput, J.-P., & Tremblay, A. (2007). Acute effects of knowledge-based work on feeding behavior and energy intake. *Physiology & Behavior, 90*(1), 66-72.

Consolvo, S., Klasnja, P., McDonald, D. W., Avrahami, D., Froehlich, J., LeGrand, L., . . . Landay, J. A. (2008). *Flowers or a robot army? Encouraging awareness & activity with personal, mobile displays.*

Paper presented at the 10th International Conference on Ubiquitous Computing, Seoul, South Korea.

Cornell, C. E., Rodin, J., & Weingarten, H. (1989). Stimulus-induced eating when satiated. *Physiology & Behavior, 45*(4), 695-704.

Critcher, C. R., & Gilovich, T. (2008). Incidental environmental anchors. *Journal of Behavioral Decision Making, 21*(3), 241-251.

Davee, A. M., Blum, J. E., Devore, R. L., Beaudoin, C. M., Kaley, L. A., Leiter, J. L., & Wigand, D. A. (2005). The vending and a la carte policy intervention in Maine public high schools. *Preventing Chronic Disease, 2* (Special), 1-6.

de Castro, J. M. (2004). The time of day of food intake influences overall intake in humans. *Journal of Nutrition, 134*(1), 104-111.

de Castro, J. M., & de Castro, E. S. (1989). Spontaneous meal patterns of humans: Influence of the presence of other people. *American Journal of Clinical Nutrition, 50*(2), 237-247.

de Droog, S. M., Valkenburg, P. M., & Buijzen, M. (2011). Using brand characters to promote young children's liking of and purchase requests for fruit. *Journal of Health Communication, 16*(1), 79-89.

Doumas, D. M., McKinley, L. L., & Book, P. (2009). Evaluation of two Web-based alcohol interventions for mandated college students. *Journal of Substance Abuse Treatment, 36*(1), 65-74.

Epstein, L. H., Masek, B. J., & Marshall, W. R. (1978). A nutritionally based school program for control of eating in obese children. *Behavior Therapy, 9*(5), 766-778.

Fadardi, J. S., & Cox, W. M. (2008). Alcohol-attentional bias and motivational structure as independent predictors of social drinkers' alcohol consumption. *Drug and Alcohol Dependence, 97*(3), 247-256.

Ferber, C., & Cabanac, M. (1987). Influence of noise on gustatory affective ratings and preference for sweet or salt. *Appetite, 8*(3), 229-235.

Ferriday, D., & Brunstrom, J. M. (2008). How does food-cue exposure lead to larger meal sizes? *British Journal of Nutrition, 100*(6), 1325-1332.

Ferriday, D., & Brunstrom, J. M. (2011). "I just can't help myself": Effects of food-cue exposure in overweight and lean individuals. *International Journal of Obesity, 35*(1), 142-149.

Field, M., Duka, T., Eastwood, B., Child, R., Santarcangelo, M., & Gayton, M. (2007). Experimental manipulation of attentional biases in heavy drinkers: Do the effects generalise? *Psychopharmacology, 192*(4), 593-608.

Field, M., & Eastwood, B. (2005). Experimental manipulation of attentional bias increases the motivation to drink alcohol. *Psychopharmacology (Berl), 183*(3), 350-357.

Field, M., Mogg, K., & Bradley, B. P. (2005). Craving and cognitive biases for alcohol cues in social drinkers. *Alcohol and Alcoholism, 40*(6), 504-510.

Flood, J. E., & Rolls, B. J. (2007). Soup preloads in a variety of forms reduce meal energy intake. *Appetite, 49*(3), 626-634.

Flood-Obbagy, J. E., & Rolls, B. J. (2009). The effect of fruit in different forms on energy intake and satiety at a meal. *Appetite, 52*(2), 416-422.

Förster, J. (2003). The influence of approach and avoidance motor actions on food intake. *European Journal of Social Psychology, 33*(3), 339-350.

Freeman, B., Chapman, S., & Rimmer, M. (2008). The case for the plain packaging of tobacco products. *Addiction, 103*(4), 580-590.

French, S. A., Jeffery, R. W., Story, M., Hannan, P., & Snyder, M. P. (1997). A pricing strategy to promote low-fat snack choices through vending machines. *American Journal of Public Health, 87*(5), 849-851.

Friese, M., Hofmann, W., & Wanke, M. (2008). When impulses take over: Moderated predictive validity of explicit and implicit attitude measures in predicting food choice and consumption behaviour. *British Journal of Social Psychology, 47*(Pt.3), 397-419.

Friese, M., Wänke, M., & Plessner, H. (2006). Implicit consumer preferences and their influence on product choice. *Psychology and Marketing, 23*(9), 727-740.

Gallagher, K., & Updegraff, J. (2011). Health message framing effects on attitudes, intentions, and behavior: A meta-analytic review. *Annals of Behavioral Medicine*, 1-16.

Gittelsohn, J., Song, H.-J., Suratkar, S., Kumar, M. B., Henry, E. G., Sharma, S., . . . Anliker, J. A. (2010). An urban food store intervention positively affects food-related psychosocial variables and food behaviors. *Health Education & Behavior, 37*(3), 390-402.

Goldberg, M. E., Liefeld, J., Madill, J., & Vredenburg, H. (1999). The effect of plain packaging on response to health warnings. *American Journal of Public Health, 89*(9), 1434-1435.

Greenfield, T. K., & Kaskutas, L. A. (1993). Early impacts of alcoholic beverage warning labels: National study findings relevant to drinking and driving behavior. *Safety Science, 16*(5-6), 689-707.

Greenhalgh, J., Dowey, A. J., Horne, P. J., Fergus, L. C., Griffiths, J. H., & Whitaker, C. J. (2009). Positive- and negative peer modelling effects on young children's consumption of novel blue foods. *Appetite, 52*(3), 646-653.

Guerrieri, R., Nederkoorn, C., Schrooten, M., Martijn, C., & Jansen, A. (2009). Inducing impulsivity leads high and low restrained eaters into overeating, whereas current dieters stick to their diet. *Appetite, 53*(1), 93-100.

Guerrieri, R., Nederkoorn, C., Stankiewicz, K., Alberts, H., Geschwind, N., Martijn, C., & Jansen, A. (2007). The influence of trait and induced state impulsivity on food intake in normal-weight healthy women. *Appetite, 49*(1), 66-73.

Hammond, D., Fong, G. T., McNeill, A., Borland, R., & Cummings, K. M. (2006). Effectiveness of cigarette warning labels in informing smokers about the risks of smoking: Findings from the International Tobacco Control (ITC) Four Country Survey. *Tobacco Control, 15* (Suppl.3), iii19-iiii25.

Hannum, S. M., Carson, L., Evans, E. M., Canene, K. A., Petr, E. L., Bui, L., & Erdman, J. W., Jr. (2004). Use of portion-controlled entrees enhances weight loss in women. *Obesity Research, 12*(3), 538-546.

Hassan, L. M., Shiu, E., Thrasher, J. F., Fong, G. T., & Hastings, G. (2008). Exploring the effectiveness of cigarette warning labels: Findings from the United States and United Kingdom arms of the International Tobacco Control (ITC) Four Country Survey. *International Journal of Nonprofit and Voluntary Sector Marketing, 13*(3), 263-274.

Healthy Study Group. (2009). HEALTHY study rationale, design and methods: Moderating risk of type 2 diabetes in multi-ethnic middle school students. *International Journal of Obesity, 33*(Suppl.4), s4-s20.

Herman, C. P., Polivy, J., & Silver, R. (1979). Effects of an observer on eating behavior: The induction of "sensible" eating. *Journal of Personality, 47*(1), 85-99.

Herman, C. P., Roth, D. A., & Polivy, J. (2003). Effects of the presence of others on food intake: A normative interpretation. *Psychological Bulletin, 129*(6), 873-886.

Herrington, J. D. (1996). Effects of music in service environments: A field study. *Journal of Services Marketing, 10*(2), 26.

Hoek, J., Wong, C., Gendall, P., Louviere, J., & Cong, K. (2011). Effects of dissuasive packaging on young adult smokers. *Tobacco Control, 20*(3), 183-188.

Hofmann, W., de Houwer, J., Perugini, M., Baeyens, F., & Crombez, G. (2010). Evaluative conditioning in humans: A meta-analysis. *Psychological Bulletin, 136*(3), 390-421.

Hofmann, W., Rauch, W., & Gawronski, B. (2007). And deplete us not into temptation: Automatic attitudes, dietary restraint, and self-regulatory resources as determinants of eating behavior. *Journal of Experimental Social Psychology, 43*(3), 497-504.

Hollands, G. J., Prestwich, A., & Marteau, T. M. (2011). Using aversive images to enhance healthy food choices and implicit attitudes: An experimental test of evaluative conditioning. *Health Psychology, 30*(2), 195-203.

Houben, K. (2011). Overcoming the urge to splurge: Influencing eating behavior by manipulating inhibitory control. *Journal of Behavior Therapy and Experimental Psychiatry, 42*(3), 384-388.

Houben, K., Havermans, R. C., & Wiers, R. W. (2010). Learning to dislike alcohol: Conditioning negative implicit attitudes toward alcohol and its effect on drinking behavior. *Psychopharmacology, 211*(1), 79-86.

Houben, K., & Jansen, A. (2011). Training inhibitory control: A recipe for resisting sweet temptations. *Appetite, 56*(2), 345-349.

Houben, K., Schoenmakers, T. M., & Wiers, R. W. (2010). I didn't feel like drinking but I don't know why: The effects of evaluative conditioning on alcohol-related attitudes, craving and behavior. *Addictive Behaviors, 35*(12), 1161-1163.

Hulshof, T., De Graaf, C., & Weststrate, J. A. (1993). The effects of preloads varying in physical state and fat content on satiety and energy intake. *Appetite, 21*(3), 273-286.

Hwang, J., & Lorenzen, C. L. (2008). Effective nutrition labeling of restaurant menu and pricing of healthy menu. *Journal of Foodservice, 19*(5), 270-276.

Jaeger, S. R., & Rose, J. M. (2008). Stated choice experimentation, contextual influences and food choice: A case study. *Food Quality and Preference, 19*(6), 539-564.

Jago, R., Baranowski, T., & Baranowski, J. C. (2007). Fruit and vegetable availability: A micro environmental mediating variable? *Public Health Nutrition, 10*(7), 681-689.

Jansen, A., & van den Hout, M. (1991). On being led into temptation: "Counterregulation" of dieters after smelling a "preload". *Addictive Behaviors, 16*(5), 247-253.

Johnson, E. J., & Goldstein, D. (2003). Do defaults save lives? (Medicine). *Science, 302*(5649), 1338-1339.

Jones, C. S. (2009). Taking up space? How customers react to health Information and health icons on restaurant menus. *Journal of Foodservice Business Research, 12*(4), 344-363.

Kelly, B., Hughes, C., Chapman, K., Louie, J. C., Dixon, H., Crawford, J., . . . Slevin, T. (2009). Consumer testing of the acceptability and effectiveness of front-of-pack food labelling systems for the Australian grocery market. *Health Promotion International, 24*(2), 120-109.

Kim, S.-Y., Nayga, R. M., Jr., & Capps, O., Jr. (2000). The effect of food label use on nutrient intakes: An endogenous switching regression analysis. *Journal of Agricultural and Resource Economics, 25*(1), 215-231.

Kozup, J. C., Creyer, E. H., & Burton, S. (2003). Making healthful food choices: The influence of health claims and nutrition information on consumers' evaluations of packaged food products and restaurant menu items. *Journal of Marketing Research, 67*(2), 19-34.

Kremers, S. P., de Bruijn, G. J., Visscher, T. L., van Mechelen, W., de Vries, N. K., & Brug, J. (2006). Environmental influences on energy balance-related behaviors: A dual-process view. *International Journal of Behavioral Nutrition and Physical Activity, 3*, 9.

Krukowski, R. A., Harvey-Berino, J., Kolodinsky, J., Narsana, R. T., & Desisto, T. P. (2006). Consumers may not use or understand calorie labeling in restaurants. *Journal of the American Dietetic Association, 106*(6), 917-920.

Lambert, K., Neal, T., Noyes, J., Parker, C., & Worrel, P. (1991). Food-related stimuli increase desire to eat in hungry and satiated human subjects. *Current Psychology: Research & Review, 10*(4), 297-303.

Larson, N., & Story, M. (2009). A review of environmental influences on food choices. *Annals of Behavioral Medicine, 38*(Suppl.1), 56-73.

Lassen, A., Hansen, K., & Trolle, E. (2007). Comparison of buffet and a la carte serving at worksite canteens on nutrient intake and fruit and vegetable consumption. *Public Health Nutrition, 10*(3), 292-297.

Leahy, K. E., Birch, L. L., & Rolls, B. J. (2008). Reducing the energy density of an entrée decreases children's energy intake at lunch. *Journal of the American Dietetic Association, 108*(1), 41-48.

Lemmens, S. G., Martens, E. A., Born, J. M., Martens, M. J., & Westerterp-Plantenga, M. S. (2011). Staggered meal consumption facilitates appetite control without affecting postprandial energy intake. *The Journal of Nutrition, 141*(3), 482-488.

Li, X. (2008). The effects of appetitive stimuli on out-of-domain consumption impatience. *Journal of Consumer Research, 34*(5), 649-656.

Lumeng, J. C., & Hillman, K. H. (2007). Eating in larger groups increases food consumption. *Archives of Disease in Childhood, 92*(5), 384-387.

Lytle, L. A., Kubik, M. Y., Perry, C., Story, M., Birnbaum, A. S., & Murray, D. M. (2006). Influencing healthful food choices in school and home environments: Results from the TEENS study. *Preventive Medicine, 43*(1), 8-13.

Macht, M., Roth, S., & Ellgring, H. (2002). Chocolate eating in healthy men during experimentally induced sadness and joy. *Appetite, 39*(2), 147-158.

MacLeod, C., Rutherford, E., Campbell, L., Ebsworthy, G., & Holker, L. (2002). Selective attention and emotional vulnerability: Assessing the causal basis of their association through the experimental manipulation of attentional bias. *Journal of Abnormal Psychology, 111*(1), 107-123.

Martens, D. (2002). Graphic tobacco warnings having desired effect. *Canadian Medical Association Journal, 166*(11), 1453.

McConahy, K. L., Smiciklas-Wright, H., Birch, L. L., Mitchell, D. C., & Picciano, M. F. (2002). Food portions are positively related to energy intake and body weight in early childhood. *Journal of Pediatrics, 140*(3), 340-347.

Meiselman, H. L., de Graaf, C., & Lesher, L. L. (2000). The effects of variety and monotony on food acceptance and intake at a midday meal. *Physiology & Behavior, 70*(1-2), 119-125.

Milliron, B.-J., Woolf, K., & Appelhans, B. M. (2011). A point-of-purchase intervention featuring in-person supermarket education affects healthful food purchases. *Journal of Nutrition Education and Behavior, 44*(3), 225-232.

Moore, L., & Tapper, K. (2008). The impact of school fruit tuck shops and school food policies on children's fruit consumption: A cluster randomised trial of schools in deprived areas. *Journal of Epidemiology and Community Health, 62*(10), 926-931.

Mourao, D. M., Bressan, J., Campbell, W. W., & Mattes, R. D. (2007). Effects of food form on appetite and energy intake in lean and obese young adults. *International Journal of Obesity (London), 31*(11), 1688-1695.

Mucha, R. F., Geier, A., & Pauli, P. (1999). Modulation of craving by cues having differential overlap with pharmacological effect: Evidence for cue approach in smokers and social drinkers. *Psychopharmacology (Berlin), 147*(3), 306-313.

Mueller, S., & Szolnoki, G. (2010). The relative influence of packaging, labelling, branding and sensory attributes on liking and purchase intent: Consumers differ in their responsiveness. *Food Quality and Preference, 21*(7), 774-783.

Munoz, J. M., Sandstead, H. H., Jacob, R. A., Logan, G. M., Jr., Reck, S. J., Klevay, L. M., . . . Shuey, W. C. (1979). Effects of some cereal brans and textured vegetable protein on plasma lipids. *American Journal of Clinical Nutrition, 32*(3), 580-592.

Nelson, D. C., Almanza, B. A., & Jaffe, W. F. (1997). The effect of point-of-sales nutrition information on the entree selection of patrons of a university foodservice operation. *Journal of Nutrition in Recipe & Menu Development, 2*(4), 29-50.

Nightingale, S. L. (1993). From the food and drug administration. *Journal of the American Medical Association, 269*(8), 974.

Nolan, L. J., & Hetherington, M. M. (2009). The effects of sham feeding-induced sensory specific satiation and food variety on subsequent food intake in humans. *Appetite, 52*(3), 720-725.

Ostafin, B. D., & Palfai, T. P. (2006). Compelled to consume: The Implicit Association Test and automatic alcohol motivation. *Psychology of Addictive Behaviors, 20*(3), 322-327.

Osterhaus, J. T. (1985). Effectiveness of floor displays on the sales of retail products. *Journal of Retailing, 61*(1), 104-116.

Osterholt, K. M., Roe, L. S., & Rolls, B. J. (2007). Incorporation of air into a snack food reduces energy intake. *Appetite, 48*(3), 351-358.

Otten, J. J., Jones, K. E., Littenberg, B., & Harvey-Berino, J. (2009). Effects of television viewing reduction on energy intake and expenditure in overweight and obese adults: A randomized controlled trial. *Archives of Internal Medicine, 169*(22), 2109-2115.

Paradis, G., O'Loughlin, J., Elliott, M., Masson, P., Renaud, L., Sacks-Silver, G., & Lampron, G. (1995). Coeur en sante St-Henri - A heart health promotion programme in a low income, low education neighbourhood in Montreal, Canada: Theoretical model and early field experience. *Journal of Epidemiology and Community Health, 49*(5), 503-512.

Park, C. W. (1989). The effects of situational factors on in-store grocery shopping behavior: The role of store environment and time available for shopping. *The Journal of consumer research, 15*(4), 422-433.

Patel, K. A., & Schlundt, D. G. (2001). Impact of moods and social context on eating behavior. *Appetite, 36*(2), 111-118.

Pedersen, S. D., Kang, J., & Kline, G. A. (2007). Portion control plate for weight loss in obese patients with type 2 diabetes mellitus: A controlled clinical trial. *Archives of Internal Medicine, 167*(12), 1277-1283.

Perugini, M. (2005). Predictive models of implicit and explicit attitudes. *British Journal of Social Psychology, 44*(Pt.1), 29-45.

Peters, E., Romer, D., Slovic, P., Jamieson, K. H., Wharfield, L., Mertz, C. K., & Carpenter, S. M. (2007). The impact and acceptability of Canadian-style cigarette warning labels among U.S. smokers and nonsmokers. *Nicotine & Tobacco Research, 9*(4), 473-481.

Pothos, E. M., & Cox, W. M. (2002). Cognitive bias for alcohol-related information in inferential processes. *Drug and Alcohol Dependence, 66*(3), 235-241.

Powell, J., Tait, S., & Lessiter, J. (2002). Cigarette smoking and attention to signals of reward and threat in the Stroop paradigm. *Addiction, 97*(9), 1163-1170.

Pratt, C. A., Lemon, S. C., Fernandez, I. D., Goetzel, R., Beresford, S. A., French, S. A., . . . Webber, L. S. (2007). Design characteristics of worksite environmental interventions for obesity prevention. *Obesity, 15*(9), 2171-2180.

Prestwich, A., Hurling, R., & Baker, S. (2011). Implicit shopping: Attitudinal determinants of the purchasing of healthy and unhealthy foods. *Psychology & Health, 26*(7), 875-885.

Qin, Y., Wu, M., Pan, X., Xiang, Q., Huang, J., Gu, Z., . . . Zhou, M. (2011). Reactions of Chinese adults to warning labels on cigarette packages: A survey in Jiangsu Province. *BMC Public Health, 11*, 133.

Ratner, R. K., & Kahn, B. E. (2002). Private vs. public consumption: The impact of impression management on variety seeking. *Journal of Consumer Research, 29*(2), 246-257.

Raynor, H. A., & Wing, R. R. (2006). Effect of limiting snack food variety across days on hedonics and consumption. *Appetite, 46*(2), 168-176.

Redden, J. P., & Hoch, S. J. (2009). The presence of variety reduces perceived quantity. *Journal of Consumer Research, 36*(3), 406-417.

Reitz, A. L., & Hawkins, R. P. (1982). Increasing the attendance of nursing home residents at group recreation activities. *Behavior Therapy, 13*(3), 283-290.

Romero, N. D., Epstein, L. H., & Salvy, S. J. (2009). Peer modeling influences girls' snack intake. *Journal of the American Dietetic Association, 109*(1), 133-136.

Rotenberg, K. J., Lancaster, C., Marsden, J., Pryce, S., Williams, J., & Lattimore, P. (2005). Effects of priming thoughts about control on anxiety and food intake as moderated by dietary restraint. *Appetite, 44*(2), 235-241.

Rozin, P., Kabnick, K., Pete, E., Fischler, C., & Shields, C. (2003). The ecology of eating: Smaller portion sizes in France than in the United States help explain the French paradox. *Psychological Science, 14*(5), 450-454.

Ruijschop, R. M., Zijlstra, N., Boelrijk, A. E., Dijkstra, A., Burgering, M. J., Graaf, C., & Westerterp-Plantenga, M. S. (2011). Effects of bite size and duration of oral processing on retro-nasal aroma release - features contributing to meal termination. *British Journal of Nutrition, 105*(2), 307-315.

Salvy, S. J., Coelho, J. S., Kieffer, E., & Epstein, L. H. (2007). Effects of social contexts on overweight and normal-weight children's food intake. *Physiology & Behavior, 92*(5), 840-846.

Salvy, S. J., Kieffer, E., & Epstein, L. H. (2008). Effects of social context on overweight and normal-weight children's food selection. *Eating Behaviors, 9*(2), 190-196.

Salvy, S.-J., Howard, M., Read, M., & Mele, E. (2009). The presence of friends increases food intake in youth. *The American Journal of Clinical Nutrition, 90*(2), 282-287.

Scheibehenne, B., Miesler, L., & Todd, P. M. (2007). Fast and frugal food choices: Uncovering individual decision heuristics. *Appetite, 49*(3), 578-589.

Schifferstein, H. N. J., Talke, K. S. S., & Oudshoorn, D. J. (2011). Can ambient scent enhance the nightlife experience? *Chemosensory Perception, 4*(1-2), 55-64.

Schneider, S., Gadinger, M., & Fischer, A. (2011). Does the effect go up in smoke? A randomized controlled trial of pictorial warnings on cigarette packaging. *Patient Education and Counseling, 86*, 77-83.

Schoenmakers, T., Wiers, R. W., Jones, B. T., Bruce, G., & Jansen, A. T. M. (2007). Attentional re-training decreases attentional bias in heavy drinkers without generalization. *Addiction, 102*(3), 399-405.

Sherman, D. K., Gangi, C., & White, M. L. (2010). Embodied cognition and health persuasion: Facilitating intention-behavior consistency via motor manipulations. *Journal of Experimental Social Psychology, 46*(2), 461-464.

Simonson, I. (1990). The effect of purchase quantity and timing on variety-seeking behavior. *Journal of Marketing Research, 27*(2), 150-162.

Simonson, I., & Winer, R. S. (1992). The influence of purchase quantity and display format on consumer preference for variety. *Journal of Consumer Research, 19*(1), 133-138.

Spiegel, T. A., & Stellar, E. (1990). Effects of variety on food intake of underweight, normal-weight and overweight women. *Appetite, 15*(1), 47-61.

Spijkerman, R., Roek, M. A., Vermulst, A., Lemmers, L., Huiberts, A., & Engels, R. C. (2010). Effectiveness of a web-based brief alcohol intervention and added value of normative feedback in reducing underage drinking: A randomized controlled trial. *Journal of Medical Internet Research, 12*(5), e65.

Stark, L. J., Collins, F. L., Jr., Osnes, P. G., & Stokes, T. F. (1986). Using reinforcement and cueing to increase healthy snack food choices in preschoolers. *Journal of Applied Behavior Analysis, 19*(4), 367-379.

Sternfeld, B., Block, C., Quesenberry, C. P., Jr., Block, T. J., Husson, G., Norris, J. C., . . . Block, G. (2009). Improving diet and physical activity with ALIVE: A worksite randomized trial. *American Journal of Preventive Medicine, 36*(6), 475-483.

Stirling, L. J., & Yeomans, M. R. (2004). Effect of exposure to a forbidden food on eating in restrained and unrestrained women. *International Journal of Eating Disorders, 35*(1), 59-68.

Stormark, K. M., Field, N. P., Hugdahl, K., & Horowitz, M. (1997). Selective processing of visual alcohol cues in abstinent alcoholics: An approach-avoidance conflict? *Addictive Behaviors, 22*(4), 509-519.

Story, M., Neumark-Sztainer, D., & French, S. (2002). Individual and environmental influences on adolescent eating behaviors. *Journal of the American Dietetic Association, 102*(Suppl.3), s40-s51.

Stote, K. S., Baer, D. J., Spears, K., Paul, D. R., Harris, G. K., Rumpler, W. V., . . . Mattson, M. P. (2007). A controlled trial of reduced meal frequency without caloric restriction in healthy, normal-weight, middle-aged adults. *American Journal of Clinical Nutrition, 85*(4), 981-988.

Strack, F., & Deutsch, R. (2004). Reflective and impulsive determinants of social behavior. *Personality and Social Psychology Review, 8*(3), 220-247.

Strahan, E. J., White, K., Fong, G. T., Fabrigar, L. R., Zanna, M. P., & Cameron, R. (2002). Enhancing the effectiveness of tobacco package warning labels: A social psychological perspective. *Tobacco Control, 11*(3), 183-190.

Stroebele, N., & de Castro, J. M. (2006). Listening to music while eating is related to increases in people's food intake and meal duration. *Appetite, 47*(3), 285-289.

Thomas, M., Desai, K. K., & Seenivasan, S. (2011). How credit card payments increase unhealthy food purchases: Visceral regulation of vices. *Journal of Consumer Research, 38*(1), 126-139.

Thorsen, A. V., Lassen, A. D., Tetens, I., Hels, O., & Mikkelsen, B. E. (2010). Long-term sustainability of a worksite canteen intervention of serving more fruit and vegetables. *Public Health Nutrition, 13*(10), 1647-1652.

Thrasher, J. F., Rousu, M. C., Anaya-Ocampo, R., Reynales-Shigematsu, L. M., Arillo-Santillan, E., & Hernandez-Avila, M. (2007). Estimating the impact of different cigarette package warning label policies: The auction method. *Addictive Behaviors, 32*(12), 2916-2925.

Thrasher, J. F., Rousu, M. C., Hammond, D., Navarro, A., & Corrigan, J. R. (2011). Estimating the impact of pictorial health warnings and "plain" cigarette packaging: Evidence from experimental auctions among adult smokers in the United States. *Health Policy, 102*(1), 41-48.

Tibor, P. (2004). Automatic processes in self-regulation: Implications for alcohol interventions. *Cognitive and Behavioral Practice, 11*(2), 190-201.

Titze, S., Martin, B. W., Seiler, R., & Marti, B. (2001). A worksite intervention module encouraging the use of stairs: Results and evaluation issues. *Soz Praventivmed, 46*(1), 13-19.

Todd, P. M., Rogers, Y., & Payne, S. (2011). Nudging the trolley in the supermarket: How to deliver the right information to shoppers. *International Journal of Mobile Human Computer Interaction 3*(2), 20-34.

Townshend, J. M., & Duka, T. (2001). Attentional bias associated with alcohol cues: Differences between heavy and occasional social drinkers. *Psychopharmacology (Berlin), 157*(1), 67-74.

Veling, H., Aarts, H., & Papies, E. K. (2011). Using stop signals to inhibit chronic dieters' responses toward palatable foods. *Behaviour Research and Therapy, 49*(11), 771-780.

Veling, H., Aarts, H., & Stroebe, W. (2011). Fear signals inhibit impulsive behavior toward rewarding food objects. *Appetite, 56*(3), 643-648.

Veltkamp, M., Custers, R., & Aarts, H. (2011). Motivating consumer behavior by subliminal conditioning in the absence of basic needs: Striking even while the iron is cold. *Journal of Consumer Psychology, 21*(1), 49-56.

Viskaal-van Dongen, M., de Graaf, C., Siebelink, E., & Kok Frans, J. (2009). Hidden fat facilitates passive overconsumption. *Journal of Nutrition, 139*(2), 394-399.

Wakefield, M., Germain, D., Durkin, S., & Henriksen, L. (2006). An experimental study of effects on schoolchildren of exposure to point-of-sale cigarette advertising and pack displays. *Health Education Research, 21*(3), 338-347.

Wansink, B., & Deshpande, R. (1994). "Out of sight, out of mind": Pantry stockpiling and brand-usage frequency. *Marketing Letters, 5*(1), 91-100.

Wansink, B., & Sobal, J. (2007). Mindless eating: The 200 daily food decisions we overlook. *Environment and Behavior, 39*(1), 106-123.

Webb, O. J., Eves, F. F., & Smith, L. (2011). Investigating behavioural mimicry in the context of stair/escalator choice. *British Journal of Health Psychology, 16*(Pt.2), 373-385.

Weijzen, P. L. G., Smeets, P. A. M., & de Graaf, C. (2009). Sip size of orangeade: Effects on intake and sensory-specific satiation. *British Journal of Nutrition, 102*(7), 1091-1097.

Whatley, B. J. E., Davee, A. M., Devore, R. L., Beaudoin, C. M., Jenkins, P. L., Kaley, L. A., & Wigand, D. A. (2007). Implementation of low-fat, low-sugar, and portion-controlled nutrition guidelines in competitive food venues of Maine public high schools. *Journal of School Health, 77*(10), 687-693.

White, A., Kavanagh, D., Stallman, H., Klein, B., Kay-Lambkin, F., Proudfoot, J., . . . Young, R. (2010). Online alcohol interventions: A systematic review. *Journal of Medical Internet Research, 12*(5), e62.

White, V., Tan, N., Wakefield, M., & Hill, D. (2003). Do adult focused anti-smoking campaigns have an impact on adolescents? The case of the Australian National Tobacco Campaign. *Tobacco Control, 12* (Suppl.2), ii23-ii29.

Wiers, R. W., Cox, W. M., Field, M., Fadardi, J. S., Palfai, T. P., Schoenmakers, T., & Stacy, A. W. (2006). The search for new ways to change implicit alcohol-related cognitions in heavy drinkers. *Alcoholism: Clinical and Experimental Research, 30*(2), 320-331.

Wiers, R. W., Rinck, M., Kordts, R., Houben, K., & Strack, F. (2010). Retraining automatic action-tendencies to approach alcohol in hazardous drinkers. *Addiction, 105*(2), 279-287.

Wiers, R. W., van de Luitgaarden, J., van den Wildenberg, E., & Smulders, F. T. (2005). Challenging implicit and explicit alcohol-related cognitions in young heavy drinkers. *Addiction, 100*(6), 806-819.

Wiers, R. W., van Woerden, N., Smulders, F. T., & de Jong, P. J. (2002). Implicit and explicit alcohol-related cognitions in heavy and light drinkers. *Journal of Abnormal Psychology, 111*(4), 648-658.

Wilson, S. (2003). The effect of music on perceived atmosphere and purchase intentions in a restaurant. *Psychology of Music, 31*(1), 93-112.

Witte, K., & Allen, M. (2000). A meta-analysis of fear appeals: Implications for effective public health campaigns. *Health Education & Behavior, 27*(5), 591-615.

Young, L. R., & Nestle, M. (2002). The contribution of expanding portion sizes to the US obesity epidemic. *American Journal of Public Health, 92*(2), 246-249.

Zack, M., Poulos, C. X., Fragopoulos, F., Woodford, T. M., & MacLeod, C. M. (2006). Negative affect words prime beer consumption in young drinkers. *Addictive Behaviors, 31*(1), 169-173.

Zepeda, L., & Deal, D. (2008). Think before you eat: Photographic food diaries as intervention tools to change dietary decision making and attitudes. *International Journal of Consumer Studies, 32*(6), 692-698.

Zijlstra, N., de Wijk, R., Mars, M., Stafleu, A., & de Graaf, C. (2009). Effect of bite size and oral processing time of a semisolid food on satiation. *The American Journal of Clinical Nutrition, 90*(2), 269-275.

Zijlstra, N., Mars, M., Stafleu, A., & de Graaf, C. (2010). The effect of texture differences on satiation in 3 pairs of solid foods. *Appetite, 55*(3), 490-497.

Articles identified but not assessed (Full-text could not be readily obtained)

Ahn, H. J., Eom, Y. K., Han, K. A., Kwon, H. R., Kim, H. J., Park, K. S., & Min, K. W. (2010). The effects of small sized rice bowl on carbohydrate intake and dietary patterns in women with type 2 diabetes. *Korean Diabetes Journal, 34*(3), 166-173.

Ahn, H. J., Han, K. A., Jang, J. Y., Lee, J. H., Park, K. S., & Min, K. W. (2011). Small rice bowl-based meal plan for energy and macronutrient intake in Korean men with type 2 diabetes: A pilot study. *Diabetes & Metabolism Journal, 35*(3), 273-281.

Ahn, H. J., Han, K. A., Kwon, H. R., Koo, B. K., Kim, H. J., Park, K. S., & Min, K. W. (2010). Small rice bowl-based meal plan versus food exchange-based meal plan for weight, glucose and lipid control in obese type 2 diabetic patients. *Korean Diabetes Journal, 34*(2), 86-94.

Albright, C. L., Flora, J. A., & Fortmann, S. P. (1990). Restaurant menu labeling: Impact of nutrition information on entree sales and patron attitudes. *Health Education & Behavior, 17*(2), 157-167.

Allred, J. B. (1995). Too much of a good thing? An overemphasis on eating low-fat foods may be contributing to the alarming increase in overweight among US adults. *Journal of the American Dietetic Association, 95*(4), 417-418.

Andersen, R. E., Bauman, A. E., Franckowiak, S. C., Reilley, S. M., & Marshall, A. L. (2008). Prompting health professionals to be activity role models--motivating stair use at the 2001 ACSM scientific meeting. *Journal of Physical Activity & Health, 5*(4), 607-618.

Andersen, R. E., Franckowiak, S. C., & Zuzak, K. B. (2000). Community intervention to encourage stair use among African-American commuters. *Medicine and Science in Sports and Exercise, 32*(Suppl.), s38.

Argo, J. J., & White, K. (2012). When do consumers eat more? The role of appearance self-esteem and food packaging cues. *Journal of Marketing, 76*(2), 67-80.

Auweele, Y. V., Boen, F., Schapendonk, W., & Dornez, K. (2005). Promoting stair use among female employees: The effects of a health sign followed by an e-mail. *Journal of Sport & Exercise Psychology, 27*(2), 188-196.

Balfour, D., Moody, R., Wise, A., & Brown, K. (1996). Food choice in response to computer-generated nutrition information provided about meal selections in workplace restaurants. *Journal of Human Nutrition and Dietetics, 9*(3), 231-237.

Barreiro-Hurlé, J., Gracia, A., & de-Magistris, T. (2010). Does nutrition information on food products lead to healthier food choices? *Food Policy, 35*(3), 221-229.

Baum, A., Riess, M., & O'Hara, J. (1974). Architectural variants of reaction to spatial invasion. *Environment and Behavior, 6*(1), 91-100.

Beatty, W. W. (1982). Dietary variety stimulates appetite in females but not in males. *Bulletin of the Psychonomic Society, 19*(4), 212-214.

Bellissimo, N., Pencharz, P. B., Thomas, S. G., & Anderson, G. H. (2007). Effect of television viewing at mealtime on food intake after a glucose preload in boys. *Pediatric Research, 61*(6), 745-749.

Berry, S. L., Beatty, W. W., & Klesges, R. C. (1985). Sensory and social influences on ice cream consumption by males and females in a laboratory setting. *Appetite, 6*(1), 41-45.

Bickmore, T., Gruber, A., & Intille, S. (2008). *Just-in-time automated counseling for physical activity promotion.* Paper presented at the American Medical Informatics Association (AMIA) Boston, MA, United States.

Bitner, M. J. (1986). Consumer responses to the physical environment in service settings. In M. Venkatesan, D. H. Schmalensee, C. E. Marshall & American Marketing Association (Eds.), *Creativity in Services Marketing* (pp. 69-82). Chicago: American Marketing Association.

Bradley, B., Field, M., Mogg, K., & de Houwer, J. (2004). Attentional and evaluative biases for smoking cues in nicotine dependence: Component processes of biases in visual orienting. *Behavioural Pharmacology, 15*(1), 29-36.

Brehm, B. J., Gates, D. M., Singler, M., Succop, P. A., & D'Alessio, D. A. (2011). Environmental changes to control obesity: A randomized controlled trial in manufacturing companies. *American Journal of Health Promotion, 25*(5), 334-340.

Brink, L., A., Nigg, C. R., Lampe, S. M., Kingston, B. A., Mootz, A. L., & van Vliet, W. (2010). Influence of schoolyard renovations on children's physical activity: The Learning Landscapes Program. *American Journal of Public Health, 100*(9), 1672-1678.

Brouillette, M. E., & White, L. W. (1991). The effects of olfactory stimulation on the appetites of nursing home residents. *Physical & Occupational Therapy in Geriatrics, 10*(1), 1-13.

Brown, R. A., & Williams, R. J. (1979). The effect of cues of quantity visible and preference on drinking by alcoholic and non-alcoholic subjects. *British Journal of Social & Clinical Psychology, 18*(1), 99-104.

Brownell, K. D., Stunkard, A. J., & Albaum, J. M. (1980). Evaluation and modification of exercise patterns in the natural environment. *American Journal of Psychiatry, 137*(12), 1540-1545.

Brunstrom, J. M., Yates, H. M., & Witcomb, G. L. (2004). Dietary restraint and heightened reactivity to food. *Physiology & Behavior, 81*(1), 85-90.

Bungum, T., Meacham, M., & Truax, N. (2007). The effects of signage and the physical environment on stair usage. *Journal of Physical Activity & Health, 4*(3), 237-244.

Burchett, H. (2003). Increasing fruit and vegetable consumption among British primary schoolchildren: A review. *Health Education, 103*(2), 99-109.

Cavazza, N., Graziani, A. R., & Guidetti, M. (2011). Looking for the "right" amount to eat at the restaurant: Social influence effects when ordering. *Social Influence, 6*(4), 274-290.

Coelho, J. S. (2007). *Eating behaviour in response to olfactory food cues: The role of dietary restraint.* Doctoral dissertation, University of Toronto, Toronto, ON, Canada.

Cohen, D. A. (2008). Obesity and the built environment: Changes in environmental cues cause energy imbalances. *International Journal of Obesity, 32*(Suppl.7), s137-s142.

Colby, J., Elder, J. P., Peterson, G., & Knisley, P. M. (1987). Promoting the selection of healthy food through menu item description in a family-style restaurant. *American Journal of Preventive Medicine, 3*(3), 171-177.

Conger, J. C., Conger, A. J., Costanzo, P. R., Wright, K. L., & Matter, J. A. (1980). The effect of social cues on the eating behavior of obese and normal subjects. *Journal of Personality, 48*(2), 258-271.

Conklin, M. T., Cranage, D. A., & Lambert, C. U. (2005). College students' use of point of selection nutrition information. *Topics in Clinical Nutrition, 20*(2), 97-108.

Conklin, M. T., Lambert, C. U., & Cranage, D. A. (2005). Nutrition information at point of selection could benefit college students. *Topics in Clinical Nutrition, 20*(2), 90-96.

Cooley, P. D., Foley, S. J., & Magnussen, C. G. (2008). Increasing stair usage in a professional workplace: A test of the efficacy of positive and negative message prompts to change pedestrian choices. *Health Promotion Journal of Australia, 19*(1), 64-67.

Cranage, D. A., Conklin, M. T., & Lambert, C. U. (2005). Effect of nutrition information in perceptions of food quality, consumption behavior and purchase intentions. *Journal of Foodservice Business Research, 7*(1), 43-61.

Cugelman, B., Thelwall, M., & Dawes, P. (2011). Online interventions for social marketing health behavior change campaigns: A meta-analysis of psychological architectures and adherence factors. *Journal of Medical Internet Research, 13*(1), e17.

Cullen, K. W., Baranowski, T., Owens, E., Marsh, T., Rittenberry, L., & de Moor, C. (2003). Availability, accessibility, and preferences for fruit, 100% fruit juice, and vegetables influence children's dietary behavior. *Health Education & Behavior, 30*(5), 615-626.

Davis, D. Z., & Rogers, T. (1982). Point-of-choice nutrition information for the modification of milk selection. *Journal of American College Health Association, 30*(6), 275-278.

Davis-Chervin, D., Rogers, T., & Clark, M. (1985). Influencing food selection with point-of-choice nutrition information. *Journal of Nutrition Education, 17*(1), 18-22.

De Castro, J. M. (1990). Social facilitation of duration and size but not rate of the spontaneous meal intake of humans. *Physiology & Behavior, 47*(6), 1129-1135.

de Wijk, R. A., Zijlstra, N., Mars, M., de Graaf, C., & Prinz, J. F. (2008). The effects of food viscosity on bite size, bite effort and food intake. *Physiology & Behavior, 95*(3), 527-532.

DeWijk, R., A., & Zijlstra, S. (2010). The effect of two ambient aromas on human physiology and food choice. *Chemical Senses, 35* (7), A50.

Dhar, R., & Simonson, I. (2003). The effect of forced choice on choice. *Journal of Marketing Research, 40*(2), 146-160.

Drews, D. R., Vaughn, D. B., & Anfiteatro, A. (1992). Beer consumption as a function of music and the presence of others. *Journal of Pennsylvania Academy of Science, 65*, 134-136.

Dubois, A., Strychar, I. M., Champagne, F., Leblanc, M. P., & Tremblay, C. (1996). The effect of a worksite cafeteria program on employees' dietary fat intakes. *Journal of the Canadian Dietetic Association-Revue De L Association Canadienne Des Dietetistes, 57*(3), 98-102.

Dumanovsky, T., Nonas, C. A., Huang, C. Y., Silver, L. D., & Bassett, M. T. (2009). What people buy from fast-food restaurants: Caloric content and menu item selection, New York City 2007. *Obesity (Silver Spring), 17*(7), 1369-1374.

Edelman, B., Engell, D., Bronstein, P., & Hirsch, E. (1986). Environmental effects on the intake of overweight and normal-weight men. *Appetite,* 7(1), 71-83.

Edworthy, J., & Waring, H. (2006). The effects of music tempo and loudness level on treadmill exercise. *Ergonomics, 49*(15), 1597-1610.

Emmons, K. M., Linnan, L. A., Shadel, W. G., Marcus, B., & Abrams, D. B. (1999). The working healthy project: A worksite health-promotion trial targeting physical activity, diet, and smoking. *Journal of Occupational and Environmental Medicine, 41*(7), 545-555.

Engell, D., Kramer, M., Zaring, D., Birch, L., & Rolls, B. (1995). Effects of serving size on food intake in children and adults. *Obesity Research, 3*(3).

Engels, R. C., Slettenhaar, G., Bogt, T., & Scholte, R. H. (2011). Effect of alcohol references in music on alcohol consumption in public drinking places. *American Journal of Addiction, 20*(6), 530-534.

Eves, F. F., Masters, R. S., McManus, A., Leung, M., Wong, P., & White, M. J. (2008). Contextual barriers to lifestyle physical activity interventions in Hong Kong. *Medicine & Science in Sports & Exercise, 40*(5), 965-971.

Fanslow, J. L., Leland, L. S., Craig, T., & Hahn, H. (1988). Reducing passive smoking by promoting self segregation of smokers using signs and thematic prompts. *Journal of Organizational Behavior Management, 9*(2), 23-34.

Fardy, P. S., & Ilmarinen, J. (1975). Evaluating the effects and feasibility of an at work stairclimbing intervention program for men. *Medicine and Science in Sports,* 7(2), 91-93.

Fathelrahman, A. I., Omar, M., Awang, R., Cummings, K. M., Borland, R., & Samin, A. S. B. M. (2010). Impact of the new Malaysian cigarette pack warnings on smokers' awareness of health risks and interest in quitting smoking. *International Journal of Environmental Research & Public Health* 7(11), 4089-4099.

Fedoroff, I. C., Polivy, J., & Herman, C. P. (1997). The effect of pre-exposure to food cues on the eating behavior of restrained and unrestrained eaters. *Appetite, 28*(1), 33-47.

Feldman, C., Mahadevan, M., Su, H., Brusca, J., & Ruzsilla, J. (2011). Menu engineering: A strategy for seniors to select healthier meals. *Perspectives in Public Health, 131*(6), 267-274.

Field, M., & Cox, W. M. (2008). Attentional bias in addictive behaviors: A review of its development, causes, and consequences. *Drug and Alcohol Dependence, 97*(1-2), 1-20.

Field, M., & Duka, T. (2002). Cues paired with a low dose of alcohol acquire conditioned incentive properties in social drinkers. *Psychopharmacology (Berlin), 159*(3), 325-334.

Field, M., Kiernan, A., Eastwood, B., & Child, R. (2008). Rapid approach responses to alcohol cues in heavy drinkers. *Journal of Behavior Therapy and Experimental Psychiatry, 39*(3), 209-218.

Fisher, J. O., & Birch, L. L. (1999). Restricting access to foods and children's eating. *Appetite, 32*(3), 405-419.

Fong, G. T., Cameron, A. J. R., Brown, K. S., Campbell, H. S., Zanna, M. P., & Murnaghan, D. (2002). *Effects of the Canadian graphic warning labels among high school students: A quasi-experimental longitudinal survey.* Paper presented at the National Conference on Tobacco or Health, San Francisco, CA, United States.

Ford, M. A., & Torok, D. (2008). Motivational signage increases physical activity on a college campus. *Journal of American College Health, 57*(2), 242-244.

Friman, P. C. (1995). Making life easier with effort: Basic findings and applied research on response effort. *Journal of Applied Behavior Analysis, 28*(4), 583-590.

Gamio, M. O. (1996). *The influence of point-of-purchase cafeteria-based nutrition education program on high school students.* Doctoral dissertation, University of Tennessee, Knoxville, TN, United States.

Geiger, C. J., Wyse, B. W., Parent, C. R., & Hansen, R. G. (1991). Review of nutrition labeling formats. *Journal of the American Dietetic Association, 91*(7), 808-812, 815.

Gibbons, M. R. D., & Henry, C. J. K. (2005). Does eating environment have an effect on food intake in the elderly? *Journal of Nutrition, Health and Aging, 9*(1), 25-29.

Girz, L., Polivy, J., Herman, C. P., & Lee, H. (2011). The effects of calorie information on food selection and intake. [Online publication]. *International Journal of Obesity (London).*

Glanz, K., Lankenau, B., Foerster, S., Temple, S., Mullis, R., & Schmid, T. (1995). Environmental and policy approaches to cardiovascular disease prevention through nutrition: Opportunities for state and local action. *Health Education Quarterly, 22*(4), 512-527.

Grimstvedt, M. E., Kerr, J., Oswalt, S. B., Fogt, D. L., Vargas-Tonsing, T. M., & Yin, Z. (2010). Using signage to promote stair use on a university campus in hidden and visible stairwells. *Journal Physical Activity & Health, 7*(2), 232-238.

Hammond, D., McDonald, P. W., Fong, G. T., Brown, K. S., & Cameron, R. (2004). The impact of cigarette warning labels and smoke-free bylaws on smoking cessation: Evidence from former smokers. *Canadian Journal of Public Health, 95*(3), 201-204.

Handel, M. N., Iversen, M. K., Jensen, E. N., Frederiksen, P., & Heitmann, B. L. (2005). The effect of a health-promoting poster on the choice of whether to take the stairs or the escalator in two S-train stations in Copenhagen. *Ugeskr Laeger, 167*(10), 1160-1163.

Hankin, J. R., Firestone, I. J., Sloan, J. J., Ager, J. W., Sokol, R. J., & Martier, S. S. (1996). Heeding the alcoholic beverage warning label during pregnancy: Multiparae versus nulliparae. *Journal of Studies on Alcohol, 57*(2), 171-177.

Hare, T. A., Malmaud, J., & Rangel, A. (2011). Focusing attention on the health aspects of foods changes value signals in vmPFC and improves dietary choice. *Journal of Neuroscience, 31*(30), 11077-11087.

Harris, P. R., Mayle, K., Mabbott, L., & Napper, L. (2007). Self-affirmation reduces smokers' defensiveness to graphic on-pack cigarette warning labels. *Health Psychology, 26*(4), 437-446.

Hearn, M. D., Baranowski, T., Baranowski, J., Doyle, C., Smith, M., Lin, L. S., & Resnicow, K. (1998). Environmental influences on dietary behavior among children: Availability and accessibility of fruits and vegetables enable consumption. *Journal of Health Education, 29*(1), 26-32.

Hoerr, S. M., & Louden, V. A. (1993). Can nutrition information increase sales of healthful vended snacks? *Journal of School Health, 63*(9), 386-390.

Hofmann, W., Friese, M., & Wiers, R. W. (2008). Impulsive versus reflective influences on health behavior: A theoretical framework and empirical review. *Health Psychology Review, 2*(2), 111-137.

Hogarth, L. C., Mogg, K., Bradley, B. P., Duka, T., & Dickinson, A. (2003). Attentional orienting towards smoking-related stimuli. *Behavioural Pharmacology, 14*(2), 153-160.

Hopkins, D. P., Briss, P. A., Ricard, C. J., Husten, C. G., Carande-Kulis, V. G., Fielding, J. E., . . . Harris, K. W. (2001). Reviews of evidence regarding interventions to reduce tobacco use and exposure to environmental tobacco smoke. *American Journal of Preventive Medicine, 20*(Suppl.2), 16-66.

Horgen, K. B. (2001). *Promoting healthy food choices: A health message and economic incentive intervention.* Doctoral dissertation, Yale University, New Haven, CT, United States.

Houweling, S. T., Stoopendaal, J., Kleefstra, N., Meyboom-de Jong, B., & Bilo, H. J. (2005). [Use of stairs in a hospital increased by a sign near the stairs or the elevator]. *Ned Tijdschr Geneeskd, 149*(52), 2900-2903.

Howie, E. K., & Young, D. R. (2011). Step it up: A multicomponent intervention to increase stair use in a university residence building. *American Journal of Health Promotion, 26*(1), 2-5.

Hume, L., Dodd, C. A., & Grigg, N. P. (2003). In-store selection of wine: No evidence for the mediation of music? *Perceptual and Motor Skills, 96*(3,Pt.2), 1252-1254.

Hunt, M. K., Hebert, J. R., Sorensen, G., Harris, D. R., Hsieh, J., Morris, D. H., & Stoddard, A. M. (1993). Impact of a worksite cancer prevention program on eating patterns of workers. *Journal of Nutrition Education, 25*(5), 236-244.

Hunt, M. K., Lederman, R., Stoddard, A., Potter, S., Phillips, J., & Sorensen, G. (2000). Process tracking results from the treatwell 5-a-day worksite study. *American Journal of Health Promotion, 14*(3), 179-187.

Jason, L. A., & Clay, R. (1978). Modifying smoking behaviors in a barber shop. *Man-Environment Systems, 8*(1), 38-40.

Jason, L. A., & Frasure, S. (1980). Monitoring and changing behaviors in supermarket managers and consumers. *Man-Environment Systems, 10*(5-6), 288-290.

Jeffery, R. W., Pirie, P. L., Rosenthal, B. S., Gerber, W. M., & Murray, D. M. (1982). Nutrition education in supermarkets: An unsuccessful attempt to influence knowledge and product sales. *Journal of Behavioral Medicine, 5*(2), 189-200.

Kerr, J., Eves, F. F., & Carroll, D. (2001). Getting more people on the stairs: The impact of a new message format. *Journal of Health Psychology, 6*(5), 495-500.

Kimes, S. E., & Robson, S. K. A. (2004). The impact of restaurant table characteristics on meal duration and spending. *Cornell Hotel and Restaurant Administration Quarterly, 45*(4), 333-346.

King, A. C., Jeffery, R. W., Fridinger, F., Dusenbury, L., Provence, S., Hedlund, S. A., & Spangler, K. (1995). Environmental and policy approaches to cardiovascular disease prevention through physical activity: Issues and opportunities. *Health Education Quarterly, 22*(4), 499-511.

Kirschenbaum, D. S., & Tomarken, A. J. (1982). Some antecedents of regulatory eating by restrained and unrestrained eaters. *Journal of Abnormal Psychology, 91*(5), 326-336.

Kolodinsky, J., Green, J., Michahelles, M., & Harvey-Berino, J. R. (2008). The use of nutritional labels by college students in a food-court setting. *Journal of American College Health 57*(3), 297-302.

Koval, J. J., Aubut, J. A., Pederson, L. L., O'Hegarty, M., & Chan, S. S. (2005). The potential effectiveness of warning labels on cigarette packages: The perceptions of young adult Canadians. *Canadian Journal of Public Health, 96*(5), 353-356.

Kreuter, M. W., Brennan, L. K., Scharff, D. P., & Lukwago, S. N. (1997). Do nutrition label readers eat healthier diets? Behavioral correlates of adults' use of food labels. *American Journal of Preventive Medicine, 13*(4), 277-283.

Kristal, A. R., Goldenhar, L., Muldoon, J., & Morton, R. F. (1997). Evaluation of a supermarket intervention to increase consumption of fruits and vegetables. *American Journal of Health Promotion, 11*(6), 422-425.

Lammers, H. B. (2003). An oceanside field experiment on background music effects on the restaurant tab. *Perceptual and Motor Skills, 96*(3), 1025-1026.

Lebens, H., Roefs, A., Martijn, C., Houben, K., Nederkoorn, C., & Jansen, A. (2011). Making implicit measures of associations with snack foods more negative through evaluative conditioning. *Eating Behaviors, 12*(4), 249-253.

LeGuellec, H., Gueguen, N., Jacob, C., & Pascual, A. (2007). Cartoon music in a candy store: A field experiment. *Psychological Reports, 100*(3 Pt.2), 1255-1258.

Lewis, A. L., & Eves, F. F. (2011). Prompts to increase stair climbing in stations; The effect of message complexity. *Journal of Physical Activity & Health*.

Lewis, A. L., & Eves, F. F. (2011). Specific effects of a calorie-based intervention on stair climbing in overweight commuters. *Annals of Behavioral Medicine, 42*(2), 257-261.

Lieux, E. M., & Manning, C. K. (1992). Evening meals selected by college students: Impact of the foodservice system. *Journal of the American Dietetic Association, 92*(5), 560-566.

Lindman, R., Lindfors, B., Dahla, E., & Toivola, H. (1987). Alcohol and ambience: Social and environmental determinants of intake and mood. *Alcohol & Alcoholism, 1*(Suppl.), 385-388.

Long, S., Meyer, C., Leung, N., & Wallis, D. J. (2011). Effects of distraction and focused attention on actual and perceived food intake in females with non-clinical eating psychopathology. *Appetite, 56*(2), 350-356.

Luyben, P. D. (2009). Applied behavior analysis: Understanding and changing behavior in the community-a representative review. *Journal of Prevention & Intervention in the Community, 37*(3), 230-253.

Malouff, J., Schutte, N., Wiener, K., Brancazio, C., & Fish, D. (1993). Important characteristics of warning displays on alcohol containers. *Journal of Studies on Alcohol, 54*(4), 457-461.

Martinko, M. J., White, J., & Hassell, B. (1989). An operant analysis of prompting in a sales environment. *Journal of Organizational Behavior Management, 10*(1), 93-107.

Mayer, J. A., Brown, T. P., Heins, J. M., & Bishop, D. B. (1987). A multi-component intervention for modifying food selections in a worksite cafeteria. *Journal of Nutrition Education, 19*(6), 277-280.

McCullum, C., & Achterberg, C. L. (1997). Food shopping and label use behavior among high school-aged adolescents. *Adolescence, 32*(125), 181-197.

McElrea, H., & Standing, L. (1992). Fast music causes fast drinking. *Perceptual and Motor Skills, 75*(2), 362.

McKinnon, G. F., Kelly, J. P., & Robison, E. D. (1981). Sales effects of point-of-purchase in-store signing. *Journal of Retailing, 57*, 49-63.

Mellen, K. R. (2008). *Health behaviors of college students: Sample comparisons, physical activity associations, and a physical activity intervention.* Doctoral dissertation, University of Iowa, Ames, IA, United States.

Milich, R., Anderson, J., & Mills, M. (1976). Effects of visual presentation of caloric values on food buying by normal and obese persons. *Perceptual and Motor Skills, 42*(1), 155-162.

Miller, D. L., Bell, E. A., Pelkman, C. L., Peters, J. C., & Rolls, B. J. (2000). Effects of dietary fat, nutrition labels, and repeated consumption on sensory-specific satiety. *Physiology & Behavior, 71*(1-2), 153-158.

Mitchell, D. J., Kahn, B. E., & Knasko, S. C. (1995). There's something in the air: Effects of congruent or incongruent ambient odor on consumer decision making. *Journal of Consumer Research, 22*(2), 229-238.

Morrin, M. (2010). Scent marketing: An overview. In A. Krishna (Ed.), *Sensory marketing: Research on the sensuality of products* (pp. 75-86). New York, NY: Routledge/Taylor & Francis Group.

Muller, T. E. (1984). Buyer response to variations in product information load. *Journal of Applied Psychology, 69*(2), 300-306.

Mullin, G. E. (2010). Comment on guiding stars: The effect of a nutrition navigation program on consumer purchases at the supermarket. *Nutrition in Clinical Practice, 25*(5), 560-561.

Olander, E. K., & Eves, F. F. (2011). Effectiveness and cost of two stair-climbing interventions—less is more. *American Journal of Health Promotion, 25*(4), 231-236.

Palfai, T. P. (2006). Activating action tendencies: The influence of action priming on alcohol consumption among male hazardous drinkers. *Journal of Studies on Alcohol, 67*(6), 926-933.

Platkin, C. S. (2009). *Think before you eat: Calories and exercise equivalents presented on menus at point-of-choice.* Doctoral dissertation, Florida International University, University Park, FL, United States.

Ragneskog, H., Brane, G., Karlsson, I., & Kihlgren, M. (1996). Influence of dinner music on food intake and symptoms common in dementia. *Scandinavia Journal of Caring Science, 10*(1), 11-17.

Reibstein, D. J., Youngblood, S. A., & Fromkin, H. L. (1975). Number of choices and perceived decision freedom as a determinant of satisfaction and consumer behavior. *Journal of Applied Psychology, 60*(4), 434-437.

Roballey, T. C., McGreevy, C., Rongo, R. R., Schwantes, M. L., Steger, P. J., Wininger, M. A., & Gardner, E. B. (1985). The effect of music on eating behavior. *Bulletin of the Psychonomic Society, 23*(3), 221-222.

Roberto, C. A., Baik, J., Harris, J. L., & Brownell, K. D. (2011). Influence of licensed characters on children's taste and snack preferences. *Pediatrics, 126*(1), 88-93.

Rolls, B. J. (1979). How variety and palatability can stimulate appetite. *Nutrition Bulletin, 5*(2), 78-86.

Rolls, B. J., & Miller, D. L. (1997). Is the low-fat message giving people a license to eat more? *Journal of American College Nutrition, 16*(6), 535-543.

Ruiter, K. G. (2005). Saying is not (always) doing: Cigarette warning labels are useless. *European Journal of Public Health, 15*(3), 329.

Russell, W. D., Dzewaltowski, D. A., & Ryan, G. J. (1999). The effectiveness of a point-of-decision prompt in deterring sedentary behavior. *American Journal of Health Promotion, 13*(5), 257-259, ii.

Russell, W. D., & Hutchinson, J. (2000). Comparison of health promotion and deterrent prompts in increasing use of stairs over escalators. *Perceptual and Motor Skills, 91*(1), 55-61.

Salvy, S.-J., Jarrin, D., Paluch, R., Irfan, N., & Pliner, P. (2007). Effects of social influence on eating in couples, friends and strangers. *Appetite, 49*(1), 92-99.

Schmitz, M. F., & Fielding, J. E. (1986). Point-of-choice nutritional labeling: Evaluation in a worksite cafeteria. *Journal of Nutrition Education, 18*(2), s65-s68.

Shannon, C., Story, M., Fulkerson, J. A., & French, S. A. (2002). Factors in the school cafeteria influencing food choices by high school students. *Journal of School Health, 72*(6), 229-234.

Shaulov, N., & Lufi, D. (2009). Music and light during indoor cycling. *Perceptual and Motor Skills, 108*(2), 597-607.

Slaunwhite, J. M., Smith, S. M., Fleming, M. T., & Fabrigar, L. R. (2009). Using normative messages to increase healthy behaviours. *International Journal of Workplace Health Management, 2*(3), 231-244.

Smith, P. C., & Curnow, R. (1966). "Arousal hypothesis" and the effects of music on purchasing behavior. *Journal of Applied Psychology, 50*(3), 255-256.

Spiegel, T. A. (2000). Rate of intake, bites, and chews: The interpretation of lean-obese differences. *Neuroscience and Biobehavioral Reviews, 24*(2), 229-237.

Sproul, A. D., Canter, D. D., & Schmidt, J. B. (2003). Does point-of-purchase nutrition labeling influence meal selections? A test in an army cafeteria. *Military Medicine, 168*(7), 556-560.

Steenhuis, I., van Assema, P., van, B., Gerard, & Glanz, K. (2004). The effectiveness of nutrition education and labeling in Dutch supermarkets. *American Journal of Health Promotion, 18*(3), 221-224.

Stetter, F., Ackermann, K., Bizer, A., Straube, E. R., & Mann, K. (1995). Effects of disease-related cues in alcoholic inpatients: Results of a controlled "Alcohol Stroop" study. *Alcoholism, Clinical and Experimental Research, 19*(3), 593-599.

Stratton, G. (2000). Promoting children's physical activity in primary school: An intervention study using playground markings. *Ergonomics, 43*(10), 1538-1546.

Stubenitsky, K., Aaron, J., Catt, S., & Mela, D. (2000). The influence of recipe modification and nutritional information on restaurant food acceptance and macronutrient intakes. *Public Health Nutrition, 3*(2), 201-209.

Stutts, M. A., Zank, G. M., Smith, K. H., & Williams, S. A. (2011). Nutrition information and children's fast food menu choices. *Journal of Consumer Affairs, 45*(1), 52-86.

Thomas, D. W., & Smith, M. (2009). The effect of music on caloric consumption among nursing home residents with dementia of the Alzheimer's type. *Activities, Adaptation & Aging, 33*(1), 1-16.

Underwood, R. L. (1996). *The effect of package pictures on choice: An examination of the moderating effects of brand type, product benefits, and individual processing style.* Doctoral dissertation, Virginia Polytechnic Institute and State University, Blacksburg, VA, United States.

Walsh, L. M., Toma, R. B., Tuveson, R. V., & Sondhi, L. (1990). Color preference and food choice among children. *Journal of Psychology: Interdisciplinary and Applied, 124*(6), 645-653.

Webb, K. L., Solomon, L. S., Sanders, J., Akiyama, C., & Crawford, P. B. (2011). Menu labeling responsive to consumer concerns and shows promise for changing patron purchases. *Journal of Hunger & Environmental Nutrition, 6*(2), 166-178.

Webb, O. J., & Eves, F. F. (2007). Promoting stair climbing: Intervention effects generalize to a subsequent stair ascent. *American Journal of Health Promotion, 22*(2), 114-199.

Wiers, R. W., Houben, K., Smulders, F. T. Y., Conrod, P. J., & Jones, B. (2006). To drink or not to drink: The role of automatic and controlled cognitive processes in the etiology of alcohol-related problems. In R. W. H. J. Wiers & A. W. Stacy (Eds.), *Handbook of Implicit Cognition and Addiction* (pp. 339-361). London: Sage Publications, Inc.

William, S. T. (1983). Effects of food priming on instrumental acquisition and performance. *Learning and Motivation, 14*(1), 107-122.

Wryobeck, J., & Chen, Y. (2003). Using priming techniques to facilitate health behaviours. *Clinical Psychologist, 7*(2), 105-108.

Yalch, R., & Spangenberg, E. (1990). Effects of store music on shopping behavior. *Journal of Consumer Marketing, 7*(2), 55-63.

Zifferblatt, S. M., Wilbur, C. S., & Pinsky, J. L. (1980). A new direction for public health care: Changing cafeteria eating habits. *Journal of the American Dietetic Association, 76*(1), 15-20.

Appendix 1 – Search strategies, dates and yields

MEDLINE (OvidSP) search strategy - 1948 to June Week 5 2011

Yield = 418,040 records

1. exp diet/
2. exp diet therapy/
3. exp food/
4. exp beverages/
5. food habits/
6. food preferences/
7. fasting/
8. adolescent nutritional physiological phenomena/
9. elder nutritional physiological phenomena/
10. exp food industry/
11. exp hunger/
12. exp appetite regulation/
13. exp appetite/
14. exp digestion/
15. exp eating/
16. exp eating disorders/
17. exp child nutrition disorders/
18. exp infant nutrition disorders/
19. nutritional requirements/
20. nutritional status/
21. nutrition assessment/
22. nutrition disorders/
23. exp nutritive value/
24. (nutri$ or calori$ or diet$ or food$ or eat$ or eat or meal$ or snack$ or cook$ or restaurant$ or supermarket$ or cafe$).ti,ab.
25. (drink$ or beverage$).ti,ab.
26. exp Alcohol Drinking/
27. exp Alcohol-Related Disorders/
28. (drink$ or drunk$ or alcohol$ or beer$ or lager$ or wine$ or cider$ or alcopop$ or spirit$ or liquor$ or distilled beverage$ or whisky$ or whiskey$ or whiskies or schnapps or liqueur$ or brandy or brandies or gin$ or rum$ or tequila$ or vodka$).ti,ab.
29. exp Tobacco/
30. exp Smoking/
31. exp Smoking Cessation/
32. (cigar$ or smoking or smoke$ or tobacco$).ti,ab.
33. exp physical exertion/
34. exp human activities/
35. exp leisure activities/
36. exp locomotion/
37. exp physical education/
38. lifestyle/
39. sedentary lifestyle/

40. yoga/
41. fitness centers/
42. motor activity/
43. (physical$ adj5 (exercis$ or train$ or activit$ or fit$ or endur$)).ti,ab.
44. (aerobic adj5 (exercis$ or train$ or activit$ or fit$ or endur$)).ti,ab.
45. (strength$ adj5 (exercis$ or train$ or activit$ or fit$ or endur$)).ti,ab.
46. (flexib$ adj5 (exercis$ or train$ or activit$ or fit$ or endur$)).ti,ab.
47. (balanc$ adj5 (exercis$ or train$ or activit$ or fit$ or endur$)).ti,ab.
48. (exercise$ adj5 (train$ or activit$ or fit$ or endur$)).ti,ab.
49. ((occupation$ or work$ or recreation$2 or leisure or play or household or home or domestic or commut$3 or transport$) adj5 (energ$ or exercis$ or train$ or activit$ or fit$ or endur$)).ti,ab.
50. ((walk$3 or hike or hiking or climbing or run$3 or jog$3 or swim$1 or swimming or bicycl$3 or cycl$3 or bike$1 or biking or gym$ or rowing or canoe$ or kayak$ or sailing or windsurf$3 or surf$3 or diving or sport$3 or rollerblading or rollerskating or skating or skiing or yoga or pilates or calisthenics or (jump$3 adj rope$1) or (lift$3 adj weight$1) or gym$ or circuit or resistance or resilience or dance or dancing or fishing or hunting or shooting) adj5 (energ$ or exercis$ or train$ or activit$ or fit$ or endur$)).ti,ab.
51. (led walk$ or health walk$).ti,ab.
52. ((leisure or fitness) adj5 (centre$ or center$ or facilit$)).ti,ab.
53. (fitness adj class$).ti,ab.
54. (fitness adj (regime$ or program$)).ti,ab.
55. cardiorespiratory fitness.ti,ab.
56. aerobic capacity.ti,ab.
57. (intensity adj2 (rest or quiet or light or moderate or vigorous)).ti,ab.
58. ((car or cars or bus or buses or train or trains or transport$) and (energ$ or activit$ or exercis$)).ti,ab.
59. (active adj (travel$4 or transport$ or commut$)).tw.
60. ((promot$ or uptak$ or encourag$ or increas$ or start$ or adher$ or sustain$ or maintain$) adj5 gym$).ti,ab.
61. ((promot$ or uptak$ or encourag$ or increas$ or start$ or adher$ or sustain$ or maintain$) adj5 physical activit$).ti,ab.
62. ((promot$ or uptak$ or encourag$ or increas$ or start$ or adher$ or sustain$ or maintain$) adj5 (circuit$ or aqua$)).ti,ab.
63. ((promot$ or uptak$ or encourag$ or increas$ or start$ or adher$ or sustain$ or maintain$) adj5 (exercis$ or exertion or keep fit or fitness class or yoga or aerobic$)).ti,ab.
64. ((decreas$ or reduc$ or discourag$) adj5 (sedentary or deskbound or inactiv$)).ti,ab.
65. (exercis$ adj aerobic$).tw.
66. (physical$ adj5 (fit$ or train$ or activ$ or endur$)).tw.
67. (exercis$ adj5 (train$ or physical$ or activ$)).tw.
68. ((lifestyle or life-style) adj5 physical$).tw.
69. ((lifestyle or life-style) adj5 activ$).tw.
70. exp behavior/
71. behavior$.ti,ab.
72. behaviour$.ti,ab.
73. environment$.ti,ab.
74. consum$.ti,ab.
75. intake$.ti,ab.
76. perform$.ti,ab.
77. exp health promotion/
78. exp primary prevention/
79. exp attention/

80. exp visual perception/
81. exp feedback, psychological/
82. exp feedback, sensory/
83. exp perception/
84. exp illusions/
85. exp psychomotor performance/
86. (change$ or alter$ or adjust$ or modif$ or adapt$ or add$ or subtract$ or restrict$ or shrink$ or shrunk or extend$ or expand$ or supplement$ or improve$ or increas$ or higher or larger or longer or remov$ or constrain$ or restrain$ or limit$ or lower$ or reduc$ or decreas$ or smaller or greater or less$ or fewer or more or choice$ or choose or chose$ or option$).ti,ab.
87. or/1-69
88. or/70-85
89. and/86-88
90. limit 89 to (english language and humans)

Embase (OvidSP) search strategy - 1980 – 2011 Week 27

Yield = 402,410 records

1. exp diet/
2. exp diet therapy/
3. exp food/
4. exp beverage/
5. exp feeding behavior/
6. exp dietary intake/
7. exp food intake/
8. exp child nutrition/
9. exp food handling/
10. exp food processing/
11. exp hunger/
12. exp digestion/
13. exp eating disorder/
14. exp nutritional disorder/
15. nutritional requirement/
16. nutritional status/
17. nutritional assessment/
18. nutritional value/
19. (nutri$ or calori$ or diet$ or food$ or eat$ or meal$ or snack$ or cook$ or restaurant$ or supermarket$ or cafe$).ti,ab.
20. (drink$ or beverage$).ti,ab.
21. exp alcohol consumption/
22. exp alcohol abuse/
23. (drink$ or drunk$ or alcohol$ or beer$ or lager$ or wine$ or cider$ or alcopop$ or spirit$ or liquor$ or distilled beverage$ or whisky$ or whiskey$ or whiskies or schnapps or liqueur$ or brandy or brandies or gin$ or rum$ or tequila$ or vodka$).ti,ab.
24. exp tobacco/
25. exp smoking/
26. exp smoking cessation/
27. (cigar$ or smoking or smoke$ or tobacco$).ti,ab.
28. exp exercise/
29. exp physical activity/
30. exp human activities/
31. exp recreation/
32. exp leisure/
33. exp locomotion/
34. exp physical education/
35. exp lifestyle/
36. sedentary lifestyle/
37. exp fitness/
38. health center/
39. yoga/
40. motor activity/
41. (physical$ adj5 (exercis$ or train$ or activit$ or fit$ or endur$)).ti,ab.
42. (aerobic adj5 (exercis$ or train$ or activit$ or fit$ or endur$)).ti,ab.
43. (strength$ adj5 (exercis$ or train$ or activit$ or fit$ or endur$)).ti,ab.
44. (flexib$ adj5 (exercis$ or train$ or activit$ or fit$ or endur$)).ti,ab.
45. (balanc$ adj5 (exercis$ or train$ or activit$ or fit$ or endur$)).ti,ab.

46. (exercise$ adj5 (train$ or activit$ or fit$ or endur$)).ti,ab.
47. ((occupation$ or work$ or recreation$2 or leisure or play or household or home or domestic or commut$3 or transport$) adj5 (energ$ or exercis$ or train$ or activit$ or fit$ or endur$)).ti,ab.
48. ((walk$3 or hike or hiking or climbing or run$3 or jog$3 or swim$1 or swimming or bicycl$3 or cycl$3 or bike$1 or biking or gym$ or rowing or canoe$ or kayak$ or sailing or windsurf$3 or surf$3 or diving or sport$3 or rollerblading or rollerskating or skating or skiing or yoga or pilates or calisthenics or (jump$3 adj rope$1) or (lift$3 adj weight$1) or gym$ or circuit or resistance or resilience or dance or dancing or fishing or hunting or shooting) adj5 (energ$ or exercis$ or train$ or activit$ or fit$ or endur$)).ti,ab.
49. (led walk$ or health walk$).ti,ab.
50. ((leisure or fitness) adj5 (centre$ or center$ or facilit$)).ti,ab.
51. (fitness adj class$).ti,ab.
52. (fitness adj (regime$ or program$)).ti,ab.
53. cardiorespiratory fitness.ti,ab.
54. aerobic capacity.ti,ab.
55. (intensity adj2 (rest or quiet or light or moderate or vigorous)).ti,ab.
56. ((car or cars or bus or buses or train or trains or transport$) and (energ$ or activit$ or exercis$)).ti,ab.
57. (active adj (travel$4 or transport$ or commut$)).tw.
58. ((promot$ or uptak$ or encourag$ or increas$ or start$ or adher$ or sustain$ or maintain$) adj5 gym$).ti,ab.
59. ((promot$ or uptak$ or encourag$ or increas$ or start$ or adher$ or sustain$ or maintain$) adj5 physical activit$).ti,ab.
60. ((promot$ or uptak$ or encourag$ or increas$ or start$ or adher$ or sustain$ or maintain$) adj5 (circuit$ or aqua$)).ti,ab.
61. ((promot$ or uptak$ or encourag$ or increas$ or start$ or adher$ or sustain$ or maintain$) adj5 (exercis$ or exertion or keep fit or fitness class or yoga or aerobic$)).ti,ab.
62. ((decreas$ or reduc$ or discourag$) adj5 (sedentary or deskbound or inactiv$)).ti,ab.
63. (exercis$ adj aerobic$).tw.
64. (physical$ adj5 (fit$ or train$ or activ$ or endur$)).tw.
65. (exercis$ adj5 (train$ or physical$ or activ$)).tw.
66. ((lifestyle or life-style) adj5 physical$).tw.
67. ((lifestyle or life-style) adj5 activ$).tw.
68. exp behavior/
69. behavior$.ti,ab.
70. behaviour$.ti,ab.
71. consum$.ti,ab.
72. intake$.ti,ab.
73. perform$.ti,ab.
74. exp health promotion/
75. exp primary prevention/
76. exp attention/
77. exp perception/
78. exp feedback, psychological/
79. exp feedback, sensory/
80. exp illusion/
81. exp psychomotor performance/
82. (change$ or alter$ or adjust$ or modif$ or adapt$ or add$ or subtract$ or restrict$ or shrink$ or shrunk or extend$ or expand$ or supplement$ or improve$ or increas$ or higher or larger or longer or remov$ or constrain$ or restrain$ or limit$ or lower$ or reduc$ or decreas$

or smaller or greater or less$ or fewer or more or choice$ or choose or chose$ or option$).ti,ab.
83. or/1-67
84. or/68-81
85. and/82-84
86. limit 85 to (human and english language)

PsycINFO search strategy – 1806 to July 2011 Week 2

Yield = 150,325 records

1. exp diets/
2. exp food/
3. exp beverages/
4. food preferences/
5. food intake/
6. eating behavior/
7. drinking behavior/
8. nutrition/
9. exp appetite/
10. digestion/
11. dietary restraint/
12. binge eating/
13. eating attitudes/
14. "rumination (eating)"/
15. satiation/
16. exp nutritional deficiencies/
17. exp eating disorders/
18. dietary supplements/
19. (nutri$ or calori$ or diet$ or food$ or eat$ or meal$ or snack$ or cook$ or restaurant$ or supermarket$ or cafe$).ti,ab.
20. (drink$ or beverage$).ti,ab.
21. exp Alcohol Drinking Patterns/
22. exp Drinking Behavior/
23. exp Alcohol Abuse/
24. (drink$ or drunk$ or alcohol$ or beer$ or lager$ or wine$ or cider$ or alcopop$ or spirit$ or liquor$ or distilled beverage$ or whisky$ or whiskey$ or whiskies or schnapps or liqueur$ or brandy or brandies or gin$ or rum$ or tequila$ or vodka$).ti,ab.
25. exp Tobacco Smoking/
26. exp Smoking Cessation/
27. (cigar$ or smoking or smoke$ or tobacco$).ti,ab.
28. exp physical activity/
29. physical health/
30. physical education/
31. physical fitness/
32. physical endurance/
33. physical strength/
34. physical agility/
35. physical dexterity/
36. leisure time/
37. exp recreation/
38. exp lifestyle/
39. locomotion/
40. exp motor processes/
41. "activities of daily living"/
42. daily activities/
43. exp sports/
44. sports medicine/
45. athletic performance/

46. exp athletes/
47. athletic training/
48. energy expenditure/
49. (physical$ adj5 (exercis$ or train$ or activit$ or fit$ or endur$)).ti,ab.
50. (aerobic adj5 (exercis$ or train$ or activit$ or fit$ or endur$)).ti,ab.
51. (strength$ adj5 (exercis$ or train$ or activit$ or fit$ or endur$)).ti,ab.
52. (flexib$ adj5 (exercis$ or train$ or activit$ or fit$ or endur$)).ti,ab.
53. (balanc$ adj5 (exercis$ or train$ or activit$ or fit$ or endur$)).ti,ab.
54. (exercise$ adj5 (train$ or activit$ or fit$ or endur$)).ti,ab.
55. ((occupation$ or work$ or recreation$2 or leisure or play or household or home or domestic or commut$3 or transport$) adj5 (energ$ or exercis$ or train$ or activit$ or fit$ or endur$)).ti,ab.
56. ((walk$3 or hike or hiking or climbing or run$3 or jog$3 or swim$1 or swimming or bicycl$3 or cycl$3 or bike$1 or biking or gym$ or rowing or canoe$ or kayak$ or sailing or windsurf$3 or surf$3 or diving or sport$3 or rollerblading or rollerskating or skating or skiing or yoga or pilates or calisthenics or (jump$3 adj rope$1) or (lift$3 adj weight$1) or gym$ or circuit or resistance or resilience or dance or dancing or fishing or hunting or shooting) adj5 (energ$ or exercis$ or train$ or activit$ or fit$ or endur$)).ti,ab.
57. (led walk$ or health walk$).ti,ab.
58. ((leisure or fitness) adj5 (centre$ or center$ or facilit$)).ti,ab.
59. (fitness adj class$).ti,ab.
60. (fitness adj (regime$ or program$)).ti,ab.
61. cardiorespiratory fitness.ti,ab.
62. aerobic capacity.ti,ab.
63. (intensity adj2 (rest or quiet or light or moderate or vigorous)).ti,ab.
64. ((car or cars or bus or buses or train or trains or transport$) and (energ$ or activit$ or exercis$)).ti,ab.
65. (active adj (travel$4 or transport$ or commut$)).tw.
66. ((promot$ or uptak$ or encourag$ or increas$ or start$ or adher$ or sustain$ or maintain$) adj5 gym$).ti,ab.
67. ((promot$ or uptak$ or encourag$ or increas$ or start$ or adher$ or sustain$ or maintain$) adj5 physical activit$).ti,ab.
68. ((promot$ or uptak$ or encourag$ or increas$ or start$ or adher$ or sustain$ or maintain$) adj5 (circuit$ or aqua$)).ti,ab.
69. ((promot$ or uptak$ or encourag$ or increas$ or start$ or adher$ or sustain$ or maintain$) adj5 (exercis$ or exertion or keep fit or fitness class or yoga or aerobic$)).ti,ab.
70. ((decreas$ or reduc$ or discourag$) adj5 (sedentary or deskbound or inactiv$)).ti,ab.
71. (exercis$ adj aerobic$).tw.
72. (physical$ adj5 (fit$ or train$ or activ$ or endur$)).tw.
73. (exercis$ adj5 (train$ or physical$ or activ$)).tw.
74. ((lifestyle or life-style) adj5 physical$).tw.
75. ((lifestyle or life-style) adj5 activ$).tw.
76. exp Behavior/
77. exp Behavior Change/
78. exp Health Behavior/
79. behavior$.ti,ab.
80. behaviour$.ti,ab.
81. consum$.ti,ab.
82. intake$.ti,ab.
83. perform$.ti,ab.
84. exp Public Health/
85. exp Health Promotion/

86. exp Prevention/
87. exp Attention/
88. exp Perception/
89. exp Feedback/
90. exp Cognitive Processes/
91. (change$ or alter$ or adjust$ or modif$ or adapt$ or add$ or subtract$ or restrict$ or shrink$ or shrunk or extend$ or expand$ or supplement$ or improve$ or increas$ or higher or larger or longer or remov$ or constrain$ or restrain$ or limit$ or lower$ or reduc$ or decreas$ or smaller or greater or less$ or fewer or more or choice$ or choose or chose$ or option$).ti,ab.
92. or/1-75
93. or/76-90
94. and/91-93
95. limit 94 to (human and english language)

Web of Knowledge search strategy (Science Citation Index (EXPANDED), Social Science Citation Index, Conference Proceedings Citation Index- Science (CPCI-S), Conference Proceedings Citation Index- Social Science & Humanities (CPCI-SSH)) – All years – 19/7/2011

Yield = 168,250 records

(((TS=(nutri* or calori* or diet* or food* or eat* or meal* or snack* or cook* or restaurant* or supermarket* or café* or drink* or beverage* or drunk* or alcohol* or beer* or lager* or wine* or cider* or alcopop* or spirit* or liquor* or distilled beverage* or whisky* or whiskey* or whiskies or schnapps or liqueur* or brandy or brandies or gin* or rum* or tequila* or vodka* or cigar* or smoking or smoke* or tobacco* or exercis* or train* or activit* or fit* or endur* or aerobic* or flexib* or balance* or recreation* or leisure or walk* or hike or hiking or climbing or run* or jog* or swim* or swimming or bicycle* or cycl* or bike* or biking or gym* or rowing or canoe* or kayak* or sailing or windsurf* or surf* or diving or sport* or rollerblading or rollerskating or skating or skiing or yoga or pilates or calisthenics or jump* or weight*or circuit or aqua* or resistance or resilience or dance or dancing or fishing or hunting or shooting or led walk* or health walk*or aerobic capacity or exertion or keep fit or yoga or inactiv* or sedentary or deskbound)) AND (TS=(behav* or consum* or intake* or perform*))) AND (TS=(Change* or alter* or adjust* or modif* or adapt* or add* or subtract* or restrict* or shrink* or shrunk or extend* or expand* or supplement* or improve* or increas* or higher or larger or longer or remov* or constrain* or restrain* or limit* or lower* or reduc* or decreas* or smaller or greater or less* or fewer or more or choice* or choose or chose* or option*)) NOT (TS=(animal model* OR animal* OR animal experiment* OR animal disease model* OR laboratory animal*))) AND Language=(English)
Refined by: [excluding] Web of Science Categories=(ENGINEERING ELECTRICAL ELECTRONIC OR CARDIAC CARDIOVASCULAR SYSTEMS OR PEDIATRICS OR MATERIALS SCIENCE MULTIDISCIPLINARY OR CLINICAL NEUROLOGY OR PHYSICS APPLIED OR RADIOLOGY NUCLEAR MEDICINE MEDICAL IMAGING OR THERMODYNAMICS OR CHEMISTRY PHYSICAL OR BIOPHYSICS OR ENVIRONMENTAL SCIENCES OR GENETICS HEREDITY OR MEDICINE GENERAL INTERNAL OR MEDICINE RESEARCH EXPERIMENTAL OR BIOCHEMISTRY MOLECULAR BIOLOGY OR INSTRUMENTS INSTRUMENTATION OR ENGINEERING INDUSTRIAL OR PHARMACOLOGY PHARMACY OR MARINE FRESHWATER BIOLOGY OR BIOLOGY OR CHEMISTRY ANALYTICAL OR POLYMER SCIENCE OR FISHERIES OR CHEMISTRY APPLIED OR SUBSTANCE ABUSE OR ENGINEERING CHEMICAL OR WATER RESOURCES OR MANAGEMENT OR ENGINEERING MECHANICAL OR MECHANICS OR PHYSICS MULTIDISCIPLINARY OR PSYCHIATRY OR VETERINARY SCIENCES OR COMPUTER SCIENCE THEORY METHODS OR ENGINEERING BIOMEDICAL OR OPTICS OR NANOSCIENCE NANOTECHNOLOGY OR RESPIRATORY SYSTEM OR COMPUTER SCIENCE SOFTWARE ENGINEERING OR ORTHOPEDICS OR COMPUTER SCIENCE ARTIFICIAL INTELLIGENCE OR METALLURGY METALLURGICAL ENGINEERING OR OBSTETRICS GYNECOLOGY OR AUTOMATION CONTROL SYSTEMS OR ENERGY FUELS OR MICROBIOLOGY OR SURGERY OR TOXICOLOGY OR ASTRONOMY ASTROPHYSICS OR TELECOMMUNICATIONS OR PLANT SCIENCES OR AGRONOMY OR ONCOLOGY OR NUCLEAR SCIENCE TECHNOLOGY OR ENDOCRINOLOGY METABOLISM OR MATERIALS SCIENCE COATINGS FILMS OR UROLOGY NEPHROLOGY OR PHYSICS ATOMIC MOLECULAR CHEMICAL OR OCEANOGRAPHY OR BIOTECHNOLOGY APPLIED MICROBIOLOGY OR GEOSCIENCES MULTIDISCIPLINARY OR HEMATOLOGY OR PHYSICS CONDENSED MATTER OR REHABILITATION OR CHEMISTRY MULTIDISCIPLINARY OR ZOOLOGY OR DENTISTRY ORAL SURGERY MEDICINE OR COMPUTER SCIENCE HARDWARE ARCHITECTURE OR ENGINEERING MANUFACTURING OR AGRICULTURE MULTIDISCIPLINARY OR ELECTROCHEMISTRY OR PERIPHERAL

VASCULAR DISEASE OR CONSTRUCTION BUILDING TECHNOLOGY OR AGRICULTURE DAIRY ANIMAL SCIENCE OR CELL BIOLOGY OR BUSINESS OR ECOLOGY OR GASTROENTEROLOGY HEPATOLOGY OR SPECTROSCOPY OR COMPUTER SCIENCE INFORMATION SYSTEMS OR METEOROLOGY ATMOSPHERIC SCIENCES OR ENGINEERING AEROSPACE OR BIOCHEMICAL RESEARCH METHODS OR IMMUNOLOGY OR COMPUTER SCIENCE INTERDISCIPLINARY APPLICATIONS) AND [excluding] Web of Science Categories=(FORESTRY OR COMPUTER SCIENCE CYBERNETICS OR IMAGING SCIENCE PHOTOGRAPHIC TECHNOLOGY OR SOIL SCIENCE OR DERMATOLOGY OR MATHEMATICS APPLIED OR VIROLOGY OR OTORHINOLARYNGOLOGY OR AGRICULTURAL ECONOMICS POLICY OR CRYSTALLOGRAPHY OR PSYCHOLOGY EDUCATIONAL OR PATHOLOGY OR ACOUSTICS OR INFECTIOUS DISEASES OR PHYSICS MATHEMATICAL OR OPHTHALMOLOGY OR NURSING OR HORTICULTURE OR DEVELOPMENTAL BIOLOGY OR BUSINESS FINANCE OR GEOCHEMISTRY GEOPHYSICS OR MATERIALS SCIENCE CHARACTERIZATION TESTING OR ENTOMOLOGY OR MATERIALS SCIENCE PAPER WOOD OR MATHEMATICS INTERDISCIPLINARY APPLICATIONS OR ROBOTICS OR MATERIALS SCIENCE CERAMICS OR MEDICINE LEGAL OR GERIATRICS GERONTOLOGY OR REMOTE SENSING OR CRITICAL CARE MEDICINE OR MATERIALS SCIENCE TEXTILES OR CHEMISTRY ORGANIC OR ORNITHOLOGY OR MATERIALS SCIENCE COMPOSITES OR RHEUMATOLOGY OR PHYSICS NUCLEAR OR MINERALOGY OR ENGINEERING GEOLOGICAL OR REPRODUCTIVE BIOLOGY OR EDUCATION EDUCATIONAL RESEARCH OR CHEMISTRY MEDICINAL OR CHEMISTRY INORGANIC NUCLEAR OR MEDICAL INFORMATICS OR PSYCHOLOGY CLINICAL OR PARASITOLOGY OR ANATOMY MORPHOLOGY OR EMERGENCY MEDICINE OR LAW OR PHYSICS FLUIDS PLASMAS OR INFORMATION SCIENCE LIBRARY SCIENCE OR MINING MINERAL PROCESSING OR ANESTHESIOLOGY OR AGRICULTURAL ENGINEERING OR GERONTOLOGY OR TROPICAL MEDICINE OR EVOLUTIONARY BIOLOGY OR GEOLOGY OR MEDICAL LABORATORY TECHNOLOGY OR PHYSICS PARTICLES FIELDS OR SOCIAL WORK OR STATISTICS PROBABILITY)
Timespan=All Years. Databases=SCI-EXPANDED, SSCI, CPCI-S, CPCI-SSH.
Lemmatization=On

EconLit (EBSCO) search strategy – 1922 – 21/7/2011

Yield = 31,660 records

(TI(nutri* or calori* or diet* or food* or eat* or meal* or snack* or cook* or restaurant* or supermarket* or café* or drink* or beverage* or drunk* or alcohol* or beer* or lager* or wine* or cider* or alcopop* or spirit* or liquor* or distilled beverage* or whisky* or whiskey* or whiskies or schnapps or liqueur* or brandy or brandies or gin* or rum* or tequila* or vodka* or cigar* or smoking or smoke* or tobacco* or exercis* or train* or activit* or fit* or endur* or aerobic* or flexib* or balance* or recreation* or leisure or walk* or hike or hiking or climbing or run* or jog* or swim* or swimming or bicycle* or cycl* or bike* or biking or gym* or rowing or canoe* or kayak* or sailing or windsurf* or surf* or diving or sport* or rollerblading or rollerskating or skating or skiing or yoga or pilates or calisthenics or jump* or weight*or circuit or aqua* or resistance or resilience or dance or dancing or fishing or hunting or shooting or led walk* or health walk*or aerobic capacity or exertion or keep fit or yoga or inactiv* or sedentary or deskbound)) OR (AB(nutri* or calori* or diet* or food* or eat* or meal* or snack* or cook* or restaurant* or supermarket* or café* or drink* or beverage* or drunk* or alcohol* or beer* or lager* or wine* or cider* or alcopop* or spirit* or liquor* or distilled beverage* or whisky* or whiskey* or whiskies or schnapps or liqueur* or brandy or brandies or gin* or rum* or tequila* or vodka* or cigar* or smoking or smoke* or tobacco* or exercis* or train* or activit* or fit* or endur* or aerobic* or flexib* or balance* or recreation* or leisure or walk* or hike or hiking or climbing or run* or jog* or swim* or swimming or bicycle* or cycl* or bike* or biking or gym* or rowing or canoe* or kayak* or sailing or windsurf* or surf* or diving or sport* or rollerblading or rollerskating or skating or skiing or yoga or pilates or calisthenics or jump* or weight*or circuit or aqua* or resistance or resilience or dance or dancing or fishing or hunting or shooting or led walk* or health walk*or aerobic capacity or exertion or keep fit or yoga or inactiv* or sedentary or deskbound))

AND

(TI(behav* or consum* or intake* or perform*)) OR (AB(behav* or consum* or intake* or perform*))

AND

(TI(change* or alter* or adjust* or modif* or adapt* or add* or subtract* or restrict* or shrink* or shrunk or extend* or expand* or supplement* or improve* or increas* or higher or larger or longer or remov* or constrain* or restrain* or limit* or lower* or reduc* or decreas* or smaller or greater or less* or fewer or more or choice* or choose or chose* or option*)) OR (AB(change* or alter* or adjust* or modif* or adapt* or add* or subtract* or restrict* or shrink* or shrunk or extend* or expand* or supplement* or improve* or increas* or higher or larger or longer or remov* or constrain* or restrain* or limit* or lower* or reduc* or decreas* or smaller or greater or less* or fewer or more or choice* or choose or chose* or option*))

Database of promoting health effectiveness reviews (DoPHER) search strategy – 27/7/11

Yield = 1854 records

nutri* OR calori* OR diet* OR food* OR eat* OR meal* OR snack* OR cook* OR restaurant* OR supermarket* OR café* OR drink* OR beverage* OR drink* OR drunk* OR alcohol* OR beer* OR lager* OR wine* OR cider* OR alcopop* OR spirit* OR liquor* OR distilled beverage* OR whisky* OR whiskey* OR whiskies OR schnapps OR liqueur* OR brandy OR brandies OR gin* OR rum* OR tequila* OR vodka* OR cigar* OR smoking OR smoke* OR tobacco* OR exercis* OR train* OR activit* OR fit* OR endur* OR aerobic* OR flexib* OR balance* OR recreation* OR leisure OR walk* OR hike OR hiking OR climbing OR run* OR jog* OR swim* OR swimming OR bicycle* OR cycl* OR bike* OR biking OR gym* OR rowing OR canoe* OR kayak* OR sailing OR windsurf* OR surf* OR diving OR sport* OR rollerblading OR rollerskating OR skating OR skiing OR yoga OR pilates OR calisthenics OR jump* OR weight* OR circuit OR aqua* OR resistance OR resilience OR dance OR dancing OR fishing OR hunting OR shooting OR led walk* OR health walk* OR aerobic capacity OR exertion OR keep fit OR yoga OR inactiv* OR sedentary OR deskbound

AND

behav* OR consum* OR intake* OR perform*

AND

change* OR alter* OR adjust* OR modif* OR adapt* OR add* OR subtract* OR restrict* OR shrink* OR shrunk OR extend* OR expand* OR supplement* OR improve* OR increas* OR higher OR larger OR longer OR remov* OR constrain* OR restrain* OR limit* OR lower* OR reduc* OR decreas* OR smaller OR greater OR less* OR fewer OR more OR choice* OR choose OR chose* OR option*

This search was repeated at EPPI-Centre using same terms but with quotation marks placed around wildcard terms (i.e. those ending in *) on 11/8/11

SPORTDiscus (EBSCOhost) search strategy – 1985 – 28/7/11

Yield = 5334 records

(TI(nutri* or calori* or diet* or food* or eat* or meal* or snack* or cook* or restaurant* or supermarket* or café* or drink* or beverage* or drunk* or alcohol* or beer* or lager* or wine* or cider* or alcopop* or spirit* or liquor* or distilled beverage* or whisky* or whiskey* or whiskies or schnapps or liqueur* or brandy or brandies or gin* or rum* or tequila* or vodka* or cigar* or smoking or smoke* or tobacco* or exercis* or train* or activit* or fit* or endur* or aerobic* or flexib* or balance* or recreation* or leisure or walk* or hike or hiking or climbing or run* or jog* or swim* or swimming or bicycle* or cycl* or bike* or biking or gym* or rowing or canoe* or kayak* or sailing or windsurf* or surf* or diving or sport* or rollerblading or rollerskating or skating or skiing or yoga or pilates or calisthenics or jump* or weight*or circuit or aqua* or resistance or resilience or dance or dancing or fishing or hunting or shooting or led walk* or health walk*or aerobic capacity or exertion or keep fit or yoga or inactiv* or sedentary or deskbound)) OR (AB(nutri* or calori* or diet* or food* or eat* or meal* or snack* or cook* or restaurant* or supermarket* or café* or drink* or beverage* or drunk* or alcohol* or beer* or lager* or wine* or cider* or alcopop* or spirit* or liquor* or distilled beverage* or whisky* or whiskey* or whiskies or schnapps or liqueur* or brandy or brandies or gin* or rum* or tequila* or vodka* or cigar* or smoking or smoke* or tobacco* or exercis* or train* or activit* or fit* or endur* or aerobic* or flexib* or balance* or recreation* or leisure or walk* or hike or hiking or climbing or run* or jog* or swim* or swimming or bicycle* or cycl* or bike* or biking or gym* or rowing or canoe* or kayak* or sailing or windsurf* or surf* or diving or sport* or rollerblading or rollerskating or skating or skiing or yoga or pilates or calisthenics or jump* or weight*or circuit or aqua* or resistance or resilience or dance or dancing or fishing or hunting or shooting or led walk* or health walk*or aerobic capacity or exertion or keep fit or yoga or inactiv* or sedentary or deskbound))

AND

(TI(behav* or consum* or intake* or perform*)) OR (AB(behav* or consum* or intake* or perform*))

AND

(TI(change* or alter* or adjust* or modif* or adapt* or add* or subtract* or restrict* or shrink* or shrunk or extend* or expand* or supplement* or improve* or increas* or higher or larger or longer or remov* or constrain* or restrain* or limit* or lower* or reduc* or decreas* or smaller or greater or less* or fewer or more or choice* or choose or chose* or option*)) OR (AB(change* or alter* or adjust* or modif* or adapt* or add* or subtract* or restrict* or shrink* or shrunk or extend* or expand* or supplement* or improve* or increas* or higher or larger or longer or remov* or constrain* or restrain* or limit* or lower* or reduc* or decreas* or smaller or greater or less* or fewer or more or choice* or choose or chose* or option*))

AND

In English language

ASSIA – Applied Social Sciences Index and Abstracts (CSA) search strategy – 1987 - 28/7/11

Yield = 28,358 records

nutri* or calori* or diet* or food* or eat* or meal* or snack* or cook* or restaurant* or supermarket* or café* or drink* or beverage* or drunk* or alcohol* or beer* or lager* or wine* or cider* or alcopop* or spirit* or liquor* or distilled beverage* or whisky* or whiskey* or whiskies or schnapps or liqueur* or brandy or brandies or gin* or rum* or tequila* or vodka* or cigar* or smoking or smoke* or tobacco* or exercis* or train* or activit* or fit* or endur* or aerobic* or flexib* or balance* or recreation* or leisure or walk* or hike or hiking or climbing or run* or jog* or swim* or swimming or bicycle* or cycl* or bike* or biking or gym* or rowing or canoe* or kayak* or sailing or windsurf* or surf* or diving or sport* or rollerblading or rollerskating or skating or skiing or yoga or pilates or calisthenics or jump* or weight*or circuit or aqua* or resistance or resilience or dance or dancing or fishing or hunting or shooting or led walk* or health walk*or aerobic capacity or exertion or keep fit or yoga or inactiv* or sedentary or deskbound

AND

behav* or consum* or intake* or perform*

AND

change* or alter* or adjust* or modif* or adapt* or add* or subtract* or restrict* or shrink* or shrunk or extend* or expand* or supplement* or improve* or increas* or higher or larger or longer or remov* or constrain* or restrain* or limit* or lower* or reduc* or decreas* or smaller or greater or less* or fewer or more or choice* or choose or chose* or option*

AND

In English language

Cochrane library search strategy - Cochrane Database of Systematic Reviews, Database of Abstracts of Reviews of Effects, Health Technology Assessment Database, NHS Economic Evaluation Database – 2/8/11

Yield = 1380 records

nutri* or calori* or diet* or food* or eat* or meal* or snack* or cook* or restaurant* or supermarket* or café* or drink* or beverage* or drunk* or alcohol* or beer* or lager* or wine* or cider* or alcopop* or spirit* or liquor* or distilled beverage* or whisky* or whiskey* or whiskies or schnapps or liqueur* or brandy or brandies or gin* or rum* or tequila* or vodka* or cigar* or smoking or smoke* or tobacco* or exercis* or train* or activit* or fit* or endur* or aerobic* or flexib* or balance* or recreation* or leisure or walk* or hike or hiking or climbing or run* or jog* or swim* or swimming or bicycle* or cycl* or bike* or biking or gym* or rowing or canoe* or kayak* or sailing or windsurf* or surf* or diving or sport* or rollerblading or rollerskating or skating or skiing or yoga or pilates or calisthenics or jump* or weight*or circuit or aqua* or resistance or resilience or dance or dancing or fishing or hunting or shooting or led walk* or health walk*or aerobic capacity or exertion or keep fit or yoga or inactiv* or sedentary or deskbound

AND

behav* or consum* or intake* or perform*

AND

change* or alter* or adjust* or modif* or adapt* or add* or subtract* or restrict* or shrink* or shrunk or extend* or expand* or supplement* or improve* or increas* or higher or larger or longer or remov* or constrain* or restrain* or limit* or lower* or reduc* or decreas* or smaller or greater or less* or fewer or more or choice* or choose or chose* or option*
