# Supplementary material for: Altering micro-environments to change population health behaviour: towards an evidence base for choice architecture interventions
Source: BMC Public Health. 2013 Dec 21;13:1218. doi: 10.1186/1471-2458-13-1218 (PMC3881502; doi:10.1186/1471-2458-13-1218)
Supplement: Additional file 3 — Description of evidence by intervention type. [file 1471-2458-13-1218-S3.pdf]

Additional file 1 – Description of evidence by intervention type

Intervention type	Range of interventions identified	Summary of evidence
Primarily alter **properties** of objects or stimuli		
**AMBIENCE**<br>Interventions or manipulated factors that alter ambient, atmospheric or aesthetic aspects of the micro-environment surrounding a behaviour, but which are independent of or incidental to it. Differentiated from priming interventions as the latter category contains interventions which, whilst also independent of or incidental to a behaviour, are explicitly designed to activate non-conscious behavioural processes via their content	• Decoration, including colour, artwork, carpeting, use of different materials (e.g. painting stairwells or school playgrounds to enhance appeal);<br>• Brightness of lighting;<br>• Music volume, tempo (e.g. altering music played in food or alcohol purchasing or consumption environments);<br>• Distraction via television or radio	A wide variety of interventions was identified, mostly studied in field trials with a variety of outcomes. The majority of studies reported an effect of the intervention on behaviour
**FUNCTIONAL DESIGN**<br>The design or adaptation of the physical micro-environment, through changes to equipment or objects. Excludes labelling and presentation	• Demarcation of supermarket trolley space for fruit and vegetables;<br>• Supermarket trolleys necessitating increased effort to push;<br>• Supermarket trolleys with handle displays to indicate qualities of products;<br>• Trays versus lack of trays within cafeterias;<br>• Trolley versus basket use within supermarket environments;<br>• School classroom and desk design to encourage standing;<br>• Work desks adapted to include exercise opportunities;<br>• Painting of school playgrounds to indicate and create activity opportunities;<br>• Marking physical activity routes within existing micro-environments;<br>• Changes in amounts of seating available in consumption environments;<br>• Tableware design (e.g. plates with surface markings to suggest food consumption, such as portion control plates));<br>• Shape and size of plates and drinking glasses;<br>Type or size of eating utensils	A wide variety of interventions and study outcomes was identified. There was no consistent overall pattern of reported findings
**LABELLING**<br>Interventions that present labelling or	• Indication of typical reference portion;<br>• Nutritional labelling indicating quantity of nutrients contained (e.g. calories, fat	Most primary research involved field trials, with many

endorsement information specific to a product, either directly applied to the product itself or at point-of-choice (e.g. shelf-edge labelling, menu labelling)	or multi-nutrient, traffic light, Guideline Daily Amounts);<br>• Health warnings;<br>• Nutrient claims (e.g. 'low fat', 'reduced salt');<br>Product endorsements (e.g. sporting or celebrity endorsements)	studies reporting multiple outcomes. There was no consistent overall pattern of reported findings
**PRESENTATION**<br>Interventions that alter the sensory qualities or visual design of the product itself, including that actually consumed and its packaging, but not factors external to that. Excludes labelling	• Elements of packaging design including plain versus branded packaging, colour of packaging;<br>• Presenting different amounts of a product on packaging illustration (to alter consumption anchors);<br>• Characteristics of the consumed substance itself including manipulating variety in appearance through altering colour of food / way food is arranged, e.g. shaping or presenting food to enhance visual appeal	Wide variety of interventions identified. Most primary research was laboratory-based. There was no consistent overall pattern of findings, with some studies reporting an effect of the intervention on behaviour and others reporting no effect
**SIZING**<br>Interventions that change the size or quantity of the product itself. This can relate to size of the overall package, size of a portion served or contained within the overall package, or size of an individual unit within a portion	• Changes to package size, portion size or unit size of a product (these may be interchangeable or synonymous depending on the targeted product)	Mostly laboratory-based studies, with the majority reporting an effect of portion size on consumption, a conclusion that is also supported by specific systematic review evidence
Primarily alter **placement** of objects or stimuli		
**AVAILABILITY**<br>Interventions that alter availability through adding behavioural options, or changing capacity for engagement with behavioural options, providing broadly equivalent options/behaviours from the previous potential behaviour set remain available within the micro-environment	• Increasing available healthier food options via e.g. increasing variety of healthy options or providing more or less of specific nutrients or foods in available options, such as introducing more low-fat items in vending machines;<br>• Altering quantity of specific available products within a given environment (i.e. stockpiling);<br>• Altering availability of stairs or of alternatives to stair use i.e. escalators and lifts, altering lift use speed, increasing stair width, implementing 'skip-stop' lifts that do not serve every floor of a building	Primary studies were identified in diet and physical activity only, mostly studied in field trials concerning food and drink options in restaurants and vending machines and the use of stairs or lifts. The majority of studies reported an effect of the intervention on behaviour, although this was often

		complicated by the concurrent implementation of multiple interventions or the assessment of multiple outcomes
**PROXIMITY**<br>Interventions that facilitate engagement with available behavioural options by making such options more immediately salient or reducing required effort, primarily through altering proximity, but also accessibility or visibility	• Altering layouts within micro-environments to increase or decrease distance of products from routes of passage or seating, e.g. placing area for dispensing certain foods at greater distance;<br>• Placing products out of immediate sight or making them less visible, e.g. displayed within or behind opaque versus transparent materials;<br>• Changing item positions within a food menu;<br>• Making purchasing process more or less convenient for certain products;<br>• Highlighting potential swaps for healthier products within a shopping environment	All primary studies identified related to dietary behaviours, with a variety of outcomes reflecting changes in consumption, purchasing or selection of products. The majority of studies reported an effect of the intervention on behaviour
Alter both **properties and placement** of objects or stimuli		
**PRIMING**<br>Interventions that involve the placement of incidental cues, objects or stimuli within the micro-environment, or within material that a person is exposed to, to induce or influence an non-conscious behavioural response via the activation of e.g. semantic relationships or associative processes	• Priming of dieting goals via recipe placed on shop door;<br>• Placing decorative objects within restaurants to prime consumption of specific food types;<br>• Placing smoking-related objects in a room incidental to an assigned task;<br>• Content of music to evoke associations (origin of music, lyrical content);<br>• Décor and table furniture of restaurants to suggest ethnic theme or elicit associations with snack versus meal consumption	Primary studies of a wide variety of interventions were identified within all four behavioural domains, with a variety of outcomes. The majority of studies reported an effect of the intervention on behaviour
**PROMPTING**<br>Interventions that contain standardised explicit verbal, visual and/or numeric information intending to promote or raise awareness of, and thus motivation for, a given behaviour. Differentiated from labelling interventions by not being specific to the content of individual products and providing	• Promotional signage and materials including posters, screens, audio, public announcements;<br>• Motivational prompts (signs, posters, footprint symbols) for stair versus lift or escalator use including on surfaces proximal to stairwells and on stair risers	Primary studies were identified within all four behavioural domains, using field-based (mainly time-series) designs. In relation to physical activity, the majority of studies reported an effect of the intervention on behaviour, supported by

more general motivational prompting		specific systematic review evidence. In relation to diet, many studies reported multiple outcomes and there was no consistent overall pattern of findings
